# Predicting p*K*_a_ of flexible polybasic tetra-aza macrocycles†

**DOI:** 10.1039/d5ra01015b

**Published:** 2025-04-07

**Authors:** Tatum K. Harvey, Kristof Pota, Magy M. Mekhail, David M. Freire, Donatus A. Agbaglo, Benjamin G. Janesko, Kayla N. Green

**Affiliations:** a Department of Chemistry & Biochemistry, Texas Christian University 2800 S. University Dr. Fort Worth TX 76129 USA kayla.green@tcu.edu b.janesko@tcu.edu; b Department of Chemistry, University of California Irvine USA mamekhai@uci.edu

## Abstract

We present physics-based p*K*_a_ predictions for a library of tetra-aza macrocycles. These flexible, polybasic molecules exhibit highly charged states and substantial prototropic tautomerism, presenting a challenge for p*K*_a_ prediction. Our computational protocol combines CREST/xTB conformational sampling, density functional theory (DFT) refinement in continuum solvent, and a linear empirical correction (LEC). This approach predicts known tetra-aza macrocycle p*K*_a_ to within a root-mean-square deviation 1.2 log units. This approach also provides reasonable predictions for the most stable protomers at different pH. We use this protocol to predict p*K*_a_ values for four novel, synthetically achievable, previously un-synthesized tetra-aza macrocycles, providing new leads for future experiments.

## Abstract 2

We present physics-based p*K*_a_ predictions for a library of tetra-aza macrocycles.

## Graphical abstract

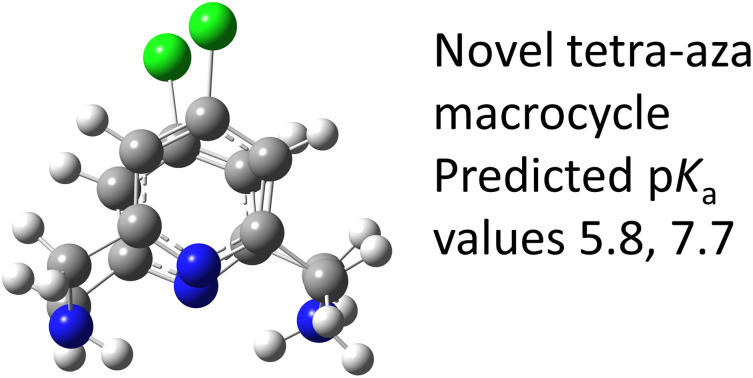

## 1. Introduction

Predicting p*K*_a_ and pH-dependent speciation (prototropic tautomerization) was^1^ and is^2^ a critical component of computational medicinal chemistry.^3,4^ The pH-dependent speciation of drug molecules governs their solubility,^5^ docking poses,^6^ and membrane permeability.^7^ Modern density functional theory (DFT) approximations can routinely predict gas-phase p*K*_a_ of rigid small molecules possessing a single acid/base site.^8^ Predicting aqueous-phase p*K*_a_ requires a model for solvent.^9–12^ Predicting aqueous-phase p*K*_a_ of large, flexible, polybasic molecules remains a challenge. Polybasic molecules are abundant in pharmaceutical design, possess prototropic tautomerism,^13,14^ and can be highly charged in solution.^15^ Flexible macrocycles can access multiple conformations within each tautomer (chameleonicity).^16^ Molecules that are both flexible and polybasic are particularly challenging for p*K*_a_ prediction.

### 1.1. Tetra-aza macrocycles

**Fig. 1:**
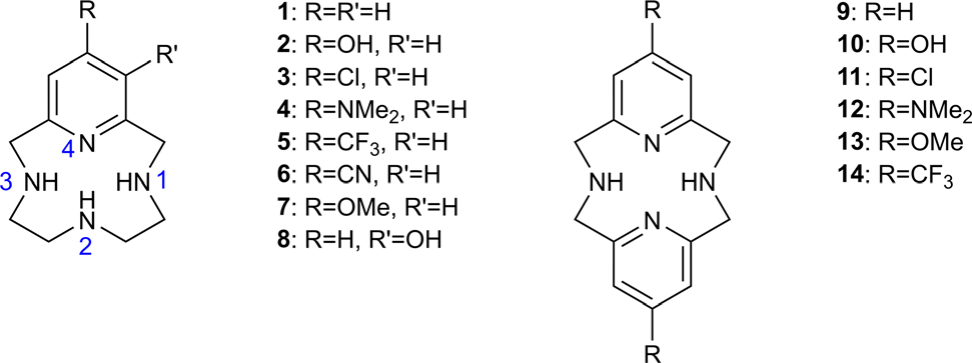
Structures of existing molecules 1–10 and previously un-synthesized molecules 11–14. Atom numbering is indicated in blue.

Our goal in this work is to validate and use a computational protocol capable of predicting the p*K*_a_ and pH-dependent speciation of flexible, polybasic, tetra-aza macrocycles (Fig. 1). Tetra-aza macrocycles combine high water solubility, tunable metal binding,^17,18^ and antioxidant activitiy.^19,20^ Tetra-aza macrocycles have been employed as catalysts, luminescent bioprobes,^21^ MRI contrast agents,^22^ and drug candidates for treating oxidative stress.^23,24^ Some tetra-aza macrocycles dis-aggregate amyloids.^25^ Other tetra-aza macrocycles have demonstrated activity as Nrf2 activators.^26^ The Green group has devised synthetic methods capable of accessing a broad range of substituted tetra-aza macrocycles, and has advanced these molecules' application as potential therapeutics. Initial experimental studies include the measured p*K*_a_ of ten substituted macrocycles (molecules 1–10) and the protonation sites of molecules 1 and 2 (Table 1).^23,27^ These molecules possess between four and six acid/base sites and are positively charged at physiological pH.^23,27^ Proton NMR methods can be used to accurately measure the p*K*_a_ and the dominant tautomers at varying pH. These studies demonstrate that substitution significantly changes the p*K*_a_.^23,27,28^ Synthesis and testing of the thousands of synthetically accessible tetra-aza macrocycle derivatives represents a major technical hurdle. Reliable predictions of the structures, docking poses, solubility, membrane permeability, and other properties of un-synthesized tetra-aza macrocycles could significantly accelerate development of lead compounds for the applications discussed above. Reliable prediction of p*K*_a_ and charge state at physiological pH is a prerequisite for such predictions. The broad range of possible synthetically accessible macrocycles, and the demonstrated impact of chemical substitution on p*K*_a_ and protonation site, motivate the use of physics-based p*K*_a_ prediction protocols for tetra-aza macrocycles.

**Table 1:** Comparison of computed and measured p*K*_a_ values for macrocycles 1–10

Molecule	p*K*_a_	Experiment	QM	QM + LEC
1	1	11.37^a^	10.73	9.63
	2	8.22^a^	3.83	6.15
	3	1.61^a^	−1.22	3.60
2	1	11.56^b^	14.10	11.34
	2	9.05^b^	9.90	9.22
	3	5.45^b^	3.41	5.94
	4	1.68^b^	−1.90	3.26
3	1	10.50^a^	12.25	10.40
	2	7.27^a^	1.73	5.09
	3	1.37^a^	−6.88	0.74
4	1	10.54^a^	13.35	10.96
	2	8.27^a^	8.04	8.28
	3	1.73^a^	−4.04	2.18
5	1	11.14^a^	10.06	9.30
	2	7.47^a^	3.02	5.74
6	1	10.6^a^	12.28	10.42
	2	7.00^a^	3.90	6.18
	3	0.85^a^	−3.71	2.34
7	1	10.32^c^	10.52	9.53
	2	8.00 c	5.87	7.18
	3	1.75^c^	−2.15	3.13
8	1	11.16^b^	13.92	11.25
	2	9.46^b^	11.13	9.84
	3	6.91^b^	3.61	6.04
	4	2.17^b^	−6.47	0.95
9	1	8.27^d^	8.50	8.50
	2	7.36^d^	5.62	7.05
10	1	11.31^d^	16.82	12.71
	2	9.35^d^	15.58	12.08
	3	5.25^d^	3.75	6.11
	4	4.21^d^	2.40	5.43
	5	0.98^d^	−5.16	1.61
RMSD			3.88	1.21

a Ref. 31 and 32.

b Ref. 27.

c Ref. 32.

d Ref. 23.

### 1.2. Physics-based p*K*_a_ prediction

Physics-based computational protocols for p*K*_a_ prediction explicitly treat conformational change, making them suitable for capturing the interplay of conformation, p*K*_a_, and prototropic tautomerism in tetra-aza macrocycles. The Statistical Assessment of Modeling of Proteins and Ligands (SAMPL) physical property challenges provide a snapshot of the state-of-the-art in physics-based p*K*_a_ prediction.^29^ In the SAMPL6 challenge, participants predicted the Gibbs free energies and p*K*_a_ values of 22 *N*-acetylsulfonamides possessing up to three acid/base sites. The most accurate physics-based quantum mechanical (QM) methods combined conformational sampling, DFT, model solvent, and a linear empirical correction (QM + LEC). The best QM + LEC methods gave root-mean-square errors (RMSE) below 0.7 log units.^2^ In the SAMPL7 challenge, participants predicted p*K*_a_ values for 22 *N*-acylsulfonamides and related bioisosteres. Participants determined experimental values (“macroscopic p*K*_a_”) from the computed free energies of individual protonation tautomers (“microscopic p*K*_a_”). Only one physics-based method gave RMSE below 1 log unit.^29^ Moreover, there was significant disagreement as to which microscopic transitions produced the measured p*K*_a_, *i.e.,* which prototropic tautomers were most stable at each pH. In the SAMPL8 challenge, participants considered more diverse compounds including several polybasic species. A QM protocol combining conformational sampling, DFT, and the COSMO-RS solvation model yielded RMSE 1.65 log units when using assignment based on the experimental transition curves.^30^

### 1.3. Overview

We use state-of-the-art physics-based computational protocols to predict the p*K*_a_ of previously un-synthesized polybasic tetra-aza macrocycles. We employ protocols similar to those applied in the SAMPL6 and SAMPL7 challenges. We use these protocols to predict the p*K*_a_ and pH-dependent speciation of molecules 11–14, four previously un-synthesized tetra-aza macrocycles. Table 2 reports the final predictions. We validate these protocols against the experimental p*K*_a_ and pH-dependent speciation behavior of previously synthesized macrocycles. Table 1 shows the experimental p*K*_a_ values and predictions of our preferred protocol. This protocol provides useful accuracy consistent with results from the SAMPL challenges. In an attempt to further refine our predictions, we systematically test the effects of the different approximations employed. We find that solvation of highly charged species is a significant source of remaining errors. Future studies will experimentally test the predictions for molecules 11–14 and will use this protocol to predict p*K*_a_ values of other tetra-aza macrocycles.

**Table 2:** Predicted p*K*_a_ values for macrocycles 11–14

Molecule	p*K*_a_	Prediction
11	1	7.73
	2	5.83
12	1	10.06
	2	9.11
13	1	8.52
	2	7.14
14	1	6.67
	2	6.39

## 2. Methods

Most calculations in this work use a common computational workflow. For each molecule of interest, the user provides a three-dimensional structure in which all N protonation sites are occupied, along with a list of the N exchangeable protons (ESI Fig. SI1,† blue nitrogen atoms). To illustrate, molecule 1 starts with a structure of the charge +4 species with *N* = 4 protonated nitrogen atoms. The approach is “black-box” in that any molecule of interest may be treated, as long as the protonated 3D structure and possible protonation sites are known. A Python script using the itertools library automatically generates all possible protonation isomers by removing all possible combinations of exchangeable protons. To illustrate, molecule 1 gives 14 protonation isomers spanning five charge states: one neutral isomer, four protonation isomers with charge +1, six isomers with charge +2, four with charge +3, and one (the original input structure) with charge +4. Spatial symmetry is not used: for example, two of the charge +1 structures for molecule 1 are redundant, with protonation on either of the two symmetry-equivalent secondary amines adjacent to the pyridine.

The computed three-dimensional structure of each protonation isomer is used as input for a metadynamics and molecular dynamics simulation using the Conformer-Rotamer Ensemble Sampling Tool (CREST).^33^ CREST calculations use the GFN2-xTB tight binding Hamiltonian,^34^ the generalized Born with surface area contributions (GBSA) continuum model for water solvent,^35^ and the iMTD-GC metadynamics-based exploration of conformational space employing a biasing potential expressed with the root-mean-square deviation in Cartesian space as a metric for the collective variables.^36^ The five lowest-energy conformations generated by CREST are refined using a Gaussian 16 DFT geometry optimization and free energy calculation.^37^ These calculations use density functional theory in an atomic orbital (AO) basis set to treat the molecule, and a continuum solvent model to treat the water solvent. Solvent is modeled using either the SMD or IEFPCM continuum models for water solvent, employing the default parameters for, *e.g.*, solvent static dielectric constant.^38,39^ The calculations also use 6-31G(d) or def2-TZVP atomic orbital basis sets,^40,41^ and the B3LYP^42,43^ or M06-2X^44^ exchange-correlation functionals. All geometry optimizations and vibrational frequency calculations are performed in continuum solvent.^45,46^ Gibbs free energies are taken to be the free-particle-rigid-rotor-harmonic-oscillator free energy of the lowest energy conformation of each protonation state. All calculations treat temperature *T* = 298.15 K. The workflow is implemented as a set of Python and Perl scripts which write and process CREST and Gaussian input and output files. This implementation is freely available at the Janesko group GitHub site.

QM calculations compute the p*K*_a_ as ΔG*/*RT*(ln 10). For any acid HA^*n*^ with charge *n*, we compute ΔG* as the Gibbs free energy of the dissociation reaction

1 HA^*n*^ (aq) ⇄ H^+^ (aq) + A^*n*−1^ (aq)

2 G* = *G*_comput_ (A^*n*−1^, aq) + *G*_expt_ (H^+^, aq) − *G*_comput_ (HA^*n*^, aq)

The computed Gibbs free energies of HA^*n*^ and A^*n*−1^ are taken directly from the Gaussian output files. *G*_expt_ (H^+^, aq) denotes the experimental Gibbs free energy of the hydrated proton at 298.15 K and standard state concentration 1 mol L^−1^. This is computed as described in ref. 47.

3 *G*_expt_(H^+^, aq) = *G*^0^_g_ (H^+^) + Δ*G*^1atm → 1 M^ + Δ*G*_aq,solv_ (H^+^)

The standard Gibbs energy of the gas phase proton, treated as an ideal gas at gas-phase concentration 1 bar, is taken as *G*^0^_g_ (H^+^) = −6.28 kcal mol^−1^. This is calculated as *G*^0^_g_= H^0^_g_ − TS^0^_g_ where H^0^_g_ = (5/2) *RT* and *S*^0^_g_ = 26.05 cal (mol^−1^·K^−1^).^48^ The factor Δ*G*^1atm → 1 M^ = 1.89 kcal mol^−1^ accounts for change of the state from 1 bar to 1 mol L^−1^. The aqueous solvation free energy of the proton at concentration 1 mol L^−1^ Δ*G*_aq,solv_ (H^+^) = −265.9 kcal mol^−1^ is taken from the work of Tissandier *et al.*,^49^ corrected to treat an ideal gas at a gas-phase concentration of 1 mol L^−1^ dissolving as an ideal solution at a liquid-phase concentration of 1 mol L^−1^ as discussed by Kelley *et al.*^50^ QM + LEC calculations employ a linear empirical correction (LEC)

4 p*K*_a_ (corrected) = *a* × p*K*_a_ (computed) + *b*

Parameters *a* and *b* are obtained as a best-fit to the experimental data in Table 1. In addition to the tests of basis set, continuum solvent, and exchange-correlation functional discussed above, several test calculations treat other aspects of the computational workflow. Test calculations using only computed solvation free energies compute ΔG* as

5 HA^*n*^ (aq) + H_2_O (aq) ⇄ H_3_O^+^ (aq) *+* A^*n*−1^ (aq)

6 ΔG* = *G*_comput_ (A^*n*−1^, aq) + Δ*G*_expt_ (H_3_O^+^, aq) − Δ*G*_comput_ (HA^*n*^, aq) − Δ*G*_comput_ (H_2_O, aq)

Other test calculations combine eqn (5) and (6) with the pK-Yay correction.^51^ Test calculations employing explicit + implicit water solvent use the QCG quantum cluster growth approach to determine the conformations of the added water molecules.^52^ This approach uses combined metadynamics and molecular dynamics with the intermolecular force field xTB-IFF^53^ to grow molecule-solvent clusters, one solvent molecule at a time. The DFT approximations are tested by comparison to the CBS-QB3 *ab initio* composite approach as implemented in Gaussian 16.^54^ We compare DFT and CBS-QB3 calculations on the aqueous-phase p*K*_a_ of six small molecules structurally similar to the tetra-aza macrocycles: dimethyl amine DMA, trimethyl amine TMA, pyridine, 3-hydroxy pyridine 3HP, 4-hydroxy pyridine 4HP, and phenol. Experimental p*K*_a_ of these molecules are taken from the Bordwell tables.^34,55–57^

## 3. Results

### 3.1 Validation

**Fig. 2:**
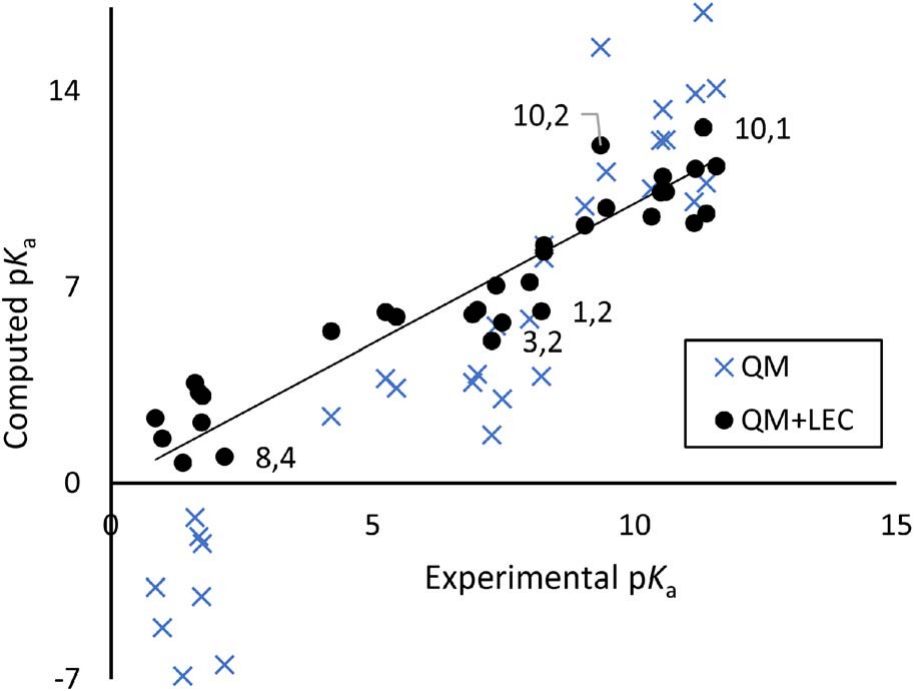
Scatter plot of experimental *vs.* computed p*K*_a_ from Table 1. Selected points are labelled as “molecule, p*K*_a_ (*n*)”.

Table 1 compares measured p*K*_a_ values to those computed with our preferred QM and QM+LEC method: CREST structure optimization, M06-2X/def2-TZVP structure refinement with the SMD continuum solvent model, and p*K*_a_ computed from the experimental proton solvation free energy. Fig. 2 shows a scatter plot of experimental *vs.* QM+LEC predicted p*K*_a_. Uncorrected QM results give root-mean-square-deviation (RMSD) 3.88 log units. LEC significantly improves the results, with RMSD 1.21 log units comparable to those seen in previous SAMPL challenges. We regard this as significant in that the present work includes flexible species with up to six exchangeable protons and measured p*K*_a_ spanning a range of ten log units.

**Fig. 3:**
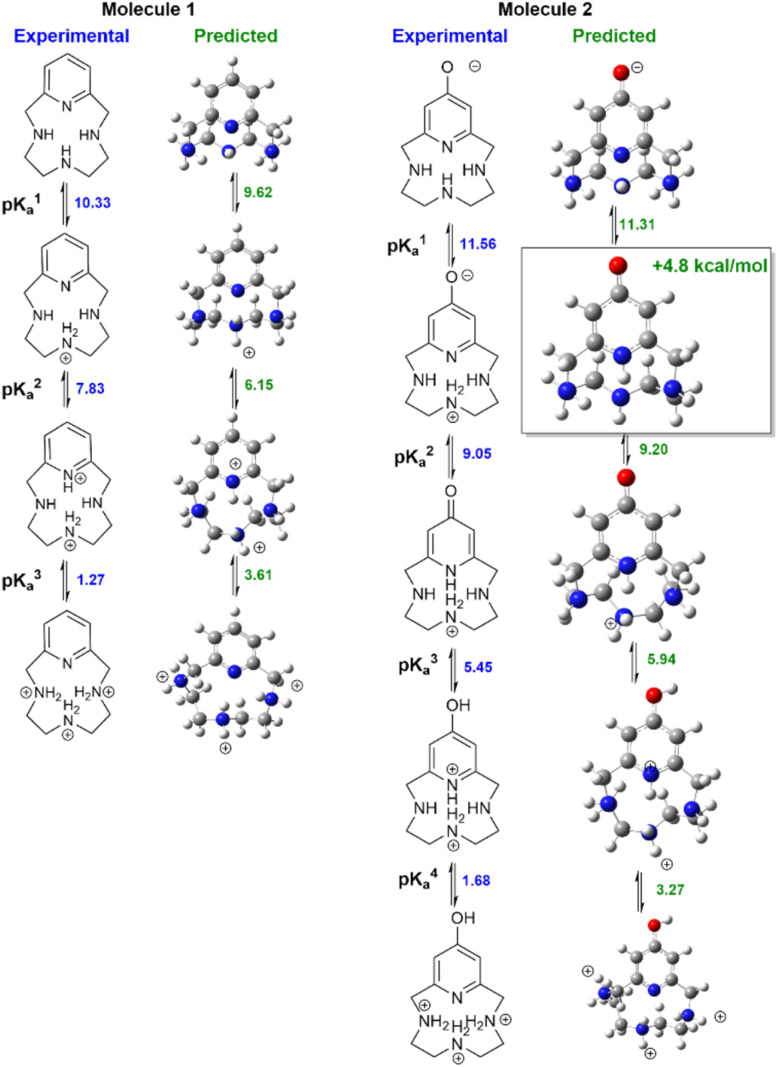
Speciation of molecules 1–2. Left columns show experimentally determined stable protomers, right columns show the predicted stable protomers.

Fig. 3 compares measured lowest-energy protomers to those computed with our preferred QM+LEC method. The predicted most stable protomers show excellent agreement with experiment. The charge +1 state of molecule 1 is correctly predicted to be protonated at nitrogen N2 (see atom labeling in Fig. 1). The charge +2 state is correctly predicted to be protonated at N2 and pyridine nitrogen N4. The charge +3 state is correctly predicted to show tautomerism, protonated at nitrogen N1, N2, N3 and not protonated at pyridine nitrogen N4.

Our protocol also provides reasonable predictions for molecule 2. The charge +1 state is correctly predicted to have the 4-pyridone structure with protonation at N2. The charge +3 state is correctly predicted to be protonated at oxygen, yielding protonated 4-hydroxy-pyridine and protonation at N2. The charge +2 state is correctly predicted to show a significant rearrangement with protonation at N1–N3. The only discrepancy is that the most stable charge-neutral state is predicted to be the 4-pyridone tautomer. This is predicted to be 4.8 kcal mol^−1^ more stable than the zwitterion assigned in ref. 27. Calculations of NMR chemical shifts upon protonation confirm that the zwitterionic structure best reproduces experimental NMR titration data (ESI Table SI1†). Calculations suggest that the zwitterionic structure has a degree of proton sharing between N2 and N4 (ESI Fig. SI2†). The suggestion of proton sharing is consistent with the basicity of the moieties involved: the measured p*K*_a_ of 4-pyridone in water is 11.09 at 20° (ref. 58 ), significantly higher than the measured p*K*_a_ of aliphatic secondary amines (dimethylamine 10.922 at 20° ref. 59) and quite close to the measured first p*K*_a_ of molecule 2. We regard this agreement as particularly significant given the discrepancies in predicted protonation tautomers seen in previous SAMPL challenges.^29^

### 3.2. Other computational protocols

Table 3 reports RMSD in p*K*_a_ computed with different choices of approximate density functional, basis set, solvent model, and choice of Δ*G*. Here “TZP” denotes the def2-TZVP basis set and "DZ" denotes the 6-31+G basis set. For each choice, we report RMSD from QM and QM + LEC calculations and include the LEC parameters *a*, *b* (eqn (4)). The choice of density functional has a relatively small effect, with B3LYP and M06-2X giving comparable QM + LEC RMSD. The basis set has a modest effect, with TZP giving an ∼0.1 log unit improvement over DZ. The solvent model significantly affects the uncorrected results, with IEFPCM giving RMSD values 6–8 log units larger than those with SMD. This is true for both B3LYP and M06-2X DFT calculations. This appears to be a systematic error corrected by the LEC. QM+LEC RMSD are comparable between SMD and IEFPCM models. The source of this systematic error appears to involve an insufficient charge screening leading to progressive destabilization of highly charged species. To illustrate this in detail, we consider the difference between the first and fourth p*K*_a_ for molecule 2. Experiments give a difference of 9.9 log units. M06-2X/def2-TZVP calculations give a difference of 33 log units for the IEFPCM model, but a difference of only 16 log units for the SMD model. This difference is reflected in the slope a of the LEC, which is around 0.5 for SMD solvent and around 0.3 for IEFPCM solvent. QM calculations using only computed solvation free energies (eqn (3) and (4)) give a much larger RMSD, a result which is improved by the pK-Yay correction. Eqn (3) and (4) and the pK-Yay correction do not change the results obtained after LEC.

**Table 3:** RMSD in p*K*_a_ (log units) computed with different model chemistries (density functional, basis set, continuum solvent), different choices of Δ*G*, and linear empirical corrections LEC. Column “Eqn (4) LEC *a*, *b*” denotes the optimum values of the fitted parameters in the LEC eqn (4)

	Δ*G*	RMSD		Eqn (4) LEC
Model chemistry		QM	QM + LEC	*a*, *b*
M06-2X/TZP/SMD	Eqn (2)	3.88	1.20	0.50, 4.22
M06-2X/DZ/SMD	Eqn (2)	3.75	1.33	0.52, 4.19
M06-2X/TZ/PCM	Eqn (2)	11.54	1.20	0.28, 7.08
B3LYP/TZP/SMD	Eqn (2)	3.84	1.30	0.49, 3.18
B3LYP/TZP/PCM	Eqn (2)	10.36	1.18	0.28, 6.45
M06-2X/TZP/SMD	Eqn (5)	13.7	1.21	0.28, 5.17
M06-2X/TZP/SMD	pKYaY	9.45	1.21	0.50, −3.20

To confirm that the density functional and basis set have a limited effect on the accuracy, ESI Table SI2† reports a small benchmark study of rigid molecules structurally similar to tetra-aza macrocycles. For these molecules, the effect of conformational flexibility and prototropic tautomerism are minimized. RMSD obtained with the accurate *ab initio* composite approach CBS-QB3 in model solvent are comparable to that obtained with DFT. This strongly suggests that errors in the QM+LEC results in Table 1 arise mostly from the model solvent.

### 3.3 Explicit solvent

Additional insight comes from considering the role of explicit solvent. Hybrid explicit+continuum solvation models can significantly improve p*K*_a_ prediction, especially for sets of related molecules where the position of explicit solvent is well-defined (*e.g.*, two explicit water molecules hydrogen-bonded to a monocarboxylic acid).^60,61^ However, for flexible polybasic macrocycles, the optimum position and orientation of explicit solvent is difficult to determine *a priori*.

**Fig. 4:**
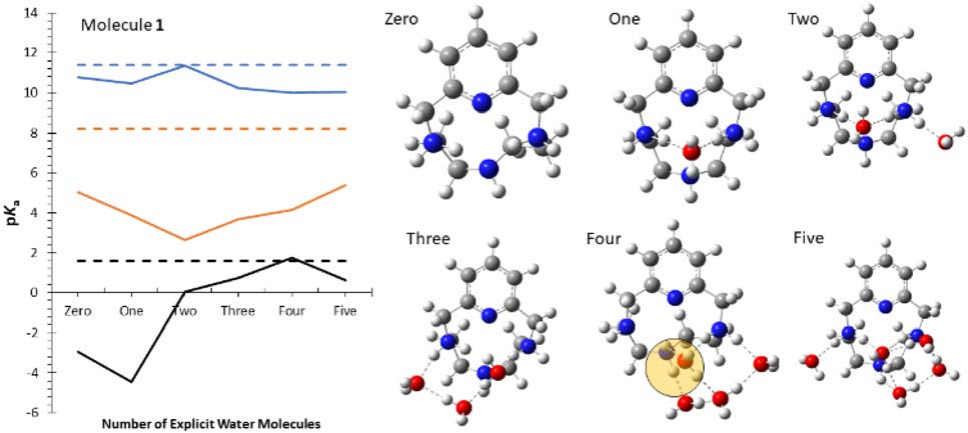
Explicit solvent calculations for molecule 1. (left) First (blue), second (red), and third (black) p*K*_a_. Horizontal dashed lines are experimental values, other lines are calculations with 0–5 explicit water molecules. (right) Computed structures for charge +2 state with 0–5 explicit water molecules.

Fig. 4 presents an initial study for molecule 1, showing p*K*_a_ computed (QM) with increasing numbers of explicit solvent molecules. Geometries are obtained using the quantum cluster growth (QCG) algorithm.^52^ ESI Fig. SI3† shows results for the hydroxypyridines used in the *ab initio* benchmark. Explicit solvent improves the computed p*K*_a_ values, consistent with previous studies.^62^ The predicted low-energy solvent configurations appear chemically reasonable. However, the improvement is not monotonic with increasing number of solvent molecules. This is not a special limitation of the QCG conformation search, it is an intrinsic limitation of any hybrid explicit+continuum solvent model.

### 3.4. Predicted p*K*_a_

We conclude by reported the predicted p*K*_a_ values for novel tetra-aza macrocycles 11–14. Table 2 reports the QM+LEC results. These are computed with our preferred method: CREST/xTB structure optimization, M06-2X/def2-TZVP structure refinement with the SMD continuum solvent model, p*K*_a_ computed from the experimental proton solvation free energy, and LEC *a* = 0.5 and *b* = 4.22. The computed values are chemically reasonable. Electron-withdrawing groups R = Cl and R = CF_3_ reduce the p*K*_a_ values relative to molecule 9 (R = H). Electron-donating groups R = NMe_2_ and R = OMe increase the p*K*_a_ values relative to molecule 9. Molecule 12 (R = NMe_2_) is predicted to have both p*K*_a_ well above 7, suggesting that it will be doubly protonated at physiological pH. Molecules 11 and 14 (R = Cl and R = CF_3_) are predicted to have both p*K*_a_ around or less than 7, suggesting that both molecules may have an appreciable concentration of charge-neutral tautomer at physiological pH.

## 4. Conclusions

Accurate prediction of the p*K*_a_ values, protonation sites, and pH-dependent speciation of polybasic drug candidates remains a significant challenge in computational medicinal chemistry. Here we used black-box methods to predict the p*K*_a_ and speciation of four previously un-tested tetra-aza macrocycle small molecules. These flexible molecules possess four to six acid/base sites and pose a significant challenge for p*K*_a_ prediction. This work included 32 p*K*_a_ values measured for 10 different aza-macrocyclic molecules as a baseline. The computational workflow employed combines exhaustive search over protonation tautomers, continuum models for water, CREST metadynamics and molecular dynamics for conformational sampling, modern density functional theory (DFT), SMD continuum solvent, and a linear empirical correction. Baseline studies give an RMSD within 1.2 log units of experimentally measured values and accurate predictions of the most stable protomer at each charge state. The predicted p*K*_a_ values for the previously un-tested macrocycles are chemcially reasonable. Our results highlight a significant step toward predicting the p*K*_a_ of large flexible molecules.

## Data availability

The data supporting this article have been included as part of the ESI.† Scripts for executing the computational workflow are available at https://github.com/bjanesko/DFTPropertyPredictionWorkflows.

## Author contributions

Investigation (T. K. H, K. P., M. M., D. M. F.), formal analysis (T. K. H, D. M. F., B. G. J, K. N. G.), writing (B. G. J., K. N. G.).

## Conflicts of interest

There are no conflicts to declare.

## Supplementary Material

RA-015-D5RA01015B-s001
