# Supplementary material for: Predicting pKa of flexible polybasic tetra-aza macrocycles
Source: RSC Adv. 2025 Apr 7;15(14):10663–70. doi: 10.1039/d5ra01015b (PMC11973477; doi:10.1039/d5ra01015b)
Supplement: RA-015-D5RA01015B-s001 [file RA-015-D5RA01015B-s001.pdf]

Supplementary Information (SI) for RSC Advances.
This journal is © The Royal Society of Chemistry 2025

Supplementary Information

Predicting pK_a of Flexible Polybasic Tetra-Aza Macrocycles

Tatum K. Harvey,[a] Kristof Pota,[b] Magy M. Mekhail,[c] David M. Freire,[a] Donatus A. Agbaglo,[a] Benjamin G. Janesko,[a,*] and Kayla N. Green[a,*]

Supporting Figures & Tables

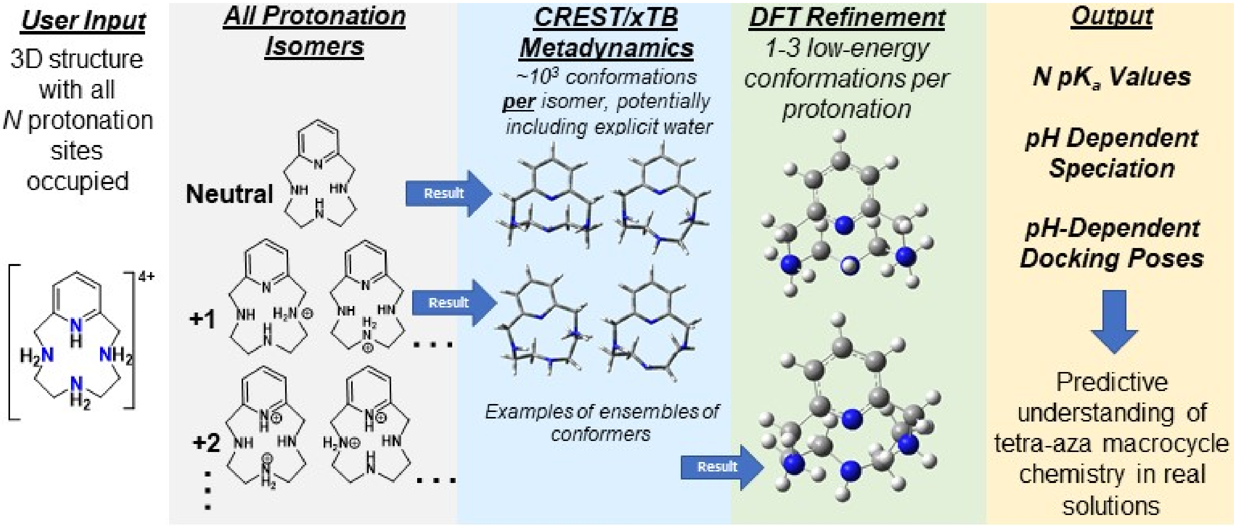

Figure SI1: Diagram of computational workflow employed, illustrated for Molecule 1.

Protons	Experiment	4-pyridone	4-hydroxy-pyridine	N2 protonated
a	~0	0.1	0.7	0.0
b	~0.2	0.3	0.2	0.1
c	~0.4	0.0	0.0	0.3
d	~1	0.5	-0.2	1.4

Table SI1: Predicted and experimental change of NMR chemical shift upon protonation of charge -1 molecule **2**. Experimental results and proton labels from ref 27, predictions are M06-2X/def2TZVP/SMD. Predictions are reported for the most stable computed neutral 4-pyridone structure, as well as the neutral 4-hydroxy-pyridine structure and the N2-protonated zwitterion assigned experimentally. Computed structures are shown in Figure SI2.

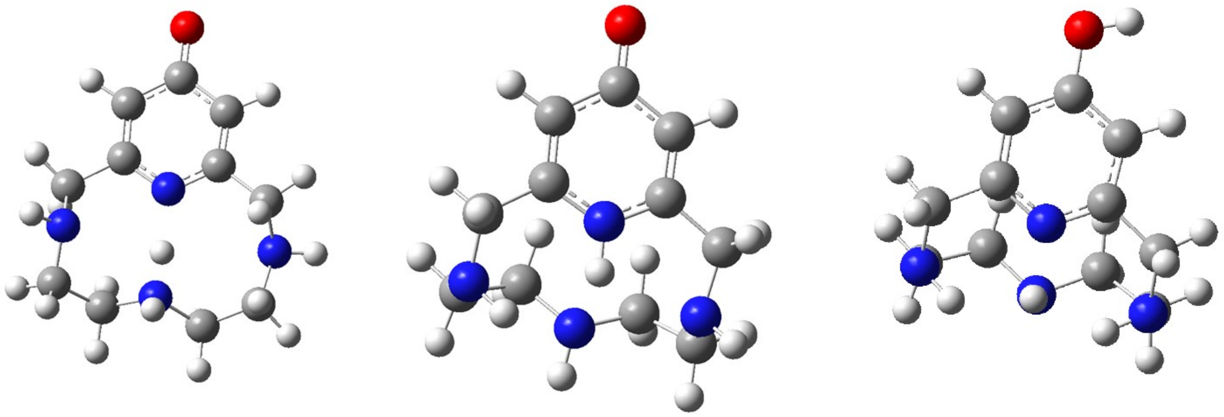

Figure SI2: Predicted structures of charge-neutral molecule **2**. (Left) N2-protonated zwitterion tautomer assigned from experimental NMR. (Center) Neutral 4-pyridone tautomer predicted to be most stable. (Right) Neutral 4-hydroxypyridine structure. The predicted structures suggest that the zwitterion may experience a degree of proton sharing between N2 and N4.

	**Exp**	**M06-2X SMD**	**M06-2X PCM**	**CBS-QB3 SMD**
DMA	10.73	13.56	8.77	14.90
TMA	9.8	13.18	9.15	14.31
Pyridine	5.17	7.37	3.18	8.41
4HP, pK_1	11.09	17.32	18.10	18.12
4HP, pK_2	3.27	7.10	3.62	7.87
3HP, pK_1	8.72	17.26	18.83	17.92
3HP, pK_2	4.86	6.57	2.36	7.70
Phenol	9.94	19.22	21.68	20.18
RMSD, QM		4.75	4.54	5.73
RMSD, QM+LEC		1.19	1.66	1.13

Table SI2: *Ab initio* and DFT computed pK_a and RMSD for a benchmark set of small molecules.

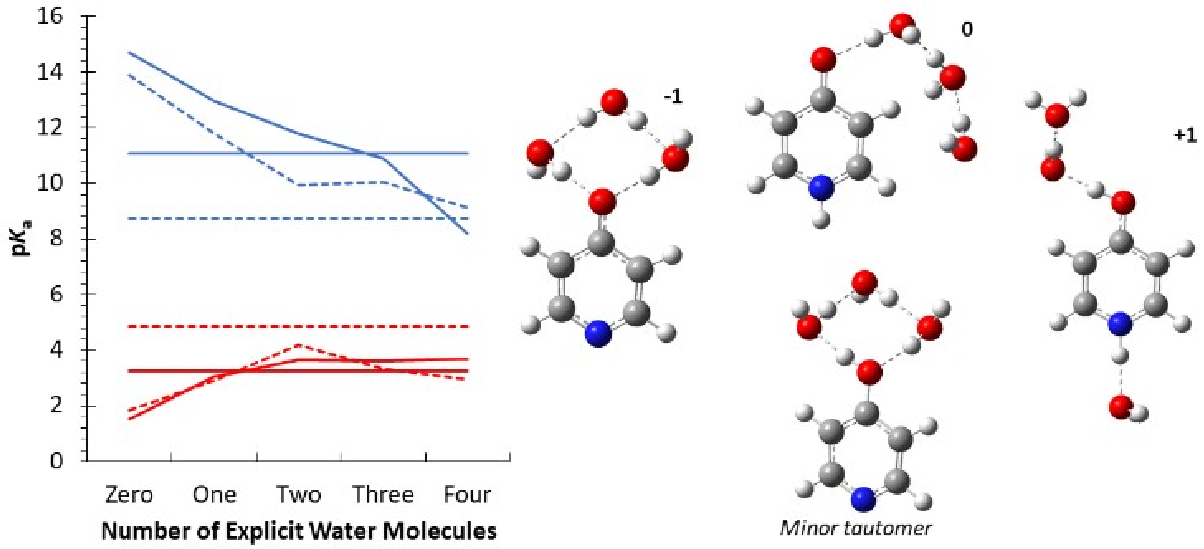

Figure SI3: Explicit solvent for hydroxypyridines. (Left) First (blue) and second (red) pKa of 4-hydroxy pyridine (solid) and 3-hydroxy pyridine (dashed). Horizontal lines are experimental values. (Right) Lowest-energy structures for 4-hydroxy pyridine with three explicit waters.

The remainder of this document includes the computed geometries and free energies of the most stable structure of each protomer, for molecules 1-14, as evaluated with our preferred computational protocol: CREST/xTB conformational analysis and M062X/def2TZVP/SMD refinement.

Most stable energy, Gibbs free energy (Ha), and geometry for protomer ./H_PyN3//0_1
E: -648.980278
G: -648.723765
Geometry:

Input orientation:

Center Number	Atomic Number	Atomic Type	Coordinates (Angstroms) X	Y	Z
1	6	0	1.150071	1.171302	-0.342261
2	6	0	1.191750	2.218475	0.571936
3	6	0	-1.194440	2.217292	0.571508
4	6	0	-1.151389	1.170144	-0.342634
5	7	0	-0.000333	0.690310	-0.816964
6	1	0	2.145235	2.583452	0.931232
7	1	0	-2.148413	2.581324	0.930470
8	6	0	-2.430596	0.538085	-0.853664
9	1	0	-3.245706	0.809939	-0.181113
10	1	0	-2.655291	0.994524	-1.821808
11	6	0	2.430139	0.540704	-0.852962
12	1	0	3.244648	0.813025	-0.179871
13	1	0	2.654870	0.997882	-1.820750
14	7	0	-2.438930	-0.907701	-1.024980
15	1	0	-1.657147	-1.177750	-1.611838
16	7	0	2.439999	-0.904980	-1.024999

17	1	0	1.658544	-1.175568	-1.612046
18	6	0	-2.438263	-1.690271	0.212486
19	1	0	-2.587531	-2.735926	-0.064362
20	1	0	-3.309935	-1.385672	0.795736
21	6	0	-1.209040	-1.596323	1.108138
22	1	0	-1.106579	-0.571824	1.493586
23	1	0	-1.369858	-2.239385	1.977637
24	6	0	2.440086	-1.688138	0.212095
25	1	0	2.590067	-2.733554	-0.065269
26	1	0	3.311628	-1.383183	0.795353
27	6	0	1.210952	-1.595460	1.107996
28	1	0	1.107717	-0.571128	1.493688
29	1	0	1.372481	-2.238570	1.977328
30	7	0	0.001065	-2.020868	0.411337
31	1	0	0.000827	-1.519512	-0.472828
32	6	0	-0.001689	2.760024	1.021180
33	1	0	-0.002218	3.575122	1.734216

Most stable energy, Gibbs free energy (Ha), and geometry for protomer ./H_PyN3//1_3

E: -649.443220

G: -649.173347

Geometry:

Input orientation:

Center Number	Atomic Number	Atomic Type	Coordinates (Angstroms) X	Y	Z
1	6	0	1.279188	-1.208180	-0.359008
2	6	0	2.338878	-1.366384	0.517432
3	6	0	2.452557	1.019610	0.644861
4	6	0	1.380137	1.083104	-0.240552
5	7	0	0.831693	-0.016960	-0.749542
6	1	0	2.661049	-2.355228	0.814786
7	1	0	2.876386	1.931996	1.043858
8	6	0	0.800696	2.406406	-0.689421
9	1	0	1.057146	3.171887	0.043400
10	1	0	1.312176	2.679279	-1.617288
11	6	0	0.507496	-2.381348	-0.903958
12	1	0	0.678492	-3.285990	-0.325664
13	1	0	0.755838	-2.564808	-1.948504
14	7	0	-0.634917	2.443732	-0.928914
15	1	0	-0.883500	1.637855	-1.495170
16	7	0	-0.941419	-2.044916	-0.842005
17	1	0	-1.482448	-2.695395	-1.414332
18	6	0	-1.462651	2.425790	0.278889
19	1	0	-2.437027	2.856032	0.028209
20	1	0	-1.006718	3.090995	1.014071
21	6	0	-1.718191	1.071994	0.928137
22	1	0	-0.778161	0.597584	1.212548
23	1	0	-2.287089	1.235490	1.853973
24	6	0	-1.496882	-2.005759	0.543021
25	1	0	-1.720566	-3.025457	0.845932
26	1	0	-0.715036	-1.611653	1.190180
27	6	0	-2.732324	-1.125717	0.566826
28	1	0	-3.088106	-1.084074	1.603395
29	1	0	-3.515762	-1.588251	-0.035069
30	7	0	-2.441941	0.190889	0.011040
31	1	0	-3.318391	0.639398	-0.233195
32	1	0	-1.062054	-1.094606	-1.226765

33	6	0	2.945009	-0.220343	1.014806
34	1	0	3.773247	-0.297310	1.707605

Most stable energy, Gibbs free energy (Ha), and geometry for protomer ./H_PyN3//1_2
E: -649.443220
G: -649.173345
Geometry:
Input orientation:

Center Number	Atomic Number	Atomic Type	Coordinates (Angstroms) X	Y	Z
1	6	0	1.379902	-1.083419	-0.240509
2	6	0	2.452355	-1.020194	0.644885
3	6	0	2.339231	1.365830	0.517506
4	6	0	1.279488	1.207890	-0.358917
5	7	0	0.831707	0.016782	-0.749465
6	1	0	2.875980	-1.932687	1.043853
7	1	0	2.661639	2.354593	0.814874
8	6	0	0.508068	2.381252	-0.903837
9	1	0	0.679248	3.285832	-0.325500
10	1	0	0.756484	2.564702	-1.948368
11	6	0	0.800161	-2.406583	-0.689405
12	1	0	1.056349	-3.172115	0.043456
13	1	0	1.311668	-2.679603	-1.617215
14	7	0	-0.940923	2.045141	-0.841944
15	1	0	-1.481789	2.695764	-1.414261
16	7	0	-0.635437	-2.443558	-0.929037
17	1	0	-0.883778	-1.637604	-1.495287
18	6	0	-1.496446	2.006050	0.543059
19	1	0	-1.719884	3.025787	0.846018
20	1	0	-0.714727	1.611710	1.190230
21	6	0	-2.732114	1.126322	0.566753
22	1	0	-3.087960	1.084706	1.603301
23	1	0	-3.515402	1.589092	-0.035155
24	6	0	-1.463287	-2.425479	0.278684
25	1	0	-2.437744	-2.855466	0.027883
26	1	0	-1.007591	-3.090840	1.013872
27	6	0	-1.718556	-1.071661	0.927991
28	1	0	-0.778435	-0.597487	1.212493
29	1	0	-2.287555	-1.235077	1.853779
30	7	0	-2.442034	-0.190324	0.010902
31	1	0	-3.318584	-0.638599	-0.233407
32	1	0	-1.061765	1.094874	-1.226745
33	6	0	2.945104	0.219636	1.014844
34	1	0	3.773376	0.296395	1.707626

Most stable energy, Gibbs free energy (Ha), and geometry for protomer ./H_PyN3//1_1
E: -649.447276
G: -649.177823
Geometry:
Input orientation:

Center Number	Atomic Number	Atomic Type	Coordinates (Angstroms) X	Y	Z
1	6	0	1.184284	-1.153696	0.183472
2	6	0	2.273930	-1.195353	-0.678255
3	6	0	2.273830	1.195470	-0.678476
4	6	0	1.184190	1.153881	0.183259

5	7	0	0.676573	0.000111	0.617330
6	1	0	2.660737	-2.147675	-1.017088
7	1	0	2.660559	2.147762	-1.017484
8	6	0	0.557365	2.445952	0.666403
9	1	0	1.152614	2.799056	1.511511
10	1	0	0.668247	3.192728	-0.129990
11	6	0	0.557559	-2.445735	0.666836
12	1	0	1.152891	-2.798698	1.511943
13	1	0	0.668424	-3.192609	-0.129469
14	7	0	-0.824274	2.325597	1.113105
15	1	0	-1.000979	3.052093	1.794292
16	7	0	-0.824056	-2.325396	1.113616
17	1	0	-1.000656	-3.051766	1.794963
18	6	0	-1.815358	2.456664	0.049516
19	1	0	-1.634429	3.330304	-0.589016
20	1	0	-2.793211	2.582464	0.515879
21	6	0	-1.847619	1.244129	-0.858711
22	1	0	-2.670682	1.310004	-1.567485
23	1	0	-0.917932	1.136581	-1.417416
24	6	0	-1.815202	-2.456758	0.050122
25	1	0	-1.634278	-3.330546	-0.588210
26	1	0	-2.793023	-2.582481	0.516571
27	6	0	-1.847556	-1.244448	-0.858400
28	1	0	-2.670621	-1.310551	-1.567149
29	1	0	-0.917882	-1.136980	-1.417142
30	7	0	-2.013607	-0.000065	-0.063948
31	1	0	-2.923221	-0.000030	0.403824
32	1	0	-1.267536	0.000048	0.658798
33	6	0	2.831178	0.000042	-1.102663
34	1	0	3.678119	0.000015	-1.777301

Most stable energy, Gibbs free energy (Ha), and geometry for protomer ./H_PyN3//1_4
E: -649.438456
G: -649.170489
Geometry:

Input orientation:

Center Number	Atomic Number	Atomic Type	Coordinates (Angstroms) X	Y	Z
1	6	0	1.267551	-1.181814	0.034464
2	6	0	2.611883	-1.198498	-0.285315
3	6	0	2.612219	1.197717	-0.285365
4	6	0	1.267882	1.181427	0.034414
5	7	0	0.647228	-0.000104	0.191723
6	1	0	3.113500	-2.149613	-0.397968
7	1	0	3.114106	2.148685	-0.398055
8	6	0	0.493937	2.467433	0.176883
9	1	0	1.166603	3.180316	0.652925
10	1	0	0.315954	2.847111	-0.837751
11	6	0	0.493227	-2.467584	0.176994
12	1	0	1.165670	-3.180640	0.653092
13	1	0	0.315152	-2.847266	-0.837622
14	7	0	-0.739345	2.363046	0.938535
15	1	0	-0.762430	3.117064	1.611467
16	7	0	-0.740041	-2.362770	0.938609
17	1	0	-0.763388	-3.116754	1.611570
18	6	0	-1.955219	2.436549	0.126165
19	1	0	-1.973154	3.333472	-0.504986

20	1	0	-2.803625	2.495366	0.810084
21	6	0	-2.121009	1.224036	-0.769154
22	1	0	-3.062254	1.311907	-1.323378
23	1	0	-1.318788	1.190540	-1.514856
24	6	0	-1.955917	-2.435902	0.126208
25	1	0	-1.974111	-3.332828	-0.504930
26	1	0	-2.804358	-2.494451	0.810107
27	6	0	-2.121324	-1.223346	-0.769133
28	1	0	-3.062574	-1.310957	-1.323388
29	1	0	-1.319067	-1.190085	-1.514808
30	7	0	-2.043659	0.000341	0.015250
31	1	0	-2.754110	0.000442	0.741569
32	1	0	-0.422627	0.000042	0.376997
33	6	0	3.287288	-0.000489	-0.446464
34	1	0	4.341984	-0.000642	-0.688848

Most stable energy, Gibbs free energy (Ha), and geometry for protomer ./H_PyN3//2_1
E: -649.898870
G: -649.613649
Geometry:
Input orientation:

Center Number	Atomic Number	Atomic Type	Coordinates (Angstroms) X	Y	Z
1	6	0	1.258228	-1.225627	-0.329654
2	6	0	2.550545	-1.426754	0.137381
3	6	0	2.743917	0.928339	0.463075
4	6	0	1.442562	1.037531	-0.004074
5	7	0	0.718718	-0.004010	-0.403864
6	1	0	2.948611	-2.431368	0.192943
7	1	0	3.290077	1.812287	0.764916
8	6	0	0.807065	2.403214	-0.047461
9	1	0	0.599161	2.762264	0.960330
10	1	0	1.476958	3.111485	-0.529146
11	6	0	0.443879	-2.416259	-0.777708
12	1	0	0.755939	-3.278288	-0.176546
13	1	0	0.707004	-2.640963	-1.814011
14	7	0	-0.476371	2.406097	-0.808695
15	1	0	-0.501891	3.238783	-1.401160
16	7	0	-0.987714	-2.177950	-0.714419
17	1	0	-1.468155	-2.938436	-1.181388
18	6	0	-1.718904	2.419246	0.024543
19	1	0	-2.549923	2.623000	-0.649347
20	1	0	-1.617577	3.250985	0.717167
21	6	0	-1.958760	1.148636	0.803400
22	1	0	-1.070160	0.791476	1.320600
23	1	0	-2.734987	1.348251	1.538660
24	6	0	-1.493980	-2.057293	0.651539
25	1	0	-1.709978	-3.020940	1.119589
26	1	0	-0.741309	-1.560390	1.265880
27	6	0	-2.761192	-1.226620	0.631225
28	1	0	-3.131602	-0.998276	1.627798
29	1	0	-3.541074	-1.724565	0.058885
30	7	0	-2.461522	0.058135	-0.074546
31	1	0	-1.749019	-0.197312	-0.780631
32	1	0	-0.478573	1.594670	-1.437506
33	1	0	-3.292915	0.390915	-0.567070
34	6	0	3.306181	-0.334162	0.529198

35	1	0	4.318315	-0.466087	0.889119

Most stable energy, Gibbs free energy (Ha), and geometry for protomer ./H_PyN3//2_6
E: -649.890512
G: -649.606718
Geometry:
Input orientation:

Center Number	Atomic Number	Atomic Type	Coordinates (Angstroms) X	Y	Z
1	6	0	1.530674	-0.476716	0.440003
2	6	0	2.775123	-0.107063	-0.004890
3	6	0	1.888528	2.068223	-0.528230
4	6	0	0.646648	1.657528	-0.060552
5	7	0	0.524851	0.416749	0.407380
6	1	0	3.591205	-0.814799	0.035059
7	1	0	2.002016	3.076214	-0.902436
8	6	0	-0.570523	2.541095	-0.066824
9	1	0	-0.253147	3.520962	0.289663
10	1	0	-0.869254	2.673486	-1.114993
11	6	0	1.201662	-1.837410	0.975397
12	1	0	0.399220	-1.796604	1.708278
13	1	0	2.082561	-2.275318	1.436729
14	7	0	-1.652884	2.034104	0.758473
15	1	0	-2.026492	2.798952	1.303288
16	7	0	0.776543	-2.772418	-0.112042
17	1	0	0.589859	-3.680249	0.324937
18	6	0	-2.753939	1.409678	0.023149
19	1	0	-3.123787	2.054648	-0.782800
20	1	0	-3.571476	1.261208	0.730328
21	6	0	-2.385762	0.071486	-0.588865
22	1	0	-3.267418	-0.333011	-1.097449
23	1	0	-1.615773	0.206614	-1.355837
24	6	0	-0.423645	-2.384613	-0.926943
25	1	0	-0.158010	-1.489102	-1.486843
26	1	0	-0.551920	-3.197050	-1.637768
27	6	0	-1.687902	-2.217073	-0.098268
28	1	0	-1.666974	-2.916330	0.739681
29	1	0	-2.528115	-2.505435	-0.737547
30	7	0	-1.880369	-0.861178	0.424740
31	1	0	-2.549984	-0.901318	1.187095
32	1	0	-0.437786	0.050446	0.684372
33	1	0	1.565641	-2.910435	-0.751175
34	6	0	2.949714	1.186577	-0.497085
35	1	0	3.923313	1.499150	-0.850383

Most stable energy, Gibbs free energy (Ha), and geometry for protomer ./H_PyN3//2_2
E: -649.898871
G: -649.613627
Geometry:
Input orientation:

Center Number	Atomic Number	Atomic Type	Coordinates (Angstroms) X	Y	Z
1	6	0	1.444402	-1.035120	-0.004039
2	6	0	2.745265	-0.923470	0.463940
3	6	0	2.547791	1.431204	0.137572
4	6	0	1.256123	1.227647	-0.330134

5	7	0	0.718913	0.005003	-0.404435
6	1	0	3.292806	-1.806376	0.766338
7	1	0	2.944035	2.436536	0.193186
8	6	0	0.439732	2.416606	-0.778862
9	1	0	0.750287	3.279517	-0.178199
10	1	0	0.702429	2.641166	-1.815306
11	6	0	0.811411	-2.401966	-0.047700
12	1	0	0.604522	-2.761793	0.960022
13	1	0	1.482398	-3.108827	-0.529936
14	7	0	-0.991476	2.175845	-0.715405
15	1	0	-1.473178	2.935450	-1.182515
16	7	0	-0.472270	-2.406701	-0.808472
17	1	0	-0.496695	-3.239192	-1.401238
18	6	0	-1.497403	2.054916	0.650696
19	1	0	-1.714970	3.018432	1.118269
20	1	0	-0.743820	1.559590	1.265182
21	6	0	-2.763274	1.222178	0.630941
22	1	0	-3.133183	0.993692	1.627668
23	1	0	-3.544022	1.718661	0.058505
24	6	0	-1.714424	-2.421936	0.025255
25	1	0	-2.545355	-2.627435	-0.648221
26	1	0	-1.611337	-3.253294	0.718057
27	6	0	-1.956343	-1.151531	0.803827
28	1	0	-1.068181	-0.792410	1.320405
29	1	0	-2.731807	-1.352366	1.539549
30	7	0	-2.461734	-0.062378	-0.074319
31	1	0	-1.750228	0.194013	-0.781056
32	1	0	-0.475876	-1.594953	-1.436898
33	1	0	-3.292871	-0.397025	-0.566015
34	6	0	3.305182	0.340049	0.530089
35	1	0	4.316858	0.473906	0.890591

Most stable energy, Gibbs free energy (Ha), and geometry for protomer ./H_PyN3//2_5

E: -649.890513

G: -649.606718

Geometry:

Input orientation:

Center Number	Atomic Number	Atomic Type	Coordinates (Angstroms) X	Y	Z
1	6	0	-0.645595	1.657966	-0.060554
2	6	0	-1.887298	2.069641	-0.527842
3	6	0	-2.775416	-0.104956	-0.004260
4	6	0	-1.531116	-0.475597	0.440225
5	7	0	-0.524604	0.417083	0.407323
6	1	0	-2.000111	3.077727	-0.901993
7	1	0	-3.592032	-0.812061	0.035916
8	6	0	-1.203040	-1.836578	0.975483
9	1	0	-0.400543	-1.796413	1.708336
10	1	0	-2.084231	-2.273895	1.436817
11	6	0	0.572246	2.540603	-0.067225
12	1	0	0.255646	3.520819	0.288992
13	1	0	0.870957	2.672454	-1.115470
14	7	0	-0.778642	-2.771828	-0.112039
15	1	0	-0.592627	-3.679824	0.324881
16	7	0	1.654333	2.033044	0.758088
17	1	0	2.028487	2.797748	1.302732
18	6	0	0.421827	-2.384898	-0.926964

19	1	0	0.156835	-1.489222	-1.486896
20	1	0	0.549505	-3.197464	-1.637753
21	6	0	1.686245	-2.218206	-0.098346
22	1	0	1.664890	-2.917381	0.739661
23	1	0	2.526216	-2.507203	-0.737656
24	6	0	2.754914	1.407780	0.022770
25	1	0	3.125195	2.052427	-0.783240
26	1	0	3.572374	1.258760	0.729922
27	6	0	2.385718	0.069828	-0.589143
28	1	0	3.267031	-0.335348	-1.097774
29	1	0	1.615756	0.205485	-1.356044
30	7	0	1.879707	-0.862406	0.424548
31	1	0	2.549309	-0.902961	1.186893
32	1	0	0.437839	0.050033	0.684106
33	1	0	-1.567856	-2.909207	-0.751164
34	6	0	-2.949164	1.188827	-0.496371
35	1	0	-3.922633	1.502163	-0.849350

Most stable energy, Gibbs free energy (Ha), and geometry for protomer ./H_PyN3//2_4
E: -649.900434
G: -649.616883
Geometry:

Input orientation:

Center Number	Atomic Number	Atomic Type	Coordinates (Angstroms) X	Y	Z
1	6	0	1.390091	-1.212841	-0.294227
2	6	0	2.555019	-1.300464	0.434526
3	6	0	2.619613	1.111051	0.567234
4	6	0	1.453142	1.162722	-0.169552
5	7	0	0.924986	0.009626	-0.587780
6	1	0	2.950928	-2.272561	0.691091
7	1	0	3.065590	2.028670	0.923533
8	6	0	0.719559	2.421087	-0.547181
9	1	0	0.840845	3.137387	0.271022
10	1	0	1.226478	2.839615	-1.419083
11	6	0	0.560658	-2.371802	-0.775369
12	1	0	0.778946	-3.232791	-0.136200
13	1	0	0.876064	-2.623538	-1.789752
14	7	0	-0.677000	2.181164	-0.885088
15	1	0	-0.939942	2.808949	-1.631554
16	7	0	-0.849692	-2.004929	-0.798971
17	1	0	-1.346663	-2.633469	-1.419897
18	6	0	-1.620722	2.333505	0.225578
19	1	0	-2.558299	2.724023	-0.172311
20	1	0	-1.256305	3.048258	0.969348
21	6	0	-1.899573	1.027531	0.944915
22	1	0	-0.979076	0.538344	1.256971
23	1	0	-2.520324	1.190243	1.823326
24	6	0	-1.459179	-2.043746	0.530668
25	1	0	-1.641734	-3.065292	0.877612
26	1	0	-0.767951	-1.591461	1.244168
27	6	0	-2.784385	-1.311543	0.547771
28	1	0	-3.193624	-1.265732	1.554744
29	1	0	-3.503293	-1.797430	-0.110013
30	7	0	-2.648012	0.092455	0.052541
31	1	0	-2.192352	0.064537	-0.865785
32	1	0	0.052246	0.067847	-1.116775

33	1	0	-3.582058	0.480230	-0.100802
34	6	0	3.175618	-0.127542	0.851333
35	1	0	4.086238	-0.183574	1.433157

Most stable energy, Gibbs free energy (Ha), and geometry for protomer ./H_PyN3//3_1
E: -650.347071
G: -650.044982
Geometry:
Input orientation:

Center Number	Atomic Number	Atomic Type	Coordinates (Angstroms) X	Y	Z
1	6	0	-1.568440	-0.032105	0.495884
2	6	0	-2.689559	0.552057	-0.063841
3	6	0	-1.326332	2.423929	-0.652135
4	6	0	-0.253518	1.763177	-0.062648
5	7	0	-0.373982	0.572815	0.508167
6	1	0	-3.637907	0.031750	-0.045830
7	1	0	-1.181289	3.398356	-1.099647
8	6	0	1.105366	2.410657	-0.084316
9	1	0	1.485029	2.472647	-1.104299
10	1	0	1.042819	3.419557	0.317560
11	6	0	-1.622777	-1.415603	1.077158
12	1	0	-2.585076	-1.609313	1.545167
13	1	0	-0.838984	-1.576485	1.813001
14	7	0	2.107943	1.665936	0.731155
15	7	0	-1.472638	-2.457933	0.004809
16	6	0	2.970693	0.685758	-0.005132
17	1	0	3.708522	0.325731	0.710257
18	1	0	3.480338	1.250500	-0.782208
19	6	0	2.240240	-0.464463	-0.654637
20	1	0	1.416429	-0.134607	-1.283884
21	1	0	2.954772	-1.000471	-1.276328
22	6	0	-0.288924	-2.367781	-0.907636
23	1	0	-0.443428	-3.146698	-1.650147
24	1	0	-0.337098	-1.410583	-1.421534
25	6	0	1.042883	-2.637527	-0.238231
26	1	0	1.727485	-3.037284	-0.982981
27	1	0	0.940163	-3.367705	0.563532
28	7	0	1.723908	-1.439732	0.348321
29	1	0	-1.471588	-3.378723	0.455877
30	1	0	1.627683	1.208865	1.513840
31	1	0	1.085278	-0.947761	0.984514
32	6	0	-2.561993	1.807864	-0.645660
33	1	0	-3.419672	2.295110	-1.089916
34	1	0	-2.309421	-2.424552	-0.586781
35	1	0	2.512859	-1.776792	0.908979
36	1	0	2.743820	2.350118	1.148572

Most stable energy, Gibbs free energy (Ha), and geometry for protomer ./H_PyN3//3_2
E: -650.335019
G: -650.033478
Geometry:
Input orientation:

Center Number	Atomic Number	Atomic Type	Coordinates (Angstroms) X	Y	Z
1	6	0	0.829961	-1.602386	-0.357216

2	6	0	1.820758	-2.051267	0.496178
3	6	0	2.741720	0.171057	0.555087
4	6	0	1.751346	0.587382	-0.301961
5	7	0	0.863431	-0.321745	-0.748815
6	1	0	1.807724	-3.083253	0.816116
7	1	0	3.464291	0.888971	0.915513
8	6	0	1.610997	2.008812	-0.752091
9	1	0	2.570574	2.507232	-0.647908
10	1	0	1.279900	2.071189	-1.786821
11	6	0	-0.318238	-2.438389	-0.848261
12	1	0	-0.341716	-3.354333	-0.251076
13	1	0	-0.128649	-2.717460	-1.886447
14	7	0	0.631632	2.798498	0.072763
15	1	0	0.834516	2.666616	1.071113
16	7	0	-1.553147	-1.667621	-0.783836
17	1	0	-2.255974	-2.118266	-1.359611
18	6	0	-0.824550	2.558263	-0.203784
19	1	0	-0.895349	2.261495	-1.250884
20	1	0	-1.332272	3.510492	-0.079866
21	6	0	-1.422325	1.502446	0.719719
22	1	0	-0.696758	0.740536	0.993105
23	1	0	-1.810451	1.941924	1.634727
24	6	0	-2.049460	-1.527546	0.586164
25	1	0	-2.520545	-2.440464	0.961273
26	1	0	-1.202083	-1.321144	1.242606
27	6	0	-3.068744	-0.409149	0.682516
28	1	0	-3.302187	-0.169767	1.717398
29	1	0	-3.984960	-0.666142	0.154091
30	7	0	-2.553925	0.832222	0.022895
31	1	0	-2.243374	0.554893	-0.916732
32	1	0	0.111282	-0.018054	-1.373042
33	1	0	0.823658	3.785155	-0.125218
34	1	0	-3.321851	1.498859	-0.091564
35	6	0	2.784769	-1.165226	0.942100
36	1	0	3.561467	-1.503956	1.614491

Most stable energy, Gibbs free energy (Ha), and geometry for protomer ./H_PyN3//3_3
E: -650.335003
G: -650.033619
Geometry:
Input orientation:

Center Number	Atomic Number	Atomic Type	Coordinates (Angstroms) X	Y	Z
1	6	0	1.746994	-0.597847	-0.302700
2	6	0	2.739210	-0.187762	0.555280
3	6	0	1.831815	2.040034	0.496718
4	6	0	0.838912	1.597442	-0.357480
5	7	0	0.864722	0.316799	-0.749688
6	1	0	3.457253	-0.910126	0.915889
7	1	0	1.824739	3.071937	0.817088
8	6	0	-0.304072	2.440342	-0.848713
9	1	0	-0.322427	3.356374	-0.251494
10	1	0	-0.112647	2.718259	-1.886869
11	6	0	1.598948	-2.018390	-0.753431
12	1	0	2.556049	-2.521763	-0.650221
13	1	0	1.266641	-2.078704	-1.787849
14	7	0	-1.543326	1.676545	-0.784431

15	1	0	-2.243467	2.131075	-1.360430
16	7	0	0.616171	-2.802803	0.072136
17	1	0	0.820758	-2.672827	1.070409
18	6	0	-2.040677	1.539772	0.585590
19	1	0	-2.506865	2.455444	0.960084
20	1	0	-1.194533	1.329194	1.242252
21	6	0	-3.065992	0.427020	0.682351
22	1	0	-3.301177	0.189608	1.717284
23	1	0	-3.980558	0.688515	0.153262
24	6	0	-0.838503	-2.552857	-0.203062
25	1	0	-0.908093	-2.255547	-1.250151
26	1	0	-1.352518	-3.501691	-0.079062
27	6	0	-1.429469	-1.493436	0.720621
28	1	0	-0.699356	-0.735732	0.993608
29	1	0	-1.819786	-1.930432	1.635877
30	7	0	-2.557507	-0.817524	0.023726
31	1	0	-2.245405	-0.542286	-0.915919
32	1	0	0.110167	0.017846	-1.373548
33	1	0	0.801633	-3.790509	-0.126894
34	1	0	-3.328983	-1.480090	-0.090520
35	6	0	2.790058	1.148002	0.943044
36	1	0	3.568203	1.481626	1.616307

Most stable energy, Gibbs free energy (Ha), and geometry for protomer ./OH_PyN3//0_1
E: -723.754024
G: -723.507241
Geometry:
Input orientation:

Center Number	Atomic Number	Atomic Type	Coordinates (Angstroms) X	Y	Z
1	6	0	-2.001680	-1.207131	0.064454
2	1	0	-2.412621	-2.166201	0.360224
3	6	0	-2.692230	-0.016261	0.423386
4	6	0	-2.016553	1.182499	0.062249
5	1	0	-2.439579	2.136836	0.356282
6	7	0	-0.238245	-0.002245	-1.025061
7	7	0	1.367593	-2.427629	-0.911832
8	1	0	1.724710	-1.627964	-1.424018
9	7	0	2.189075	0.013623	0.700853
10	1	0	1.821722	0.010870	-0.247390
11	7	0	1.337431	2.443629	-0.911991
12	1	0	1.706121	1.648481	-1.423002
13	6	0	-0.088159	-2.428429	-1.010074
14	1	0	-0.472411	-3.247279	-0.399446
15	1	0	-0.349968	-2.658782	-2.046837
16	6	0	-0.811285	-1.149173	-0.630546
17	6	0	1.907941	-2.427811	0.449211
18	1	0	2.987064	-2.577826	0.370981
19	1	0	1.500909	-3.299763	0.965956
20	6	0	-0.825566	1.138203	-0.632571
21	6	0	1.651607	-1.198753	1.313356
22	1	0	0.574586	-1.099071	1.505512
23	1	0	2.129601	-1.357439	2.284009
24	6	0	-0.117996	2.425533	-1.013870
25	1	0	-0.379941	2.649695	-2.051940
26	1	0	-0.514206	3.241052	-0.406441
27	6	0	1.874441	2.451108	0.450352

28	1	0	1.453511	3.316823	0.966467
29	1	0	2.951451	2.616833	0.374790
30	6	0	1.634149	1.218030	1.313448
31	1	0	2.108916	1.382769	2.284682
32	1	0	0.558449	1.103136	1.504491
33	8	0	-3.814370	-0.022542	1.048019

Most stable energy, Gibbs free energy (Ha), and geometry for protomer ./OH_PyN3//1_3
E: -724.221588
G: -723.960358
Geometry:
Input orientation:

Center Number	Atomic Number	Atomic Type	Coordinates (Angstroms) X	Y	Z
1	6	0	-2.006945	-1.239313	0.308748
2	1	0	-2.337494	-2.212570	0.651438
3	6	0	-2.627564	-0.061099	0.822995
4	6	0	-2.040290	1.157789	0.372096
5	1	0	-2.412555	2.096725	0.765992
6	7	0	-0.473142	0.027591	-1.038340
7	7	0	1.238762	-2.066549	-0.862351
8	1	0	1.811317	-2.720358	-1.398113
9	7	0	2.688941	0.115264	0.218709
10	1	0	3.593192	0.567677	0.134570
11	7	0	1.132874	2.435824	-0.914871
12	1	0	1.456844	1.624044	-1.433114
13	6	0	-0.214096	-2.340662	-1.070178
14	1	0	-0.475532	-3.243075	-0.523065
15	1	0	-0.361189	-2.500888	-2.137206
16	6	0	-0.967318	-1.128868	-0.583253
17	6	0	1.654463	-2.082293	0.570510
18	1	0	1.835400	-3.114691	0.858790
19	1	0	0.815523	-1.700197	1.149175
20	6	0	-0.992860	1.151868	-0.522582
21	6	0	2.891441	-1.223956	0.759471
22	1	0	3.125001	-1.221483	1.831466
23	1	0	3.733208	-1.680965	0.237424
24	6	0	-0.327761	2.434907	-0.968232
25	1	0	-0.623101	2.628154	-2.002751
26	1	0	-0.701007	3.264638	-0.367112
27	6	0	1.692684	2.351332	0.436832
28	1	0	1.084538	2.969895	1.099778
29	1	0	2.692238	2.796852	0.420874
30	6	0	1.827307	0.960593	1.046334
31	1	0	2.233530	1.069491	2.061905
32	1	0	0.850414	0.485922	1.141853
33	8	0	-3.595904	-0.098779	1.655654
34	1	0	1.417451	-1.110069	-1.208264

Most stable energy, Gibbs free energy (Ha), and geometry for protomer ./OH_PyN3//1_2
E: -724.221587
G: -723.960360
Geometry:
Input orientation:

Center Number	Atomic Number	Atomic Type	Coordinates (Angstroms) X	Y	Z

1	6	0	-2.038786	-1.161159	0.371554
2	1	0	-2.409839	-2.100537	0.765540
3	6	0	-2.627929	0.056994	0.822022
4	6	0	-2.008843	1.235976	0.307707
5	1	0	-2.340849	2.208835	0.650116
6	7	0	-0.472873	-0.029018	-1.038697
7	7	0	1.136579	-2.434941	-0.913805
8	1	0	1.459737	-1.622863	-1.432087
9	7	0	2.688852	-0.111987	0.219572
10	1	0	3.593746	-0.563223	0.136020
11	7	0	1.235996	2.067512	-0.862721
12	1	0	1.807804	2.721897	-1.398571
13	6	0	-0.324016	-2.436083	-0.968035
14	1	0	-0.696451	-3.266293	-0.367077
15	1	0	-0.618462	-2.629798	-2.002718
16	6	0	-0.991105	-1.153935	-0.522814
17	6	0	1.695464	-2.349271	0.438196
18	1	0	2.695581	-2.793554	0.423018
19	1	0	1.087680	-2.968318	1.101024
20	6	0	-0.968822	1.126823	-0.584005
21	6	0	1.828003	-0.958117	1.047206
22	1	0	0.850450	-0.484693	1.142103
23	1	0	2.233941	-1.066057	2.062990
24	6	0	-0.217180	2.339592	-1.070970
25	1	0	-0.364229	2.499415	-2.138065
26	1	0	-0.480007	3.241741	-0.524089
27	6	0	1.651298	2.084331	0.570253
28	1	0	0.812679	1.701395	1.148827
29	1	0	1.830818	3.117077	0.858172
30	6	0	2.889364	1.227723	0.759872
31	1	0	3.730649	1.685688	0.237888
32	1	0	3.122624	1.225983	1.831934
33	8	0	-3.596533	0.093473	1.654433
34	1	0	1.416022	1.111148	-1.208249

Most stable energy, Gibbs free energy (Ha), and geometry for protomer ./OH_PyN3//1_1
E: -724.228406
G: -723.968634
Geometry:
Input orientation:

Center Number	Atomic Number	Atomic Type	Coordinates (Angstroms) X	Y	Z
1	6	0	-2.142444	-1.149528	0.080206
2	1	0	-2.629795	-2.102649	-0.080072
3	6	0	-2.830971	0.056145	-0.291642
4	6	0	-2.098077	1.270467	-0.079433
5	1	0	-2.542999	2.215381	-0.364146
6	7	0	-0.281686	0.076690	0.818825
7	7	0	1.268638	-2.454577	0.921684
8	1	0	1.724880	-1.691929	1.409597
9	7	0	2.243255	-0.128250	-0.634370
10	1	0	3.252103	-0.241978	-0.638775
11	7	0	1.370424	2.254812	0.928398
12	1	0	1.722178	2.947715	1.575372
13	6	0	-0.172328	-2.370940	1.069649
14	1	0	-0.409971	-2.491188	2.129834
15	1	0	-0.627188	-3.212654	0.546962

16	6	0	-0.891463	-1.117694	0.613757
17	6	0	1.770497	-2.528294	-0.448045
18	1	0	1.264497	-3.354742	-0.951491
19	1	0	2.830211	-2.788775	-0.392828
20	6	0	-0.848464	1.250694	0.459293
21	6	0	1.628026	-1.270804	-1.296856
22	1	0	2.055347	-1.470002	-2.289280
23	1	0	0.572006	-1.041008	-1.457654
24	6	0	-0.046610	2.504334	0.719401
25	1	0	-0.237058	3.200709	-0.105704
26	1	0	-0.454345	2.961421	1.622765
27	6	0	2.169911	2.316219	-0.297780
28	1	0	3.222607	2.309233	-0.009003
29	1	0	1.983422	3.248174	-0.847474
30	6	0	1.900737	1.153956	-1.237222
31	1	0	0.838611	1.133084	-1.497960
32	1	0	2.438881	1.335613	-2.176045
33	8	0	-3.989908	0.043383	-0.786793
34	1	0	0.708601	0.114994	1.061334

Most stable energy, Gibbs free energy (Ha), and geometry for protomer ./OH_PyN3//1_4

E: -724.219342

G: -723.960945

Geometry:

Input orientation:

Center Number	Atomic Number	Atomic Type	Coordinates (Angstroms) X	Y	Z
1	6	0	-2.443443	-1.017599	-0.355244
2	1	0	-2.986146	-1.920241	-0.611255
3	6	0	-3.157237	0.208756	-0.227527
4	6	0	-2.354832	1.327947	0.150365
5	1	0	-2.828671	2.292485	0.289301
6	7	0	-0.364456	0.015190	0.190176
7	7	0	0.783628	-2.524008	0.628117
8	1	0	0.640507	-3.366410	1.168165
9	7	0	2.296788	-0.135449	0.172357
10	1	0	1.207263	-0.055278	0.303317
11	7	0	0.909435	2.720570	-0.157858
12	1	0	0.876754	3.723403	-0.272623
13	6	0	-0.328876	-2.369689	-0.313098
14	1	0	-1.036812	-3.189485	-0.192485
15	1	0	0.031014	-2.427690	-1.346330
16	6	0	-1.083579	-1.066488	-0.147346
17	6	0	2.107029	-2.603194	0.017280
18	1	0	2.188615	-3.408984	-0.723358
19	1	0	2.826070	-2.812334	0.810843
20	6	0	-1.002558	1.188086	0.345991
21	6	0	2.502374	-1.322723	-0.688045
22	1	0	3.547997	-1.367576	-0.991380
23	1	0	1.897111	-1.154323	-1.579949
24	6	0	-0.150998	2.353347	0.789261
25	1	0	-0.803572	3.213263	0.932625
26	1	0	0.279310	2.107214	1.767492
27	6	0	2.280589	2.367540	0.214930
28	1	0	2.396927	2.283817	1.302837
29	1	0	2.932754	3.177410	-0.111305
30	6	0	2.813780	1.110002	-0.446374

31	1	0	2.525602	1.089263	-1.496822
32	1	0	3.901315	1.080417	-0.384445
33	8	0	-4.416419	0.302143	-0.422549
34	1	0	2.734583	-0.277128	1.083550

Most stable energy, Gibbs free energy (Ha), and geometry for protomer ./OH_PyN3//1_5
E: -724.223923
G: -723.964507
Geometry:
Input orientation:

Center Number	Atomic Number	Atomic Type	Coordinates (Angstroms) X	Y	Z
1	6	0	-1.976117	-1.215905	-0.068998
2	1	0	-2.404076	-2.164409	-0.366128
3	6	0	-2.602641	-0.023231	-0.405645
4	6	0	-1.997053	1.179725	-0.068247
5	1	0	-2.439950	2.123505	-0.362857
6	7	0	-0.225776	-0.002333	1.013624
7	7	0	1.393636	-2.425576	0.924857
8	1	0	1.757168	-1.638326	1.451069
9	7	0	2.215073	0.018718	-0.688541
10	1	0	1.873515	0.015008	0.268548
11	7	0	1.352170	2.447773	0.927679
12	1	0	1.727881	1.666034	1.453558
13	6	0	-0.059157	-2.430508	1.017707
14	1	0	-0.331062	-2.655807	2.052748
15	1	0	-0.441780	-3.248897	0.405948
16	6	0	-0.782357	-1.155313	0.632218
17	6	0	1.939973	-2.422392	-0.433750
18	1	0	1.539860	-3.296523	-0.951931
19	1	0	3.019218	-2.565895	-0.350006
20	6	0	-0.800996	1.139944	0.633298
21	6	0	1.679458	-1.194893	-1.298176
22	1	0	2.156907	-1.353056	-2.268994
23	1	0	0.602013	-1.098281	-1.493320
24	6	0	-0.100593	2.428133	1.017915
25	1	0	-0.495938	3.238810	0.403975
26	1	0	-0.378666	2.650423	2.051962
27	6	0	1.900513	2.454698	-0.430155
28	1	0	2.977276	2.614713	-0.344602
29	1	0	1.487719	3.323184	-0.947886
30	6	0	1.660177	1.224427	-1.296407
31	1	0	0.584447	1.110770	-1.492252
32	1	0	2.135289	1.391756	-2.266840
33	8	0	-3.774254	-0.083793	-1.085709
34	1	0	-4.098648	0.809993	-1.263525

Most stable energy, Gibbs free energy (Ha), and geometry for protomer ./OH_PyN3//2_7
E: -724.684228
G: -724.413186
Geometry:
Input orientation:

Center Number	Atomic Number	Atomic Type	Coordinates (Angstroms) X	Y	Z
1	6	0	-2.275736	-1.206122	0.149585
2	1	0	-2.795817	-2.148512	0.251433

3	6	0	-2.960071	-0.001428	0.288922
4	6	0	-2.276700	1.202674	0.144512
5	1	0	-2.794298	2.148292	0.240986
6	7	0	-0.297424	-0.001028	-0.264893
7	7	0	1.114382	-2.360667	-0.942145
8	1	0	1.165371	-3.111917	-1.616530
9	7	0	2.410292	0.001121	0.031028
10	1	0	3.172758	0.001588	-0.640162
11	7	0	1.113218	2.359265	-0.948012
12	1	0	1.164428	3.109398	-1.623631
13	6	0	-0.148187	-2.468402	-0.230781
14	1	0	-0.008087	-2.841752	0.791688
15	1	0	-0.798737	-3.185714	-0.730029
16	6	0	-0.929966	-1.182245	-0.123128
17	6	0	2.296861	-2.435125	-0.081533
18	1	0	3.171883	-2.497633	-0.730753
19	1	0	2.286940	-3.331327	0.550906
20	6	0	-0.929628	1.179080	-0.127839
21	6	0	2.430889	-1.221911	0.818703
22	1	0	1.590975	-1.178882	1.521217
23	1	0	3.339869	-1.322537	1.423059
24	6	0	-0.150788	2.466515	-0.239623
25	1	0	-0.802316	3.180017	-0.743099
26	1	0	-0.014187	2.844256	0.781783
27	6	0	2.293767	2.436995	-0.085067
28	1	0	2.280885	3.334052	0.546097
29	1	0	3.169965	2.500195	-0.732602
30	6	0	2.428045	1.225249	0.817066
31	1	0	3.335991	1.328016	1.422620
32	1	0	1.587149	1.182128	1.518430
33	8	0	-4.271356	-0.053095	0.551982
34	1	0	-4.639168	0.839337	0.633864
35	1	0	0.763186	-0.001023	-0.417968

Most stable energy, Gibbs free energy (Ha), and geometry for protomer ./OH_PyN3//2_1

E: -724.686837

G: -724.412567

Geometry:

Input orientation:

Center Number	Atomic Number	Atomic Type	Coordinates (Angstroms) X	Y	Z
1	6	0	-2.253407	-1.100055	0.281493
2	1	0	-2.736144	-1.967619	0.712062
3	6	0	-2.875897	0.186774	0.408118
4	6	0	-2.114827	1.293423	-0.123522
5	1	0	-2.490512	2.299827	0.002031
6	7	0	-0.441845	-0.167174	-0.894262
7	7	0	1.065795	-2.409009	-0.789130
8	1	0	1.373407	-3.188171	-1.355179
9	7	0	2.639934	-0.019997	0.319854
10	1	0	3.575677	-0.369747	0.146022
11	7	0	1.302917	2.157833	-0.795274
12	1	0	1.808497	2.964450	-1.169399
13	6	0	-0.359991	-2.563455	-0.544360
14	1	0	-0.578240	-3.187431	0.329304
15	1	0	-0.809483	-3.050415	-1.411795
16	6	0	-1.062092	-1.242804	-0.357291

17	6	0	1.878748	-2.369723	0.430110
18	1	0	2.898152	-2.649159	0.156472
19	1	0	1.523257	-3.107395	1.159951
20	6	0	-0.930021	1.091951	-0.748617
21	6	0	1.923732	-1.015915	1.118127
22	1	0	0.913119	-0.653860	1.322761
23	1	0	2.405532	-1.153915	2.094299
24	6	0	-0.106646	2.223474	-1.284554
25	1	0	-0.064645	2.197962	-2.372844
26	1	0	-0.529400	3.169626	-0.959482
27	6	0	1.485162	2.096094	0.689316
28	1	0	0.602915	1.620611	1.114036
29	1	0	1.555581	3.112128	1.067228
30	6	0	2.736102	1.282715	0.972215
31	1	0	3.604436	1.808345	0.573250
32	1	0	2.857777	1.209649	2.058124
33	8	0	-3.983675	0.353451	0.970435
34	1	0	1.769991	1.318603	-1.169717
35	1	0	0.467166	-0.360081	-1.310022

Most stable energy, Gibbs free energy (Ha), and geometry for protomer ./OH_PyN3//2_3
E: -724.687434
G: -724.411685
Geometry:

Input orientation:

Center Number	Atomic Number	Atomic Type	Coordinates (Angstroms) X	Y	Z
1	6	0	-2.150238	-1.080967	0.296898
2	1	0	-2.575714	-2.028207	0.605113
3	6	0	-2.711445	0.135730	0.794040
4	6	0	-2.015631	1.315648	0.387622
5	1	0	-2.338829	2.279412	0.761852
6	7	0	-0.455488	0.075241	-0.947206
7	7	0	1.068821	-2.160873	-0.845328
8	1	0	1.569846	-2.877249	-1.373416
9	7	0	2.713085	-0.075902	0.207475
10	1	0	3.652529	0.286769	0.080425
11	7	0	1.276831	2.206896	-0.886628
12	1	0	1.726598	2.905590	-1.478578
13	6	0	-0.403811	-2.313777	-1.023041
14	1	0	-0.728245	-3.187632	-0.463636
15	1	0	-0.585584	-2.469522	-2.085570
16	6	0	-1.058945	-1.047688	-0.534800
17	6	0	1.505977	-2.183964	0.581430
18	1	0	1.604911	-3.222873	0.885530
19	1	0	0.712164	-1.724768	1.167823
20	6	0	-0.934867	1.223991	-0.453483
21	6	0	2.814484	-1.435228	0.733751
22	1	0	3.085310	-1.457126	1.795466
23	1	0	3.597051	-1.954517	0.179743
24	6	0	-0.195281	2.455119	-0.900904
25	1	0	-0.457291	2.698469	-1.930355
26	1	0	-0.400427	3.313560	-0.267155
27	6	0	1.930237	2.217771	0.464149
28	1	0	1.380925	2.915010	1.092552
29	1	0	2.939891	2.596680	0.313488
30	6	0	1.984107	0.837184	1.083302

31	1	0	2.460301	0.942524	2.065109
32	1	0	0.976999	0.454548	1.251608
33	8	0	-3.720802	0.163719	1.568039
34	1	0	1.327943	-1.237485	-1.223042
35	1	0	1.442142	1.283064	-1.310373

Most stable energy, Gibbs free energy (Ha), and geometry for protomer ./OH_PyN3//2_6
E: -724.682071
G: -724.405492
Geometry:
Input orientation:

Center Number	Atomic Number	Atomic Type	Coordinates (Angstroms) X	Y	Z
1	6	0	-2.012627	-1.248414	0.311150
2	1	0	-2.411817	-2.171072	0.715648
3	6	0	-2.723431	-0.028406	0.500947
4	6	0	-2.065814	1.123433	-0.028925
5	1	0	-2.518154	2.097158	0.117377
6	7	0	-0.243060	-0.161337	-0.900750
7	7	0	1.335777	-2.334177	-0.914709
8	1	0	1.674788	-3.114910	-1.480137
9	7	0	2.184961	0.260429	0.577436
10	1	0	3.194697	0.411784	0.502522
11	7	0	1.240022	2.299589	-0.998800
12	1	0	1.648567	2.986766	-1.618280
13	6	0	-0.102970	-2.558719	-0.593382
14	1	0	-0.158727	-3.200357	0.283693
15	1	0	-0.533167	-3.088198	-1.442261
16	6	0	-0.823304	-1.247433	-0.376443
17	6	0	2.254751	-2.205473	0.260743
18	1	0	3.263122	-2.103596	-0.136846
19	1	0	2.184189	-3.139816	0.812676
20	6	0	-0.868557	1.016065	-0.694205
21	6	0	1.916076	-1.065501	1.191362
22	1	0	0.874015	-1.084567	1.503626
23	1	0	2.538920	-1.152043	2.078909
24	6	0	-0.199324	2.255909	-1.244183
25	1	0	-0.344108	2.268222	-2.326686
26	1	0	-0.709062	3.134745	-0.833645
27	6	0	1.619131	2.630013	0.372491
28	1	0	0.986803	3.409035	0.813546
29	1	0	2.644128	3.000344	0.354274
30	6	0	1.550435	1.409792	1.281252
31	1	0	2.074218	1.577300	2.219297
32	1	0	0.523654	1.116795	1.493468
33	8	0	-3.839953	0.035211	1.111887
34	1	0	1.791231	0.307407	-0.376824
35	1	0	1.379175	-1.492085	-1.503136

Most stable energy, Gibbs free energy (Ha), and geometry for protomer ./OH_PyN3//2_10
E: -724.691126
G: -724.418406
Geometry:
Input orientation:

Center Number	Atomic Number	Atomic Type	Coordinates (Angstroms) X	Y	Z

1	6	0	-1.961321	-1.197258	-0.415626
2	1	0	-2.372438	-2.145990	-0.738576
3	6	0	-2.535636	-0.000199	-0.824078
4	6	0	-1.953035	1.202204	-0.442603
5	1	0	-2.359772	2.143572	-0.788069
6	7	0	-0.303077	0.009217	0.798663
7	7	0	1.233554	-2.321499	1.149473
8	1	0	1.476158	-3.046010	1.812320
9	7	0	2.306658	-0.006335	-0.152259
10	1	0	1.629557	0.002448	0.635661
11	7	0	1.244389	2.334193	1.116919
12	1	0	1.485838	3.065482	1.772652
13	6	0	-0.188607	-2.433364	0.852763
14	1	0	-0.697019	-2.743661	1.768413
15	1	0	-0.393208	-3.204051	0.099179
16	6	0	-0.837088	-1.143551	0.392457
17	6	0	2.106236	-2.463682	-0.011883
18	1	0	1.850817	-3.335980	-0.626476
19	1	0	3.125906	-2.599157	0.350431
20	6	0	-0.830711	1.157547	0.367319
21	6	0	2.058650	-1.252424	-0.922233
22	1	0	2.810488	-1.326581	-1.705548
23	1	0	1.081631	-1.139577	-1.391972
24	6	0	-0.174186	2.451502	0.804814
25	1	0	-0.365464	3.207493	0.033133
26	1	0	-0.687684	2.786036	1.709000
27	6	0	2.129933	2.454435	-0.037051
28	1	0	3.147569	2.581878	0.333886
29	1	0	1.890715	3.323239	-0.663021
30	6	0	2.076830	1.234879	-0.935864
31	1	0	1.102236	1.127715	-1.411865
32	1	0	2.835138	1.293689	-1.714213
33	8	0	-3.634122	0.044868	-1.613587
34	1	0	-3.936172	-0.852341	-1.814067
35	1	0	3.254458	-0.011034	0.231970

Most stable energy, Gibbs free energy (Ha), and geometry for protomer ./OH_PyN3//2_2
E: -724.686836
G: -724.412577
Geometry:
Input orientation:

Center Number	Atomic Number	Atomic Type	Coordinates (Angstroms) X	Y	Z
1	6	0	-2.113387	-1.296248	-0.125461
2	1	0	-2.487770	-2.303184	-0.000270
3	6	0	-2.876158	-0.190714	0.406034
4	6	0	-2.255366	1.096987	0.279860
5	1	0	-2.739454	1.963832	0.710359
6	7	0	-0.442012	0.166720	-0.895216
7	7	0	1.305817	-2.156042	-0.795597
8	1	0	1.812666	-2.961847	-1.169746
9	7	0	2.639042	0.023226	0.321383
10	1	0	3.574341	0.374571	0.148395
11	7	0	1.062708	2.410542	-0.787994
12	1	0	1.369980	3.190299	-1.353402
13	6	0	-0.103395	-2.223412	-1.285664
14	1	0	-0.525064	-3.170172	-0.960970

15	1	0	-0.060868	-2.197656	-2.373927
16	6	0	-0.928539	-1.093076	-0.749947
17	6	0	1.487147	-2.094717	0.689137
18	1	0	1.558815	-3.110823	1.066617
19	1	0	0.604003	-1.620684	1.113592
20	6	0	-1.063956	1.241443	-0.358347
21	6	0	2.736731	-1.279632	0.973187
22	1	0	2.857606	-1.206903	2.059214
23	1	0	3.606097	-1.803795	0.574547
24	6	0	-0.363543	2.563047	-0.544855
25	1	0	-0.812726	3.049191	-1.412911
26	1	0	-0.583685	3.186866	0.328445
27	6	0	1.874280	2.371777	0.432165
28	1	0	1.516830	3.108490	1.162021
29	1	0	2.893582	2.652914	0.159895
30	6	0	1.920536	1.017641	1.119455
31	1	0	2.401111	1.155802	2.096211
32	1	0	0.910225	0.654023	1.322843
33	8	0	-3.983923	-0.358951	0.967922
34	1	0	1.771976	-1.316058	-1.169474
35	1	0	0.467114	0.360793	-1.310163

Most stable energy, Gibbs free energy (Ha), and geometry for protomer ./OH_PyN3//2_5
E: -724.682072
G: -724.405490
Geometry:

Input orientation:

Center Number	Atomic Number	Atomic Type	Coordinates (Angstroms) X	Y	Z
1	6	0	-2.066696	-1.121980	0.029171
2	1	0	-2.519686	-2.095423	-0.116997
3	6	0	-2.723634	0.030244	-0.500709
4	6	0	-2.012022	1.249808	-0.311097
5	1	0	-2.410668	2.172696	-0.715607
6	7	0	-0.243025	0.161686	0.900700
7	7	0	1.238498	-2.300153	0.998761
8	1	0	1.646689	-2.987556	1.618224
9	7	0	2.184805	-0.261715	-0.577580
10	1	0	1.791277	-0.308490	0.376769
11	7	0	1.337147	2.333469	0.914591
12	1	0	1.379929	1.491359	1.503038
13	6	0	-0.200790	-2.255552	1.244313
14	1	0	-0.345449	-2.267687	2.326835
15	1	0	-0.711126	-3.134102	0.833904
16	6	0	-0.869297	-1.015330	0.694310
17	6	0	1.617232	-2.630868	-0.372563
18	1	0	0.984297	-3.409438	-0.813543
19	1	0	2.641965	-3.001934	-0.354452
20	6	0	-0.822627	1.248112	0.376373
21	6	0	1.549310	-1.410598	-1.281310
22	1	0	2.072821	-1.578489	-2.219439
23	1	0	0.522716	-1.116831	-1.493358
24	6	0	-0.101425	2.558950	0.593153
25	1	0	-0.156699	3.200508	-0.284015
26	1	0	-0.531307	3.088841	1.441935
27	6	0	2.256119	2.204158	-0.260788
28	1	0	3.264395	2.101611	0.136870

29	1	0	2.186217	3.138549	-0.812721
30	6	0	1.916749	1.064420	-1.191439
31	1	0	0.874706	1.084164	-1.503717
32	1	0	2.539649	1.150602	-2.078982
33	8	0	-3.840281	-0.032697	-1.111495
34	1	0	3.194451	-0.413798	-0.502904
35	1	0	1.676624	3.113993	1.480028

Most stable energy, Gibbs free energy (Ha), and geometry for protomer ./OH_PyN3//2_8
E: -724.687014
G: -724.413828
Geometry:
Input orientation:

Center Number	Atomic Number	Atomic Type	Coordinates (Angstroms) X	Y	Z
1	6	0	-2.148904	-1.137975	0.280499
2	1	0	-2.587061	-2.060797	0.640940
3	6	0	-2.745329	0.084095	0.563485
4	6	0	-2.130007	1.259531	0.139434
5	1	0	-2.543839	2.225917	0.393435
6	7	0	-0.413459	-0.029893	-0.914993
7	7	0	1.157599	-2.425793	-0.914158
8	1	0	1.426969	-1.612094	-1.459337
9	7	0	2.760282	-0.089905	0.195706
10	1	0	3.679046	-0.496300	0.055222
11	7	0	1.254803	2.069722	-0.828788
12	1	0	1.828826	2.738552	-1.344815
13	6	0	-0.294639	-2.450547	-0.821170
14	1	0	-0.591137	-3.217334	-0.105020
15	1	0	-0.697384	-2.757517	-1.790994
16	6	0	-0.975729	-1.148050	-0.460321
17	6	0	1.857947	-2.366786	0.370442
18	1	0	2.871736	-2.750504	0.221133
19	1	0	1.362427	-3.052404	1.059848
20	6	0	-0.965847	1.138268	-0.590213
21	6	0	1.981603	-1.002034	1.034264
22	1	0	0.995688	-0.570807	1.211528
23	1	0	2.453772	-1.138093	2.017211
24	6	0	-0.190983	2.346822	-1.047189
25	1	0	-0.333858	2.523260	-2.112547
26	1	0	-0.461189	3.241158	-0.491185
27	6	0	1.657416	2.058693	0.608320
28	1	0	0.829694	1.626754	1.168342
29	1	0	1.797199	3.088148	0.928141
30	6	0	2.924080	1.239957	0.771386
31	1	0	3.744985	1.739807	0.255417
32	1	0	3.166535	1.216761	1.840783
33	8	0	-3.892639	0.191034	1.269361
34	1	0	-4.226905	-0.687319	1.499453
35	1	0	1.451926	1.122003	-1.188353

Most stable energy, Gibbs free energy (Ha), and geometry for protomer ./OH_PyN3//2_4
E: -724.696360
G: -724.420901
Geometry:
Input orientation:

Center Atomic Atomic Coordinates (Angstroms)

Number	Number	Type	X	Y	Z
1	6	0	-2.151055	-1.257057	0.091424
2	1	0	-2.535930	-2.226278	0.379481
3	6	0	-2.843423	-0.069064	0.522577
4	6	0	-2.193620	1.175797	0.203100
5	1	0	-2.615210	2.101994	0.570557
6	7	0	-0.517163	0.039962	-0.980396
7	7	0	1.235256	-2.067219	-0.834771
8	1	0	1.797588	-2.748632	-1.331625
9	7	0	2.723691	0.091656	0.387235
10	1	0	3.651693	0.522505	0.386874
11	7	0	1.140926	2.263008	-0.843053
12	1	0	1.574478	2.980793	-1.408067
13	6	0	-0.178430	-2.351078	-1.073021
14	1	0	-0.537824	-3.239759	-0.545098
15	1	0	-0.320493	-2.513578	-2.142915
16	6	0	-1.008509	-1.174925	-0.636710
17	6	0	1.575291	-2.099974	0.587330
18	1	0	1.720787	-3.117277	0.962556
19	1	0	0.746267	-1.674084	1.154707
20	6	0	-1.052944	1.199597	-0.533931
21	6	0	2.843463	-1.320161	0.861839
22	1	0	3.069378	-1.291267	1.925697
23	1	0	3.687965	-1.752190	0.327419
24	6	0	-0.306111	2.449798	-0.908790
25	1	0	-0.565444	2.703910	-1.938549
26	1	0	-0.657454	3.262172	-0.265203
27	6	0	1.694119	2.328109	0.510601
28	1	0	1.092444	2.965679	1.167086
29	1	0	2.691595	2.765969	0.452852
30	6	0	1.803627	0.965585	1.171302
31	1	0	2.223414	1.055803	2.171186
32	1	0	0.840662	0.464350	1.241936
33	8	0	-3.918011	-0.118853	1.169566
34	1	0	2.408465	0.074704	-0.588562
35	1	0	0.360965	0.079249	-1.485788

Most stable energy, Gibbs free energy (Ha), and geometry for protomer ./OH_PyN3//2_9
E: -724.687015
G: -724.413829
Geometry:
Input orientation:

Center Number	Atomic Number	Atomic Type	Coordinates (Angstroms) X	Y	Z
1	6	0	-2.131746	-1.257100	0.137919
2	1	0	-2.546727	-2.223084	0.391569
3	6	0	-2.746107	-0.081079	0.561726
4	6	0	-2.148294	1.140412	0.279193
5	1	0	-2.585740	2.063636	0.639470
6	7	0	-0.413268	0.030678	-0.915367
7	7	0	1.252810	-2.070687	-0.828336
8	1	0	1.826449	-2.739998	-1.344164
9	7	0	2.759731	0.087236	0.197621
10	1	0	3.678959	0.492837	0.057888
11	7	0	1.160349	2.424950	-0.912830
12	1	0	1.429350	1.611063	-1.457916

13	6	0	-0.193134	-2.346252	-1.047678
14	1	0	-0.464619	-3.240387	-0.491965
15	1	0	-0.335526	-2.522392	-2.113150
16	6	0	-0.967034	-1.136953	-0.591034
17	6	0	1.654564	-2.060391	0.609019
18	1	0	1.793151	-3.090058	0.928679
19	1	0	0.826931	-1.627774	1.168647
20	6	0	-0.974661	1.149364	-0.460912
21	6	0	2.921923	-1.242933	0.773035
22	1	0	3.163789	-1.220263	1.842578
23	1	0	3.742646	-1.743435	0.257403
24	6	0	-0.291950	2.451186	-0.821111
25	1	0	-0.693512	2.758639	-1.791271
26	1	0	-0.588283	3.218231	-0.105168
27	6	0	1.859509	2.364918	0.372378
28	1	0	1.364048	3.050812	1.061551
29	1	0	2.873797	2.747700	0.224062
30	6	0	1.981288	0.999840	1.035880
31	1	0	2.452826	1.135128	2.019239
32	1	0	0.994814	0.569518	1.212241
33	8	0	-3.893937	-0.186862	1.266933
34	1	0	-4.227346	0.691856	1.496877
35	1	0	1.451139	-1.123090	-1.187570

Most stable energy, Gibbs free energy (Ha), and geometry for protomer ./OH_PyN3//3_1
E: -725.132112
G: -724.843427
Geometry:

Input orientation:

Center Number	Atomic Number	Atomic Type	Coordinates (Angstroms) X	Y	Z
1	6	0	-2.334555	-1.007746	0.094243
2	1	0	-2.902246	-1.924945	0.009005
3	6	0	-2.966366	0.246530	-0.229565
4	6	0	-2.138636	1.413449	-0.050195
5	1	0	-2.556498	2.390699	-0.251155
6	7	0	-0.323417	0.090101	0.659301
7	7	0	0.390168	-2.914800	-0.290932
8	1	0	0.834250	-3.777800	0.037872
9	7	0	2.376415	-0.275826	0.495757
10	1	0	2.993595	-0.156141	1.293550
11	7	0	1.107994	2.675928	-0.405597
12	1	0	1.204982	3.684085	-0.546788
13	6	0	-0.333414	-2.317060	0.873739
14	1	0	0.396794	-2.151111	1.662795
15	1	0	-1.048725	-3.063580	1.207120
16	6	0	-1.043714	-1.045580	0.513529
17	6	0	1.433017	-2.071712	-0.962804
18	1	0	0.913802	-1.229780	-1.417245
19	1	0	1.823941	-2.694819	-1.762829
20	6	0	-0.853792	1.300865	0.377490
21	6	0	2.556100	-1.637847	-0.027075
22	1	0	2.629512	-2.330367	0.812497
23	1	0	3.494754	-1.708938	-0.584079
24	6	0	0.020391	2.494716	0.620279
25	1	0	-0.591816	3.390310	0.584753
26	1	0	0.506014	2.434100	1.592495

27	6	0	2.491006	2.155991	-0.098779
28	1	0	2.666393	2.316326	0.964088
29	1	0	3.160220	2.802567	-0.660541
30	6	0	2.743322	0.721039	-0.517064
31	1	0	2.196619	0.512015	-1.439700
32	1	0	3.806993	0.638277	-0.759934
33	8	0	-4.149279	0.318473	-0.630608
34	1	0	-0.300293	-3.188023	-0.996984
35	1	0	0.797476	2.297611	-1.307358
36	1	0	0.713676	-0.001043	0.832721

Most stable energy, Gibbs free energy (Ha), and geometry for protomer ./OH_PyN3//3_7
E: -724.916353
G: -724.627528
Geometry:
Input orientation:

Center Number	Atomic Number	Atomic Type	Coordinates (Angstroms) X	Y	Z
1	6	0	-2.175813	-1.170758	0.264578
2	1	0	-2.615347	-2.115188	0.566349
3	6	0	-2.740744	0.042697	0.668066
4	6	0	-2.119317	1.241506	0.313146
5	1	0	-2.514691	2.196123	0.646004
6	7	0	-0.440969	0.021009	-0.889236
7	7	0	1.150861	-2.164819	-0.780867
8	1	0	1.669446	-2.896185	-1.277147
9	7	0	2.753036	0.001519	0.140779
10	1	0	3.685626	0.366428	-0.042634
11	7	0	1.208153	2.211725	-0.981311
12	1	0	1.598075	2.904005	-1.626286
13	6	0	-0.316113	-2.376059	-0.949823
14	1	0	-0.611571	-3.242905	-0.358167
15	1	0	-0.498848	-2.575462	-2.007953
16	6	0	-1.025253	-1.120812	-0.505626
17	6	0	1.597439	-2.109527	0.646625
18	1	0	1.715711	-3.133134	1.002013
19	1	0	0.800093	-1.628839	1.217450
20	6	0	-0.972520	1.169651	-0.465182
21	6	0	2.897748	-1.329170	0.737456
22	1	0	3.190815	-1.289038	1.795804
23	1	0	3.681954	-1.862361	0.192958
24	6	0	-0.269143	2.423418	-0.917164
25	1	0	-0.594876	2.683400	-1.927360
26	1	0	-0.468092	3.264008	-0.252576
27	6	0	1.950687	2.300631	0.324678
28	1	0	1.436251	3.030052	0.950868
29	1	0	2.946093	2.679249	0.083884
30	6	0	2.057720	0.951924	1.011054
31	1	0	2.586555	1.113063	1.961220
32	1	0	1.063897	0.564449	1.254575
33	8	0	-3.860394	0.000610	1.421841
34	1	0	-4.147813	0.897549	1.654378
35	1	0	1.396686	-1.255351	-1.205119
36	1	0	1.385819	1.279197	-1.387457

Most stable energy, Gibbs free energy (Ha), and geometry for protomer ./OH_PyN3//3_5
E: -725.136665
G: -724.849683

Geometry:

Input orientation:

Center Number	Atomic Number	Atomic Type	Coordinates (Angstroms) X	Y	Z
1	6	0	-2.117867	-1.390951	0.325503
2	1	0	-2.561676	-2.313295	0.677033
3	6	0	-2.863456	-0.219290	0.267549
4	6	0	-2.270510	0.969012	-0.188153
5	1	0	-2.839814	1.886499	-0.238316
6	7	0	-0.273068	-0.223921	-0.520350
7	7	0	1.331693	-2.415471	-0.759705
8	1	0	1.496878	-3.252632	-1.301093
9	7	0	2.419868	0.277946	-0.339740
10	1	0	3.141072	0.117048	-1.036339
11	7	0	0.408586	2.886902	0.092646
12	1	0	-0.343205	3.244288	0.690052
13	6	0	0.093222	-2.580036	-0.018920
14	1	0	0.268061	-2.801691	1.041782
15	1	0	-0.477382	-3.416932	-0.420960
16	6	0	-0.799065	-1.368598	-0.076324
17	6	0	2.514730	-2.154419	0.062249
18	1	0	3.387696	-2.257545	-0.584259
19	1	0	2.614569	-2.883781	0.874975
20	6	0	-0.960721	0.933664	-0.569311
21	6	0	2.523492	-0.769485	0.681654
22	1	0	1.684665	-0.667496	1.377821
23	1	0	3.438189	-0.659592	1.274649
24	6	0	-0.205572	2.137223	-1.046800
25	1	0	0.593765	1.859692	-1.729055
26	1	0	-0.880869	2.827066	-1.545780
27	6	0	1.384277	2.147500	0.962271
28	1	0	0.830963	1.361306	1.472841
29	1	0	1.696461	2.874445	1.707603
30	6	0	2.597823	1.624847	0.208482
31	1	0	2.840691	2.306826	-0.608670
32	1	0	3.439078	1.648607	0.908856
33	8	0	-4.145222	-0.160828	0.627089
34	1	0	-4.462512	-1.025833	0.927506
35	1	0	0.883949	3.703755	-0.303944
36	1	0	0.758007	-0.174067	-0.742054

Most stable energy, Gibbs free energy (Ha), and geometry for protomer ./OH_PyN3//3_2

E: -725.137677

G: -724.846372

Geometry:

Input orientation:

Center Number	Atomic Number	Atomic Type	Coordinates (Angstroms) X	Y	Z
1	6	0	-1.945289	-1.526110	-0.041561
2	1	0	-2.178186	-2.545758	-0.317910
3	6	0	-2.802821	-0.463770	-0.496038
4	6	0	-2.356638	0.867823	-0.159857
5	1	0	-2.921136	1.719534	-0.515057
6	7	0	-0.526959	0.004189	1.045268
7	7	0	1.496509	-1.926540	0.813610

8	1	0	2.147570	-2.535238	1.297602
9	7	0	2.850914	0.218746	-0.338295
10	1	0	2.629531	0.045976	0.651168
11	7	0	0.282424	2.928680	-0.093115
12	1	0	0.383332	3.935048	0.066912
13	6	0	0.132784	-2.329098	1.150053
14	1	0	0.065822	-2.440888	2.233624
15	1	0	-0.161838	-3.277491	0.691417
16	6	0	-0.837622	-1.272422	0.701102
17	6	0	1.759079	-1.976014	-0.624762
18	1	0	0.878935	-1.607290	-1.153385
19	1	0	1.942283	-2.990502	-0.990136
20	6	0	-1.240952	1.065179	0.583663
21	6	0	2.967910	-1.129170	-0.975353
22	1	0	3.882280	-1.566618	-0.579495
23	1	0	3.067324	-0.989691	-2.049320
24	6	0	-0.735160	2.439041	0.902112
25	1	0	-1.559538	3.145478	0.862958
26	1	0	-0.262163	2.483905	1.881059
27	6	0	1.646991	2.317498	-0.000775
28	1	0	1.795769	2.053248	1.046962
29	1	0	2.363384	3.089146	-0.267659
30	6	0	1.800015	1.102796	-0.907776
31	1	0	0.880532	0.527520	-0.981125
32	1	0	2.109402	1.384662	-1.911034
33	8	0	-3.841185	-0.669785	-1.160417
34	1	0	3.755862	0.694794	-0.377455
35	1	0	-0.095562	2.828216	-1.042018
36	1	0	0.326085	0.159027	1.575316

Most stable energy, Gibbs free energy (Ha), and geometry for protomer ./OH_PyN3//3_9
E: -725.142674
G: -724.854124
Geometry:

Input orientation:

Center Number	Atomic Number	Atomic Type	Coordinates (Angstroms) X	Y	Z
1	6	0	-2.269083	-1.198084	0.081747
2	1	0	-2.747771	-2.167235	0.015780
3	6	0	-2.942101	-0.050440	-0.325253
4	6	0	-2.290611	1.172303	-0.264715
5	1	0	-2.779326	2.081059	-0.590290
6	7	0	-0.345429	0.100189	0.635477
7	7	0	1.193380	-2.224428	0.762372
8	1	0	1.655195	-3.012180	1.202122
9	7	0	2.761391	-0.145113	-0.124418
10	1	0	2.130137	-0.328375	0.675091
11	7	0	1.106118	2.364733	0.824594
12	1	0	1.120875	1.546194	1.445585
13	6	0	-0.237743	-2.319167	1.003754
14	1	0	-0.394130	-2.444586	2.077714
15	1	0	-0.694818	-3.182672	0.507941
16	6	0	-0.975143	-1.079828	0.555008
17	6	0	1.541955	-2.188909	-0.656707
18	1	0	0.764680	-1.647292	-1.198734
19	1	0	1.622359	-3.180345	-1.109073
20	6	0	-0.996080	1.183520	0.220411

21	6	0	2.865684	-1.467248	-0.818020
22	1	0	3.667654	-2.015038	-0.327685
23	1	0	3.127699	-1.294089	-1.859048
24	6	0	-0.280611	2.508062	0.298296
25	1	0	-0.225767	2.980537	-0.681200
26	1	0	-0.814050	3.176920	0.970980
27	6	0	2.190149	2.265977	-0.203772
28	1	0	3.130781	2.434744	0.318692
29	1	0	2.029596	3.082148	-0.903394
30	6	0	2.230376	0.961816	-0.964515
31	1	0	1.258914	0.664029	-1.353617
32	1	0	2.917599	1.086205	-1.798578
33	8	0	-4.206209	-0.077433	-0.796983
34	1	0	-4.549322	-0.982253	-0.787490
35	1	0	3.673396	0.129858	0.246186
36	1	0	1.316817	3.180904	1.402540

Most stable energy, Gibbs free energy (Ha), and geometry for protomer ./OH_PyN3//3_6
E: -725.136665
G: -724.849689
Geometry:
Input orientation:

Center Number	Atomic Number	Atomic Type	Coordinates (Angstroms) X	Y	Z
1	6	0	-2.270463	-0.969282	0.189409
2	1	0	-2.839595	-1.886858	0.239886
3	6	0	-2.863791	0.218886	-0.266146
4	6	0	-2.118415	1.390659	-0.324509
5	1	0	-2.562508	2.312903	-0.675945
6	7	0	-0.273084	0.224009	0.520707
7	7	0	0.408744	-2.886820	-0.092283
8	1	0	0.884411	-3.703554	0.304185
9	7	0	2.419857	-0.277491	0.338861
10	1	0	3.141398	-0.116404	1.035067
11	7	0	1.331472	2.415813	0.759093
12	1	0	1.496813	3.253049	1.300318
13	6	0	-0.205020	-2.137093	1.047343
14	1	0	0.594572	-1.859390	1.729229
15	1	0	-0.880025	-2.826965	1.546677
16	6	0	-0.960531	-0.933686	0.570042
17	6	0	1.383906	-2.147349	-0.962444
18	1	0	0.830199	-1.361302	-1.472819
19	1	0	1.695845	-2.874315	-1.707859
20	6	0	-0.799446	1.368559	0.076784
21	6	0	2.597744	-1.624420	-0.209308
22	1	0	2.841141	-2.306266	0.607799
23	1	0	3.438651	-1.648125	-0.910103
24	6	0	0.092610	2.580146	0.018911
25	1	0	0.266886	2.801803	-1.041885
26	1	0	-0.477936	3.416955	0.421211
27	6	0	2.514130	2.154848	-0.063437
28	1	0	3.387412	2.258174	0.582613
29	1	0	2.613446	2.884139	-0.876290
30	6	0	2.522786	0.769847	-0.682698
31	1	0	1.683616	0.667645	-1.378418
32	1	0	3.437195	0.660036	-1.276153
33	8	0	-4.145694	0.160208	-0.625153

34	1	0	-4.463220	1.025162	-0.925468
35	1	0	-0.343260	-3.244390	-0.689312
36	1	0	0.758085	0.174346	0.741979

Most stable energy, Gibbs free energy (Ha), and geometry for protomer ./OH_PyN3//3_10
E: -725.142675
G: -724.854090
Geometry:
Input orientation:

Center Number	Atomic Number	Atomic Type	Coordinates (Angstroms) X	Y	Z
1	6	0	-2.291875	-1.167255	0.265984
2	1	0	-2.782053	-2.074841	0.592603
3	6	0	-2.940722	0.056862	0.326803
4	6	0	-2.265749	1.202927	-0.081460
5	1	0	-2.742257	2.173130	-0.015185
6	7	0	-0.345508	-0.099621	-0.637008
7	7	0	1.102022	-2.367071	-0.824098
8	1	0	1.311750	-3.183905	-1.401478
9	7	0	2.760668	0.139866	0.125169
10	1	0	3.672186	-0.137223	-0.245076
11	7	0	1.197889	2.222195	-0.763792
12	1	0	1.661329	3.008908	-1.203691
13	6	0	-0.285539	-2.507535	-0.299287
14	1	0	-0.232887	-2.981124	0.679784
15	1	0	-0.819936	-3.174442	-0.973167
16	6	0	-0.997952	-1.181390	-0.220777
17	6	0	2.185134	-2.270020	0.205438
18	1	0	3.126026	-2.440772	-0.315930
19	1	0	2.022182	-3.085592	0.905197
20	6	0	-0.972648	1.081778	-0.556184
21	6	0	2.227138	-0.965569	0.965605
22	1	0	1.255921	-0.665910	1.353896
23	1	0	2.913602	-1.090738	1.800177
24	6	0	-0.232962	2.319337	-1.006004
25	1	0	-0.388599	2.443822	-2.080183
26	1	0	-0.688710	3.184197	-0.511341
27	6	0	1.545635	2.186759	0.655520
28	1	0	0.766885	1.647180	1.197476
29	1	0	1.628017	3.178283	1.107330
30	6	0	2.867640	1.462221	0.817937
31	1	0	3.671062	2.007856	0.327581
32	1	0	3.128815	1.289181	1.859194
33	8	0	-4.204142	0.086842	0.800168
34	1	0	-4.545225	0.992437	0.790780
35	1	0	2.130033	0.323979	-0.674632
36	1	0	1.119000	-1.548872	-1.445480

Most stable energy, Gibbs free energy (Ha), and geometry for protomer ./OH_PyN3//3_8
E: -725.147090
G: -724.859049
Geometry:
Input orientation:

Center Number	Atomic Number	Atomic Type	Coordinates (Angstroms) X	Y	Z
1	6	0	-2.271241	-1.220240	-0.050615

2	1	0	-2.729102	-2.174031	-0.270863
3	6	0	-2.905731	-0.026012	-0.415117
4	6	0	-2.279573	1.203735	-0.196322
5	1	0	-2.739673	2.127263	-0.521772
6	7	0	-0.508476	0.053055	0.797130
7	7	0	1.196211	-2.040332	0.809155
8	1	0	1.739487	-2.695444	1.359898
9	7	0	2.890014	0.017424	-0.285452
10	1	0	2.515207	0.003195	0.669405
11	7	0	1.169128	2.189623	0.843638
12	1	0	1.552536	2.829467	1.524975
13	6	0	-0.220419	-2.342636	0.966471
14	1	0	-0.420085	-2.554827	2.018446
15	1	0	-0.552146	-3.206366	0.382320
16	6	0	-1.047731	-1.155278	0.556484
17	6	0	1.625265	-2.099890	-0.588503
18	1	0	0.855930	-1.636092	-1.208595
19	1	0	1.740393	-3.126346	-0.948935
20	6	0	-1.052485	1.215839	0.416688
21	6	0	2.949855	-1.392264	-0.779231
22	1	0	3.736179	-1.882914	-0.208082
23	1	0	3.234015	-1.361394	-1.828804
24	6	0	-0.255584	2.458387	0.708390
25	1	0	-0.479167	3.192804	-0.071008
26	1	0	-0.625781	2.863443	1.652314
27	6	0	1.938651	2.291446	-0.398300
28	1	0	2.933147	2.667024	-0.154064
29	1	0	1.484403	2.997404	-1.100086
30	6	0	2.082612	0.961399	-1.113390
31	1	0	1.116887	0.500287	-1.310786
32	1	0	2.608436	1.083779	-2.057896
33	8	0	-4.095597	-0.125792	-1.005698
34	1	0	-4.448151	0.746451	-1.238615
35	1	0	3.841512	0.387044	-0.218342
36	1	0	0.414440	0.088693	1.229009

Most stable energy, Gibbs free energy (Ha), and geometry for protomer ./OH_PyN3//3_3

E: -725.137677

G: -724.846375

Geometry:

Input orientation:

Center Number	Atomic Number	Atomic Type	Coordinates (Angstroms) X	Y	Z
1	6	0	-2.355620	-0.868743	-0.160671
2	1	0	-2.919643	-1.720906	-0.515537
3	6	0	-2.802300	0.462455	-0.497748
4	6	0	-1.945385	1.525432	-0.043615
5	1	0	-2.178653	2.544821	-0.320610
6	7	0	-0.526728	-0.003602	1.044576
7	7	0	0.284205	-2.928552	-0.091634
8	1	0	0.385325	-3.934801	0.069008
9	7	0	2.851657	-0.217735	-0.337153
10	1	0	2.629576	-0.044434	0.652064
11	7	0	1.495931	1.927914	0.812702
12	1	0	2.146492	2.537296	1.296503
13	6	0	-0.733904	-2.438627	0.902899
14	1	0	-0.261226	-2.482649	1.882039

15	1	0	-1.558005	-3.145399	0.863953
16	6	0	-1.240107	-1.065175	0.583356
17	6	0	1.648545	-2.316920	0.000918
18	1	0	2.365271	-3.088512	-0.265231
19	1	0	1.796797	-2.052046	1.048565
20	6	0	-0.837858	1.272668	0.699571
21	6	0	1.801580	-1.102695	-0.906719
22	1	0	2.111752	-1.385033	-1.909601
23	1	0	0.881849	-0.527931	-0.981029
24	6	0	0.131871	2.330069	1.148259
25	1	0	-0.162975	3.278016	0.688846
26	1	0	0.064370	2.442567	2.231724
27	6	0	1.759067	1.976411	-0.625597
28	1	0	1.941997	2.990699	-0.991661
29	1	0	0.879290	1.606918	-1.154293
30	6	0	2.968399	1.129808	-0.975046
31	1	0	3.068378	0.989654	-2.048874
32	1	0	3.882403	1.567897	-0.579051
33	8	0	-3.840583	0.667634	-1.162516
34	1	0	3.756873	-0.693347	-0.375423
35	1	0	-0.093405	-2.828740	-1.040759
36	1	0	0.326132	-0.157729	1.575149

Most stable energy, Gibbs free energy (Ha), and geometry for protomer ./OH_PyN3//3_4
E: -725.125244
G: -724.834334
Geometry:
Input orientation:

Center Number	Atomic Number	Atomic Type	Coordinates (Angstroms) X	Y	Z
1	6	0	-2.308505	-0.957509	-0.001491
2	1	0	-2.862380	-1.883308	-0.104814
3	6	0	-2.827071	0.239436	-0.594529
4	6	0	-1.988386	1.382373	-0.444555
5	1	0	-2.282919	2.323638	-0.893443
6	7	0	-0.375262	0.171123	0.851415
7	7	0	0.288861	-2.872944	0.076348
8	1	0	0.843805	-3.632015	0.483639
9	7	0	3.017034	-0.376644	-0.721831
10	1	0	3.910129	-0.188453	-0.256103
11	7	0	1.374692	2.093289	1.043292
12	1	0	1.198762	1.184970	1.502592
13	6	0	-0.494110	-2.203920	1.182231
14	1	0	0.197655	-2.005219	1.995217
15	1	0	-1.234147	-2.932929	1.500722
16	6	0	-1.117052	-0.936135	0.675303
17	6	0	1.167596	-1.993173	-0.744162
18	1	0	0.526441	-1.243087	-1.200815
19	1	0	1.586073	-2.616745	-1.532313
20	6	0	-0.818783	1.288404	0.272078
21	6	0	2.278528	-1.388365	0.092402
22	1	0	1.915827	-0.900181	0.991097
23	1	0	3.002570	-2.150864	0.372132
24	6	0	0.063217	2.503835	0.457802
25	1	0	0.245005	3.016514	-0.484974
26	1	0	-0.396897	3.205724	1.150573
27	6	0	2.509025	1.977553	0.077822

28	1	0	3.408824	1.793686	0.663009
29	1	0	2.600983	2.954015	-0.390471
30	6	0	2.352828	0.940199	-1.019989
31	1	0	1.318650	0.756729	-1.301866
32	1	0	2.870274	1.317982	-1.897864
33	8	0	-3.936246	0.267892	-1.215496
34	1	0	3.257585	-0.807935	-1.620421
35	1	0	-0.381025	-3.308332	-0.564196
36	1	0	1.654326	2.765623	1.758972

Most stable energy, Gibbs free energy (Ha), and geometry for protomer ./OH_PyN3//4_2
E: -725.578226
G: -725.275967
Geometry:
Input orientation:

Center Number	Atomic Number	Atomic Type	Coordinates (Angstroms) X	Y	Z
1	6	0	-2.324182	-1.022521	-0.094106
2	1	0	-2.905941	-1.931471	-0.032327
3	6	0	-2.912466	0.217935	0.169549
4	6	0	-2.150543	1.383942	0.050633
5	1	0	-2.594689	2.353949	0.231539
6	7	0	-0.291893	0.076903	-0.554036
7	7	0	0.461380	-2.923685	0.328384
8	1	0	-0.200843	-3.195319	1.061924
9	7	0	2.329730	-0.236814	-0.527873
10	1	0	2.850625	-0.093138	-1.389116
11	7	0	1.136102	2.683716	0.440034
12	1	0	0.871445	2.297963	1.353385
13	6	0	-0.297742	-2.332216	-0.814217
14	1	0	-1.025636	-3.077214	-1.122531
15	1	0	0.402936	-2.169369	-1.629959
16	6	0	-1.002627	-1.057529	-0.453075
17	6	0	1.535554	-2.082716	0.953033
18	1	0	1.991166	-2.721968	1.704231
19	1	0	1.037985	-1.262438	1.467856
20	6	0	-0.831108	1.279082	-0.314415
21	6	0	2.581643	-1.606966	-0.047198
22	1	0	3.557382	-1.653435	0.441329
23	1	0	2.619570	-2.280107	-0.904022
24	6	0	0.019197	2.494589	-0.546482
25	1	0	0.467237	2.459935	-1.538064
26	1	0	-0.611746	3.374940	-0.476774
27	6	0	2.511319	2.187135	0.071529
28	1	0	3.196300	2.829580	0.618594
29	1	0	2.643197	2.365750	-0.994862
30	6	0	2.788942	0.751102	0.459744
31	1	0	3.868106	0.656128	0.603453
32	1	0	2.318978	0.535168	1.422946
33	8	0	-4.195602	0.230969	0.512879
34	1	0	-4.515706	1.135068	0.656510
35	1	0	0.890284	-3.788548	-0.016969
36	1	0	1.222638	3.693365	0.581540
37	1	0	0.807098	-0.017136	-0.710693

Most stable energy, Gibbs free energy (Ha), and geometry for protomer ./OH_PyN3//4_1
E: -725.578292
G: -725.270911

Geometry:

Input orientation:

Center Number	Atomic Number	Atomic Type	Coordinates (Angstroms) X	Y	Z
1	6	0	-1.928804	-1.343507	0.248570
2	1	0	-2.264973	-2.280315	0.672714
3	6	0	-2.561621	-0.113391	0.666710
4	6	0	-2.006535	1.086648	0.087129
5	1	0	-2.397537	2.049570	0.390452
6	7	0	-0.479151	-0.172194	-1.190418
7	7	0	0.973658	-2.870209	-0.153477
8	1	0	0.618897	-3.045833	0.793815
9	7	0	2.771618	0.267152	0.985463
10	1	0	2.879979	0.658329	1.927576
11	7	0	0.457609	2.976553	-0.269748
12	1	0	-0.218380	3.527020	0.269876
13	6	0	-0.188058	-2.582768	-1.064285
14	1	0	-0.845558	-3.445145	-1.004871
15	1	0	0.218488	-2.496858	-2.070238
16	6	0	-0.919245	-1.341308	-0.654228
17	6	0	2.060419	-1.838464	-0.112943
18	1	0	2.059717	-1.316734	-1.067891
19	1	0	3.003184	-2.371179	-0.015834
20	6	0	-0.983357	1.027209	-0.799621
21	6	0	1.861839	-0.915314	1.077505
22	1	0	0.840474	-0.548903	1.162737
23	1	0	2.110884	-1.436514	1.999310
24	6	0	-0.292757	2.240919	-1.348889
25	1	0	0.419465	1.976596	-2.126225
26	1	0	-1.010248	2.949883	-1.753540
27	6	0	1.229817	2.161631	0.712210
28	1	0	0.509691	1.534031	1.232353
29	1	0	1.649161	2.862309	1.430647
30	6	0	2.338487	1.356132	0.054974
31	1	0	2.047247	0.885951	-0.880593
32	1	0	3.208850	1.978691	-0.135859
33	8	0	-3.500078	-0.090674	1.485108
34	1	0	3.707734	-0.045689	0.704630
35	1	0	1.385659	-3.752147	-0.472683
36	1	0	1.080955	3.650627	-0.726537
37	1	0	0.249763	-0.194791	-1.897015

Most stable energy, Gibbs free energy (Ha), and geometry for protomer ./OH_PyN3//4_3

E: -725.582393

G: -725.278345

Geometry:

Input orientation:

Center Number	Atomic Number	Atomic Type	Coordinates (Angstroms) X	Y	Z
1	6	0	-2.030917	-1.423055	-0.063966
2	1	0	-2.344428	-2.419103	-0.346755
3	6	0	-2.766130	-0.302716	-0.453898
4	6	0	-2.302729	0.981559	-0.132535
5	1	0	-2.843649	1.857506	-0.461501
6	7	0	-0.498289	-0.001235	0.995782

7	7	0	1.418388	-1.993230	0.785401
8	1	0	2.038780	-2.619393	1.287153
9	7	0	2.928992	0.096582	-0.317820
10	1	0	2.692723	-0.062558	0.669607
11	7	0	0.458511	2.897667	-0.068422
12	1	0	0.590540	3.901043	0.091349
13	6	0	0.037339	-2.365022	1.068721
14	1	0	-0.070885	-2.530297	2.142176
15	1	0	-0.286100	-3.272070	0.550429
16	6	0	-0.878657	-1.246290	0.658740
17	6	0	1.722001	-2.038726	-0.646242
18	1	0	0.875275	-1.621997	-1.194336
19	1	0	1.862812	-3.058021	-1.016578
20	6	0	-1.152008	1.108358	0.590318
21	6	0	2.982014	-1.254440	-0.958224
22	1	0	3.861406	-1.743770	-0.544165
23	1	0	3.113977	-1.116832	-2.028902
24	6	0	-0.572389	2.446941	0.927755
25	1	0	-1.363602	3.191628	0.911124
26	1	0	-0.093325	2.445851	1.904490
27	6	0	1.803132	2.242637	0.031401
28	1	0	1.928710	1.957239	1.076477
29	1	0	2.544754	2.998369	-0.210291
30	6	0	1.936327	1.041127	-0.897239
31	1	0	0.995790	0.509818	-1.021007
32	1	0	2.291692	1.331401	-1.882548
33	8	0	-3.887072	-0.384166	-1.155209
34	1	0	-4.124297	-1.305876	-1.341134
35	1	0	3.858563	0.523821	-0.346630
36	1	0	0.080737	2.811221	-1.019458
37	1	0	0.372757	0.087368	1.521833

Most stable energy, Gibbs free energy (Ha), and geometry for protomer ./OH_PyN3//4_5
E: -725.590962
G: -725.285660
Geometry:
Input orientation:

Center Number	Atomic Number	Atomic Type	Coordinates (Angstroms) X	Y	Z
1	6	0	-2.258545	-0.912666	0.185657
2	1	0	-2.822864	-1.834466	0.236133
3	6	0	-2.820472	0.235060	-0.375792
4	6	0	-2.064565	1.396465	-0.437469
5	1	0	-2.469957	2.301767	-0.871957
6	7	0	-0.235486	0.283473	0.620835
7	7	0	0.276304	-2.879023	0.049820
8	1	0	0.790593	-3.670774	0.449550
9	7	0	2.538419	-0.407103	0.227000
10	1	0	1.769638	-0.307588	0.902204
11	7	0	1.339793	2.477105	0.751964
12	1	0	1.185105	1.833943	1.536721
13	6	0	-0.274022	-2.060895	1.183773
14	1	0	0.552425	-1.801370	1.840922
15	1	0	-0.959509	-2.713754	1.718220
16	6	0	-0.968340	-0.838038	0.658948
17	6	0	1.151067	-2.188978	-0.951289
18	1	0	0.577159	-1.377462	-1.393293

19	1	0	1.331862	-2.926909	-1.728662
20	6	0	-0.777283	1.364734	0.071576
21	6	0	2.499351	-1.762004	-0.408788
22	1	0	2.866094	-2.470930	0.332323
23	1	0	3.208010	-1.729079	-1.233470
24	6	0	0.068362	2.607600	-0.017383
25	1	0	0.325275	2.824147	-1.054366
26	1	0	-0.474540	3.459183	0.385965
27	6	0	2.549311	2.070828	-0.035568
28	1	0	3.392451	2.096523	0.653313
29	1	0	2.689653	2.837781	-0.793688
30	6	0	2.453824	0.732983	-0.730563
31	1	0	1.543333	0.631601	-1.316218
32	1	0	3.306132	0.645737	-1.401289
33	8	0	-4.078856	0.156759	-0.839576
34	1	0	-4.355488	1.003192	-1.220072
35	1	0	3.419049	-0.331729	0.745509
36	1	0	-0.515896	-3.276746	-0.464936
37	1	0	1.555945	3.387232	1.165954

Most stable energy, Gibbs free energy (Ha), and geometry for protomer ./OH_PyN3//4_4
E: -725.582395
G: -725.278298
Geometry:
Input orientation:

Center Number	Atomic Number	Atomic Type	Coordinates (Angstroms) X	Y	Z
1	6	0	-2.304069	-0.979868	0.131964
2	1	0	-2.845790	-1.855302	0.460968
3	6	0	-2.766589	0.304881	0.452643
4	6	0	-2.030236	1.424477	0.062778
5	1	0	-2.342988	2.420888	0.345123
6	7	0	-0.498383	0.001091	-0.995947
7	7	0	0.456090	-2.897822	0.069895
8	1	0	0.078072	-2.810419	1.020738
9	7	0	2.928450	-0.098464	0.318352
10	1	0	3.857765	-0.526258	0.347159
11	7	0	1.419716	1.992017	-0.785994
12	1	0	2.040497	2.617757	-1.287800
13	6	0	-0.574222	-2.446987	-0.926896
14	1	0	-1.365909	-3.191168	-0.910080
15	1	0	-0.094847	-2.446676	-1.903481
16	6	0	-1.153089	-1.107841	-0.590319
17	6	0	1.801274	-2.243909	-0.030018
18	1	0	1.927174	-1.958976	-1.075205
19	1	0	2.542271	-3.000165	0.211949
20	6	0	-0.877884	1.246564	-0.659500
21	6	0	1.935264	-1.042182	0.898204
22	1	0	0.995069	-0.510217	1.021785
23	1	0	2.290530	-1.332296	1.883596
24	6	0	0.038931	2.364511	-1.069706
25	1	0	-0.069005	2.529416	-2.143254
26	1	0	-0.284031	3.271987	-0.551867
27	6	0	1.723041	2.037885	0.645724
28	1	0	0.875881	1.622025	1.193822
29	1	0	1.864568	3.057232	1.015635
30	6	0	2.982397	1.252742	0.958270

31	1	0	3.862234	1.741246	0.544183
32	1	0	3.114054	1.115471	2.029029
33	8	0	-3.887861	0.387597	1.153339
34	1	0	-4.124550	1.309645	1.338313
35	1	0	2.692119	0.060554	-0.669087
36	1	0	0.587400	-3.901410	-0.089119
37	1	0	0.372665	-0.088412	-1.521863

Most stable energy, Gibbs free energy (Ha), and geometry for protomer ./Cl_PyN3//0_1
E: -1108.578083
G: -1108.335681
Geometry:
Input orientation:

Center Number	Atomic Number	Atomic Type	Coordinates (Angstroms) X	Y	Z
1	6	0	0.374401	-1.148406	-0.843941
2	6	0	1.635040	-1.200475	-0.259263
3	6	0	1.632808	1.203690	-0.257694
4	6	0	0.372260	1.150046	-0.842509
5	7	0	-0.215060	0.000497	-1.166076
6	1	0	2.094995	-2.146492	-0.008153
7	1	0	2.090997	2.150243	-0.005394
8	6	0	-0.413416	2.410333	-1.134527
9	1	0	0.018015	3.242537	-0.577435
10	1	0	-0.281199	2.636398	-2.196381
11	6	0	-0.409250	-2.409641	-1.137235
12	1	0	0.024380	-3.242051	-0.582173
13	1	0	-0.278053	-2.633556	-2.199663
14	7	0	-1.843420	2.335014	-0.851364
15	1	0	-2.166385	1.431176	-1.187507
16	7	0	-1.839041	-2.337411	-0.852187
17	1	0	-2.164314	-1.434101	-1.187527
18	6	0	-2.184745	2.417197	0.571041
19	1	0	-3.266306	2.559163	0.643717
20	1	0	-1.720471	3.316275	0.982187
21	6	0	-1.801305	1.222737	1.437199
22	1	0	-0.721302	1.070076	1.403329
23	1	0	-2.046083	1.459488	2.482098
24	6	0	-2.178515	-2.420960	0.570583
25	1	0	-3.259573	-2.566142	0.644444
26	1	0	-1.711137	-3.318765	0.980998
27	6	0	-1.797730	-1.225573	1.436668
28	1	0	-0.718176	-1.069945	1.402229
29	1	0	-2.041294	-1.463152	2.481670
30	7	0	-2.446872	-0.002269	0.975712
31	1	0	-3.411604	-0.003754	1.290930
32	6	0	2.261847	0.002032	0.011057
33	17	0	3.835864	0.003014	0.739697

Most stable energy, Gibbs free energy (Ha), and geometry for protomer ./Cl_PyN3//1_3
E: -1109.045983
G: -1108.787284
Geometry:
Input orientation:

Center Number	Atomic Number	Atomic Type	Coordinates (Angstroms) X	Y	Z

1	6	0	0.564081	-1.111651	0.693033
2	6	0	1.778765	-1.219461	0.039258
3	6	0	1.769235	1.180986	-0.122985
4	6	0	0.545483	1.177845	0.540081
5	7	0	-0.021194	0.051849	0.962218
6	1	0	2.220506	-2.183125	-0.172225
7	1	0	2.214660	2.107794	-0.458342
8	6	0	-0.177972	2.472186	0.841829
9	1	0	0.177524	2.806924	1.820956
10	1	0	0.136796	3.228746	0.122647
11	6	0	-0.215659	-2.329316	1.114223
12	1	0	-0.131299	-2.493720	2.187704
13	1	0	0.106201	-3.221781	0.583074
14	7	0	-1.630744	2.418966	0.869814
15	1	0	-1.912084	1.608374	1.413363
16	7	0	-1.650342	-2.084901	0.806787
17	1	0	-1.897341	-1.143162	1.150803
18	6	0	-2.272831	2.338567	-0.443683
19	1	0	-1.765787	3.035925	-1.112431
20	1	0	-3.301583	2.695960	-0.339041
21	6	0	-2.331772	0.970253	-1.108646
22	1	0	-2.758358	1.093622	-2.113767
23	1	0	-1.328558	0.561962	-1.237748
24	6	0	-1.967432	-2.085405	-0.652342
25	1	0	-1.117584	-1.637758	-1.164963
26	1	0	-2.067870	-3.118178	-0.975986
27	6	0	-3.238228	-1.291081	-0.888297
28	1	0	-4.077140	-1.805206	-0.417302
29	1	0	-3.421472	-1.273141	-1.969388
30	7	0	-3.129266	0.041828	-0.306564
31	1	0	-4.061630	0.429243	-0.208738
32	6	0	2.383300	-0.035057	-0.351906
33	17	0	3.903717	-0.087708	-1.174680
34	1	0	-2.237970	-2.767436	1.288718

Most stable energy, Gibbs free energy (Ha), and geometry for protomer ./Cl_PyN3//1_2
E: -1109.045983
G: -1108.787285
Geometry:

Input orientation:

Center Number	Atomic Number	Atomic Type	Coordinates (Angstroms) X	Y	Z
1	6	0	0.545947	-1.177402	-0.539983
2	6	0	1.769692	-1.180024	0.123098
3	6	0	1.778279	1.220419	-0.039279
4	6	0	0.563645	1.112094	-0.693059
5	7	0	-0.021168	-0.051651	-0.962189
6	1	0	2.215484	-2.106636	0.458511
7	1	0	2.219634	2.184268	0.172165
8	6	0	-0.216582	2.329432	-1.114293
9	1	0	0.104919	3.222039	-0.583165
10	1	0	-0.132289	2.493841	-2.187778
11	6	0	-0.176988	-2.472048	-0.841672
12	1	0	0.138039	-3.228430	-0.122417
13	1	0	0.178694	-2.806720	-1.820754
14	7	0	-1.651166	2.084450	-0.806846
15	1	0	-2.239073	2.766726	-1.288803

16	7	0	-1.629779	-2.419400	-0.869741
17	1	0	-1.911412	-1.608938	-1.413330
18	6	0	-1.968266	2.084881	0.652280
19	1	0	-2.069102	3.117625	0.975893
20	1	0	-1.118255	1.637572	1.164924
21	6	0	-3.238764	1.290077	0.888237
22	1	0	-3.422015	1.272084	1.969325
23	1	0	-4.077865	1.803875	0.417222
24	6	0	-2.271976	-2.339217	0.443713
25	1	0	-3.300588	-2.696996	0.339015
26	1	0	-1.764713	-3.036374	1.112505
27	6	0	-2.331471	-0.970914	1.108652
28	1	0	-1.328419	-0.562235	1.237783
29	1	0	-2.758048	-1.094429	2.113758
30	7	0	-3.129291	-0.042803	0.306527
31	1	0	-4.061509	-0.430566	0.208688
32	6	0	2.383273	0.036275	0.351961
33	17	0	3.903645	0.089583	1.174774
34	1	0	-1.897789	1.142597	-1.150821

Most stable energy, Gibbs free energy (Ha), and geometry for protomer ./Cl_PyN3//1_1
E: -1108.814199
G: -1108.555320
Geometry:
Input orientation:

Center Number	Atomic Number	Atomic Type	Coordinates (Angstroms) X	Y	Z
1	6	0	0.458396	-1.175903	0.363006
2	6	0	1.594104	-1.187176	-0.449386
3	6	0	1.560827	1.226025	-0.366794
4	6	0	0.429522	1.134005	0.442904
5	7	0	-0.090858	-0.043913	0.814757
6	1	0	2.017209	-2.121474	-0.803016
7	1	0	1.957984	2.191421	-0.660956
8	6	0	-0.238368	2.401714	0.940398
9	1	0	0.183045	2.629312	1.925386
10	1	0	0.045914	3.224856	0.268481
11	6	0	-0.175286	-2.490608	0.791570
12	1	0	0.397773	-2.850043	1.654427
13	1	0	-0.031243	-3.225656	-0.006236
14	7	0	-1.686243	2.287877	1.091275
15	1	0	-1.990217	2.988131	1.759338
16	7	0	-1.584478	-2.467435	1.151980
17	1	0	-1.764789	-1.710214	1.806573
18	6	0	-2.443015	2.480411	-0.146902
19	1	0	-2.081201	3.334385	-0.737908
20	1	0	-3.483535	2.676635	0.125677
21	6	0	-2.384896	1.249072	-1.040333
22	1	0	-3.097169	1.322080	-1.863450
23	1	0	-1.385900	1.090862	-1.455224
24	6	0	-2.526393	-2.432723	0.035619
25	1	0	-2.385274	-3.339109	-0.561148
26	1	0	-3.538414	-2.474158	0.449979
27	6	0	-2.439706	-1.256511	-0.933664
28	1	0	-3.177679	-1.364741	-1.731038
29	1	0	-1.449320	-1.172785	-1.387135
30	7	0	-2.713004	0.034911	-0.239008

31	1	0	-3.694381	0.069076	0.058295
32	6	0	2.141071	0.039007	-0.794761
33	17	0	3.554825	0.094775	-1.812235
34	1	0	-2.121530	0.091581	0.610039

Most stable energy, Gibbs free energy (Ha), and geometry for protomer ./Cl_PyN3//1_4
E: -1109.051232
G: -1108.793049
Geometry:
Input orientation:

Center Number	Atomic Number	Atomic Type	Coordinates (Angstroms) X	Y	Z
1	6	0	0.423866	-1.134775	0.443385
2	6	0	1.609830	-1.236542	-0.271202
3	6	0	1.675194	1.164543	-0.348885
4	6	0	0.482025	1.167756	0.364511
5	7	0	-0.109412	0.045056	0.768483
6	1	0	2.020138	-2.202815	-0.530462
7	1	0	2.136856	2.090632	-0.663917
8	6	0	-0.160589	2.491391	0.736680
9	1	0	0.402137	2.880003	1.590111
10	1	0	-0.002660	3.196012	-0.081501
11	6	0	-0.277420	-2.399016	0.892720
12	1	0	0.140061	-2.669416	1.865313
13	1	0	-0.007494	-3.201457	0.195749
14	7	0	-1.571490	2.482309	1.074150
15	1	0	-1.769856	1.731712	1.726261
16	7	0	-1.719135	-2.268329	1.046587
17	1	0	-2.032434	-2.959834	1.714561
18	6	0	-2.494522	2.454621	-0.053389
19	1	0	-2.333208	3.352403	-0.652118
20	1	0	-3.510289	2.510449	0.341582
21	6	0	-2.406224	1.272309	-1.008063
22	1	0	-3.125650	1.381161	-1.817919
23	1	0	-1.411512	1.172846	-1.441006
24	6	0	-2.480101	-2.446175	-0.186887
25	1	0	-2.143394	-3.311406	-0.770144
26	1	0	-3.522721	-2.611668	0.085918
27	6	0	-2.389860	-1.227955	-1.087465
28	1	0	-3.089413	-1.297482	-1.917578
29	1	0	-1.385648	-1.093291	-1.489262
30	7	0	-2.705167	-0.003488	-0.305139
31	1	0	-3.685038	-0.019427	-0.011200
32	6	0	2.233621	-0.061774	-0.651113
33	17	0	3.715485	-0.133362	-1.543801
34	1	0	-2.112680	-0.054969	0.541272

Most stable energy, Gibbs free energy (Ha), and geometry for protomer ./Cl_PyN3//2_1
E: -1109.501063
G: -1109.227532
Geometry:
Input orientation:

Center Number	Atomic Number	Atomic Type	Coordinates (Angstroms) X	Y	Z
1	6	0	0.603466	-1.050489	0.643396
2	6	0	1.949816	-1.131344	0.318709

3	6	0	1.938006	1.240709	-0.044399
4	6	0	0.592050	1.214360	0.290765
5	7	0	-0.059269	0.110829	0.637032
6	1	0	2.459480	-2.084948	0.321670
7	1	0	2.428431	2.165797	-0.315039
8	6	0	-0.171853	2.512084	0.257680
9	1	0	0.327914	3.251076	0.880259
10	1	0	-0.224835	2.901811	-0.758381
11	6	0	-0.135239	-2.311115	1.024017
12	1	0	-0.008814	-2.469717	2.097545
13	1	0	0.352213	-3.151286	0.516350
14	7	0	-1.563287	2.359377	0.770183
15	1	0	-1.583580	1.553492	1.406575
16	7	0	-1.556975	-2.228649	0.738230
17	1	0	-2.022825	-3.024914	1.158241
18	6	0	-2.631630	2.230948	-0.270873
19	1	0	-2.482248	3.049783	-0.969770
20	1	0	-3.583265	2.376380	0.238552
21	6	0	-2.621951	0.925113	-1.028881
22	1	0	-3.281861	1.034178	-1.886759
23	1	0	-1.632414	0.645444	-1.384849
24	6	0	-1.854917	-2.195136	-0.692427
25	1	0	-1.075563	-1.626546	-1.202536
26	1	0	-1.885104	-3.186594	-1.151198
27	6	0	-3.194835	-1.518369	-0.900445
28	1	0	-3.994638	-2.092901	-0.437759
29	1	0	-3.425207	-1.355204	-1.950581
30	7	0	-3.162078	-0.192229	-0.207409
31	1	0	-4.099937	0.058694	0.112915
32	6	0	2.611385	0.036086	-0.017353
33	17	0	4.290321	-0.017210	-0.422166
34	1	0	-1.791029	3.182779	1.331584
35	1	0	-2.569825	-0.352422	0.624821

Most stable energy, Gibbs free energy (Ha), and geometry for protomer ./Cl_PyN3//2_6

E: -1109.487133

G: -1109.214165

Geometry:

Input orientation:

Center Number	Atomic Number	Atomic Type	Coordinates (Angstroms) X	Y	Z
1	6	0	0.400622	-1.024153	0.867163
2	6	0	1.685693	-1.117796	0.395837
3	6	0	1.709851	1.281977	0.070835
4	6	0	0.413163	1.323977	0.549738
5	7	0	-0.168211	0.192531	0.950323
6	1	0	2.165181	-2.082718	0.318892
7	1	0	2.195454	2.191591	-0.253441
8	6	0	-0.415870	2.575626	0.660106
9	1	0	-0.048205	3.126540	1.528021
10	1	0	-0.210864	3.190194	-0.223303
11	6	0	-0.353218	-2.227622	1.347568
12	1	0	-0.455329	-2.178342	2.431912
13	1	0	0.198154	-3.125631	1.081871
14	7	0	-1.822425	2.263440	0.835366
15	1	0	-2.233141	2.941398	1.463113
16	7	0	-1.730107	-2.371596	0.794537

17	1	0	-2.310490	-1.563186	1.047233
18	6	0	-2.583814	2.249092	-0.419908
19	1	0	-2.465704	3.194611	-0.962233
20	1	0	-3.639179	2.140878	-0.165770
21	6	0	-2.169188	1.124134	-1.350795
22	1	0	-2.674514	1.267429	-2.312001
23	1	0	-1.097973	1.191649	-1.563077
24	6	0	-1.860641	-2.568020	-0.685889
25	1	0	-1.127629	-3.312188	-0.988519
26	1	0	-2.862837	-2.966116	-0.834167
27	6	0	-1.687673	-1.260314	-1.437065
28	1	0	-1.991667	-1.442419	-2.474027
29	1	0	-0.635795	-0.969487	-1.470613
30	7	0	-2.439530	-0.197033	-0.785618
31	1	0	-3.434117	-0.393040	-0.862546
32	6	0	2.338296	0.053628	0.011334
33	17	0	3.945253	-0.048768	-0.577236
34	1	0	-1.149297	0.323252	1.231206
35	1	0	-2.143000	-3.182684	1.262921

Most stable energy, Gibbs free energy (Ha), and geometry for protomer ./Cl_PyN3//2_2
E: -1109.501062
G: -1109.227545
Geometry:

Input orientation:

Center Number	Atomic Number	Atomic Type	Coordinates (Angstroms) X	Y	Z
1	6	0	0.591841	-1.214923	0.291406
2	6	0	1.938273	-1.242141	-0.041735
3	6	0	1.951215	1.129822	0.321943
4	6	0	0.604321	1.049837	0.644623
5	7	0	-0.059204	-0.111005	0.636994
6	1	0	2.428459	-2.167499	-0.311876
7	1	0	2.461535	2.083071	0.325896
8	6	0	-0.133996	2.310945	1.024419
9	1	0	-0.008120	2.469817	2.097970
10	1	0	0.354233	3.150701	0.516799
11	6	0	-0.173162	-2.511956	0.256384
12	1	0	0.325993	-3.252402	0.877702
13	1	0	-0.226587	-2.899977	-0.760315
14	7	0	-1.555593	2.229192	0.737794
15	1	0	-2.021264	3.025747	1.157456
16	7	0	-1.564454	-2.358738	0.769186
17	1	0	-1.584327	-1.552826	1.405562
18	6	0	-1.852674	2.195776	-0.693044
19	1	0	-1.073426	1.626570	-1.202618
20	1	0	-1.881842	3.187225	-1.151905
21	6	0	-3.192963	1.520004	-0.901851
22	1	0	-3.992622	2.095160	-0.439689
23	1	0	-3.422799	1.356973	-1.952127
24	6	0	-2.632819	-2.229671	-0.271737
25	1	0	-2.484171	-3.048735	-0.970522
26	1	0	-3.584541	-2.374223	0.237785
27	6	0	-2.621961	-0.923935	-1.029893
28	1	0	-3.281573	-1.032594	-1.888049
29	1	0	-1.632067	-0.644972	-1.385437
30	7	0	-3.161659	0.193864	-0.208747

31	1	0	-4.099911	-0.056322	0.111007
32	6	0	2.612455	-0.037981	-0.013417
33	17	0	4.292020	0.014233	-0.415778
34	1	0	-1.792495	-3.182048	1.330595
35	1	0	-2.569814	0.353589	0.623854

Most stable energy, Gibbs free energy (Ha), and geometry for protomer ./Cl_PyN3//2_5
E: -1109.487134
G: -1109.214151
Geometry:
Input orientation:

Center Number	Atomic Number	Atomic Type	Coordinates (Angstroms) X	Y	Z
1	6	0	0.415724	-1.322370	0.549644
2	6	0	1.712427	-1.279036	0.070932
3	6	0	1.684707	1.121028	0.393368
4	6	0	0.399731	1.026045	0.864741
5	7	0	-0.167392	-0.191356	0.948989
6	1	0	2.199471	-2.188288	-0.252215
7	1	0	2.162848	2.086548	0.315531
8	6	0	-0.355097	2.229118	1.344598
9	1	0	-0.455174	2.181182	2.429198
10	1	0	0.194641	3.127505	1.076776
11	6	0	-0.411484	-2.575146	0.661143
12	1	0	-0.042287	-3.125252	1.528916
13	1	0	-0.206327	-3.189719	-0.222236
14	7	0	-1.733182	2.370686	0.793954
15	1	0	-2.312087	1.561902	1.048787
16	7	0	-1.818335	-2.264967	0.837420
17	1	0	-2.227473	-2.942957	1.466154
18	6	0	-1.866818	2.565629	-0.686391
19	1	0	-1.135981	3.311194	-0.990845
20	1	0	-2.870155	2.961433	-0.833091
21	6	0	-1.692398	1.257861	-1.437002
22	1	0	-1.998126	1.438798	-2.473668
23	1	0	-0.639995	0.969078	-1.471840
24	6	0	-2.580934	-2.252681	-0.417123
25	1	0	-2.461607	-3.198256	-0.959085
26	1	0	-3.636252	-2.146265	-0.162040
27	6	0	-2.169164	-1.127412	-1.348892
28	1	0	-2.675075	-1.271971	-2.309605
29	1	0	-1.098007	-1.193150	-1.562066
30	7	0	-2.441304	0.193495	-0.783971
31	1	0	-3.436378	0.387544	-0.859537
32	6	0	2.339059	-0.049819	0.010173
33	17	0	3.945964	0.054359	-0.578239
34	1	0	-1.148399	-0.323304	1.229670
35	1	0	-2.146191	3.181747	1.262303

Most stable energy, Gibbs free energy (Ha), and geometry for protomer ./Cl_PyN3//2_4
E: -1109.500010
G: -1109.227056
Geometry:
Input orientation:

Center Number	Atomic Number	Atomic Type	Coordinates (Angstroms) X	Y	Z

1	6	0	0.682923	-1.139221	-0.695229
2	6	0	1.994279	-1.183161	-0.284492
3	6	0	1.974165	1.238504	-0.102252
4	6	0	0.659401	1.231109	-0.518468
5	7	0	0.097140	0.059640	-0.821030
6	1	0	2.496189	-2.132332	-0.164425
7	1	0	2.458860	2.170146	0.152367
8	6	0	-0.198911	2.459246	-0.663813
9	1	0	0.020084	3.118440	0.181559
10	1	0	0.136824	2.974528	-1.565991
11	6	0	-0.171820	-2.337941	-1.002221
12	1	0	0.201807	-3.179806	-0.410934
13	1	0	-0.039057	-2.588938	-2.056322
14	7	0	-1.615513	2.149000	-0.786498
15	1	0	-2.018066	2.745432	-1.495105
16	7	0	-1.573220	-2.029709	-0.760248
17	1	0	-2.153242	-2.680518	-1.277704
18	6	0	-2.396267	2.268686	0.447182
19	1	0	-3.399514	2.606148	0.184154
20	1	0	-1.968547	3.010704	1.127259
21	6	0	-2.501601	0.958396	1.202737
22	1	0	-1.520820	0.527810	1.397238
23	1	0	-3.015933	1.094297	2.151535
24	6	0	-1.918634	-2.085148	0.660371
25	1	0	-1.967123	-3.110258	1.039600
26	1	0	-1.136419	-1.578311	1.228873
27	6	0	-3.262757	-1.437288	0.915294
28	1	0	-3.493259	-1.412445	1.978159
29	1	0	-4.053267	-1.969441	0.388806
30	7	0	-3.297925	-0.029044	0.414119
31	1	0	-2.970174	-0.027845	-0.558122
32	6	0	2.631595	0.021428	-0.006669
33	17	0	4.269236	-0.004804	0.509344
34	1	0	-0.880278	0.081222	-1.120998
35	1	0	-4.269407	0.290907	0.390796

Most stable energy, Gibbs free energy (Ha), and geometry for protomer ./Cl_PyN3//3_2
E: -1109.932886
G: -1109.643312
Geometry:
Input orientation:

Center Number	Atomic Number	Atomic Type	Coordinates (Angstroms) X	Y	Z
1	6	0	0.526931	-1.160291	0.859084
2	6	0	1.772998	-1.273088	0.277219
3	6	0	1.899954	1.140663	0.178829
4	6	0	0.660904	1.208713	0.764298
5	7	0	0.043329	0.063596	1.113153
6	1	0	2.177261	-2.252817	0.065804
7	1	0	2.414227	2.048497	-0.102891
8	6	0	-0.034155	2.515075	1.007432
9	1	0	-0.601915	2.497677	1.935628
10	1	0	0.709675	3.306261	1.048328
11	6	0	-0.360657	-2.326908	1.188842
12	1	0	-0.355908	-2.471327	2.270826
13	1	0	0.071796	-3.218035	0.725539
14	7	0	-0.992146	2.887017	-0.090885

15	1	0	-1.208378	3.879239	0.047012
16	7	0	-1.720726	-2.039911	0.752706
17	1	0	-2.358042	-2.684250	1.208016
18	6	0	-2.292675	2.141526	-0.125981
19	1	0	-3.050078	2.839126	-0.471499
20	1	0	-2.520321	1.867325	0.904180
21	6	0	-2.235727	0.916947	-1.030747
22	1	0	-2.484455	1.165237	-2.059386
23	1	0	-1.257594	0.442899	-1.028162
24	6	0	-1.867705	-2.135881	-0.700906
25	1	0	-0.991563	-1.680687	-1.166497
26	1	0	-1.908296	-3.169623	-1.054804
27	6	0	-3.131608	-1.437014	-1.164445
28	1	0	-4.019498	-1.975599	-0.838693
29	1	0	-3.152783	-1.322026	-2.245563
30	7	0	-3.233181	-0.076231	-0.549585
31	1	0	-4.176480	0.292905	-0.694606
32	6	0	2.459022	-0.114968	-0.045941
33	17	0	4.003984	-0.220814	-0.770991
34	1	0	-0.887655	0.106693	1.537647
35	1	0	-0.518346	2.826342	-1.000250
36	1	0	-3.104200	-0.207815	0.461723

Most stable energy, Gibbs free energy (Ha), and geometry for protomer ./Cl_PyN3//3_3
E: -1109.932887
G: -1109.643330
Geometry:
Input orientation:

Center Number	Atomic Number	Atomic Type	Coordinates (Angstroms) X	Y	Z
1	6	0	0.661972	-1.207080	-0.763955
2	6	0	1.900976	-1.138368	-0.178495
3	6	0	1.772209	1.275308	-0.275771
4	6	0	0.526203	1.161847	-0.857655
5	7	0	0.043495	-0.062274	-1.112325
6	1	0	2.415892	-2.045958	0.102827
7	1	0	2.175740	2.255240	-0.063895
8	6	0	-0.362194	2.327994	-1.186902
9	1	0	0.069105	3.219033	-0.722349
10	1	0	-0.356666	2.473528	-2.268736
11	6	0	-0.031946	-2.513940	-1.007621
12	1	0	0.712591	-3.304459	-1.048645
13	1	0	-0.599517	-2.496752	-1.935933
14	7	0	-1.722350	2.039364	-0.752169
15	1	0	-2.359906	2.683447	-1.207505
16	7	0	-0.989818	-2.887157	0.090363
17	1	0	-0.516427	-2.825886	0.999891
18	6	0	-1.870676	2.134055	0.701392
19	1	0	-1.912107	3.167489	1.056101
20	1	0	-0.994738	1.678904	1.167400
21	6	0	-3.134574	1.434132	1.163291
22	1	0	-3.156856	1.318808	2.244353
23	1	0	-4.022491	1.972177	0.836725
24	6	0	-2.291487	-2.143566	0.124978
25	1	0	-2.518980	-1.869449	-0.905236
26	1	0	-3.048019	-2.842401	0.469893
27	6	0	-2.236853	-0.919129	1.030090

28	1	0	-1.259075	-0.444334	1.028956
29	1	0	-2.486753	-1.167852	2.058338
30	7	0	-3.234443	0.073452	0.547931
31	1	0	-3.104321	0.205399	-0.463191
32	6	0	2.459119	0.117574	0.046830
33	17	0	4.004011	0.224226	0.771908
34	1	0	-0.887542	-0.105830	-1.536690
35	1	0	-1.204610	-3.879678	-0.047621
36	1	0	-4.177634	-0.296440	0.691675

Most stable energy, Gibbs free energy (Ha), and geometry for protomer ./NMe2_PyN3//0_1

E: -782.940409

G: -782.615820

Geometry:

Input orientation:

Center Number	Atomic Number	Atomic Type	Coordinates (Angstroms) X	Y	Z
1	6	0	0.046665	-1.316021	0.845975
2	6	0	1.320867	-1.376130	0.314975
3	6	0	2.089089	-0.199908	0.208054
4	6	0	1.452433	0.992876	0.595981
5	6	0	0.167973	0.950836	1.110816
6	7	0	-0.513775	-0.184689	1.285649
7	1	0	1.695357	-2.328673	-0.030374
8	1	0	1.938382	1.952595	0.490474
9	6	0	-0.539261	2.243130	1.461498
10	1	0	-1.459934	2.002565	1.994091
11	1	0	0.098826	2.811237	2.141479
12	6	0	-0.802808	-2.567300	0.956484
13	1	0	-0.666236	-2.967478	1.965168
14	1	0	-0.422041	-3.320195	0.264538
15	7	0	-0.876924	3.115861	0.335190
16	1	0	-0.051557	3.228439	-0.246227
17	7	0	-2.232719	-2.401537	0.723921
18	1	0	-2.554761	-1.609996	1.271502
19	6	0	-1.992500	2.684591	-0.510486
20	1	0	-2.204044	3.515118	-1.184351
21	1	0	-2.870375	2.572157	0.130429
22	6	0	-1.821084	1.409338	-1.347342
23	1	0	-2.186430	1.585879	-2.361795
24	1	0	-0.746128	1.192028	-1.439891
25	6	0	-2.616330	-2.215394	-0.677698
26	1	0	-2.285833	-3.098280	-1.229481
27	1	0	-3.707593	-2.203837	-0.717585
28	6	0	-2.089257	-0.984246	-1.402831
29	1	0	-2.440713	-1.025339	-2.437960
30	1	0	-0.991403	-1.023516	-1.447978
31	7	0	-2.549020	0.263311	-0.799933
32	1	0	-2.302600	0.213952	0.185793
33	7	0	3.374528	-0.214897	-0.259581
34	6	0	3.847977	-1.407576	-0.944325
35	1	0	4.887083	-1.258797	-1.225160
36	1	0	3.801540	-2.275835	-0.288497
37	1	0	3.266592	-1.622634	-1.847692
38	6	0	3.970732	1.045126	-0.675175
39	1	0	4.985065	0.855621	-1.014987
40	1	0	3.409286	1.517161	-1.488957

41	1	0	4.024044	1.744194	0.158433

Most stable energy, Gibbs free energy (Ha), and geometry for protomer ./NMe2_PyN3//1_3
E: -783.408224
G: -783.069456
Geometry:
Input orientation:

Center Number	Atomic Number	Atomic Type	Coordinates (Angstroms) X	Y	Z
1	6	0	0.284296	-1.127462	-1.008509
2	6	0	1.555281	-1.149198	-0.471843
3	6	0	2.197648	0.061961	-0.134270
4	6	0	1.438315	1.239018	-0.304227
5	6	0	0.184354	1.143598	-0.869672
6	7	0	-0.381526	0.005231	-1.259663
7	1	0	2.025554	-2.103749	-0.287514
8	1	0	1.805370	2.205725	0.006645
9	6	0	-0.653955	2.379919	-1.082857
10	1	0	-0.393566	3.174028	-0.387287
11	1	0	-0.524792	2.746351	-2.100485
12	6	0	-0.462576	-2.405709	-1.318442
13	1	0	0.063461	-3.250580	-0.873124
14	1	0	-0.442428	-2.549387	-2.401948
15	7	0	-2.101644	2.050684	-0.916596
16	1	0	-2.650647	2.584683	-1.589549
17	7	0	-1.856853	-2.433225	-0.883994
18	1	0	-2.291175	-1.572083	-1.205583
19	6	0	-2.660577	2.264383	0.453817
20	1	0	-3.744370	2.233055	0.352834
21	1	0	-2.359698	3.255918	0.784783
22	6	0	-2.178540	1.178854	1.393206
23	1	0	-1.087928	1.195878	1.446252
24	1	0	-2.545057	1.416255	2.397426
25	6	0	-2.042697	-2.502450	0.566906
26	1	0	-3.079358	-2.794477	0.756133
27	1	0	-1.413628	-3.306648	0.954422
28	6	0	-1.761789	-1.232590	1.363579
29	1	0	-0.720550	-0.929362	1.247322
30	1	0	-1.906854	-1.456825	2.429077
31	7	0	-2.605352	-0.128746	0.912162
32	1	0	-3.560676	-0.296677	1.211423
33	7	0	3.463748	0.090523	0.356870
34	6	0	3.965953	1.320766	0.947145
35	1	0	4.999263	1.168286	1.245325
36	1	0	3.941724	2.135631	0.224089
37	1	0	3.386179	1.619254	1.826627
38	6	0	4.091165	-1.155082	0.768396
39	1	0	5.104340	-0.942889	1.097011
40	1	0	3.548279	-1.635786	1.588593
41	1	0	4.148202	-1.854194	-0.065465
42	1	0	-2.199657	1.044613	-1.138722

Most stable energy, Gibbs free energy (Ha), and geometry for protomer ./NMe2_PyN3//1_2
E: -783.412020
G: -783.075581
Geometry:
Input orientation:

Center Number	Atomic Number	Atomic Type	Coordinates (Angstroms) X	Y	Z
1	6	0	0.373605	-1.179705	0.231920
2	6	0	1.727338	-1.207167	0.046375
3	6	0	2.462513	-0.000378	-0.055811
4	6	0	1.726760	1.206242	0.044169
5	6	0	0.373324	1.178357	0.231948
6	7	0	-0.276207	-0.000734	0.337145
7	1	0	2.214098	-2.168497	-0.017552
8	1	0	2.213242	2.167919	-0.016723
9	6	0	-0.421377	2.462013	0.284994
10	1	0	0.201046	3.196357	0.795011
11	1	0	-0.529803	2.814913	-0.748118
12	6	0	-0.421401	-2.463334	0.279889
13	1	0	0.199831	-3.199362	0.788914
14	1	0	-0.527925	-2.813048	-0.754533
15	7	0	-1.712593	2.354708	0.946885
16	1	0	-1.789157	3.104042	1.620924
17	7	0	-1.713991	-2.357779	0.939416
18	1	0	-1.792199	-3.109414	1.610700
19	6	0	-2.859223	2.435801	0.039393
20	1	0	-2.818028	3.332886	-0.591011
21	1	0	-3.760036	2.504257	0.651938
22	6	0	-2.965445	1.225373	-0.868863
23	1	0	-3.828534	1.352982	-1.533565
24	1	0	-2.082322	1.164490	-1.514594
25	6	0	-2.858787	-2.435462	0.029309
26	1	0	-2.816000	-3.329843	-0.604825
27	1	0	-3.760759	-2.506905	0.639797
28	6	0	-2.963912	-1.221260	-0.874014
29	1	0	-3.825969	-1.346285	-1.540557
30	1	0	-2.079805	-1.157405	-1.518138
31	7	0	-3.026902	0.000368	-0.088124
32	1	0	-3.854773	-0.001410	0.499738
33	7	0	3.789031	-0.000369	-0.228997
34	6	0	4.518723	-1.258724	-0.296166
35	1	0	4.372539	-1.844435	0.613198
36	1	0	4.195918	-1.856181	-1.151022
37	1	0	5.576833	-1.043708	-0.401983
38	6	0	4.513454	1.258238	-0.335530
39	1	0	4.415151	1.847666	0.578284
40	1	0	5.564314	1.044182	-0.499630
41	1	0	4.145072	1.851950	-1.174001
42	1	0	-1.321095	-0.000752	0.457904

Most stable energy, Gibbs free energy (Ha), and geometry for protomer ./NMe2_PyN3//1_1

E: -783.383798

G: -783.044389

Geometry:

Input orientation:

Center Number	Atomic Number	Atomic Type	Coordinates (Angstroms) X	Y	Z
1	6	0	0.292019	-1.009795	-0.990925
2	6	0	1.558157	-0.951066	-0.420659
3	6	0	2.050725	0.299998	-0.108012
4	6	0	1.302103	1.436881	-0.320799

5	6	0	0.043419	1.276346	-0.894253
6	7	0	-0.418246	0.084424	-1.258701
7	1	0	2.110376	-1.857303	-0.208623
8	1	0	1.665440	2.414856	-0.030241
9	6	0	-0.882456	2.452366	-1.112114
10	1	0	-0.518598	3.308814	-0.543752
11	1	0	-0.822269	2.722310	-2.170042
12	6	0	-0.363956	-2.332455	-1.319460
13	1	0	0.180916	-3.138032	-0.826518
14	1	0	-0.259655	-2.488449	-2.396759
15	7	0	-2.281444	2.211814	-0.772249
16	1	0	-2.524259	1.292777	-1.133671
17	7	0	-1.777972	-2.429550	-0.971263
18	1	0	-2.218088	-1.557949	-1.255216
19	6	0	-2.563988	2.202421	0.665526
20	1	0	-3.650112	2.222930	0.787871
21	1	0	-2.180405	3.130332	1.095045
22	6	0	-2.017204	1.024135	1.463925
23	1	0	-0.928711	0.994616	1.387476
24	1	0	-2.246416	1.189502	2.525982
25	6	0	-2.040955	-2.604957	0.459371
26	1	0	-3.095285	-2.871752	0.569997
27	1	0	-1.460032	-3.460475	0.810754
28	6	0	-1.755119	-1.409097	1.361278
29	1	0	-0.698666	-1.141303	1.302210
30	1	0	-1.937956	-1.709804	2.402402
31	7	0	-2.541151	-0.244235	0.968848
32	1	0	-3.492435	-0.360561	1.302986
33	7	0	3.371955	0.428355	0.520019
34	6	0	3.395609	-0.175100	1.887640
35	1	0	2.594627	0.270094	2.471298
36	1	0	3.253130	-1.247731	1.788287
37	1	0	4.364730	0.042849	2.327965
38	6	0	4.463554	-0.127756	-0.334752
39	1	0	4.409359	0.344957	-1.311201
40	1	0	5.408625	0.096044	0.152323
41	1	0	4.322532	-1.201664	-0.415465
42	1	0	3.554425	1.429964	0.630432

Most stable energy, Gibbs free energy (Ha), and geometry for protomer ./NMe2_PyN3//1_4

E: -783.408223

G: -783.069458

Geometry:

Input orientation:

Center Number	Atomic Number	Atomic Type	Coordinates (Angstroms) X	Y	Z
1	6	0	0.183120	-1.143824	-0.869058
2	6	0	1.437622	-1.239617	-0.304882
3	6	0	2.197659	-0.062840	-0.136090
4	6	0	1.555399	1.148504	-0.473305
5	6	0	0.283842	1.127165	-1.008571
6	7	0	-0.382714	-0.005340	-1.258744
7	1	0	1.804593	-2.206388	0.005889
8	1	0	2.026285	2.102901	-0.289748
9	6	0	-0.462861	2.405616	-1.318069
10	1	0	0.064080	3.250430	-0.873719
11	1	0	-0.444019	2.548857	-2.401654

12	6	0	-0.655919	-2.379855	-1.081084
13	1	0	-0.395343	-3.173834	-0.385435
14	1	0	-0.527721	-2.746700	-2.098682
15	7	0	-1.856596	2.433866	-0.881875
16	1	0	-2.291663	1.572837	-1.202763
17	7	0	-2.103316	-2.049866	-0.913802
18	1	0	-2.653092	-2.583667	-1.586278
19	6	0	-2.040524	2.503395	0.569249
20	1	0	-3.076690	2.796310	0.759802
21	1	0	-1.410282	3.307099	0.955882
22	6	0	-1.759612	1.233399	1.365705
23	1	0	-0.718705	0.929460	1.248333
24	1	0	-1.903328	1.457875	2.431338
25	6	0	-2.661295	-2.263079	0.457058
26	1	0	-3.745150	-2.231186	0.356947
27	1	0	-2.360684	-3.254728	0.787934
28	6	0	-2.177892	-1.177765	1.395999
29	1	0	-1.087244	-1.195406	1.448114
30	1	0	-2.543686	-1.414986	2.400521
31	7	0	-2.604401	0.130073	0.915364
32	1	0	-3.559277	0.298648	1.215678
33	7	0	3.464285	-0.091744	0.353648
34	6	0	4.092618	1.153753	0.764154
35	1	0	3.550500	1.635133	1.584452
36	1	0	5.105884	0.941183	1.092238
37	1	0	4.149473	1.852437	-0.070090
38	6	0	3.966710	-1.321953	0.943832
39	1	0	5.000643	-1.169968	1.240094
40	1	0	3.388263	-1.619487	1.824511
41	1	0	3.940658	-2.137248	0.221337
42	1	0	-2.200902	-1.043778	-1.136089

Most stable energy, Gibbs free energy (Ha), and geometry for protomer ./NMe2_PyN3//2_7
E: -783.855135
G: -783.501189
Geometry:

Input orientation:

Center Number	Atomic Number	Atomic Type	Coordinates (Angstroms) X	Y	Z
1	6	0	0.196239	-1.252590	-0.576117
2	6	0	1.516964	-1.391068	-0.159735
3	6	0	2.250353	-0.239287	0.016536
4	6	0	1.696455	1.006449	-0.194296
5	6	0	0.371950	1.049523	-0.611606
6	7	0	-0.341014	-0.058595	-0.808512
7	1	0	1.946781	-2.368321	0.024349
8	1	0	2.255763	1.920661	-0.041772
9	6	0	-0.267984	2.399037	-0.866814
10	1	0	0.005090	3.059022	-0.033269
11	1	0	0.211557	2.812686	-1.756199
12	6	0	-0.638070	-2.499584	-0.784727
13	1	0	-0.466275	-3.161323	0.074181
14	1	0	-0.224671	-3.013165	-1.655036
15	7	0	-1.704676	2.371340	-1.091408
16	1	0	-1.937574	3.102121	-1.750298
17	7	0	-2.054313	-2.265741	-1.018497
18	1	0	-2.392978	-2.972699	-1.657020

19	6	0	-2.507588	2.577212	0.110747
20	1	0	-3.536583	2.762169	-0.199695
21	1	0	-2.173061	3.443138	0.694855
22	6	0	-2.472279	1.377603	1.033803
23	1	0	-1.468406	1.196852	1.419085
24	1	0	-3.146206	1.513687	1.876976
25	6	0	-2.881726	-2.311151	0.183735
26	1	0	-3.926031	-2.347895	-0.128433
27	1	0	-2.683589	-3.199576	0.795646
28	6	0	-2.666112	-1.103614	1.071402
29	1	0	-1.647027	-1.067362	1.456698
30	1	0	-3.354185	-1.109997	1.914308
31	7	0	-2.876303	0.149600	0.302859
32	1	0	-2.268941	0.089504	-0.536513
33	7	0	3.648296	-0.350533	0.449612
34	6	0	4.594298	0.177824	-0.580093
35	1	0	4.393777	-0.328472	-1.520052
36	1	0	4.434279	1.248274	-0.673250
37	1	0	5.604044	-0.028293	-0.236260
38	6	0	3.878091	0.293721	1.778366
39	1	0	4.903950	0.088979	2.071399
40	1	0	3.718720	1.362796	1.670332
41	1	0	3.178186	-0.133425	2.490972
42	1	0	-3.851576	0.221211	0.003574
43	1	0	3.848982	-1.348621	0.559913

Most stable energy, Gibbs free energy (Ha), and geometry for protomer ./NMe2_PyN3//2_1
E: -783.855131
G: -783.501249
Geometry:
Input orientation:

Center Number	Atomic Number	Atomic Type	Coordinates (Angstroms) X	Y	Z
1	6	0	0.202784	-1.251577	0.572152
2	6	0	1.523118	-1.393299	0.155586
3	6	0	2.258601	-0.243301	-0.023232
4	6	0	1.707137	1.003907	0.185539
5	6	0	0.382993	1.050259	0.603539
6	7	0	-0.332007	-0.056165	0.802780
7	1	0	1.951066	-2.371685	-0.026756
8	1	0	2.268192	1.916742	0.031195
9	6	0	-0.254269	2.401361	0.856989
10	1	0	0.226095	2.815122	1.745882
11	1	0	0.020086	3.059820	0.022653
12	6	0	-0.633873	-2.496661	0.782865
13	1	0	-0.220772	-3.010228	1.653322
14	1	0	-0.464093	-3.159653	-0.075492
15	7	0	-1.690970	2.376706	1.081748
16	1	0	-1.922501	3.108966	1.739468
17	7	0	-2.049464	-2.259773	1.017405
18	1	0	-2.388970	-2.964826	1.657571
19	6	0	-2.493717	2.581913	-0.120600
20	1	0	-2.157539	3.446011	-0.706488
21	1	0	-3.522253	2.769625	0.189711
22	6	0	-2.461185	1.380475	-1.041410
23	1	0	-3.135323	1.516253	-1.884461
24	1	0	-1.457886	1.197026	-1.426881

25	6	0	-2.877910	-2.305765	-0.184075
26	1	0	-2.682254	-3.195851	-0.794367
27	1	0	-3.922061	-2.339523	0.128939
28	6	0	-2.660171	-1.100459	-1.074242
29	1	0	-3.348731	-1.106933	-1.916751
30	1	0	-1.641232	-1.067137	-1.460191
31	7	0	-2.867170	0.154633	-0.307908
32	1	0	-3.842104	0.228794	-0.008133
33	7	0	3.656230	-0.358219	-0.456287
34	6	0	3.888021	0.284835	-1.785266
35	1	0	3.186724	-0.140302	-2.497701
36	1	0	3.732224	1.354477	-1.677712
37	1	0	4.913193	0.076610	-2.078231
38	6	0	4.603193	0.168447	0.573465
39	1	0	4.445154	1.239235	0.666320
40	1	0	4.401481	-0.337259	1.513489
41	1	0	5.612586	-0.039729	0.229834
42	1	0	-2.259346	0.094832	0.531134
43	1	0	3.854499	-1.356854	-0.566092

Most stable energy, Gibbs free energy (Ha), and geometry for protomer ./NMe2_PyN3//2_3
E: -783.867261
G: -783.513904
Geometry:
Input orientation:

Center Number	Atomic Number	Atomic Type	Coordinates (Angstroms) X	Y	Z
1	6	0	0.364970	-1.309471	0.526309
2	6	0	1.639327	-1.269397	0.042562
3	6	0	2.347139	-0.040331	0.007731
4	6	0	1.635595	1.128727	0.414897
5	6	0	0.366681	1.025798	0.887914
6	7	0	-0.229076	-0.186702	0.973562
7	1	0	2.080431	-2.187118	-0.315260
8	1	0	2.077484	2.109975	0.341162
9	6	0	-0.415926	2.229321	1.317806
10	1	0	-0.563689	2.229712	2.397307
11	1	0	0.109906	3.133105	1.023119
12	6	0	-0.451167	-2.571823	0.623003
13	1	0	-0.101330	-3.113202	1.503940
14	1	0	-0.218688	-3.189474	-0.251334
15	7	0	-1.769049	2.258019	0.692409
16	1	0	-2.333685	1.462377	1.023896
17	7	0	-1.871777	-2.303150	0.768659
18	1	0	-2.277265	-3.036113	1.334446
19	6	0	-1.813667	2.180403	-0.801867
20	1	0	-0.937490	1.626162	-1.134248
21	1	0	-1.765736	3.190052	-1.199615
22	6	0	-3.096412	1.462258	-1.184479
23	1	0	-3.950748	2.065379	-0.874884
24	1	0	-3.127275	1.369949	-2.274897
25	6	0	-2.599669	-2.232841	-0.502363
26	1	0	-2.264346	-3.020567	-1.187448
27	1	0	-3.654610	-2.415004	-0.288502
28	6	0	-2.480164	-0.901235	-1.223234
29	1	0	-2.902210	-1.025946	-2.228121
30	1	0	-1.430212	-0.630140	-1.363181

31	7	0	-3.162542	0.173453	-0.500740
32	1	0	-4.134868	-0.090836	-0.387527
33	7	0	3.605793	0.032132	-0.414526
34	6	0	4.294024	1.317349	-0.473899
35	1	0	3.798950	1.994012	-1.172161
36	1	0	4.323303	1.785514	0.510738
37	1	0	5.311485	1.152017	-0.810844
38	6	0	4.299124	-1.173213	-0.853942
39	1	0	5.320557	-0.914621	-1.110700
40	1	0	4.316304	-1.918887	-0.057949
41	1	0	3.813561	-1.605675	-1.730521
42	1	0	-2.247530	3.106105	1.006479
43	1	0	-1.192165	-0.314491	1.289434

Most stable energy, Gibbs free energy (Ha), and geometry for protomer ./NMe2_PyN3//2_6

E: -783.873984

G: -783.520066

Geometry:

Input orientation:

Center	Atomic	Atomic	Coordinates (Angstroms)		
Number	Number	Type	X	Y	Z
1	6	0	0.423040	-1.087715	0.920385
2	6	0	1.686970	-1.139740	0.382108
3	6	0	2.344124	0.067877	0.048366
4	6	0	1.600472	1.255596	0.233075
5	6	0	0.342372	1.179808	0.786010
6	7	0	-0.246416	0.043208	1.158568
7	1	0	2.143392	-2.101605	0.203129
8	1	0	1.990688	2.219663	-0.056980
9	6	0	-0.450698	2.432878	1.042822
10	1	0	-0.308848	2.749449	2.076091
11	1	0	-0.158584	3.245012	0.382545
12	6	0	-0.329928	-2.348046	1.256003
13	1	0	-0.356472	-2.512179	2.332408
14	1	0	0.095090	-3.220239	0.765985
15	7	0	-1.911170	2.186356	0.869384
16	1	0	-2.123793	1.261909	1.270543
17	7	0	-1.736548	-2.187106	0.796394
18	1	0	-2.078948	-1.276120	1.135677
19	6	0	-2.423845	2.215795	-0.541121
20	1	0	-1.845114	2.957700	-1.085729
21	1	0	-3.459138	2.548221	-0.482941
22	6	0	-2.354293	0.859160	-1.206489
23	1	0	-2.681657	0.991526	-2.244278
24	1	0	-1.324904	0.500203	-1.237478
25	6	0	-1.890691	-2.173936	-0.688503
26	1	0	-1.010786	-1.681924	-1.099634
27	1	0	-1.906982	-3.204405	-1.033690
28	6	0	-3.163672	-1.440488	-1.060075
29	1	0	-4.023508	-1.989163	-0.674527
30	1	0	-3.235682	-1.430376	-2.153392
31	7	0	-3.184508	-0.098838	-0.481989
32	1	0	-4.141599	0.238468	-0.491176
33	7	0	3.598746	0.081420	-0.447457
34	6	0	4.155634	1.321967	-0.963166
35	1	0	3.584801	1.697176	-1.817504
36	1	0	4.169134	2.091520	-0.190817

37	1	0	5.178609	1.141022	-1.278780
38	6	0	4.250758	-1.171888	-0.792426
39	1	0	3.723773	-1.695747	-1.595195
40	1	0	5.263842	-0.959798	-1.119761
41	1	0	4.305236	-1.831121	0.074246
42	1	0	-2.326098	-2.918201	1.198102
43	1	0	-2.418934	2.883690	1.414865

Most stable energy, Gibbs free energy (Ha), and geometry for protomer ./NMe2_PyN3//2_10
E: -783.866948
G: -783.512237
Geometry:
Input orientation:

Center Number	Atomic Number	Atomic Type	Coordinates (Angstroms) X	Y	Z
1	6	0	0.351098	-1.236067	0.602817
2	6	0	1.649122	-1.252769	0.139595
3	6	0	2.371151	-0.044522	0.094350
4	6	0	1.662250	1.115558	0.477055
5	6	0	0.359744	1.018747	0.915810
6	7	0	-0.301519	-0.146683	1.005636
7	1	0	2.080560	-2.188817	-0.183187
8	1	0	2.115767	2.093134	0.411544
9	6	0	-0.387811	2.272936	1.301765
10	1	0	-0.447410	2.328487	2.390927
11	1	0	0.189965	3.138210	0.960091
12	6	0	-0.387612	-2.546583	0.700962
13	1	0	-0.070690	-3.082376	1.595077
14	1	0	-0.204551	-3.177528	-0.165723
15	7	0	-1.749116	2.264916	0.778222
16	1	0	-2.249522	3.067293	1.142321
17	7	0	-1.857538	-2.341887	0.824586
18	1	0	-2.002134	-1.527715	1.434510
19	6	0	-1.816089	2.269108	-0.681880
20	1	0	-0.953330	1.731199	-1.078976
21	1	0	-1.799606	3.271808	-1.115492
22	6	0	-3.089729	1.569135	-1.117366
23	1	0	-3.967764	2.121784	-0.789419
24	1	0	-3.138924	1.412378	-2.192242
25	6	0	-2.613860	-2.190200	-0.459488
26	1	0	-2.272007	-2.984224	-1.119327
27	1	0	-3.662736	-2.363662	-0.221810
28	6	0	-2.451642	-0.861875	-1.162254
29	1	0	-2.931465	-0.950454	-2.134974
30	1	0	-1.413327	-0.576608	-1.310570
31	7	0	-3.143497	0.237272	-0.436917
32	1	0	-4.115447	-0.034904	-0.275211
33	7	0	3.661611	0.006398	-0.312887
34	6	0	4.259220	-1.168814	-0.926232
35	1	0	5.298913	-0.953585	-1.154106
36	1	0	4.232697	-2.018188	-0.243748
37	1	0	3.746522	-1.450292	-1.851384
38	6	0	4.277946	1.298416	-0.570324
39	1	0	5.314958	1.140320	-0.850889
40	1	0	3.773083	1.836987	-1.378260
41	1	0	4.261518	1.921055	0.323935
42	1	0	-2.695525	0.409078	0.481070

43	1	0	-2.252387	-3.150869	1.309001

Most stable energy, Gibbs free energy (Ha), and geometry for protomer ./NMe2_PyN3//2_2
E: -783.849810
G: -783.496312
Geometry:
Input orientation:

Center Number	Atomic Number	Atomic Type	Coordinates (Angstroms) X	Y	Z
1	6	0	0.264268	-1.314526	0.658205
2	6	0	1.551296	-1.396006	0.128247
3	6	0	2.275478	-0.231234	0.020308
4	6	0	1.740691	0.987739	0.398611
5	6	0	0.459720	0.960535	0.919638
6	7	0	-0.243672	-0.159349	1.067737
7	1	0	1.958250	-2.344251	-0.201024
8	1	0	2.273991	1.920775	0.275331
9	6	0	-0.253011	2.222095	1.329147
10	1	0	-0.237644	2.340206	2.411910
11	1	0	0.182379	3.102569	0.863568
12	6	0	-0.586272	-2.554087	0.825620
13	1	0	-0.314683	-2.983228	1.795079
14	1	0	-0.293119	-3.285995	0.072373
15	7	0	-1.673916	2.113050	0.907110
16	1	0	-2.029255	1.187523	1.195580
17	7	0	-2.027250	-2.375838	0.779832
18	1	0	-2.273118	-1.567097	1.342744
19	6	0	-1.877067	2.188377	-0.570607
20	1	0	-1.032847	1.682916	-1.036690
21	1	0	-1.861808	3.235761	-0.860685
22	6	0	-3.190535	1.519625	-0.929002
23	1	0	-4.014254	2.092074	-0.500333
24	1	0	-3.292877	1.552795	-2.020269
25	6	0	-2.588033	-2.195318	-0.560398
26	1	0	-2.112565	-2.916946	-1.226443
27	1	0	-3.649523	-2.457089	-0.519853
28	6	0	-2.483807	-0.808493	-1.179083
29	1	0	-2.857581	-0.861945	-2.210960
30	1	0	-1.441795	-0.490934	-1.235978
31	7	0	-3.242377	0.164589	-0.392623
32	1	0	-4.210680	-0.136563	-0.367106
33	7	0	3.622213	-0.285395	-0.557287
34	6	0	3.679817	0.397078	-1.887106
35	1	0	3.492410	1.456072	-1.731552
36	1	0	4.674523	0.239622	-2.294670
37	1	0	2.922414	-0.042606	-2.529901
38	6	0	4.664188	0.250632	0.370909
39	1	0	4.579747	-0.271386	1.319776
40	1	0	5.632804	0.069555	-0.086801
41	1	0	4.497053	1.316530	0.496505
42	1	0	-2.235182	2.830315	1.369984
43	1	0	3.838540	-1.274244	-0.714316

Most stable energy, Gibbs free energy (Ha), and geometry for protomer ./NMe2_PyN3//2_5
E: -783.867261
G: -783.513915
Geometry:
Input orientation:

Center Number	Atomic Number	Atomic Type	Coordinates (Angstroms) X	Y	Z
1	6	0	0.366931	-1.025363	-0.888090
2	6	0	1.635996	-1.127983	-0.415415
3	6	0	2.347206	0.041206	-0.008036
4	6	0	1.638912	1.270014	-0.042239
5	6	0	0.364421	1.309778	-0.525653
6	7	0	-0.229305	0.186942	-0.973169
7	1	0	2.078278	-2.109088	-0.342137
8	1	0	2.079754	2.187772	0.315812
9	6	0	-0.452220	2.571851	-0.621692
10	1	0	-0.219664	3.189339	0.252737
11	1	0	-0.102902	3.113612	-1.502601
12	6	0	-0.415354	-2.229008	-1.318225
13	1	0	0.110818	-3.132727	-1.023945
14	1	0	-0.563323	-2.229086	-2.397699
15	7	0	-1.872784	2.302694	-0.766933
16	1	0	-2.278746	3.035739	-1.332276
17	7	0	-1.768344	-2.258322	-0.692571
18	1	0	-2.246699	-3.106367	-1.006943
19	6	0	-2.600191	2.231623	0.504326
20	1	0	-3.655267	2.413552	0.290921
21	1	0	-2.264868	3.019165	1.189622
22	6	0	-2.480032	0.899750	1.224596
23	1	0	-1.429952	0.628922	1.364093
24	1	0	-2.901790	1.023906	2.229673
25	6	0	-1.812625	-2.181419	0.801749
26	1	0	-1.764205	-3.191235	1.199012
27	1	0	-0.936576	-1.626991	1.134156
28	6	0	-3.095533	-1.463924	1.185023
29	1	0	-3.126133	-1.372110	2.275491
30	1	0	-3.949732	-2.067219	0.875390
31	7	0	-3.162312	-0.174844	0.501868
32	1	0	-4.134761	0.089182	0.389094
33	7	0	3.606009	-0.030924	0.413832
34	6	0	4.294723	-1.315909	0.472617
35	1	0	5.312185	-1.150325	0.809432
36	1	0	3.800024	-1.992997	1.170733
37	1	0	4.323985	-1.783714	-0.512192
38	6	0	4.299034	1.174532	0.853428
39	1	0	3.813692	1.606438	1.730404
40	1	0	5.320694	0.916261	1.109608
41	1	0	4.315539	1.920531	0.057727
42	1	0	-2.333231	-1.462642	-1.023547
43	1	0	-1.192523	0.314463	-1.288750

Most stable energy, Gibbs free energy (Ha), and geometry for protomer ./NMe2_PyN3//2_8
E: -783.877577
G: -783.523801
Geometry:
Input orientation:

Center Number	Atomic Number	Atomic Type	Coordinates (Angstroms) X	Y	Z
1	6	0	0.407596	-1.222207	-0.707545
2	6	0	1.662596	-1.227161	-0.177802

3	6	0	2.368600	-0.002365	-0.010662
4	6	0	1.668925	1.200986	-0.316887
5	6	0	0.412307	1.143575	-0.836583
6	7	0	-0.159497	-0.055747	-1.069481
7	1	0	2.090593	-2.171839	0.120074
8	1	0	2.099237	2.170431	-0.118902
9	6	0	-0.440913	2.342588	-1.148346
10	1	0	-0.001563	3.213830	-0.653166
11	1	0	-0.417316	2.520839	-2.224901
12	6	0	-0.417725	-2.459314	-0.936059
13	1	0	-0.041618	-3.245871	-0.274679
14	1	0	-0.247183	-2.783043	-1.964823
15	7	0	-1.820980	2.080849	-0.751921
16	1	0	-2.426837	2.765681	-1.189370
17	7	0	-1.845394	-2.211948	-0.769517
18	1	0	-2.349547	-2.892810	-1.320893
19	6	0	-2.000064	2.126410	0.699065
20	1	0	-2.054234	3.148974	1.084265
21	1	0	-1.133448	1.661127	1.172012
22	6	0	-3.267310	1.409923	1.115177
23	1	0	-3.371205	1.387303	2.197853
24	1	0	-4.143149	1.888954	0.680793
25	6	0	-2.316836	-2.278040	0.615119
26	1	0	-3.335141	-2.668949	0.611932
27	1	0	-1.706693	-2.956354	1.220156
28	6	0	-2.318058	-0.926501	1.305070
29	1	0	-1.335201	-0.460531	1.289301
30	1	0	-2.640657	-1.021724	2.339858
31	7	0	-3.278460	-0.004350	0.631452
32	1	0	-3.079140	0.001996	-0.374849
33	7	0	3.616591	0.020822	0.453247
34	6	0	4.303645	1.290669	0.656988
35	1	0	4.320171	1.871656	-0.265573
36	1	0	3.817304	1.880921	1.436024
37	1	0	5.326220	1.089429	0.957285
38	6	0	4.285073	-1.220120	0.826522
39	1	0	5.295368	-0.989507	1.147002
40	1	0	3.761338	-1.716339	1.645490
41	1	0	4.334849	-1.902658	-0.022873
42	1	0	-4.220919	-0.386922	0.739176
43	1	0	-1.109655	-0.076672	-1.428304

Most stable energy, Gibbs free energy (Ha), and geometry for protomer ./NMe2_PyN3//2_4
E: -783.849810
G: -783.496360
Geometry:

Input orientation:

Center Number	Atomic Number	Atomic Type	Coordinates (Angstroms) X	Y	Z
1	6	0	0.460501	-0.959606	-0.918611
2	6	0	1.742084	-0.986952	-0.399188
3	6	0	2.276784	0.231737	-0.019833
4	6	0	1.551949	1.396291	-0.125248
5	6	0	0.264264	1.314999	-0.653722
6	7	0	-0.243658	0.160167	-1.064177
7	1	0	2.275911	-1.919949	-0.277857
8	1	0	1.958951	2.344342	0.204588

9	6	0	-0.586497	2.554778	-0.818559
10	1	0	-0.295413	3.283908	-0.061783
11	1	0	-0.312345	2.987730	-1.785608
12	6	0	-0.251804	-2.220915	-1.329686
13	1	0	0.183473	-3.101799	-0.864790
14	1	0	-0.235586	-2.337821	-2.412583
15	7	0	-2.027455	2.376039	-0.777001
16	1	0	-2.271834	1.568269	-1.341888
17	7	0	-1.672957	-2.112686	-0.908568
18	1	0	-2.233623	-2.829827	-1.372356
19	6	0	-2.592075	2.193794	0.561252
20	1	0	-3.653813	2.454076	0.517741
21	1	0	-2.119636	2.915642	1.229216
22	6	0	-2.487747	0.806705	1.179377
23	1	0	-1.445535	0.490022	1.237735
24	1	0	-2.863439	0.859089	2.210575
25	6	0	-1.877139	-2.189201	0.568892
26	1	0	-1.861142	-3.236774	0.858275
27	1	0	-1.033746	-1.683271	1.035951
28	6	0	-3.191580	-1.522008	0.926568
29	1	0	-3.295030	-1.555835	2.017671
30	1	0	-4.014292	-2.095019	0.496687
31	7	0	-3.244308	-0.166549	0.390947
32	1	0	-4.212988	0.133464	0.365135
33	7	0	3.624213	0.285699	0.556129
34	6	0	3.683391	-0.397627	1.885379
35	1	0	3.495363	-1.456455	1.729370
36	1	0	4.678685	-0.240811	2.291773
37	1	0	2.927010	0.041856	2.529523
38	6	0	4.665225	-0.249389	-0.373553
39	1	0	4.498876	-1.315403	-0.499138
40	1	0	4.579108	0.272683	-1.322237
41	1	0	5.634345	-0.067610	0.082801
42	1	0	-2.028479	-1.187038	-1.196455
43	1	0	3.840585	1.274472	0.713594

Most stable energy, Gibbs free energy (Ha), and geometry for protomer ./NMe2_PyN3//2_9
E: -783.866948
G: -783.512240
Geometry:

Input orientation:

Center Number	Atomic Number	Atomic Type	Coordinates (Angstroms) X	Y	Z
1	6	0	0.359719	-1.018726	-0.915677
2	6	0	1.662132	-1.115712	-0.476798
3	6	0	2.371167	0.044280	-0.093941
4	6	0	1.649256	1.252606	-0.139114
5	6	0	0.351273	1.236062	-0.602487
6	7	0	-0.301416	0.146812	-1.005482
7	1	0	2.115547	-2.093336	-0.411252
8	1	0	2.080762	2.188583	0.183785
9	6	0	-0.387219	2.546705	-0.700770
10	1	0	-0.204161	3.177669	0.165890
11	1	0	-0.070043	3.082376	-1.594872
12	6	0	-0.388003	-2.272771	-1.301766
13	1	0	0.189610	-3.138148	-0.960065
14	1	0	-0.447490	-2.328279	-2.390938

15	7	0	-1.857119	2.342199	-0.824691
16	1	0	-2.251780	3.151176	-1.309264
17	7	0	-1.749337	-2.264546	-0.778346
18	1	0	-2.249900	-3.066765	-1.142574
19	6	0	-2.613857	2.190662	0.459167
20	1	0	-3.662632	2.364256	0.221120
21	1	0	-2.272106	2.984668	1.119072
22	6	0	-2.452008	0.862356	1.162051
23	1	0	-1.413745	0.577066	1.310727
24	1	0	-2.932151	0.951074	2.134601
25	6	0	-1.816450	-2.268757	0.681714
26	1	0	-1.800055	-3.271459	1.115334
27	1	0	-0.953691	-1.730887	1.078897
28	6	0	-3.090042	-1.568639	1.117178
29	1	0	-3.139074	-1.411768	2.192042
30	1	0	-3.968182	-2.121191	0.789381
31	7	0	-3.143639	-0.236815	0.436594
32	1	0	-2.695385	-0.408777	-0.481256
33	7	0	3.661519	-0.006911	0.313398
34	6	0	4.259466	1.168217	0.926536
35	1	0	3.746636	1.450171	1.851466
36	1	0	5.299001	0.952614	1.154751
37	1	0	4.233448	2.017432	0.243814
38	6	0	4.277775	-1.299064	0.570298
39	1	0	5.314820	-1.141149	0.850828
40	1	0	3.772942	-1.837942	1.378048
41	1	0	4.261220	-1.921362	-0.324206
42	1	0	-4.115537	0.035350	0.274544
43	1	0	-2.001685	1.528021	-1.434621

Most stable energy, Gibbs free energy (Ha), and geometry for protomer ./NMe2_PyN3//3_7
E: -784.315827
G: -783.945773
Geometry:

Input orientation:

Center Number	Atomic Number	Atomic Type	Coordinates (Angstroms) X	Y	Z
1	6	0	0.326857	-1.106635	-0.928171
2	6	0	1.558671	-1.231308	-0.362016
3	6	0	2.317247	-0.076610	-0.022219
4	6	0	1.687596	1.188531	-0.223138
5	6	0	0.457318	1.254173	-0.794975
6	7	0	-0.178410	0.122296	-1.181190
7	1	0	1.927703	-2.225213	-0.162241
8	1	0	2.166377	2.109950	0.070315
9	6	0	-0.246765	2.561482	-0.997498
10	1	0	0.483903	3.364933	-1.023445
11	1	0	-0.829452	2.567033	-1.916572
12	6	0	-0.555744	-2.276382	-1.264269
13	1	0	-0.075858	-3.181847	-0.882079
14	1	0	-0.628691	-2.363285	-2.349806
15	7	0	-1.196893	2.891745	0.121693
16	1	0	-0.700105	2.836639	1.019011
17	7	0	-1.896340	-2.061305	-0.727645
18	1	0	-2.528972	-2.734725	-1.145676
19	6	0	-2.462761	2.092559	0.176981
20	1	0	-2.698116	1.807928	-0.849039

21	1	0	-3.244901	2.754459	0.538283
22	6	0	-2.333813	0.872925	1.079685
23	1	0	-1.350770	0.413790	1.017136
24	1	0	-2.528927	1.120727	2.120138
25	6	0	-1.947909	-2.175369	0.729700
26	1	0	-1.958648	-3.212546	1.075849
27	1	0	-1.050357	-1.716239	1.146605
28	6	0	-3.188055	-1.489693	1.274595
29	1	0	-3.141470	-1.367217	2.354027
30	1	0	-4.089163	-2.039454	1.009293
31	7	0	-3.339581	-0.136202	0.655073
32	1	0	-3.249066	-0.281549	-0.359265
33	7	0	3.539096	-0.167841	0.485133
34	6	0	4.265493	1.033788	0.884393
35	1	0	3.729393	1.567166	1.670534
36	1	0	5.239344	0.741752	1.261030
37	1	0	4.403617	1.701772	0.033628
38	6	0	4.156076	-1.475308	0.685769
39	1	0	4.177297	-2.035203	-0.249483
40	1	0	5.174559	-1.330686	1.028870
41	1	0	3.609487	-2.053759	1.432407
42	1	0	-4.280487	0.221535	0.837991
43	1	0	-1.454254	3.875801	0.000789
44	1	0	-1.102528	0.179499	-1.603039

Most stable energy, Gibbs free energy (Ha), and geometry for protomer ./NMe2_PyN3//3_5

E: -784.299960

G: -783.932343

Geometry:

Input orientation:

Center Number	Atomic Number	Atomic Type	Coordinates (Angstroms) X	Y	Z
1	6	0	0.423512	-1.248136	-0.654689
2	6	0	1.731299	-1.381817	-0.241452
3	6	0	2.435517	-0.227360	0.055435
4	6	0	1.871156	1.032396	-0.022849
5	6	0	0.558319	1.117213	-0.441539
6	7	0	-0.073207	-0.011368	-0.762968
7	1	0	2.175348	-2.362830	-0.136102
8	1	0	2.412781	1.931366	0.238579
9	6	0	-0.226413	2.396815	-0.560466
10	1	0	-0.032893	2.984297	0.342092
11	1	0	0.199597	2.952979	-1.397675
12	6	0	-0.514153	-2.378526	-0.976753
13	1	0	-0.223391	-3.241587	-0.369672
14	1	0	-0.370006	-2.648620	-2.024737
15	7	0	-1.645943	2.172778	-0.790648
16	1	0	-1.954314	2.770995	-1.543486
17	7	0	-1.892959	-1.960097	-0.776875
18	1	0	-2.506465	-2.562159	-1.314492
19	6	0	-2.517241	2.368935	0.371836
20	1	0	-3.475515	2.751313	0.018287
21	1	0	-2.105709	3.107537	1.065422
22	6	0	-2.752784	1.089177	1.150153
23	1	0	-1.813362	0.605591	1.411302
24	1	0	-3.309253	1.283061	2.064403
25	6	0	-2.286863	-1.988487	0.632389

26	1	0	-2.398594	-3.008278	1.013173
27	1	0	-1.498485	-1.519699	1.224619
28	6	0	-3.605877	-1.276614	0.845932
29	1	0	-3.863614	-1.239158	1.902130
30	1	0	-4.404764	-1.774716	0.299153
31	7	0	-3.567203	0.130970	0.342085
32	1	0	-3.216316	0.115415	-0.621593
33	7	0	3.828232	-0.359799	0.482949
34	6	0	4.764988	0.199981	-0.544516
35	1	0	4.593000	1.270836	-0.610717
36	1	0	5.776451	-0.007502	-0.207344
37	1	0	4.561901	-0.289269	-1.492929
38	6	0	4.077841	0.239702	1.832242
39	1	0	3.976111	1.318056	1.755496
40	1	0	3.356354	-0.175190	2.530278
41	1	0	5.091800	-0.025867	2.117130
42	1	0	-4.523183	0.491626	0.292696
43	1	0	-1.046867	0.083496	-1.067990
44	1	0	4.028528	-1.361828	0.560191

Most stable energy, Gibbs free energy (Ha), and geometry for protomer ./NMe2_PyN3//3_9
E: -784.310361
G: -783.942380
Geometry:
Input orientation:

Center Number	Atomic Number	Atomic Type	Coordinates (Angstroms) X	Y	Z
1	6	0	0.477812	-1.086009	-1.032617
2	6	0	1.703797	-1.051637	-0.450858
3	6	0	2.253653	0.200038	-0.030630
4	6	0	1.427488	1.350692	-0.167021
5	6	0	0.212784	1.250992	-0.777529
6	7	0	-0.205621	0.059532	-1.241896
7	1	0	2.223321	-1.982942	-0.282981
8	1	0	1.732202	2.312471	0.215860
9	6	0	-0.750600	2.389098	-0.988697
10	1	0	-0.555592	3.152554	-0.229916
11	1	0	-0.527355	2.829259	-1.962450
12	6	0	-0.270231	-2.345243	-1.339879
13	1	0	0.382555	-3.116957	-1.736995
14	1	0	-1.083447	-2.159322	-2.037174
15	7	0	-2.136477	1.927426	-0.989255
16	1	0	-2.652385	2.468731	-1.670457
17	7	0	-0.875338	-2.905070	-0.076035
18	1	0	-0.134765	-3.392707	0.438302
19	6	0	-2.810599	2.073223	0.301890
20	1	0	-3.887858	2.044344	0.129252
21	1	0	-2.584629	3.048043	0.748596
22	6	0	-2.447378	1.044262	1.359405
23	1	0	-1.386785	0.798082	1.363701
24	1	0	-2.698071	1.449146	2.336715
25	6	0	-1.496690	-1.938583	0.877533
26	1	0	-1.746775	-2.510894	1.769312
27	1	0	-0.730230	-1.214111	1.144759
28	6	0	-2.751109	-1.290801	0.316325
29	1	0	-3.547610	-2.027358	0.227837
30	1	0	-2.619600	-0.813491	-0.648389

31	7	0	-3.227999	-0.243216	1.265629
32	1	0	-4.195616	-0.013543	1.018668
33	7	0	3.466790	0.271762	0.504514
34	6	0	3.995396	1.554248	0.956552
35	1	0	5.020075	1.412657	1.282578
36	1	0	3.984378	2.280265	0.143124
37	1	0	3.409701	1.947385	1.789451
38	6	0	4.259988	-0.936894	0.704424
39	1	0	4.437216	-1.443819	-0.244677
40	1	0	5.215192	-0.657582	1.134990
41	1	0	3.755707	-1.625240	1.384430
42	1	0	-3.269036	-0.654337	2.203735
43	1	0	-1.563501	-3.617219	-0.344088
44	1	0	-1.118774	0.046113	-1.688994

Most stable energy, Gibbs free energy (Ha), and geometry for protomer ./NMe2_PyN3//3_6
E: -784.304106
G: -783.935177
Geometry:
Input orientation:

Center Number	Atomic Number	Atomic Type	Coordinates (Angstroms) X	Y	Z
1	6	0	0.333693	-1.173751	-0.580213
2	6	0	1.686227	-1.348277	-0.308814
3	6	0	2.435511	-0.234776	-0.005728
4	6	0	1.860456	1.017628	0.044188
5	6	0	0.502273	1.087359	-0.224339
6	7	0	-0.239770	0.030013	-0.540506
7	1	0	2.130737	-2.335530	-0.331024
8	1	0	2.424643	1.909154	0.285926
9	6	0	-0.170500	2.430250	-0.122597
10	1	0	-0.256281	2.731131	0.921566
11	1	0	0.416581	3.180995	-0.645770
12	6	0	-0.493430	-2.384905	-0.936096
13	1	0	-0.097439	-3.235143	-0.368091
14	1	0	-0.326943	-2.605839	-1.992975
15	7	0	-1.540872	2.431349	-0.714161
16	1	0	-1.657694	3.295749	-1.248048
17	7	0	-1.913550	-2.179660	-0.726818
18	1	0	-2.423099	-2.935482	-1.169850
19	6	0	-2.673259	2.373030	0.265289
20	1	0	-3.581001	2.597350	-0.293302
21	1	0	-2.497735	3.169483	0.983790
22	6	0	-2.809708	1.063038	1.002925
23	1	0	-1.869830	0.705697	1.418669
24	1	0	-3.513621	1.215410	1.818149
25	6	0	-2.283617	-2.112909	0.685998
26	1	0	-2.423743	-3.096058	1.142012
27	1	0	-1.485876	-1.611651	1.237126
28	6	0	-3.569207	-1.322550	0.818187
29	1	0	-3.846905	-1.139535	1.853407
30	1	0	-4.386731	-1.824969	0.305647
31	7	0	-3.374856	-0.005189	0.135514
32	1	0	-2.730818	-0.214832	-0.646450
33	7	0	3.864162	-0.397210	0.279744
34	6	0	4.719461	0.316280	-0.718358
35	1	0	4.446624	-0.028985	-1.711461

36	1	0	4.544641	1.383506	-0.614966
37	1	0	5.754829	0.075381	-0.493859
38	6	0	4.207963	0.010471	1.677407
39	1	0	3.568289	-0.539281	2.361985
40	1	0	5.253730	-0.234330	1.840735
41	1	0	4.047695	1.081131	1.769413
42	1	0	-4.258769	0.322860	-0.259774
43	1	0	-1.609920	1.662334	-1.390137
44	1	0	4.073955	-1.396848	0.196814

Most stable energy, Gibbs free energy (Ha), and geometry for protomer ./NMe2_PyN3//3_10
E: -784.313697
G: -783.943813
Geometry:
Input orientation:

Center Number	Atomic Number	Atomic Type	Coordinates (Angstroms) X	Y	Z
1	6	0	0.351639	-1.089646	0.737585
2	6	0	1.662942	-1.132964	0.329257
3	6	0	2.341858	0.076384	0.045599
4	6	0	1.595260	1.267911	0.216387
5	6	0	0.287677	1.196367	0.635282
6	7	0	-0.339873	0.044724	0.901763
7	1	0	2.154172	-2.091033	0.241767
8	1	0	2.036158	2.237309	0.037708
9	6	0	-0.508446	2.455801	0.841413
10	1	0	-1.124400	2.388830	1.735395
11	1	0	0.158716	3.308430	0.927231
12	6	0	-0.402987	-2.356712	1.025406
13	1	0	-1.199094	-2.181398	1.745439
14	1	0	0.255306	-3.133545	1.405379
15	7	0	-1.433730	2.765071	-0.309732
16	1	0	-1.467634	3.783715	-0.394900
17	7	0	-1.033009	-2.918647	-0.214295
18	1	0	-1.567605	-3.750114	0.055751
19	6	0	-2.860912	2.303163	-0.227247
20	1	0	-3.417748	2.983935	-0.865951
21	1	0	-3.192074	2.445783	0.799923
22	6	0	-3.113792	0.898473	-0.731350
23	1	0	-4.136109	0.845632	-1.099263
24	1	0	-2.444063	0.654395	-1.555581
25	6	0	-1.924773	-2.014774	-1.006859
26	1	0	-1.304692	-1.205419	-1.387116
27	1	0	-2.254371	-2.608590	-1.855033
28	6	0	-3.142064	-1.529485	-0.233856
29	1	0	-3.375401	-2.192551	0.598070
30	1	0	-4.001628	-1.507894	-0.899653
31	7	0	-2.981567	-0.151583	0.314546
32	1	0	-3.696271	0.004192	1.028798
33	7	0	3.622148	0.093499	-0.354328
34	6	0	4.289768	1.360446	-0.610336
35	1	0	4.324289	1.978503	0.289617
36	1	0	5.306272	1.161285	-0.933970
37	1	0	3.779855	1.920819	-1.396342
38	6	0	4.361708	-1.152836	-0.481121
39	1	0	3.893562	-1.814997	-1.212255
40	1	0	5.370186	-0.929589	-0.813444

41	1	0	4.418123	-1.677485	0.475207
42	1	0	-2.020060	-0.061903	0.766096
43	1	0	-0.286835	-3.238867	-0.839197
44	1	0	-1.016217	2.424090	-1.182956

Most stable energy, Gibbs free energy (Ha), and geometry for protomer ./NMe2_PyN3//3_8
E: -784.304108
G: -783.935113
Geometry:

Input orientation:

Center Number	Atomic Number	Atomic Type	Coordinates (Angstroms) X	Y	Z
1	6	0	0.503505	-1.086878	-0.220055
2	6	0	1.861833	-1.016742	0.047442
3	6	0	2.436549	0.235832	-0.002990
4	6	0	1.686883	1.349012	-0.306046
5	6	0	0.334275	1.174008	-0.577088
6	7	0	-0.238946	-0.029817	-0.536514
7	1	0	2.426463	-1.908099	0.288738
8	1	0	2.131105	2.336392	-0.328727
9	6	0	-0.492655	2.385148	-0.933652
10	1	0	-0.099116	3.234643	-0.362783
11	1	0	-0.322478	2.608080	-1.989506
12	6	0	-0.168804	-2.429821	-0.116225
13	1	0	-0.256576	-2.727963	0.928553
14	1	0	0.419909	-3.181627	-0.636028
15	7	0	-1.913378	2.178950	-0.729789
16	1	0	-2.421682	2.934356	-1.174934
17	7	0	-1.537921	-2.433932	-0.710608
18	1	0	-1.652429	-3.299935	-1.242421
19	6	0	-2.288881	2.112431	0.681616
20	1	0	-2.431758	3.095674	1.136574
21	1	0	-1.492768	1.612285	1.236060
22	6	0	-3.574255	1.320981	0.809108
23	1	0	-3.856132	1.138823	1.843361
24	1	0	-4.390056	1.822127	0.292588
25	6	0	-2.672536	-2.374450	0.266095
26	1	0	-3.578772	-2.601159	-0.293991
27	1	0	-2.497814	-3.168869	0.987041
28	6	0	-2.811998	-1.062793	1.000022
29	1	0	-1.873461	-0.703809	1.417323
30	1	0	-3.517933	-1.213767	1.813763
31	7	0	-3.375683	0.003090	0.128718
32	1	0	-2.729137	0.212527	-0.651201
33	7	0	3.865366	0.398558	0.281566
34	6	0	4.210604	-0.010437	1.678470
35	1	0	4.051143	-1.081292	1.769454
36	1	0	5.256345	0.234852	1.841187
37	1	0	3.571146	0.538099	2.364217
38	6	0	4.719973	-0.313631	-0.718065
39	1	0	5.755516	-0.072998	-0.494097
40	1	0	4.545214	-1.380982	-0.615842
41	1	0	4.446372	0.032881	-1.710530
42	1	0	-4.257723	-0.326931	-0.269110
43	1	0	-1.606504	-1.667065	-1.389011
44	1	0	4.074710	1.398357	0.199432

Most stable energy, Gibbs free energy (Ha), and geometry for protomer ./NMe2_PyN3//3_3

E: -784.309194
G: -783.940125
Geometry:
Input orientation:

Center Number	Atomic Number	Atomic Type	Coordinates (Angstroms) X	Y	Z
1	6	0	0.585777	-1.033372	-0.575353
2	6	0	1.917596	-1.018771	-0.195946
3	6	0	2.493215	0.221981	0.001824
4	6	0	1.768660	1.386376	-0.145127
5	6	0	0.440736	1.256739	-0.525000
6	7	0	-0.118961	0.077670	-0.753677
7	1	0	2.463337	-1.941963	-0.051736
8	1	0	2.209283	2.357535	0.043245
9	6	0	-0.442312	2.462030	-0.701365
10	1	0	-0.238260	3.216821	0.054857
11	1	0	-0.291124	2.899827	-1.687885
12	6	0	-0.143679	-2.328497	-0.809388
13	1	0	0.155748	-3.087209	-0.089794
14	1	0	0.052881	-2.697332	-1.815818
15	7	0	-1.867669	2.062334	-0.595599
16	1	0	-2.458267	2.808173	-0.970756
17	7	0	-1.606588	-2.111965	-0.683443
18	1	0	-2.103797	-2.912135	-1.081635
19	6	0	-2.320071	1.732891	0.788677
20	1	0	-2.356489	2.664252	1.349375
21	1	0	-1.570665	1.080656	1.231753
22	6	0	-3.684523	1.075381	0.725984
23	1	0	-4.018493	0.902477	1.753764
24	1	0	-4.384506	1.776459	0.268510
25	6	0	-2.079463	-1.885430	0.714380
26	1	0	-1.994640	-2.832237	1.243481
27	1	0	-1.407471	-1.162160	1.170743
28	6	0	-3.514972	-1.397263	0.690556
29	1	0	-3.846046	-1.293193	1.728315
30	1	0	-4.132621	-2.167912	0.226616
31	7	0	-3.688691	-0.155690	-0.065240
32	1	0	-4.567302	-0.208756	-0.561426
33	7	0	3.897025	0.310480	0.411812
34	6	0	4.131159	-0.329905	1.743418
35	1	0	5.165399	-0.144271	2.018839
36	1	0	3.450723	0.118342	2.461907
37	1	0	3.946997	-1.395919	1.645049
38	6	0	4.820320	-0.241600	-0.627693
39	1	0	4.642353	-1.309987	-0.710515
40	1	0	4.616071	0.262305	-1.568060
41	1	0	5.836710	-0.048607	-0.296335
42	1	0	-1.867668	-1.278531	-1.226047
43	1	0	-2.023371	1.222126	-1.168113
44	1	0	4.119922	1.305114	0.513775

Most stable energy, Gibbs free energy (Ha), and geometry for protomer ./NMe2_PyN3//3_4
E: -784.310637
G: -783.941991
Geometry:
Input orientation:

Center Number	Atomic Number	Atomic Type	Coordinates (Angstroms) X	Y	Z
1	6	0	0.382131	-1.126161	0.631057
2	6	0	1.705626	-1.145845	0.311005
3	6	0	2.390533	0.078114	0.060212
4	6	0	1.635621	1.278725	0.199510
5	6	0	0.314263	1.218492	0.527854
6	7	0	-0.290812	0.035379	0.738330
7	1	0	2.211500	-2.097953	0.260651
8	1	0	2.088371	2.247854	0.057527
9	6	0	-0.518536	2.450650	0.726786
10	1	0	-1.100755	2.381277	1.643475
11	1	0	0.137393	3.313524	0.787802
12	6	0	-0.403810	-2.369926	0.922738
13	1	0	-1.165139	-2.181565	1.676412
14	1	0	0.257927	-3.154598	1.278681
15	7	0	-1.482385	2.717708	-0.398069
16	1	0	-1.495178	3.731382	-0.533910
17	7	0	-1.091603	-2.912076	-0.288381
18	1	0	-1.602963	-3.750170	0.005177
19	6	0	-2.918123	2.291091	-0.227894
20	1	0	-3.486356	2.966278	-0.862556
21	1	0	-3.186646	2.480114	0.810024
22	6	0	-3.217358	0.868958	-0.652151
23	1	0	-4.271627	0.836226	-0.941986
24	1	0	-2.638942	0.628498	-1.547489
25	6	0	-2.045856	-2.007240	-1.011669
26	1	0	-1.452413	-1.205447	-1.446961
27	1	0	-2.437534	-2.611139	-1.825931
28	6	0	-3.184036	-1.495620	-0.134886
29	1	0	-3.340088	-2.169393	0.708494
30	1	0	-4.097556	-1.515654	-0.734884
31	7	0	-2.949486	-0.138284	0.382331
32	1	0	-3.589765	0.024255	1.154546
33	7	0	3.674756	0.100174	-0.279368
34	6	0	4.348988	1.369933	-0.528820
35	1	0	3.859871	1.914616	-1.337505
36	1	0	4.348802	1.992803	0.366965
37	1	0	5.375392	1.168767	-0.815292
38	6	0	4.423585	-1.145584	-0.406663
39	1	0	4.445303	-1.684037	0.541867
40	1	0	3.978326	-1.788422	-1.167451
41	1	0	5.441432	-0.911714	-0.698263
42	1	0	-0.379493	-3.220974	-0.957196
43	1	0	-1.113346	2.324224	-1.271044
44	1	0	-1.360485	-0.001443	0.816555

Most stable energy, Gibbs free energy (Ha), and geometry for protomer ./CF3_PyN3//0_1
E: -986.069598
G: -985.814455
Geometry:
Input orientation:

Center Number	Atomic Number	Atomic Type	Coordinates (Angstroms) X	Y	Z
1	6	0	-0.220506	1.110832	0.942802
2	6	0	1.086258	1.178545	0.466122

3	6	0	1.736173	-0.007574	0.185602
4	6	0	1.078152	-1.215186	0.348496
5	6	0	-0.225914	-1.190346	0.825958
6	7	0	-0.837446	-0.050148	1.152192
7	1	0	1.561140	2.137183	0.307684
8	1	0	1.552086	-2.154893	0.094830
9	6	0	-1.005573	-2.476359	1.007448
10	1	0	-0.503538	-3.268352	0.449817
11	1	0	-0.942778	-2.744890	2.065550
12	6	0	-0.988438	2.378016	1.258614
13	1	0	-0.485918	3.217925	0.776770
14	1	0	-0.911647	2.540284	2.337293
15	7	0	-2.409492	-2.461249	0.626934
16	1	0	-2.883451	-1.710591	1.117385
17	7	0	-2.395680	2.411026	0.893576
18	1	0	-2.870659	1.615689	1.306505
19	6	0	-2.676182	-2.382477	-0.810595
20	1	0	-3.749988	-2.523581	-0.948711
21	1	0	-2.177611	-3.229997	-1.285702
22	6	0	-2.255922	-1.112162	-1.539900
23	1	0	-1.160917	-1.012909	-1.518549
24	1	0	-2.534636	-1.214821	-2.592062
25	6	0	-2.674665	2.488138	-0.541675
26	1	0	-3.749662	2.641405	-0.655080
27	1	0	-2.180555	3.383067	-0.925899
28	6	0	-2.257691	1.305863	-1.407935
29	1	0	-1.162464	1.205688	-1.400743
30	1	0	-2.539231	1.523825	-2.441604
31	7	0	-2.904637	0.069525	-0.982215
32	1	0	-2.767944	0.014323	0.023113
33	6	0	3.155815	-0.011301	-0.308021
34	9	0	4.007396	-0.413747	0.648378
35	9	0	3.322584	-0.846500	-1.341746
36	9	0	3.564628	1.195345	-0.708052

Most stable energy, Gibbs free energy (Ha), and geometry for protomer ./CF3_PyN3//1_3
E: -986.530893
G: -986.261666
Geometry:

Input orientation:

Center Number	Atomic Number	Atomic Type	Coordinates (Angstroms) X	Y	Z
1	6	0	-0.037089	-1.176599	0.673117
2	6	0	1.249924	-1.175262	0.146103
3	6	0	1.889823	0.039635	-0.022255
4	6	0	1.247695	1.221871	0.305216
5	6	0	-0.031739	1.117998	0.827595
6	7	0	-0.640088	-0.045808	1.027734
7	1	0	1.727631	-2.105823	-0.134831
8	1	0	1.706517	2.187923	0.148447
9	6	0	-0.844759	2.335332	1.181759
10	1	0	-0.824049	2.516024	2.255821
11	1	0	-0.494239	3.222259	0.659397
12	6	0	-0.784829	-2.470926	0.906359
13	1	0	-0.476216	-2.831471	1.892471
14	1	0	-0.436988	-3.211754	0.185821
15	7	0	-2.257110	2.081968	0.793628

16	1	0	-2.520050	1.139829	1.124496
17	7	0	-2.236295	-2.412522	0.864912
18	1	0	-2.542122	-1.598926	1.390419
19	6	0	-2.489784	2.078974	-0.681289
20	1	0	-1.613272	1.627061	-1.142779
21	1	0	-2.567544	3.111279	-1.012585
22	6	0	-3.747650	1.288605	-0.987632
23	1	0	-4.610100	1.806821	-0.566171
24	1	0	-3.869364	1.268257	-2.077282
25	6	0	-2.815978	-2.339996	-0.477586
26	1	0	-2.279513	-3.042935	-1.116778
27	1	0	-3.849366	-2.694557	-0.419321
28	6	0	-2.839036	-0.975898	-1.152131
29	1	0	-3.212133	-1.103081	-2.177791
30	1	0	-1.829614	-0.569890	-1.229421
31	7	0	-3.676002	-0.042904	-0.397086
32	1	0	-4.613756	-0.426761	-0.349706
33	6	0	3.283242	0.046564	-0.589841
34	9	0	3.338615	-0.586034	-1.769331
35	9	0	3.753354	1.280219	-0.782142
36	9	0	4.144426	-0.582046	0.222406
37	1	0	-2.874723	2.762801	1.238923

Most stable energy, Gibbs free energy (Ha), and geometry for protomer ./CF3_PyN3//1_2
E: -986.536234
 G: -986.267056
Geometry:
                        Input orientation:

Center Number	Atomic Number	Atomic Type	Coordinates (Angstroms) X	Y	Z
1	6	0	-0.153289	-1.069944	0.724804
2	6	0	1.103438	-1.172601	0.147925
3	6	0	1.746589	-0.003788	-0.229129
4	6	0	1.131896	1.218044	-0.052474
5	6	0	-0.136376	1.230131	0.524121
6	7	0	-0.740792	0.114571	0.919697
7	1	0	1.558944	-2.142109	-0.010902
8	1	0	1.601379	2.142527	-0.360286
9	6	0	-0.843293	2.553790	0.745731
10	1	0	-0.373106	3.014026	1.619458
11	1	0	-0.622972	3.207926	-0.099834
12	6	0	-0.872961	-2.323037	1.174259
13	1	0	-0.550160	-2.528302	2.197474
14	1	0	-0.517801	-3.156073	0.555610
15	7	0	-2.279148	2.525112	0.948051
16	1	0	-2.523480	1.808950	1.622812
17	7	0	-2.325063	-2.220971	1.176747
18	1	0	-2.689478	-2.880769	1.850764
19	6	0	-3.088324	2.409593	-0.258749
20	1	0	-2.889864	3.277559	-0.889465
21	1	0	-4.138293	2.459310	0.034403
22	6	0	-2.882062	1.180619	-1.132648
23	1	0	-3.519666	1.227993	-2.013795
24	1	0	-1.847588	1.082929	-1.460144
25	6	0	-2.955344	-2.488271	-0.113099
26	1	0	-2.543297	-3.374696	-0.609606
27	1	0	-4.016142	-2.667070	0.064934

28	6	0	-2.801294	-1.319457	-1.068541
29	1	0	-3.409437	-1.454236	-1.960163
30	1	0	-1.764261	-1.178122	-1.372357
31	7	0	-3.224482	-0.063559	-0.393767
32	1	0	-2.726311	-0.054703	0.512056
33	6	0	3.107141	-0.103866	-0.862830
34	9	0	3.975587	-0.741041	-0.065741
35	9	0	3.064239	-0.794711	-2.011023
36	9	0	3.633602	1.089692	-1.143188
37	1	0	-4.229292	-0.091829	-0.202470

Most stable energy, Gibbs free energy (Ha), and geometry for protomer ./CF3_PyN3//1_1
E: -986.529264
G: -986.263038
Geometry:
Input orientation:

Center Number	Atomic Number	Atomic Type	Coordinates (Angstroms) X	Y	Z
1	6	0	0.027404	1.259522	-0.110039
2	6	0	1.394293	1.471305	-0.262679
3	6	0	2.178491	0.401114	-0.647654
4	6	0	1.608960	-0.840976	-0.881189
5	6	0	0.240608	-0.971404	-0.701727
6	7	0	-0.511138	0.062639	-0.321259
7	1	0	1.818069	2.451721	-0.090345
8	1	0	2.205221	-1.688216	-1.191740
9	6	0	-0.476031	-2.275733	-0.947835
10	1	0	-1.163474	-2.123060	-1.786859
11	1	0	0.259854	-3.013064	-1.264832
12	6	0	-0.888858	2.384179	0.315240
13	1	0	-0.412863	3.327434	0.047802
14	1	0	-0.947717	2.370753	1.409305
15	7	0	-1.196813	-2.774890	0.224896
16	1	0	-0.900424	-3.725459	0.390071
17	7	0	-2.220955	2.295403	-0.279165
18	1	0	-2.394071	3.130540	-0.821554
19	6	0	-2.660145	-2.755590	0.171604
20	1	0	-3.030751	-3.699888	0.569620
21	1	0	-3.024003	-2.679561	-0.859188
22	6	0	-3.292722	-1.664977	1.015222
23	1	0	-4.355221	-1.852565	1.161823
24	1	0	-2.807377	-1.615480	1.988671
25	6	0	-3.317343	2.135851	0.671186
26	1	0	-3.318004	2.901858	1.456219
27	1	0	-4.251518	2.230935	0.116062
28	6	0	-3.287551	0.792063	1.367695
29	1	0	-4.194261	0.644752	1.952282
30	1	0	-2.429094	0.700927	2.034578
31	7	0	-3.165114	-0.319420	0.391498
32	1	0	-3.861027	-0.216135	-0.349623
33	6	0	3.653306	0.597181	-0.871990
34	9	0	4.352520	-0.515022	-0.625989
35	9	0	3.911466	0.943732	-2.142209
36	9	0	4.159076	1.561848	-0.097775
37	1	0	-2.205633	-0.212007	-0.039497

Most stable energy, Gibbs free energy (Ha), and geometry for protomer ./CF3_PyN3//1_4
E: -986.530893

G: -986.261637
Geometry:
Input orientation:

Center Number	Atomic Number	Atomic Type	Coordinates (Angstroms) X	Y	Z
1	6	0	-0.031517	-1.117170	-0.827873
2	6	0	1.247736	-1.221327	-0.305156
3	6	0	1.889530	-0.039289	0.023766
4	6	0	1.249475	1.175639	-0.143600
5	6	0	-0.037347	1.177269	-0.671141
6	7	0	-0.640004	0.046744	-1.027110
7	1	0	1.706677	-2.187449	-0.149178
8	1	0	1.726883	2.106015	0.138443
9	6	0	-0.785079	2.471762	-0.903479
10	1	0	-0.438167	3.211692	-0.181566
11	1	0	-0.475388	2.833679	-1.888754
12	6	0	-0.844196	-2.334296	-1.183502
13	1	0	-0.493787	-3.221632	-0.661756
14	1	0	-0.822962	-2.514043	-2.257713
15	7	0	-2.236587	2.413053	-0.863745
16	1	0	-2.541616	1.599748	-1.390168
17	7	0	-2.256772	-2.081495	-0.795822
18	1	0	-2.874036	-2.762157	-1.241867
19	6	0	-2.817760	2.339426	0.478052
20	1	0	-3.851260	2.693497	0.418818
21	1	0	-2.282382	3.042273	1.118251
22	6	0	-2.840861	0.974931	1.151772
23	1	0	-1.831347	0.569244	1.229587
24	1	0	-3.214769	1.101380	2.177229
25	6	0	-2.490146	-2.079553	0.678982
26	1	0	-2.567695	-3.112100	1.009574
27	1	0	-1.614026	-1.627614	1.141187
28	6	0	-3.748452	-1.289824	0.985188
29	1	0	-3.870778	-1.270246	2.074786
30	1	0	-4.610492	-1.808064	0.562918
31	7	0	-3.676934	0.042093	0.395560
32	1	0	-4.614800	0.425620	0.347772
33	6	0	3.282918	-0.046587	0.591426
34	9	0	4.144568	0.580378	-0.221649
35	9	0	3.338844	0.587389	1.770112
36	9	0	3.752115	-1.280372	0.785173
37	1	0	-2.519794	-1.139185	-1.126133

Most stable energy, Gibbs free energy (Ha), and geometry for protomer ./CF3_PyN3//2_1
E: -986.982760
G: -986.698660
Geometry:
Input orientation:

Center Number	Atomic Number	Atomic Type	Coordinates (Angstroms) X	Y	Z
1	6	0	0.099821	-1.130720	-0.718870
2	6	0	1.418756	-1.153897	-0.326497
3	6	0	2.043445	0.057000	-0.051451
4	6	0	1.372484	1.262401	-0.139446
5	6	0	0.047813	1.241692	-0.538747

6	7	0	-0.498168	0.063218	-0.831396
7	1	0	1.932548	-2.099303	-0.217778
8	1	0	1.843566	2.202972	0.108149
9	6	0	-0.829118	2.456964	-0.669622
10	1	0	-0.623852	3.105291	0.187651
11	1	0	-0.494840	2.992698	-1.560531
12	6	0	-0.747885	-2.336307	-1.015529
13	1	0	-0.366283	-3.171291	-0.419484
14	1	0	-0.615338	-2.593903	-2.068119
15	7	0	-2.239764	2.126390	-0.803097
16	1	0	-2.644254	2.709644	-1.521503
17	7	0	-2.150744	-2.036703	-0.771723
18	1	0	-2.727570	-2.690728	-1.288742
19	6	0	-3.034361	2.245539	0.422080
20	1	0	-4.039849	2.565628	0.146166
21	1	0	-2.624384	2.999938	1.099217
22	6	0	-3.126826	0.939928	1.187108
23	1	0	-2.141425	0.523212	1.388936
24	1	0	-3.647092	1.075899	2.132613
25	6	0	-2.493702	-2.095039	0.649579
26	1	0	-2.524848	-3.120185	1.030472
27	1	0	-1.719218	-1.574279	1.216151
28	6	0	-3.847967	-1.469025	0.904688
29	1	0	-4.077676	-1.446332	1.967771
30	1	0	-4.630140	-2.015116	0.379978
31	7	0	-3.907259	-0.062346	0.401384
32	1	0	-4.884060	0.240966	0.378117
33	6	0	3.498352	0.032600	0.356772
34	9	0	3.889154	1.182461	0.901911
35	9	0	3.740304	-0.936087	1.242081
36	9	0	4.281487	-0.195162	-0.702964
37	1	0	-3.579914	-0.056881	-0.570955
38	1	0	-1.481255	0.072389	-1.119137

Most stable energy, Gibbs free energy (Ha), and geometry for protomer ./CF3_PyN3//2_3
E: -986.987185
G: -986.704362
Geometry:
Input orientation:

Center Number	Atomic Number	Atomic Type	Coordinates (Angstroms) X	Y	Z
1	6	0	-0.105525	0.935354	0.918708
2	6	0	1.186108	1.066674	0.431291
3	6	0	1.828121	-0.061535	-0.053061
4	6	0	1.183549	-1.280822	-0.061935
5	6	0	-0.110465	-1.315801	0.442286
6	7	0	-0.728701	-0.248539	0.929954
7	1	0	1.669456	2.035335	0.420301
8	1	0	1.647655	-2.175101	-0.454049
9	6	0	-0.851373	-2.627754	0.453244
10	1	0	-0.739692	-3.149718	-0.495512
11	1	0	-0.459247	-3.263278	1.246348
12	6	0	-0.820484	2.141297	1.483064
13	1	0	-0.343752	3.039079	1.074190
14	1	0	-0.640350	2.144578	2.560531
15	7	0	-2.304365	-2.443216	0.714965
16	1	0	-2.661969	-3.290430	1.163566

17	7	0	-2.260883	2.140812	1.271591
18	1	0	-2.690285	2.715308	1.984133
19	6	0	-3.161770	-2.198406	-0.491079
20	1	0	-4.191335	-2.164346	-0.139404
21	1	0	-3.036614	-3.064963	-1.135809
22	6	0	-2.808299	-0.956686	-1.273984
23	1	0	-1.749447	-0.904629	-1.518470
24	1	0	-3.367090	-0.973225	-2.206981
25	6	0	-2.686621	2.623462	-0.039896
26	1	0	-3.732510	2.920796	0.036979
27	1	0	-2.111467	3.490872	-0.380900
28	6	0	-2.554349	1.533768	-1.091740
29	1	0	-1.512234	1.300595	-1.306472
30	1	0	-3.054918	1.801158	-2.018947
31	7	0	-3.177178	0.290443	-0.553336
32	1	0	-4.195902	0.393909	-0.541168
33	6	0	3.227104	0.080109	-0.590454
34	9	0	4.072136	0.510643	0.354843
35	9	0	3.712967	-1.068771	-1.061707
36	9	0	3.278668	0.971667	-1.588365
37	1	0	-2.410097	-1.685200	1.399557
38	1	0	-2.864012	0.258638	0.429402

Most stable energy, Gibbs free energy (Ha), and geometry for protomer ./CF3_PyN3//2_5
E: -986.987185
G: -986.703585
Geometry:
Input orientation:

Center Number	Atomic Number	Atomic Type	Coordinates (Angstroms) X	Y	Z
1	6	0	-0.109578	-1.313739	-0.445363
2	6	0	1.186377	-1.283771	0.053512
3	6	0	1.833521	-0.065652	0.046730
4	6	0	1.192204	1.065836	-0.430547
5	6	0	-0.101941	0.939536	-0.912977
6	7	0	-0.727408	-0.242911	-0.926084
7	1	0	1.650107	-2.180640	0.440096
8	1	0	1.677887	2.033318	-0.417835
9	6	0	-0.816303	2.149544	-1.469537
10	1	0	-0.345132	3.044080	-1.047200
11	1	0	-0.626946	2.165211	-2.545318
12	6	0	-0.854758	-2.623235	-0.460224
13	1	0	-0.740363	-3.151037	0.484903
14	1	0	-0.469086	-3.255428	-1.259103
15	7	0	-2.258308	2.143140	-1.269899
16	1	0	-2.683977	2.718634	-1.983861
17	7	0	-2.308324	-2.430023	-0.713691
18	1	0	-2.671074	-3.266911	-1.177031
19	6	0	-2.696051	2.620512	0.039569
20	1	0	-3.743943	2.909198	-0.043054
21	1	0	-2.130170	3.492360	0.384935
22	6	0	-2.560555	1.531469	1.091124
23	1	0	-1.517711	1.303949	1.308467
24	1	0	-3.064873	1.795956	2.017160
25	6	0	-3.159472	-2.200939	0.499574
26	1	0	-4.191553	-2.171899	0.154854
27	1	0	-3.023625	-3.069415	1.139458

28	6	0	-2.807946	-0.958272	1.281113
29	1	0	-1.749114	-0.904972	1.525886
30	1	0	-3.368269	-0.970979	2.213196
31	7	0	-3.175210	0.284321	0.552343
32	1	0	-4.194139	0.384707	0.530713
33	6	0	3.234932	0.070776	0.579240
34	9	0	3.719176	-1.080526	1.046214
35	9	0	4.077748	0.500916	-0.368174
36	9	0	3.292672	0.959996	1.578909
37	1	0	-2.412739	-1.657098	-1.382373
38	1	0	-2.852860	0.246745	-0.427227

Most stable energy, Gibbs free energy (Ha), and geometry for protomer ./CN_PyN3//0_1
E: -741.223375
G: -740.973624
Geometry:
Input orientation:

Center Number	Atomic Number	Atomic Type	Coordinates (Angstroms) X	Y	Z
1	6	0	0.504050	-1.152108	-0.824141
2	6	0	1.762735	-1.202231	-0.237448
3	6	0	2.395844	0.003374	0.037196
4	6	0	1.759399	1.207396	-0.236613
5	6	0	0.500855	1.154212	-0.823371
6	7	0	-0.079085	0.000366	-1.145796
7	1	0	2.225994	-2.147021	0.012230
8	1	0	2.220044	2.153290	0.013707
9	6	0	-0.287041	2.410865	-1.122070
10	1	0	0.141418	3.244763	-0.565263
11	1	0	-0.149864	2.634058	-2.183858
12	6	0	-0.280508	-2.410648	-1.123651
13	1	0	0.150312	-3.243865	-0.567654
14	1	0	-0.143072	-2.632587	-2.185665
15	7	0	-1.717388	2.334578	-0.844601
16	1	0	-2.041893	1.432889	-1.184681
17	7	0	-1.710978	-2.338333	-0.845757
18	1	0	-2.037935	-1.437346	-1.185363
19	6	0	-2.065398	2.416498	0.576157
20	1	0	-3.147498	2.556672	0.643093
21	1	0	-1.604472	3.316237	0.989398
22	6	0	-1.684937	1.222181	1.443914
23	1	0	-0.603830	1.073856	1.421629
24	1	0	-1.941427	1.455497	2.486780
25	6	0	-2.058421	-2.421861	0.575048
26	1	0	-3.140093	-2.565234	0.642182
27	1	0	-1.594770	-3.320426	0.987802
28	6	0	-1.681291	-1.226818	1.443271
29	1	0	-0.600623	-1.075322	1.420925
30	1	0	-1.936971	-1.461351	2.486067
31	7	0	-2.320457	-0.003145	0.971487
32	1	0	-3.292024	-0.004672	1.265139
33	6	0	3.698989	0.004956	0.640882
34	7	0	4.742660	0.006358	1.121185

Most stable energy, Gibbs free energy (Ha), and geometry for protomer ./CN_PyN3//1_3
E: -741.690362
G: -741.424073
Geometry:

Input orientation:

Center	Atomic	Atomic	Coordinates (Angstroms)		
Number	Number	Type	X	Y	Z
1	6	0	0.705740	-1.121296	-0.699857
2	6	0	1.937396	-1.233757	-0.082086
3	6	0	2.561708	-0.049335	0.298617
4	6	0	1.941250	1.172296	0.085693
5	6	0	0.697775	1.175838	-0.540346
6	7	0	0.121675	0.048997	-0.943850
7	1	0	2.386371	-2.197544	0.112827
8	1	0	2.403210	2.095651	0.407886
9	6	0	-0.027998	2.472615	-0.823935
10	1	0	0.277974	3.214722	-0.086031
11	1	0	0.341731	2.827633	-1.790772
12	6	0	-0.087765	-2.332221	-1.113004
13	1	0	0.232431	-3.225431	-0.582001
14	1	0	-0.008910	-2.499660	-2.186503
15	7	0	-1.479257	2.418866	-0.870665
16	1	0	-1.756530	1.609301	-1.417670
17	7	0	-1.518753	-2.079764	-0.798998
18	1	0	-2.110891	-2.761408	-1.276875
19	6	0	-2.139965	2.345364	0.433809
20	1	0	-3.168533	2.697414	0.311419
21	1	0	-1.645612	3.050113	1.104194
22	6	0	-2.201883	0.982072	1.107994
23	1	0	-1.198861	0.575976	1.246040
24	1	0	-2.634586	1.111483	2.109647
25	6	0	-1.830933	-2.076619	0.661359
26	1	0	-1.929386	-3.108584	0.988075
27	1	0	-0.980370	-1.626366	1.170541
28	6	0	-3.101987	-1.282409	0.896174
29	1	0	-3.283896	-1.259043	1.977280
30	1	0	-3.941079	-1.799333	0.428613
31	7	0	-2.993664	0.047508	0.307404
32	1	0	-3.926329	0.432467	0.203260
33	6	0	3.841857	-0.096757	0.948520
34	7	0	4.865785	-0.133966	1.467190
35	1	0	-1.766688	-1.138429	-1.143470

Most stable energy, Gibbs free energy (Ha), and geometry for protomer ./CN_PyN3//1_2
E: -741.695370
G: -741.431055
Geometry:

Input orientation:

Center	Atomic	Atomic	Coordinates (Angstroms)		
Number	Number	Type	X	Y	Z
1	6	0	0.592796	-1.156521	-0.399932
2	6	0	1.795179	-1.205381	0.294036
3	6	0	2.397236	0.000019	0.628100
4	6	0	1.795128	1.205407	0.294079
5	6	0	0.592732	1.156527	-0.399867
6	7	0	0.032739	-0.000003	-0.748896
7	1	0	2.239827	-2.150280	0.575354
8	1	0	2.239744	2.150313	0.575421
9	6	0	-0.094060	2.448350	-0.790992

10	1	0	0.088334	3.179932	0.006108
11	1	0	0.415641	2.823173	-1.681343
12	6	0	-0.093970	-2.448356	-0.791059
13	1	0	0.088471	-3.179954	0.006015
14	1	0	0.415717	-2.823133	-1.681436
15	7	0	-1.509615	2.316975	-1.101364
16	1	0	-1.755394	3.032764	-1.772122
17	7	0	-1.509540	-2.317014	-1.101388
18	1	0	-1.755316	-3.032808	-1.772141
19	6	0	-2.395882	2.453502	0.050803
20	1	0	-3.413691	2.574627	-0.321551
21	1	0	-2.156289	3.331052	0.663685
22	6	0	-2.339570	1.245413	0.963512
23	1	0	-1.358470	1.134278	1.425319
24	1	0	-3.084751	1.318621	1.753057
25	6	0	-2.395775	-2.453574	0.050802
26	1	0	-3.413587	-2.574741	-0.321529
27	1	0	-2.156133	-3.331115	0.663679
28	6	0	-2.339500	-1.245482	0.963508
29	1	0	-1.358397	-1.134304	1.425299
30	1	0	-3.084663	-1.318725	1.753066
31	7	0	-2.594638	-0.000040	0.194380
32	1	0	-3.551574	-0.000066	-0.167089
33	6	0	3.639560	0.000023	1.350296
34	7	0	4.633070	0.000130	1.926584
35	1	0	-1.941109	-0.000019	-0.610042

Most stable energy, Gibbs free energy (Ha), and geometry for protomer ./CN_PyN3//1_1
E: -741.690361
G: -741.424065
Geometry:

Input orientation:

Center Number	Atomic Number	Atomic Type	Coordinates (Angstroms) X	Y	Z
1	6	0	0.697271	-1.176205	-0.540499
2	6	0	1.940959	-1.173281	0.085156
3	6	0	2.562030	0.048037	0.297988
4	6	0	1.938141	1.232780	-0.082456
5	6	0	0.706281	1.120931	-0.699899
6	7	0	0.121587	-0.049092	-0.943776
7	1	0	2.402586	-2.096885	0.407112
8	1	0	2.387614	2.196344	0.112409
9	6	0	-0.086765	2.332238	-1.112790
10	1	0	0.233922	3.225267	-0.581780
11	1	0	-0.008018	2.499726	-2.186291
12	6	0	-0.029134	-2.472660	-0.823913
13	1	0	0.276417	-3.214769	-0.085838
14	1	0	0.340480	-2.828063	-1.790653
15	7	0	-1.517824	2.080408	-0.798555
16	1	0	-2.109713	2.762418	-1.276215
17	7	0	-1.480382	-2.418181	-0.870715
18	1	0	-1.757191	-1.608296	-1.417494
19	6	0	-1.829716	2.077200	0.661862
20	1	0	-1.927590	3.109167	0.988746
21	1	0	-0.979277	1.626449	1.170813
22	6	0	-3.101117	1.283592	0.896815
23	1	0	-3.282851	1.260208	1.977955

24	1	0	-3.940043	1.800991	0.429476
25	6	0	-2.141066	-2.344586	0.433789
26	1	0	-3.169836	-2.696013	0.311311
27	1	0	-1.647119	-3.049770	1.104009
28	6	0	-2.202193	-0.981393	1.108234
29	1	0	-1.198937	-0.575858	1.246259
30	1	0	-2.634850	-1.110752	2.109918
31	7	0	-2.993558	-0.046293	0.307884
32	1	0	-3.926428	-0.430752	0.203733
33	6	0	3.842385	0.094849	0.947522
34	7	0	4.866483	0.131567	1.465890
35	1	0	-1.766263	1.139242	-1.143124

Most stable energy, Gibbs free energy (Ha), and geometry for protomer ./CN_PyN3//1_4
E: -741.695714
G: -741.430370
Geometry:
Input orientation:

Center Number	Atomic Number	Atomic Type	Coordinates (Angstroms) X	Y	Z
1	6	0	0.621544	-1.180176	0.442041
2	6	0	1.842585	-1.181866	-0.221696
3	6	0	2.421599	0.044700	-0.510366
4	6	0	1.782082	1.225179	-0.154023
5	6	0	0.568811	1.129843	0.513434
6	7	0	0.023474	-0.050515	0.814338
7	1	0	2.317045	-2.108802	-0.514244
8	1	0	2.208969	2.188035	-0.398827
9	6	0	-0.146342	2.393929	0.938099
10	1	0	0.241453	2.665361	1.922611
11	1	0	0.146511	3.194892	0.248827
12	6	0	-0.043650	-2.497540	0.792765
13	1	0	0.487962	-2.889737	1.664392
14	1	0	0.137466	-3.203165	-0.019792
15	7	0	-1.591692	2.263479	1.046441
16	1	0	-1.925434	2.949637	1.709889
17	7	0	-1.464461	-2.477448	1.081749
18	1	0	-1.680471	-1.725953	1.727050
19	6	0	-2.314124	2.449706	-0.208900
20	1	0	-1.955771	3.315255	-0.778410
21	1	0	-3.363770	2.619038	0.032687
22	6	0	-2.201182	1.233543	-1.109508
23	1	0	-2.871462	1.309010	-1.962752
24	1	0	-1.184698	1.093313	-1.477178
25	6	0	-2.349199	-2.448382	-0.075962
26	1	0	-2.170148	-3.347312	-0.667851
27	1	0	-3.377652	-2.501150	0.285018
28	6	0	-2.225724	-1.267768	-1.028570
29	1	0	-2.914929	-1.378121	-1.864002
30	1	0	-1.216056	-1.169001	-1.425464
31	7	0	-2.551703	0.009784	-0.340379
32	1	0	-3.543531	0.027851	-0.089286
33	6	0	3.677727	0.095426	-1.206161
34	7	0	4.682475	0.135332	-1.761118
35	1	0	-2.000814	0.063386	0.532468

Most stable energy, Gibbs free energy (Ha), and geometry for protomer ./CN_PyN3//1_5
E: -741.620549

G: -741.358062
Geometry:

Input orientation:

Center Number	Atomic Number	Atomic Type	Coordinates (Angstroms) X	Y	Z
1	6	0	0.458191	-1.158026	-0.861268
2	6	0	1.718711	-1.215364	-0.278458
3	6	0	2.339896	-0.000535	-0.013437
4	6	0	1.719323	1.214534	-0.278809
5	6	0	0.458775	1.157655	-0.861600
6	7	0	-0.114975	-0.000089	-1.181027
7	1	0	2.188202	-2.156604	-0.028727
8	1	0	2.189289	2.155608	-0.029339
9	6	0	-0.335136	2.410955	-1.155527
10	1	0	0.113769	3.251501	-0.625933
11	1	0	-0.230187	2.613890	-2.224778
12	6	0	-0.336321	-2.411025	-1.154860
13	1	0	0.112197	-3.251641	-0.625049
14	1	0	-0.231469	-2.614293	-2.224058
15	7	0	-1.754458	2.342616	-0.830995
16	1	0	-2.103704	1.448543	-1.166034
17	7	0	-1.755609	-2.341948	-0.830328
18	1	0	-2.104443	-1.447777	-1.165534
19	6	0	-2.059007	2.423502	0.599588
20	1	0	-3.138130	2.567331	0.697130
21	1	0	-1.582717	3.320357	1.001206
22	6	0	-1.658892	1.224686	1.452493
23	1	0	-0.576738	1.083852	1.419191
24	1	0	-1.906001	1.446812	2.500141
25	6	0	-2.060168	-2.422357	0.600279
26	1	0	-3.139355	-2.565671	0.697875
27	1	0	-1.584278	-3.319325	1.002112
28	6	0	-1.659489	-1.223499	1.452856
29	1	0	-0.577268	-1.083196	1.419518
30	1	0	-1.906713	-1.445207	2.500565
31	7	0	-2.289330	0.000677	0.973748
32	1	0	-3.266407	0.000957	1.249299
33	6	0	3.631273	-0.000733	0.578194
34	7	0	4.664280	-0.001057	1.048575
35	1	0	5.589147	-0.001202	1.469983

Most stable energy, Gibbs free energy (Ha), and geometry for protomer ./CN_PyN3//2_7
E: -742.084863
G: -741.807582
Geometry:

Input orientation:

Center Number	Atomic Number	Atomic Type	Coordinates (Angstroms) X	Y	Z
1	6	0	0.664728	-1.185693	-0.552705
2	6	0	1.934454	-1.200084	0.021976
3	6	0	2.557518	0.024897	0.205208
4	6	0	1.945325	1.226110	-0.143230
5	6	0	0.689189	1.120819	-0.710441
6	7	0	0.091511	-0.048491	-0.925416
7	1	0	2.408587	-2.124771	0.321225

8	1	0	2.418635	2.181211	0.034879
9	6	0	-0.104479	2.336909	-1.105223
10	1	0	0.226075	3.223490	-0.569460
11	1	0	-0.027701	2.511836	-2.177808
12	6	0	-0.078391	-2.476289	-0.817159
13	1	0	0.204521	-3.203199	-0.055203
14	1	0	0.311212	-2.858474	-1.765956
15	7	0	-1.533856	2.092004	-0.785467
16	1	0	-2.120447	2.786256	-1.252493
17	7	0	-1.525096	-2.403994	-0.895021
18	1	0	-1.786112	-1.592959	-1.447528
19	6	0	-1.840951	2.076809	0.676376
20	1	0	-1.923055	3.106350	1.014846
21	1	0	-0.996410	1.607631	1.178629
22	6	0	-3.123257	1.298281	0.901577
23	1	0	-3.306031	1.262291	1.982038
24	1	0	-3.954531	1.833575	0.440835
25	6	0	-2.216135	-2.336704	0.393653
26	1	0	-3.248260	-2.664605	0.239740
27	1	0	-1.753249	-3.062922	1.063416
28	6	0	-2.264607	-0.983820	1.088532
29	1	0	-1.256561	-0.596556	1.244720
30	1	0	-2.710700	-1.121500	2.083069
31	7	0	-3.032098	-0.024388	0.293966
32	1	0	-3.969955	-0.391935	0.174919
33	6	0	3.851769	0.056308	0.795900
34	7	0	4.884040	0.077456	1.265044
35	1	0	5.809678	0.093324	1.686274
36	1	0	-1.798138	1.158355	-1.138810

Most stable energy, Gibbs free energy (Ha), and geometry for protomer ./CN_PyN3//2_1
E: -742.145153
G: -741.863899
Geometry:
Input orientation:

Center Number	Atomic Number	Atomic Type	Coordinates (Angstroms) X	Y	Z
1	6	0	0.779495	-1.090202	-0.513088
2	6	0	2.155620	-1.148950	-0.321008
3	6	0	2.834507	0.030975	-0.064280
4	6	0	2.141664	1.231585	0.018860
5	6	0	0.771239	1.183229	-0.168135
6	7	0	0.109836	0.060672	-0.441552
7	1	0	2.678652	-2.094085	-0.374540
8	1	0	2.648326	2.164549	0.223373
9	6	0	-0.023175	2.452155	-0.018214
10	1	0	-0.206337	2.657797	1.036617
11	1	0	0.523617	3.291807	-0.438395
12	6	0	0.039523	-2.370121	-0.819547
13	1	0	0.469706	-3.158601	-0.189812
14	1	0	0.257285	-2.642035	-1.854713
15	7	0	-1.347102	2.374320	-0.707786
16	1	0	-1.475204	3.226417	-1.258469
17	7	0	-1.397019	-2.250448	-0.666897
18	1	0	-1.840499	-3.049489	-1.105272
19	6	0	-2.537993	2.254982	0.192497
20	1	0	-3.418927	2.402440	-0.430262

21	1	0	-2.469226	3.075886	0.902113
22	6	0	-2.621218	0.953157	0.950995
23	1	0	-1.679897	0.675844	1.421880
24	1	0	-3.376224	1.067045	1.726027
25	6	0	-1.823926	-2.172333	0.728996
26	1	0	-1.901733	-3.150138	1.210879
27	1	0	-1.090104	-1.591749	1.290114
28	6	0	-3.173417	-1.486571	0.789768
29	1	0	-3.508449	-1.304281	1.808063
30	1	0	-3.925662	-2.065627	0.258165
31	7	0	-3.058051	-0.172308	0.082401
32	1	0	-3.948295	0.072547	-0.355767
33	6	0	4.258945	0.013951	0.121352
34	7	0	5.397534	0.000795	0.269121
35	1	0	-2.366729	-0.345318	-0.666742
36	1	0	-1.327127	1.598145	-1.378308

Most stable energy, Gibbs free energy (Ha), and geometry for protomer ./CN_PyN3//2_3
E: -742.145153
G: -741.863902
Geometry:

Input orientation:

Center Number	Atomic Number	Atomic Type	Coordinates (Angstroms) X	Y	Z
1	6	0	0.771334	-1.183210	-0.167971
2	6	0	2.141747	-1.231461	0.019064
3	6	0	2.834492	-0.030765	-0.063855
4	6	0	2.155475	1.149121	-0.320422
5	6	0	0.779353	1.090248	-0.512585
6	7	0	0.109822	-0.060689	-0.441271
7	1	0	2.648514	-2.164390	0.223482
8	1	0	2.678390	2.094328	-0.373775
9	6	0	0.039315	2.370152	-0.819042
10	1	0	0.469319	3.158621	-0.189166
11	1	0	0.257248	2.642182	-1.854142
12	6	0	-0.022981	-2.452224	-0.018214
13	1	0	-0.206292	-2.657902	1.036595
14	1	0	0.523918	-3.291818	-0.438319
15	7	0	-1.397248	2.250361	-0.666738
16	1	0	-1.840713	3.049385	-1.105158
17	7	0	-1.346814	-2.374417	-0.707926
18	1	0	-1.474848	-3.226556	-1.258563
19	6	0	-1.824513	2.172087	0.729019
20	1	0	-1.902513	3.149834	1.210997
21	1	0	-1.090819	1.591497	1.290294
22	6	0	-3.173988	1.486242	0.789276
23	1	0	-3.509345	1.303879	1.807450
24	1	0	-3.926069	2.065338	0.257479
25	6	0	-2.537877	-2.255151	0.192146
26	1	0	-3.418637	-2.402704	-0.430842
27	1	0	-2.469228	-3.076072	0.901764
28	6	0	-2.621590	-0.953364	0.950592
29	1	0	-1.680645	-0.675705	1.422002
30	1	0	-3.376920	-1.067446	1.725294
31	7	0	-3.058323	0.172031	0.081864
32	1	0	-3.948442	-0.072951	-0.356491
33	6	0	4.258918	-0.013617	0.121882

34	7	0	5.397503	-0.000349	0.269661
35	1	0	-1.326855	-1.598247	-1.378464
36	1	0	-2.366837	0.345052	-0.667132

Most stable energy, Gibbs free energy (Ha), and geometry for protomer ./CN_PyN3//2_10
E: -742.061647
G: -741.788393
Geometry:
Input orientation:

Center Number	Atomic Number	Atomic Type	Coordinates (Angstroms) X	Y	Z
1	6	0	0.700837	-1.089834	-0.654238
2	6	0	2.057798	-1.117902	-0.394718
3	6	0	2.680955	0.107953	-0.185584
4	6	0	2.006078	1.322943	-0.200964
5	6	0	0.647799	1.282622	-0.459979
6	7	0	0.097556	0.096755	-0.702674
7	1	0	2.598763	-2.051917	-0.346913
8	1	0	2.508806	2.258774	-0.001794
9	6	0	-0.260155	2.483408	-0.446170
10	1	0	-0.101511	2.986298	0.514715
11	1	0	0.105264	3.163969	-1.217508
12	6	0	-0.171715	-2.298389	-0.875247
13	1	0	0.044707	-3.015262	-0.076464
14	1	0	0.152658	-2.760373	-1.809540
15	7	0	-1.650342	2.154360	-0.688928
16	1	0	-2.021152	2.815495	-1.354817
17	7	0	-1.573707	-1.933995	-0.954971
18	1	0	-1.987999	-2.390753	-1.755939
19	6	0	-2.541836	2.092255	0.482554
20	1	0	-3.444598	2.659889	0.255859
21	1	0	-2.067024	2.571323	1.345917
22	6	0	-2.945423	0.675467	0.855675
23	1	0	-3.747192	0.713996	1.607262
24	1	0	-3.356635	0.187635	-0.030034
25	6	0	-2.376066	-2.251995	0.230968
26	1	0	-3.411534	-2.019731	-0.017634
27	1	0	-2.334065	-3.322117	0.467450
28	6	0	-1.959746	-1.506176	1.491660
29	1	0	-1.005861	-1.903638	1.848687
30	1	0	-2.710871	-1.749570	2.256813
31	7	0	-1.783740	-0.071697	1.309783
32	1	0	-1.450345	0.333653	2.177645
33	6	0	4.083368	0.119542	0.089415
34	7	0	5.194419	0.130475	0.308182
35	1	0	6.191964	0.141016	0.512692
36	1	0	-0.925099	0.071042	-0.835084

Most stable energy, Gibbs free energy (Ha), and geometry for protomer ./CN_PyN3//2_2
E: -742.150428
G: -741.870272
Geometry:
Input orientation:

Center Number	Atomic Number	Atomic Type	Coordinates (Angstroms) X	Y	Z
1	6	0	0.860335	-1.148899	0.405283

2	6	0	2.182929	-1.203269	-0.002889
3	6	0	2.842767	0.005816	-0.182979
4	6	0	2.181834	1.211775	0.016042
5	6	0	0.859537	1.150232	0.422429
6	7	0	0.232314	-0.001476	0.628441
7	1	0	2.676735	-2.147825	-0.184444
8	1	0	2.675014	2.159333	-0.150888
9	6	0	0.058559	2.401511	0.661635
10	1	0	0.281287	2.810593	1.646746
11	1	0	0.272748	3.157613	-0.090654
12	6	0	0.062532	-2.405123	0.629361
13	1	0	0.304162	-2.837463	1.599878
14	1	0	0.262387	-3.143377	-0.144373
15	7	0	-1.391643	2.091231	0.614894
16	1	0	-1.575008	1.254797	1.184445
17	7	0	-1.388499	-2.094064	0.617979
18	1	0	-1.554061	-1.256582	1.191278
19	6	0	-1.915824	1.808107	-0.753754
20	1	0	-1.222503	1.119002	-1.230014
21	1	0	-1.915014	2.747271	-1.302686
22	6	0	-3.315679	1.235539	-0.649161
23	1	0	-3.957478	1.982302	-0.178904
24	1	0	-3.686941	1.078206	-1.666500
25	6	0	-1.948175	-1.815576	-0.738067
26	1	0	-1.268486	-1.127994	-1.235752
27	1	0	-1.960508	-2.757318	-1.282364
28	6	0	-3.343974	-1.242134	-0.602548
29	1	0	-3.966389	-1.970175	-0.079563
30	1	0	-3.753892	-1.124555	-1.610488
31	7	0	-3.376009	0.011441	0.150881
32	1	0	-4.227688	0.030704	0.694273
33	6	0	4.215143	0.009898	-0.608182
34	7	0	5.311741	0.013502	-0.947874
35	1	0	-1.905087	-2.869135	1.040247
36	1	0	-1.918335	2.867507	1.022336

Most stable energy, Gibbs free energy (Ha), and geometry for protomer ./CN_PyN3//2_5

E: -742.140842

G: -741.860741

Geometry:

Input orientation:

Center Number	Atomic Number	Atomic Type	Coordinates (Angstroms) X	Y	Z
1	6	0	0.810400	-1.219373	-0.420119
2	6	0	2.123947	-1.230762	0.009374
3	6	0	2.787648	-0.014254	0.104351
4	6	0	2.158113	1.194508	-0.187909
5	6	0	0.848807	1.154387	-0.611988
6	7	0	0.266745	-0.044862	-0.734711
7	1	0	2.605188	-2.161501	0.273827
8	1	0	2.667491	2.139828	-0.069427
9	6	0	-0.007514	2.348165	-0.931649
10	1	0	0.332368	3.182270	-0.309560
11	1	0	0.166598	2.621504	-1.974129
12	6	0	-0.060148	-2.439736	-0.557236
13	1	0	0.080077	-3.044365	0.343901
14	1	0	0.337039	-3.019906	-1.392382

15	7	0	-1.414000	2.019829	-0.752928
16	1	0	-1.978498	2.670829	-1.287294
17	7	0	-1.457691	-2.116851	-0.802340
18	1	0	-1.796374	-2.684311	-1.565858
19	6	0	-1.819276	2.056971	0.652825
20	1	0	-1.859164	3.076595	1.048023
21	1	0	-1.074182	1.522045	1.245063
22	6	0	-3.189095	1.441018	0.842535
23	1	0	-3.462915	1.413229	1.895010
24	1	0	-3.942607	2.000398	0.290404
25	6	0	-2.353853	-2.265448	0.347356
26	1	0	-3.332224	-2.575899	-0.021385
27	1	0	-2.003195	-3.037977	1.037184
28	6	0	-2.508416	-0.980322	1.135833
29	1	0	-1.541744	-0.563631	1.412487
30	1	0	-3.087551	-1.142835	2.042064
31	7	0	-3.245969	0.038023	0.327104
32	1	0	-4.224878	-0.252188	0.264260
33	6	0	4.154331	0.001429	0.551905
34	7	0	5.244854	0.013136	0.907976
35	1	0	-0.708418	-0.061830	-1.050289
36	1	0	-2.881838	0.033290	-0.631863

Most stable energy, Gibbs free energy (Ha), and geometry for protomer ./CN_PyN3//2_8
E: -742.090849
G: -741.814737
Geometry:
Input orientation:

Center Number	Atomic Number	Atomic Type	Coordinates (Angstroms) X	Y	Z
1	6	0	0.615030	-1.160637	-0.353475
2	6	0	1.937707	-1.214223	0.069993
3	6	0	2.579078	0.000422	0.270632
4	6	0	1.937331	1.214853	0.069897
5	6	0	0.614670	1.160820	-0.353568
6	7	0	-0.002908	-0.000013	-0.560272
7	1	0	2.442226	-2.156073	0.236668
8	1	0	2.441560	2.156871	0.236497
9	6	0	-0.125504	2.461275	-0.586974
10	1	0	-0.045998	3.053461	0.334043
11	1	0	0.433462	3.007532	-1.348612
12	6	0	-0.124729	-2.461348	-0.586756
13	1	0	-0.044984	-3.053431	0.334307
14	1	0	0.434381	-3.007484	-1.348375
15	7	0	-1.504418	2.321595	-1.025617
16	1	0	-1.694776	3.036553	-1.714604
17	7	0	-1.503711	-2.322160	-1.025335
18	1	0	-1.693881	-3.037256	-1.714231
19	6	0	-2.493125	2.447161	0.042114
20	1	0	-3.476665	2.545748	-0.418676
21	1	0	-2.325100	3.332560	0.666656
22	6	0	-2.486697	1.244745	0.960448
23	1	0	-1.528851	1.137286	1.470279
24	1	0	-3.272040	1.317560	1.709938
25	6	0	-2.492303	-2.447913	0.042477
26	1	0	-3.475839	-2.546912	-0.418232
27	1	0	-2.323926	-3.333162	0.667137

28	6	0	-2.486229	-1.245358	0.960632
29	1	0	-1.528387	-1.137499	1.470393
30	1	0	-3.271506	-1.318327	1.710176
31	7	0	-2.697887	-0.000404	0.179432
32	1	0	-3.631476	-0.000605	-0.238276
33	6	0	3.935828	0.000639	0.700377
34	7	0	5.017247	0.000788	1.043106
35	1	0	5.986669	0.000933	1.349065
36	1	0	-1.991732	-0.000327	-0.577781

Most stable energy, Gibbs free energy (Ha), and geometry for protomer ./CN_PyN3//2_9
E: -742.084863
G: -741.807605
Geometry:
Input orientation:

Center Number	Atomic Number	Atomic Type	Coordinates (Angstroms) X	Y	Z
1	6	0	0.688982	-1.121282	0.709135
2	6	0	1.945100	-1.226835	0.141845
3	6	0	2.557709	-0.025710	-0.206090
4	6	0	1.935081	1.199462	-0.022382
5	6	0	0.665421	1.185313	0.552313
6	7	0	0.091799	0.048130	0.924564
7	1	0	2.418036	-2.182044	-0.036684
8	1	0	2.409631	2.124079	-0.321197
9	6	0	-0.077394	2.476005	0.817494
10	1	0	0.313090	2.858045	1.765969
11	1	0	0.204857	3.203036	0.055445
12	6	0	-0.105012	-2.337250	1.103708
13	1	0	-0.027468	-2.513050	2.176096
14	1	0	0.224773	-3.223602	0.567078
15	7	0	-1.524031	2.403601	0.896481
16	1	0	-1.784571	1.591965	1.448354
17	7	0	-1.534497	-2.091479	0.785260
18	1	0	-1.797945	-1.157697	1.138936
19	6	0	-2.216029	2.337525	-0.391733
20	1	0	-1.752893	3.063490	-1.061598
21	1	0	-3.247745	2.666410	-0.237117
22	6	0	-2.266039	0.984947	-1.087001
23	1	0	-2.713120	1.123165	-2.080991
24	1	0	-1.258375	0.597197	-1.244256
25	6	0	-1.842923	-2.075803	-0.676312
26	1	0	-0.998710	-1.606654	-1.179138
27	1	0	-1.925568	-3.105226	-1.015017
28	6	0	-3.125255	-1.296916	-0.900104
29	1	0	-3.956270	-1.832147	-0.438860
30	1	0	-3.308863	-1.260365	-1.980389
31	7	0	-3.033191	0.025539	-0.291983
32	1	0	-3.970855	0.393313	-0.172107
33	6	0	3.852077	-0.057297	-0.796576
34	7	0	4.884394	-0.078124	-1.265627
35	1	0	5.810026	-0.093893	-1.686864
36	1	0	-2.121128	-2.785388	1.252749

Most stable energy, Gibbs free energy (Ha), and geometry for protomer ./CN_PyN3//3_1
E: -742.590521
G: -742.292963
Geometry:

Input orientation:

Center Number	Atomic Number	Atomic Type	X	Y	Z
1	6	0	0.608670	-1.324840	-0.105754
2	6	0	1.946051	-1.318750	0.274264
3	6	0	2.642048	-0.128898	0.151274
4	6	0	2.005641	1.011816	-0.331649
5	6	0	0.674896	0.900329	-0.684676
6	7	0	0.000403	-0.249420	-0.583482
7	1	0	2.422668	-2.214657	0.648010
8	1	0	2.532343	1.950972	-0.430037
9	6	0	-0.100162	2.091558	-1.167648
10	1	0	0.538884	2.780274	-1.714476
11	1	0	-0.927080	1.795433	-1.808267
12	6	0	-0.189972	-2.590758	0.043445
13	1	0	-0.408002	-2.783601	1.094643
14	1	0	0.375151	-3.433458	-0.347814
15	7	0	-0.661836	2.873369	-0.015794
16	1	0	0.122213	3.291572	0.495860
17	7	0	-1.486505	-2.532888	-0.693052
18	1	0	-1.668374	-3.462569	-1.080412
19	6	0	-1.505160	2.144489	0.984885
20	1	0	-1.714213	2.873345	1.763802
21	1	0	-0.896781	1.359006	1.426622
22	6	0	-2.836667	1.664165	0.445984
23	1	0	-3.541756	1.608514	1.272418
24	1	0	-3.231405	2.353579	-0.299134
25	6	0	-2.696498	-2.171620	0.116131
26	1	0	-3.551415	-2.252110	-0.553254
27	1	0	-2.781480	-2.931076	0.890230
28	6	0	-2.652265	-0.819614	0.786601
29	1	0	-1.733187	-0.663265	1.346136
30	1	0	-3.488318	-0.767759	1.481328
31	7	0	-2.828456	0.303438	-0.179584
32	1	0	-3.725273	0.178596	-0.659550
33	6	0	4.028854	-0.069924	0.520920
34	7	0	5.137065	-0.021117	0.815586
35	1	0	-1.390664	-1.906157	-1.499972
36	1	0	-2.092256	0.239928	-0.891582
37	1	0	-1.206970	3.651822	-0.401161

Most stable energy, Gibbs free energy (Ha), and geometry for protomer ./CN_PyN3//3_7

E: -742.538341

G: -742.245722

Geometry:

Input orientation:

Center Number	Atomic Number	Atomic Type	X	Y	Z
1	6	0	0.758759	-1.161999	-0.107290
2	6	0	2.131071	-1.213846	0.061684
3	6	0	2.804882	-0.001540	-0.027269
4	6	0	2.138742	1.187609	-0.271177
5	6	0	0.758316	1.121344	-0.445488
6	7	0	0.100507	-0.033438	-0.365076
7	1	0	2.649086	-2.142754	0.254287

8	1	0	2.665823	2.129996	-0.329117
9	6	0	0.010228	2.398233	-0.738717
10	1	0	0.402034	3.169741	-0.063891
11	1	0	0.272108	2.707862	-1.752738
12	6	0	-0.035211	-2.428764	0.050174
13	1	0	-0.255012	-2.598734	1.104505
14	1	0	0.533928	-3.277071	-0.319930
15	7	0	-1.427414	2.249863	-0.652839
16	1	0	-1.865532	3.042928	-1.107169
17	7	0	-1.334140	-2.389311	-0.688798
18	1	0	-1.424222	-3.258039	-1.221792
19	6	0	-1.913854	2.156929	0.722753
20	1	0	-2.024003	3.131269	1.205415
21	1	0	-1.196949	1.583235	1.312157
22	6	0	-3.256347	1.456292	0.723402
23	1	0	-3.635188	1.273966	1.726224
24	1	0	-3.989811	2.025917	0.156496
25	6	0	-2.556897	-2.280447	0.170186
26	1	0	-3.414360	-2.442779	-0.480881
27	1	0	-2.499834	-3.096401	0.886700
28	6	0	-2.680386	-0.975790	0.917206
29	1	0	-1.760028	-0.686412	1.421472
30	1	0	-3.461947	-1.093190	1.665037
31	7	0	-3.098514	0.140219	0.027405
32	1	0	-3.970788	-0.117703	-0.438994
33	6	0	4.218896	0.017544	0.141190
34	7	0	5.344396	0.033933	0.276795
35	1	0	6.353106	0.051608	0.406540
36	1	0	-1.313631	-1.634161	-1.382540
37	1	0	-2.386604	0.316148	-0.700588

Most stable energy, Gibbs free energy (Ha), and geometry for protomer ./CN_PyN3//3_5
E: -742.538341
G: -742.245733
Geometry:

Input orientation:

Center Number	Atomic Number	Atomic Type	Coordinates (Angstroms) X	Y	Z
1	6	0	0.759250	-1.120840	-0.445164
2	6	0	2.139731	-1.186276	-0.270930
3	6	0	2.805184	0.003337	-0.027380
4	6	0	2.130693	1.215275	0.061347
5	6	0	0.758397	1.162588	-0.107506
6	7	0	0.100798	0.033588	-0.364967
7	1	0	2.667382	-2.128355	-0.328670
8	1	0	2.648199	2.144530	0.253633
9	6	0	-0.036325	2.428899	0.049764
10	1	0	-0.256117	2.598904	1.104099
11	1	0	0.532249	3.277502	-0.320535
12	6	0	0.011854	-2.398190	-0.738164
13	1	0	0.403893	-3.169297	-0.063007
14	1	0	0.274119	-2.708006	-1.752025
15	7	0	-1.335298	2.388528	-0.689085
16	1	0	-1.425951	3.257107	-1.222222
17	7	0	-1.425887	-2.250523	-0.652636
18	1	0	-1.863491	-3.043843	-1.107016
19	6	0	-2.557934	2.279065	0.170020

20	1	0	-3.415520	2.441063	-0.480974
21	1	0	-2.501181	3.095011	0.886571
22	6	0	-2.680826	0.974313	0.917001
23	1	0	-1.760306	0.685299	1.421178
24	1	0	-3.462361	1.091369	1.664914
25	6	0	-1.912671	-2.157761	0.722855
26	1	0	-2.022275	-3.132134	1.205583
27	1	0	-1.196204	-1.583585	1.312321
28	6	0	-3.255636	-1.458035	0.723241
29	1	0	-3.634806	-1.275920	1.725977
30	1	0	-3.988595	-2.028161	0.156180
31	7	0	-3.098604	-0.141875	0.027244
32	1	0	-3.971104	0.115624	-0.438976
33	6	0	4.219223	-0.014851	0.140944
34	7	0	5.344747	-0.030408	0.276476
35	1	0	6.353466	-0.047388	0.406212
36	1	0	-2.386772	-0.317496	-0.700876
37	1	0	-1.314356	1.633283	-1.382720

Most stable energy, Gibbs free energy (Ha), and geometry for protomer ./CN_PyN3//3_2
E: -742.572310
G: -742.276615
Geometry:
Input orientation:

Center Number	Atomic Number	Atomic Type	Coordinates (Angstroms) X	Y	Z
1	6	0	0.713008	-1.218280	0.750338
2	6	0	1.971241	-1.298423	0.179918
3	6	0	2.640709	-0.120726	-0.110132
4	6	0	2.057665	1.124235	0.129297
5	6	0	0.808903	1.159172	0.701152
6	7	0	0.219963	-0.006478	1.021796
7	1	0	2.401291	-2.264174	-0.044462
8	1	0	2.564041	2.043444	-0.128597
9	6	0	0.074743	2.436803	0.974783
10	1	0	-0.523733	2.363357	1.880746
11	1	0	0.800673	3.237952	1.084535
12	6	0	-0.168063	-2.396528	1.056359
13	1	0	-0.072452	-2.629295	2.118840
14	1	0	0.210871	-3.251852	0.489500
15	7	0	-0.846180	2.849403	-0.141701
16	1	0	-0.982286	3.859070	-0.031666
17	7	0	-1.554210	-2.062662	0.761025
18	1	0	-2.163814	-2.708815	1.250119
19	6	0	-2.212204	2.225571	-0.175675
20	1	0	-2.883237	2.976223	-0.582384
21	1	0	-2.491583	2.037603	0.860419
22	6	0	-2.254063	0.951049	-1.013072
23	1	0	-2.604229	1.147312	-2.022920
24	1	0	-1.277423	0.480936	-1.087446
25	6	0	-1.838686	-2.100872	-0.674695
26	1	0	-1.013893	-1.620133	-1.204034
27	1	0	-1.904678	-3.121286	-1.063010
28	6	0	-3.146640	-1.405532	-0.994591
29	1	0	-3.989940	-1.942696	-0.564877
30	1	0	-3.289933	-1.307735	-2.068474
31	7	0	-3.191677	-0.031306	-0.400058

32	1	0	-2.968539	-0.125008	0.598181
33	6	0	3.951055	-0.178825	-0.696841
34	7	0	4.998509	-0.221749	-1.161963
35	1	0	-0.711614	0.009192	1.450188
36	1	0	-4.147003	0.330423	-0.463236
37	1	0	-0.374131	2.727726	-1.045808

Most stable energy, Gibbs free energy (Ha), and geometry for protomer ./CN_PyN3//3_9
E: -742.528918
G: -742.237547
Geometry:
Input orientation:

Center Number	Atomic Number	Atomic Type	Coordinates (Angstroms) X	Y	Z
1	6	0	0.794455	-1.211694	0.383482
2	6	0	2.118086	-1.232043	-0.023343
3	6	0	2.768522	-0.008748	-0.102217
4	6	0	2.154390	1.210189	0.171862
5	6	0	0.835114	1.168223	0.572299
6	7	0	0.256357	-0.032324	0.683636
7	1	0	2.608581	-2.161393	-0.276255
8	1	0	2.674293	2.150974	0.062551
9	6	0	-0.027381	2.360753	0.876852
10	1	0	0.168965	2.661636	1.907892
11	1	0	0.294032	3.180845	0.226755
12	6	0	-0.086335	-2.426018	0.501481
13	1	0	0.339740	-3.050403	1.289135
14	1	0	0.011509	-2.985695	-0.433831
15	7	0	-1.431739	2.014012	0.735065
16	1	0	-1.991249	2.655016	1.286484
17	7	0	-1.467949	-2.095440	0.810423
18	1	0	-1.768806	-2.641342	1.604992
19	6	0	-1.877060	2.041973	-0.658910
20	1	0	-1.153013	1.496748	-1.267777
21	1	0	-1.920864	3.058906	-1.060480
22	6	0	-3.257002	1.436518	-0.804195
23	1	0	-3.989273	2.008127	-0.236180
24	1	0	-3.559781	1.403820	-1.848503
25	6	0	-2.422334	-2.264889	-0.289041
26	1	0	-2.110588	-3.054898	-0.977177
27	1	0	-3.381901	-2.561213	0.136203
28	6	0	-2.607403	-0.994845	-1.094630
29	1	0	-3.218904	-1.171927	-1.976427
30	1	0	-1.650462	-0.584630	-1.412594
31	7	0	-3.315488	0.038078	-0.276544
32	1	0	-2.931914	0.036185	0.674798
33	6	0	4.138579	0.001106	-0.519155
34	7	0	5.221428	0.005919	-0.847840
35	1	0	6.196675	0.009378	-1.143294
36	1	0	-0.725066	-0.046288	0.989151
37	1	0	-4.295850	-0.241149	-0.190136

Most stable energy, Gibbs free energy (Ha), and geometry for protomer ./CN_PyN3//3_6
E: -742.542595
G: -742.251569
Geometry:
Input orientation:

Center Number	Atomic Number	Atomic Type	Coordinates (Angstroms) X	Y	Z
1	6	0	0.834573	-1.154565	0.413931
2	6	0	2.167105	-1.213798	0.036267
3	6	0	2.815041	0.004997	-0.120956
4	6	0	2.167330	1.220435	0.064340
5	6	0	0.834944	1.153408	0.438709
6	7	0	0.210335	-0.002952	0.620882
7	1	0	2.671123	-2.154446	-0.136588
8	1	0	2.671784	2.164507	-0.086960
9	6	0	0.029275	2.402337	0.668595
10	1	0	0.248806	2.813018	1.653865
11	1	0	0.249799	3.155799	-0.084669
12	6	0	0.031259	-2.408796	0.622743
13	1	0	0.274012	-2.850397	1.588894
14	1	0	0.232954	-3.138856	-0.158410
15	7	0	-1.419348	2.091528	0.612890
16	1	0	-1.612212	1.259244	1.185535
17	7	0	-1.418399	-2.095734	0.612307
18	1	0	-1.587965	-1.261882	1.189789
19	6	0	-1.934103	1.806230	-0.759461
20	1	0	-1.237871	1.116156	-1.230141
21	1	0	-1.928440	2.744763	-1.309377
22	6	0	-3.335123	1.235101	-0.662385
23	1	0	-3.979608	1.984791	-0.200637
24	1	0	-3.698861	1.073018	-1.681588
25	6	0	-1.980076	-1.816425	-0.743576
26	1	0	-1.301442	-1.129452	-1.243637
27	1	0	-1.993613	-2.758551	-1.287068
28	6	0	-3.374808	-1.241805	-0.604801
29	1	0	-3.995628	-1.966897	-0.075895
30	1	0	-3.788580	-1.128356	-1.611592
31	7	0	-3.401393	0.014956	0.143193
32	1	0	-4.252695	0.040362	0.686958
33	6	0	4.185105	0.009889	-0.515594
34	7	0	5.273931	0.014600	-0.829919
35	1	0	6.251475	0.019024	-1.112575
36	1	0	-1.933470	-2.873427	1.032338
37	1	0	-1.946892	2.871284	1.013299

Most stable energy, Gibbs free energy (Ha), and geometry for protomer ./CN_PyN3//3_4
E: -742.572309
G: -742.276577
Geometry:
Input orientation:

Center Number	Atomic Number	Atomic Type	Coordinates (Angstroms) X	Y	Z
1	6	0	0.809156	-1.158481	0.701718
2	6	0	2.058145	-1.123745	0.130376
3	6	0	2.641098	0.121177	-0.109560
4	6	0	1.971372	1.298960	0.179512
5	6	0	0.712923	1.219001	0.749499
6	7	0	0.219962	0.007291	1.021434
7	1	0	2.564771	-2.043033	-0.126751
8	1	0	2.401407	2.264631	-0.045248
9	6	0	-0.168392	2.397276	1.054739

10	1	0	-0.072465	2.630981	2.116987
11	1	0	0.210145	3.252208	0.487025
12	6	0	0.074808	-2.435865	0.975906
13	1	0	-0.524455	-2.361525	1.881273
14	1	0	0.800602	-3.236927	1.087092
15	7	0	-1.554530	2.062798	0.760155
16	1	0	-2.164189	2.708824	1.249339
17	7	0	-0.845120	-2.849572	-0.141027
18	1	0	-0.980685	-3.859270	-0.030655
19	6	0	-1.839727	2.100370	-0.675444
20	1	0	-1.014908	1.619957	-1.205022
21	1	0	-1.906424	3.120638	-1.064029
22	6	0	-3.147471	1.404308	-0.994631
23	1	0	-3.990843	1.941065	-0.564552
24	1	0	-3.291211	1.306423	-2.068446
25	6	0	-2.211539	-2.226663	-0.176315
26	1	0	-2.881462	-2.977510	-0.584499
27	1	0	-2.492424	-2.039698	0.859547
28	6	0	-2.253097	-0.951596	-1.012890
29	1	0	-2.602117	-1.147319	-2.023237
30	1	0	-1.276560	-0.481055	-1.085897
31	7	0	-3.191686	0.030014	-0.400168
32	1	0	-2.969098	0.123663	0.598187
33	6	0	3.951699	0.179106	-0.695724
34	7	0	4.999361	0.221993	-1.160382
35	1	0	-0.711729	-0.008224	1.449580
36	1	0	-0.372567	-2.727981	-1.044880
37	1	0	-4.146713	-0.332425	-0.463818

Most stable energy, Gibbs free energy (Ha), and geometry for protomer ./OMe_PyN3//0_1
E: -763.511566
G: -763.225132
Geometry:

Input orientation:

Center Number	Atomic Number	Atomic Type	Coordinates (Angstroms) X	Y	Z
1	6	0	-1.460128	1.554558	0.182884
2	1	0	-1.757235	2.558927	-0.089770
3	6	0	-2.313948	0.481392	-0.066904
4	6	0	-1.879236	-0.804354	0.230145
5	1	0	-2.475571	-1.678822	0.013556
6	7	0	0.171542	0.073380	1.100260
7	7	0	2.143759	2.203461	0.749083
8	1	0	2.449303	1.380401	1.256637
9	7	0	2.368479	-0.369277	-0.858618
10	1	0	2.142431	-0.299203	0.129747
11	7	0	1.306697	-2.600595	0.923295
12	1	0	1.849367	-1.878986	1.385613
13	6	0	0.734845	2.442177	1.027909
14	1	0	0.413567	3.316019	0.459112
15	1	0	0.640852	2.700432	2.086244
16	6	0	-0.227586	1.304057	0.754134
17	6	0	2.501219	2.090788	-0.666092
18	1	0	3.591460	2.070271	-0.724457
19	1	0	2.171207	3.005570	-1.163230
20	6	0	-0.620236	-0.954182	0.804527
21	6	0	1.952256	0.901169	-1.445078

22	1	0	0.857117	0.968740	-1.507378
23	1	0	2.325303	0.964905	-2.470861
24	6	0	-0.109108	-2.341880	1.141474
25	1	0	-0.328031	-2.517249	2.198595
26	1	0	-0.685103	-3.075402	0.575370
27	6	0	1.714812	-2.720492	-0.478176
28	1	0	1.129293	-3.527296	-0.924141
29	1	0	2.758908	-3.039930	-0.486363
30	6	0	1.575519	-1.487061	-1.361667
31	1	0	1.913208	-1.747046	-2.368514
32	1	0	0.514377	-1.213212	-1.450675
33	8	0	-3.510175	0.772805	-0.620230
34	6	0	-4.388290	-0.311080	-0.905458
35	1	0	-4.642889	-0.852160	0.007221
36	1	0	-5.283546	0.133690	-1.330565
37	1	0	-3.937538	-0.993657	-1.627640

Most stable energy, Gibbs free energy (Ha), and geometry for protomer ./OMe_PyN3//1_3

E: -763.974800

G: -763.674933

Geometry:

Input orientation:

Center Number	Atomic Number	Atomic Type	Coordinates (Angstroms) X	Y	Z
1	6	0	-1.966846	0.921661	0.085019
2	1	0	-2.489322	1.825996	-0.191573
3	6	0	-2.479020	-0.341014	-0.191680
4	6	0	-1.721099	-1.467412	0.140756
5	1	0	-2.069974	-2.458800	-0.114879
6	7	0	-0.036804	-0.061062	1.088231
7	7	0	1.320305	2.461077	0.967009
8	1	0	1.723512	1.668246	1.457534
9	7	0	2.962772	0.285031	-0.382822
10	1	0	3.850378	0.773409	-0.329924
11	7	0	1.785292	-2.015145	0.738344
12	1	0	2.466223	-2.639853	1.172877
13	6	0	-0.129416	2.358402	1.050610
14	1	0	-0.575137	3.115016	0.404324
15	1	0	-0.434029	2.600777	2.072942
16	6	0	-0.732296	1.009504	0.720998
17	6	0	1.859003	2.478342	-0.394537
18	1	0	2.847229	2.946860	-0.361541
19	1	0	1.225296	3.126253	-1.002388
20	6	0	-0.515160	-1.266698	0.771297
21	6	0	2.015186	1.138103	-1.101039
22	1	0	1.053868	0.627388	-1.172026
23	1	0	2.353425	1.328642	-2.129106
24	6	0	0.400141	-2.413070	1.112062
25	1	0	0.395655	-2.612984	2.182703
26	1	0	0.140968	-3.319667	0.570942
27	6	0	2.024998	-1.951293	-0.733430
28	1	0	1.104320	-1.595862	-1.192887
29	1	0	2.226211	-2.958927	-1.087978
30	6	0	3.182912	-1.011400	-1.013529
31	1	0	4.100411	-1.439609	-0.607758
32	1	0	3.298290	-0.944167	-2.102070
33	8	0	-3.657129	-0.568659	-0.799281

34	6	0	-4.443296	0.560687	-1.170464
35	1	0	-5.342262	0.159230	-1.628875
36	1	0	-4.707272	1.150222	-0.291384
37	1	0	-3.907544	1.182507	-1.889068
38	1	0	1.945422	-1.059009	1.094716

Most stable energy, Gibbs free energy (Ha), and geometry for protomer ./OMe_PyN3//1_2
E: -763.978804
G: -763.678743
Geometry:
Input orientation:

Center Number	Atomic Number	Atomic Type	Coordinates (Angstroms) X	Y	Z
1	6	0	-1.862147	0.933682	0.033813
2	1	0	-2.390089	1.849589	-0.189252
3	6	0	-2.363985	-0.315883	-0.311883
4	6	0	-1.601012	-1.451710	-0.042200
5	1	0	-1.957353	-2.427979	-0.344154
6	7	0	0.079438	-0.099199	0.967578
7	7	0	1.387196	2.370316	1.208163
8	1	0	1.622746	3.104570	1.862679
9	7	0	2.537432	0.201393	-0.275118
10	1	0	3.519023	0.288220	-0.001503
11	7	0	1.881477	-2.255273	1.040027
12	1	0	2.279960	-2.965744	1.639480
13	6	0	-0.061240	2.339356	1.057951
14	1	0	-0.415564	3.078075	0.328010
15	1	0	-0.504178	2.609997	2.019051
16	6	0	-0.626519	0.987610	0.668541
17	6	0	2.117597	2.621950	-0.030428
18	1	0	3.152583	2.845678	0.230873
19	1	0	1.720044	3.479969	-0.586753
20	6	0	-0.386019	-1.297492	0.594470
21	6	0	2.087163	1.433976	-0.971205
22	1	0	1.079417	1.235603	-1.336628
23	1	0	2.736819	1.601008	-1.827937
24	6	0	0.454296	-2.518582	0.909183
25	1	0	0.101622	-2.917994	1.862630
26	1	0	0.243805	-3.280988	0.148906
27	6	0	2.617724	-2.265036	-0.220072
28	1	0	2.384229	-3.141380	-0.837629
29	1	0	3.682260	-2.298069	0.014846
30	6	0	2.332784	-1.039418	-1.065624
31	1	0	2.988646	-1.006198	-1.933351
32	1	0	1.299963	-1.020134	-1.412916
33	8	0	-3.546486	-0.515488	-0.926089
34	6	0	-4.344139	0.627692	-1.219653
35	1	0	-3.821631	1.296782	-1.904859
36	1	0	-5.245576	0.248562	-1.692257
37	1	0	-4.603183	1.161598	-0.304144
38	1	0	1.957533	0.110119	0.583050

Most stable energy, Gibbs free energy (Ha), and geometry for protomer ./OMe_PyN3//1_1
E: -763.974800
G: -763.674930
Geometry:
Input orientation:

Center Number	Atomic Number	Atomic Type	Coordinates (Angstroms) X	Y	Z
1	6	0	1.720374	1.468179	-0.140751
2	1	0	2.068787	2.459621	0.115305
3	6	0	2.478837	0.341996	0.191171
4	6	0	1.967238	-0.920804	-0.086039
5	1	0	2.490146	-1.825012	0.190151
6	7	0	0.036696	0.061449	-1.088759
7	7	0	-1.786244	2.014603	-0.737427
8	1	0	-1.946047	1.058477	-1.093973
9	7	0	-2.962087	-0.286463	0.383528
10	1	0	-3.849413	-0.775370	0.330830
11	7	0	-1.319310	-2.461380	-0.967591
12	1	0	-1.723216	-1.668658	-1.457720
13	6	0	-0.401387	2.413245	-1.111502
14	1	0	-0.142444	3.319819	-0.570234
15	1	0	-0.397348	2.613427	-2.182094
16	6	0	0.514508	1.267173	-0.771338
17	6	0	-2.025475	1.950304	0.734405
18	1	0	-2.227155	2.957746	1.089232
19	1	0	-1.104463	1.595291	1.193510
20	6	0	0.732694	-1.008949	-0.721986
21	6	0	-3.182756	1.009668	1.014661
22	1	0	-3.297712	0.942080	2.103226
23	1	0	-4.100647	1.437447	0.609327
24	6	0	0.130315	-2.357963	-1.052034
25	1	0	0.434382	-2.599592	-2.074701
26	1	0	0.576799	-3.114735	-0.406459
27	6	0	-1.857174	-2.479183	0.394277
28	1	0	-1.222750	-3.126851	1.001640
29	1	0	-2.845163	-2.948239	0.361799
30	6	0	-2.013682	-1.139155	1.101106
31	1	0	-2.351287	-1.330070	2.129313
32	1	0	-1.052595	-0.627943	1.171694
33	8	0	3.656883	0.569944	0.798780
34	6	0	4.443645	-0.559179	1.169378
35	1	0	5.342446	-0.157483	1.627904
36	1	0	3.908266	-1.181601	1.887737
37	1	0	4.707847	-1.148174	0.290002
38	1	0	-2.467593	2.639095	-1.171612

Most stable energy, Gibbs free energy (Ha), and geometry for protomer ./OMe_PyN3//1_4

E: -763.971952

G: -763.674706

Geometry:

Input orientation:

Center Number	Atomic Number	Atomic Type	Coordinates (Angstroms) X	Y	Z
1	6	0	-2.085566	0.933911	0.000740
2	1	0	-2.670829	1.839177	-0.059104
3	6	0	-2.654596	-0.331687	-0.129744
4	6	0	-1.840400	-1.463052	-0.030733
5	1	0	-2.270262	-2.450880	-0.123463
6	7	0	0.022410	-0.073284	0.321238
7	7	0	1.216874	2.409730	0.974432
8	1	0	1.220573	3.157582	1.654483

9	7	0	2.704493	0.197744	-0.078934
10	1	0	3.494137	0.269971	0.555901
11	7	0	1.683328	-2.291396	0.915261
12	1	0	1.834448	-3.041419	1.575920
13	6	0	-0.079475	2.393466	0.318986
14	1	0	-0.024227	2.791198	-0.702606
15	1	0	-0.777986	3.033260	0.857265
16	6	0	-0.729080	1.034834	0.221885
17	6	0	2.347187	2.609324	0.065160
18	1	0	3.238797	2.756643	0.676999
19	1	0	2.218835	3.503842	-0.556584
20	6	0	-0.496771	-1.312481	0.188957
21	6	0	2.563522	1.421764	-0.852726
22	1	0	1.700772	1.296540	-1.516686
23	1	0	3.428270	1.619355	-1.496806
24	6	0	0.410871	-2.516560	0.250007
25	1	0	-0.146213	-3.299048	0.764136
26	1	0	0.548298	-2.865400	-0.781341
27	6	0	2.832256	-2.239608	0.008662
28	1	0	2.885554	-3.126712	-0.634468
29	1	0	3.734650	-2.220720	0.622131
30	6	0	2.808503	-1.011712	-0.880734
31	1	0	3.694889	-1.018698	-1.525699
32	1	0	1.938031	-1.043931	-1.545401
33	8	0	-3.952184	-0.549770	-0.339044
34	6	0	-4.820350	0.579823	-0.451348
35	1	0	-4.525547	1.202866	-1.295989
36	1	0	-5.812154	0.171868	-0.619521
37	1	0	-4.808265	1.161680	0.470411
38	1	0	1.082121	0.031017	0.435186

Most stable energy, Gibbs free energy (Ha), and geometry for protomer ./OMe_PyN3//2_1
E: -764.430355
G: -764.114884
Geometry:
Input orientation:

Center Number	Atomic Number	Atomic Type	Coordinates (Angstroms) X	Y	Z
1	6	0	-1.871478	1.455942	-0.146404
2	1	0	-2.277984	2.407183	-0.463839
3	6	0	-2.667569	0.314171	-0.132397
4	6	0	-2.099326	-0.894134	0.260762
5	1	0	-2.661676	-1.816262	0.262437
6	7	0	-0.003464	0.188516	0.651581
7	7	0	1.678986	2.299779	0.757290
8	1	0	1.614525	1.506997	1.407280
9	7	0	3.034945	-0.410400	-0.184810
10	1	0	3.985789	-0.242036	0.150021
11	7	0	1.271920	-2.284136	0.780175
12	1	0	1.659954	-3.115346	1.211265
13	6	0	0.313349	2.564300	0.220636
14	1	0	0.418361	2.941564	-0.795678
15	1	0	-0.131763	3.347156	0.830869
16	6	0	-0.556245	1.332711	0.248276
17	6	0	2.740642	2.051615	-0.268384
18	1	0	3.696819	2.101800	0.250464
19	1	0	2.684067	2.875234	-0.975755

20	6	0	-0.766534	-0.908283	0.644076
21	6	0	2.608454	0.746461	-1.017057
22	1	0	1.598267	0.558066	-1.374496
23	1	0	3.279670	0.786039	-1.872515
24	6	0	-0.150322	-2.219112	1.074228
25	1	0	-0.280964	-2.324582	2.153784
26	1	0	-0.717372	-3.028534	0.600989
27	6	0	1.567657	-2.299260	-0.651439
28	1	0	0.838225	-1.674992	-1.170316
29	1	0	1.514978	-3.296860	-1.094358
30	6	0	2.959263	-1.738920	-0.868673
31	1	0	3.709224	-2.373793	-0.401274
32	1	0	3.201072	-1.605035	-1.920373
33	8	0	-3.945737	0.459584	-0.520636
34	6	0	-4.777016	-0.699157	-0.521067
35	1	0	-5.754163	-0.362954	-0.855018
36	1	0	-4.388741	-1.450122	-1.210349
37	1	0	-4.852776	-1.117518	0.483422
38	1	0	2.416127	-0.514595	0.638613
39	1	0	1.974917	3.110070	1.305243

Most stable energy, Gibbs free energy (Ha), and geometry for protomer ./OMe_PyN3//2_3
E: -764.430357
G: -764.114860
Geometry:

Input orientation:

Center Number	Atomic Number	Atomic Type	Coordinates (Angstroms) X	Y	Z
1	6	0	-2.100298	0.891941	0.261870
2	1	0	-2.663539	1.813525	0.264238
3	6	0	-2.667452	-0.316659	-0.131945
4	6	0	-1.870253	-1.457652	-0.146862
5	1	0	-2.275903	-2.409059	-0.464894
6	7	0	-0.003275	-0.188919	0.651448
7	7	0	1.269664	2.285008	0.780015
8	1	0	1.657257	3.116379	1.211193
9	7	0	3.033719	0.413686	-0.187203
10	1	0	3.984992	0.246352	0.146923
11	7	0	1.681327	-2.298458	0.755172
12	1	0	1.978367	-3.108739	1.302542
13	6	0	-0.152232	2.218298	1.075406
14	1	0	-0.720585	3.027475	0.603328
15	1	0	-0.281936	2.322787	2.155172
16	6	0	-0.767416	0.907138	0.644819
17	6	0	1.564069	2.301144	-0.651844
18	1	0	1.509938	3.298890	-1.094262
19	1	0	0.834818	1.676367	-1.170370
20	6	0	-0.555058	-1.333410	0.247594
21	6	0	2.956045	1.742307	-0.870629
22	1	0	3.196954	1.609008	-1.922605
23	1	0	3.705816	2.377788	-0.403752
24	6	0	0.315765	-2.564113	0.218906
25	1	0	-0.128331	-3.347780	0.828830
26	1	0	0.420778	-2.940647	-0.797681
27	6	0	2.742233	-2.048626	-0.270882
28	1	0	2.686523	-2.872241	-0.978329
29	1	0	3.698685	-2.097535	0.247571

30	6	0	2.607989	-0.743529	-1.019334
31	1	0	3.278663	-0.782204	-1.875262
32	1	0	1.597340	-0.556285	-1.376072
33	8	0	-3.945534	-0.463062	-0.520083
34	6	0	-4.777920	0.694889	-0.519685
35	1	0	-5.754768	0.357969	-0.853785
36	1	0	-4.854008	1.112511	0.485087
37	1	0	-4.390413	1.446674	-1.208504
38	1	0	1.616296	-1.506084	1.405614
39	1	0	2.415332	0.517046	0.636674

Most stable energy, Gibbs free energy (Ha), and geometry for protomer ./OMe_PyN3//2_6
E: -764.424808
G: -764.110285
Geometry:
Input orientation:

Center Number	Atomic Number	Atomic Type	Coordinates (Angstroms) X	Y	Z
1	6	0	-2.005726	1.063564	-0.109802
2	1	0	-2.562800	1.939290	-0.408285
3	6	0	-2.587110	-0.199349	-0.036278
4	6	0	-1.808242	-1.304274	0.359255
5	1	0	-2.249565	-2.289304	0.418866
6	7	0	0.028353	0.136971	0.602811
7	7	0	1.343696	2.513393	0.793719
8	1	0	1.433788	3.357304	1.342364
9	7	0	2.756024	-0.007368	0.246351
10	1	0	3.499613	0.235704	0.893675
11	7	0	1.074568	-2.848170	-0.125174
12	1	0	0.340630	-3.294829	-0.683595
13	6	0	0.048307	2.525051	0.136526
14	1	0	0.122153	2.787816	-0.926615
15	1	0	-0.600013	3.270532	0.596529
16	6	0	-0.670596	1.204338	0.216890
17	6	0	2.494384	2.422153	-0.107232
18	1	0	3.386287	2.635149	0.484167
19	1	0	2.441554	3.167540	-0.909806
20	6	0	-0.496386	-1.104990	0.665125
21	6	0	2.648098	1.058966	-0.754636
22	1	0	1.785737	0.850745	-1.396050
23	1	0	3.526587	1.082501	-1.409141
24	6	0	0.437421	-2.206415	1.066572
25	1	0	1.233071	-1.841314	1.711446
26	1	0	-0.110804	-2.988922	1.584163
27	6	0	1.884958	-1.973149	-1.037500
28	1	0	1.200793	-1.259269	-1.494212
29	1	0	2.239320	-2.641374	-1.818186
30	6	0	3.067718	-1.309034	-0.349432
31	1	0	3.450084	-1.970155	0.430848
32	1	0	3.856171	-1.209961	-1.102761
33	8	0	-3.858012	-0.455210	-0.315847
34	6	0	-4.697006	0.628649	-0.726598
35	1	0	-4.758131	1.377958	0.062843
36	1	0	-4.314438	1.074561	-1.644615
37	1	0	-5.674138	0.190715	-0.903006
38	1	0	1.065172	0.224256	0.767033
39	1	0	1.679151	-3.599244	0.221651

Most stable energy, Gibbs free energy (Ha), and geometry for protomer ./OMe_PyN3//2_2
E: -764.436807
G: -764.122247
Geometry:
Input orientation:

Center Number	Atomic Number	Atomic Type	Coordinates (Angstroms) X	Y	Z
1	6	0	2.186354	0.967612	-0.092655
2	1	0	2.719052	1.892449	0.073178
3	6	0	2.761112	-0.278448	0.148250
4	6	0	1.991632	-1.431115	-0.029790
5	1	0	2.406551	-2.404085	0.196862
6	7	0	0.154380	-0.099937	-0.754186
7	7	0	-1.294054	2.102539	-0.705293
8	1	0	-1.544703	1.258445	-1.236408
9	7	0	-3.416862	0.177917	-0.079398
10	1	0	-4.296229	0.225283	-0.574778
11	7	0	-1.617450	-2.056492	-0.581864
12	1	0	-2.222212	-2.791179	-0.955387
13	6	0	0.174986	2.298849	-0.804252
14	1	0	0.468729	3.063521	-0.088811
15	1	0	0.393869	2.652297	-1.811417
16	6	0	0.874986	0.991558	-0.537294
17	6	0	-1.794275	1.902859	0.686211
18	1	0	-1.705041	2.855571	1.204013
19	1	0	-1.139731	1.176369	1.162594
20	6	0	0.699102	-1.285745	-0.477855
21	6	0	-3.236063	1.434726	0.648731
22	1	0	-3.586443	1.361433	1.682982
23	1	0	-3.834501	2.202379	0.155414
24	6	0	-0.196776	-2.477629	-0.688873
25	1	0	-0.053479	-2.893004	-1.686002
26	1	0	-0.011483	-3.254679	0.048966
27	6	0	-2.061374	-1.711048	0.799725
28	1	0	-1.301865	-1.063889	1.232661
29	1	0	-2.107634	-2.636239	1.370049
30	6	0	-3.418279	-1.036686	0.737601
31	1	0	-4.131840	-1.738226	0.302378
32	1	0	-3.739156	-0.838383	1.765034
33	8	0	4.018758	-0.457283	0.578252
34	6	0	4.824204	0.701389	0.787191
35	1	0	4.938032	1.260873	-0.142137
36	1	0	4.386834	1.338486	1.556910
37	1	0	5.790969	0.333965	1.117975
38	1	0	-1.752424	-1.213151	-1.154955
39	1	0	-1.777158	2.898561	-1.127544

Most stable energy, Gibbs free energy (Ha), and geometry for protomer ./OMe_PyN3//2_5
E: -764.435052
G: -764.119857
Geometry:
Input orientation:

Center Number	Atomic Number	Atomic Type	Coordinates (Angstroms) X	Y	Z
1	6	0	-1.884044	1.467157	0.152563

2	1	0	-2.283896	2.453327	-0.036318
3	6	0	-2.657547	0.326618	-0.122741
4	6	0	-2.113600	-0.949741	0.053306
5	1	0	-2.656290	-1.849144	-0.195338
6	7	0	-0.156944	0.059291	0.840955
7	7	0	1.709843	1.983492	0.705272
8	1	0	2.349731	2.599275	1.194120
9	7	0	3.166718	-0.236589	-0.424061
10	1	0	4.092234	-0.671770	-0.393032
11	7	0	1.323266	-2.215745	0.852934
12	1	0	1.675760	-2.860972	1.545585
13	6	0	0.342307	2.423468	0.946741
14	1	0	0.052873	3.298596	0.357019
15	1	0	0.238069	2.683169	2.001758
16	6	0	-0.617741	1.308328	0.634810
17	6	0	2.044392	1.959885	-0.718332
18	1	0	2.206889	2.961184	-1.128353
19	1	0	1.204080	1.529955	-1.266693
20	6	0	-0.832098	-1.053247	0.540587
21	6	0	3.300081	1.151005	-0.963204
22	1	0	3.518863	1.070933	-2.025845
23	1	0	4.152438	1.597508	-0.453923
24	6	0	-0.123629	-2.360663	0.776898
25	1	0	-0.488157	-2.752571	1.728502
26	1	0	-0.441384	-3.059620	-0.002583
27	6	0	2.038283	-2.427064	-0.407759
28	1	0	1.509010	-3.125693	-1.062554
29	1	0	3.010911	-2.864980	-0.179090
30	6	0	2.253686	-1.141539	-1.182657
31	1	0	2.727901	-1.340012	-2.141509
32	1	0	1.319693	-0.611826	-1.359215
33	8	0	-3.882906	0.550713	-0.577673
34	6	0	-4.715431	-0.571395	-0.885618
35	1	0	-4.866946	-1.183745	0.003219
36	1	0	-5.660442	-0.151433	-1.214735
37	1	0	-4.270896	-1.163978	-1.685069
38	1	0	0.795757	-0.045260	1.188942
39	1	0	2.843691	-0.171913	0.547505

Most stable energy, Gibbs free energy (Ha), and geometry for protomer ./OMe_PyN3//2_4
E: -764.424808
G: -764.110294
Geometry:
Input orientation:

Center Number	Atomic Number	Atomic Type	Coordinates (Angstroms) X	Y	Z
1	6	0	-1.808528	1.304216	0.360408
2	1	0	-2.249782	2.289212	0.420946
3	6	0	-2.587778	0.199517	-0.035031
4	6	0	-2.006455	-1.063354	-0.109707
5	1	0	-2.563715	-1.938947	-0.408227
6	7	0	0.028205	-0.137183	0.601866
7	7	0	1.074412	2.848114	-0.125263
8	1	0	1.679024	3.599173	0.221573
9	7	0	2.755885	0.007059	0.245037
10	1	0	3.499584	-0.236277	0.892145
11	7	0	1.343274	-2.513696	0.791380

12	1	0	1.433264	-3.357142	1.340717
13	6	0	0.437679	2.206012	1.066572
14	1	0	-0.110314	2.988391	1.584628
15	1	0	1.233562	1.840615	1.711021
16	6	0	-0.496415	1.104777	0.665215
17	6	0	1.884747	1.973432	-1.038028
18	1	0	2.239177	2.642025	-1.818391
19	1	0	1.200589	1.259773	-1.495068
20	6	0	-0.671077	-1.204320	0.215949
21	6	0	3.067529	1.308989	-0.350303
22	1	0	3.855988	1.210158	-1.103679
23	1	0	3.449988	1.969872	0.430146
24	6	0	0.047930	-2.524977	0.134402
25	1	0	-0.600353	-3.270953	0.593695
26	1	0	0.121655	-2.786818	-0.928993
27	6	0	2.494121	-2.422371	-0.109153
28	1	0	2.441713	-3.167723	-0.911828
29	1	0	3.385912	-2.635367	0.482446
30	6	0	2.647956	-1.058986	-0.756253
31	1	0	3.526414	-1.082422	-1.410821
32	1	0	1.785602	-0.850599	-1.397610
33	8	0	-3.858948	0.455650	-0.313390
34	6	0	-4.698496	-0.628063	-0.723444
35	1	0	-4.317104	-1.073650	-1.642109
36	1	0	-4.758690	-1.377611	0.065835
37	1	0	-5.675842	-0.190024	-0.898458
38	1	0	1.065017	-0.224838	0.765537
39	1	0	0.340242	3.294779	-0.683409

Most stable energy, Gibbs free energy (Ha), and geometry for protomer ./OMe_PyN3//3_1
E: -764.879017
G: -764.548318
Geometry:
Input orientation:

Center Number	Atomic Number	Atomic Type	Coordinates (Angstroms) X	Y	Z
1	6	0	-1.981681	1.061480	-0.122846
2	1	0	-2.518519	1.929234	-0.478109
3	6	0	-2.564282	-0.196317	-0.049936
4	6	0	-1.793700	-1.270107	0.412975
5	1	0	-2.220540	-2.263028	0.465549
6	7	0	0.072856	0.184173	0.737300
7	7	0	1.340701	2.595853	0.783949
8	1	0	1.375720	1.934062	1.566741
9	7	0	2.845318	-0.061880	0.107340
10	1	0	3.722894	0.149158	0.591564
11	7	0	0.977669	-2.857853	-0.008718
12	1	0	0.212389	-3.333912	-0.497260
13	6	0	-0.015593	2.547244	0.165116
14	1	0	0.085207	2.836153	-0.881255
15	1	0	-0.629936	3.291774	0.666484
16	6	0	-0.658481	1.189741	0.278979
17	6	0	2.501283	2.385597	-0.140884
18	1	0	3.401406	2.542883	0.451503
19	1	0	2.434351	3.165342	-0.896239
20	6	0	-0.492999	-1.034402	0.782903
21	6	0	2.537903	1.047228	-0.838067

22	1	0	1.604727	0.815936	-1.348008
23	1	0	3.339422	1.075010	-1.573769
24	6	0	0.397301	-2.171121	1.196354
25	1	0	1.222762	-1.840155	1.822318
26	1	0	-0.160411	-2.935578	1.731598
27	6	0	1.694353	-2.024175	-1.025566
28	1	0	0.987921	-1.297936	-1.422037
29	1	0	1.945207	-2.711595	-1.829391
30	6	0	2.986401	-1.408375	-0.528838
31	1	0	3.473246	-2.055979	0.199042
32	1	0	3.654630	-1.275894	-1.376291
33	8	0	-3.825141	-0.470718	-0.400325
34	6	0	-4.628703	0.598728	-0.899412
35	1	0	-4.741530	1.375839	-0.142607
36	1	0	-4.186777	1.016885	-1.804547
37	1	0	-5.595273	0.160091	-1.127489
38	1	0	1.612005	-3.591516	0.322824
39	1	0	2.090751	-0.080150	0.808515
40	1	0	1.464471	3.524378	1.195160

Most stable energy, Gibbs free energy (Ha), and geometry for protomer ./OMe_PyN3//3_2
E: -764.623577
G: -764.292846
Geometry:

Input orientation:

Center	Atomic	Atomic	Coordinates (Angstroms)		
Number	Number	Type	X	Y	Z
1	6	0	-1.587682	1.399955	0.121353
2	1	0	-1.936108	2.379652	-0.187819
3	6	0	-2.313321	0.242014	-0.238310
4	6	0	-1.821602	-1.026025	0.109066
5	1	0	-2.312434	-1.943643	-0.190518
6	7	0	-0.022970	0.024988	1.207150
7	7	0	1.161853	2.978600	-0.028801
8	1	0	1.512939	3.906674	0.238928
9	7	0	2.484569	-0.280439	-0.634926
10	1	0	3.500701	-0.322048	-0.782329
11	7	0	1.463409	-2.273996	1.228880
12	1	0	1.854839	-2.860604	1.958060
13	6	0	0.470102	2.419906	1.183422
14	1	0	-0.124090	3.233526	1.600242
15	1	0	1.244470	2.130663	1.895305
16	6	0	-0.426602	1.264404	0.833190
17	6	0	2.335098	2.223940	-0.588766
18	1	0	3.015414	2.039132	0.245644
19	1	0	2.816342	2.914734	-1.279238
20	6	0	-0.654654	-1.106317	0.842310
21	6	0	1.945628	0.937457	-1.317772
22	1	0	0.863344	0.815480	-1.404339
23	1	0	2.358127	0.931780	-2.325936
24	6	0	0.006850	-2.397447	1.256129
25	1	0	-0.311199	-2.619458	2.279691
26	1	0	-0.371421	-3.196816	0.606880
27	6	0	2.071124	-2.647409	-0.053554
28	1	0	1.650101	-3.576648	-0.459278
29	1	0	3.140108	-2.805375	0.112872
30	6	0	1.873094	-1.567631	-1.104537

31	1	0	2.352461	-1.837362	-2.045820
32	1	0	0.814957	-1.373142	-1.297143
33	8	0	-3.426497	0.446779	-0.925827
34	6	0	-4.209006	-0.689160	-1.330874
35	1	0	-3.625083	-1.326036	-1.999087
36	1	0	-4.538297	-1.247957	-0.451930
37	1	0	-5.064913	-0.273997	-1.857732
38	1	0	0.814553	-0.081556	1.784077
39	1	0	2.359889	-0.219846	0.383707
40	1	0	0.474019	3.149727	-0.775344

Most stable energy, Gibbs free energy (Ha), and geometry for protomer ./OMe_PyN3//3_3
E: -764.866158
G: -764.538443
Geometry:
Input orientation:

Center Number	Atomic Number	Atomic Type	Coordinates (Angstroms) X	Y	Z
1	6	0	-2.032712	0.995122	0.110240
2	1	0	-2.604044	1.899752	-0.031505
3	6	0	-2.606573	-0.274736	-0.026263
4	6	0	-1.818409	-1.412734	0.200546
5	1	0	-2.252881	-2.399920	0.127032
6	7	0	0.025821	-0.018921	0.644523
7	7	0	0.959655	2.787852	-0.383511
8	1	0	0.875576	3.800093	-0.506934
9	7	0	2.656280	0.079269	0.444528
10	1	0	3.206167	0.303434	1.269994
11	7	0	1.186673	-2.863593	-0.348297
12	1	0	0.535794	-3.226806	-1.051269
13	6	0	-0.037313	2.410930	0.675594
14	1	0	-0.797609	3.185801	0.681018
15	1	0	0.485297	2.417306	1.630156
16	6	0	-0.702308	1.084692	0.447634
17	6	0	2.417942	2.506264	-0.124065
18	1	0	2.597700	2.696106	0.933028
19	1	0	2.954360	3.250132	-0.707268
20	6	0	-0.501775	-1.250253	0.530279
21	6	0	2.881644	1.132260	-0.556837
22	1	0	2.378454	0.854989	-1.486843
23	1	0	3.948504	1.206535	-0.782081
24	6	0	0.407948	-2.401781	0.839663
25	1	0	1.118110	-2.135100	1.619048
26	1	0	-0.178452	-3.251905	1.177155
27	6	0	2.085954	-1.865336	-1.016630
28	1	0	1.441449	-1.132555	-1.499813
29	1	0	2.597147	-2.424810	-1.795089
30	6	0	3.095562	-1.231279	-0.067651
31	1	0	3.290162	-1.892715	0.777473
32	1	0	4.037329	-1.119561	-0.609828
33	8	0	-3.870951	-0.486931	-0.348789
34	6	0	-4.721176	0.642327	-0.582589
35	1	0	-4.342566	1.230195	-1.418657
36	1	0	-5.693656	0.227514	-0.826427
37	1	0	-4.787790	1.254956	0.316303
38	1	0	1.128819	0.057557	0.746049
39	1	0	1.758732	-3.656348	-0.040569

40	1	0	0.688655	2.375352	-1.283000

Most stable energy, Gibbs free energy (Ha), and geometry for protomer ./OMe_PyN3//3_4
E: -764.870696
G: -764.539081
Geometry:

Input orientation:

Center Number	Atomic Number	Atomic Type	Coordinates (Angstroms) X	Y	Z
1	6	0	-1.939411	0.981833	0.243671
2	1	0	-2.415221	1.931582	0.051429
3	6	0	-2.508477	-0.232422	-0.152153
4	6	0	-1.803496	-1.430569	0.066837
5	1	0	-2.213885	-2.371720	-0.270770
6	7	0	-0.107692	-0.198436	1.116105
7	7	0	1.401736	2.148232	0.814310
8	1	0	1.939852	2.871947	1.278422
9	7	0	3.094294	0.392000	-0.506770
10	1	0	4.081338	0.140002	-0.602711
11	7	0	1.240397	-2.840553	-0.163273
12	1	0	0.774334	-2.810984	-1.078256
13	6	0	0.018414	2.222010	1.270009
14	1	0	-0.529572	3.076650	0.864252
15	1	0	0.011419	2.300116	2.358658
16	6	0	-0.717449	0.972838	0.872024
17	6	0	1.526724	2.290302	-0.637743
18	1	0	1.450528	3.328328	-0.971762
19	1	0	0.709726	1.745009	-1.113386
20	6	0	-0.597202	-1.388065	0.698534
21	6	0	2.858682	1.739017	-1.112497
22	1	0	2.893774	1.639505	-2.194714
23	1	0	3.684355	2.363553	-0.776798
24	6	0	0.230159	-2.615170	0.925202
25	1	0	0.778700	-2.562251	1.863517
26	1	0	-0.417913	-3.486687	0.930143
27	6	0	2.435175	-1.936160	-0.152670
28	1	0	3.284960	-2.525598	-0.485067
29	1	0	2.596981	-1.653945	0.888218
30	6	0	2.251947	-0.711303	-1.043193
31	1	0	2.572330	-0.905750	-2.063517
32	1	0	1.221059	-0.365642	-1.073622
33	8	0	-3.673126	-0.343617	-0.759656
34	6	0	-4.430585	0.844825	-1.023714
35	1	0	-3.873294	1.502891	-1.689922
36	1	0	-5.341511	0.506404	-1.505947
37	1	0	-4.664078	1.354833	-0.089605
38	1	0	0.804729	-0.167481	1.573213
39	1	0	1.576146	-3.801739	-0.049923
40	1	0	2.894241	0.502684	0.496803

Most stable energy, Gibbs free energy (Ha), and geometry for protomer ./metaOH_PyN3//0_1
E: -723.743623
G: -723.497276
Geometry:

Input orientation:

Center Number	Atomic Number	Atomic Type	Coordinates (Angstroms) X	Y	Z

1	6	0	-0.736491	1.497656	0.522560
2	6	0	-0.245550	2.495027	-0.304473
3	6	0	1.911065	1.377731	-0.366987
4	6	0	1.292264	0.386955	0.461443
5	7	0	0.050379	0.483809	0.910095
6	1	0	-0.898388	3.301454	-0.619348
7	6	0	2.108638	-0.824632	0.860771
8	1	0	2.801823	-1.055042	0.050679
9	1	0	2.734965	-0.547024	1.716010
10	6	0	-2.176598	1.478239	0.980564
11	1	0	-2.710251	2.283878	0.471828
12	1	0	-2.218810	1.696893	2.050848
13	7	0	1.366733	-2.039402	1.186946
14	1	0	0.525718	-1.773553	1.689450
15	7	0	-2.922906	0.237248	0.774525
16	1	0	-2.504950	-0.494592	1.338347
17	6	0	1.002212	-2.844642	0.016593
18	1	0	0.486428	-3.737261	0.377055
19	1	0	1.927614	-3.178968	-0.456788
20	6	0	0.142324	-2.172745	-1.045413
21	1	0	0.670652	-1.289003	-1.433529
22	1	0	0.024125	-2.860814	-1.886808
23	6	0	-3.053619	-0.216113	-0.611032
24	1	0	-3.818478	-0.995901	-0.624674
25	1	0	-3.439998	0.619515	-1.199509
26	6	0	-1.809553	-0.763850	-1.309414
27	1	0	-1.101511	0.046696	-1.527939
28	1	0	-2.120254	-1.177771	-2.272765
29	7	0	-1.171905	-1.812608	-0.519639
30	1	0	-0.989255	-1.385936	0.384971
31	6	0	1.071278	2.445343	-0.738155
32	1	0	1.472461	3.219439	-1.383885
33	8	0	3.150710	1.279736	-0.742357

Most stable energy, Gibbs free energy (Ha), and geometry for protomer ./metaOH_PyN3//1_3
E: -724.211952
G: -723.951771
Geometry:
Input orientation:

Center Number	Atomic Number	Atomic Type	Coordinates (Angstroms) X	Y	Z
1	6	0	0.038462	-1.716643	-0.474049
2	6	0	0.877666	-2.338547	0.446158
3	6	0	2.378642	-0.419283	0.383187
4	6	0	1.437839	0.118373	-0.539987
5	7	0	0.350078	-0.516816	-0.957933
6	1	0	0.608255	-3.309868	0.845234
7	6	0	1.573381	1.531198	-1.037613
8	1	0	2.319600	2.089334	-0.479162
9	1	0	1.807097	1.577064	-2.100686
10	6	0	-1.271955	-2.314002	-0.926782
11	1	0	-1.480506	-3.206316	-0.334639
12	1	0	-1.176839	-2.637521	-1.966553
13	7	0	0.250734	2.200306	-0.848984
14	1	0	0.192813	3.055260	-1.403924
15	7	0	-2.431440	-1.423999	-0.851956

16	1	0	-2.206654	-0.585158	-1.379504
17	6	0	-0.069137	2.497756	0.577535
18	1	0	0.409292	3.437454	0.842012
19	1	0	0.372414	1.700174	1.172735
20	6	0	-1.573322	2.563673	0.768131
21	1	0	-1.760475	2.719248	1.837905
22	1	0	-1.968488	3.428166	0.232848
23	6	0	-2.796593	-1.008678	0.504988
24	1	0	-3.862075	-0.758458	0.509794
25	1	0	-2.673146	-1.864264	1.171890
26	6	0	-2.050511	0.184867	1.091808
27	1	0	-0.985956	-0.031848	1.185095
28	1	0	-2.433907	0.362424	2.106596
29	7	0	-2.222471	1.366452	0.245977
30	1	0	-3.215786	1.552616	0.155675
31	1	0	-0.473926	1.545468	-1.181817
32	6	0	2.035657	-1.707989	0.860933
33	1	0	2.691651	-2.176964	1.586189
34	8	0	3.433856	0.223674	0.761717

Most stable energy, Gibbs free energy (Ha), and geometry for protomer ./metaOH_PyN3//1_2

E: -724.214941

G: -723.954608

Geometry:

Input orientation:

Center Number	Atomic Number	Atomic Type	Coordinates (Angstroms) X	Y	Z
1	6	0	0.353407	-1.572165	-0.293734
2	6	0	1.299837	-2.064813	0.590689
3	6	0	2.312751	0.139770	0.739928
4	6	0	1.267542	0.552042	-0.144839
5	7	0	0.368843	-0.279830	-0.650706
6	1	0	1.264957	-3.106859	0.887138
7	6	0	1.213289	2.014900	-0.529326
8	1	0	1.374639	2.616604	0.368190
9	1	0	2.073372	2.222508	-1.174628
10	6	0	-0.705417	-2.474804	-0.884706
11	1	0	-0.770531	-3.374495	-0.259575
12	1	0	-0.385461	-2.799882	-1.877985
13	7	0	-0.003078	2.483694	-1.181452
14	1	0	-0.345322	1.764170	-1.809408
15	7	0	-2.009587	-1.836104	-1.050116
16	1	0	-2.555518	-2.398722	-1.689587
17	6	0	-1.061416	2.896273	-0.265369
18	1	0	-1.921797	3.211761	-0.858371
19	1	0	-0.717367	3.770514	0.289577
20	6	0	-1.521538	1.878848	0.769223
21	1	0	-0.689718	1.515720	1.372441
22	1	0	-2.268923	2.312737	1.431337
23	6	0	-2.779877	-1.675136	0.179403
24	1	0	-3.822487	-1.525198	-0.102569
25	1	0	-2.733682	-2.558246	0.828521
26	6	0	-2.319938	-0.480090	1.001099
27	1	0	-1.360304	-0.659411	1.483604
28	1	0	-3.053286	-0.222083	1.762374
29	7	0	-2.127581	0.692835	0.110953
30	1	0	-1.474564	0.366264	-0.626742

31	1	0	-3.012996	0.949058	-0.331975
32	6	0	2.277630	-1.222410	1.097456
33	1	0	3.024868	-1.596103	1.789264
34	8	0	3.200189	0.976424	1.177553

Most stable energy, Gibbs free energy (Ha), and geometry for protomer ./metaOH_PyN3//1_1
E: -724.217456
G: -723.958262
Geometry:
Input orientation:

Center Number	Atomic Number	Atomic Type	Coordinates (Angstroms) X	Y	Z
1	6	0	-0.603715	-1.539727	0.465744
2	6	0	-0.003509	-2.486463	-0.351503
3	6	0	1.981349	-1.171751	-0.315009
4	6	0	1.312065	-0.248518	0.494690
5	7	0	0.065480	-0.467473	0.897213
6	1	0	-0.566198	-3.345872	-0.692471
7	6	0	2.008272	1.018892	0.948336
8	1	0	2.539623	0.786268	1.876342
9	1	0	2.772081	1.283590	0.217208
10	6	0	-2.047896	-1.682615	0.898129
11	1	0	-2.058450	-2.038711	1.931489
12	1	0	-2.513484	-2.460796	0.291002
13	7	0	1.166175	2.186303	1.165748
14	1	0	0.341422	1.907735	1.686846
15	7	0	-2.883722	-0.490864	0.835303
16	1	0	-2.484146	0.229482	1.426584
17	6	0	0.774614	2.891677	-0.057985
18	1	0	1.689092	3.224119	-0.553288
19	1	0	0.227652	3.787892	0.241485
20	6	0	-0.062318	2.121261	-1.070078
21	1	0	-0.222936	2.758472	-1.943724
22	1	0	0.502280	1.245045	-1.423117
23	6	0	-3.147298	0.037909	-0.503706
24	1	0	-3.561998	-0.774545	-1.104524
25	1	0	-3.928956	0.794121	-0.404859
26	6	0	-1.980245	0.654218	-1.269408
27	1	0	-2.366555	1.054771	-2.210576
28	1	0	-1.248965	-0.121181	-1.537176
29	7	0	-1.351972	1.729969	-0.510186
30	1	0	-1.144999	1.334418	0.402597
31	6	0	1.315318	-2.307907	-0.737424
32	1	0	1.820407	-3.024502	-1.375231
33	8	0	3.276131	-0.899865	-0.661564
34	1	0	3.611700	-1.598596	-1.239303

Most stable energy, Gibbs free energy (Ha), and geometry for protomer ./metaOH_PyN3//1_4
E: -724.211872
G: -723.951266
Geometry:
Input orientation:

Center Number	Atomic Number	Atomic Type	Coordinates (Angstroms) X	Y	Z
1	6	0	0.006171	-1.770833	-0.422804
2	6	0	0.902799	-2.600296	0.223439

3	6	0	2.501703	-0.765867	0.285965
4	6	0	1.475543	0.006320	-0.351432
5	7	0	0.311296	-0.500331	-0.708923
6	1	0	0.624208	-3.619183	0.465610
7	6	0	1.752700	1.462601	-0.639117
8	1	0	2.195225	1.913772	0.251929
9	1	0	2.540607	1.502680	-1.400107
10	6	0	-1.381278	-2.204617	-0.808420
11	1	0	-1.729098	-3.039988	-0.204168
12	1	0	-1.455319	-2.473677	-1.862250
13	7	0	0.615406	2.273365	-1.066227
14	1	0	-0.045675	1.654884	-1.528284
15	7	0	-2.305851	-1.054449	-0.599666
16	1	0	-3.201953	-1.227027	-1.057144
17	6	0	-0.092412	2.930297	0.035145
18	1	0	-0.713257	3.729236	-0.381832
19	1	0	0.649620	3.413931	0.671795
20	6	0	-0.987031	2.051613	0.897694
21	1	0	-0.441703	1.160782	1.214564
22	1	0	-1.261904	2.604875	1.806305
23	6	0	-2.528967	-0.684608	0.825730
24	1	0	-3.231049	-1.394100	1.256936
25	1	0	-1.570731	-0.780131	1.332601
26	6	0	-3.053105	0.738900	0.889630
27	1	0	-3.156923	1.011376	1.946795
28	1	0	-4.045933	0.775375	0.438834
29	7	0	-2.178960	1.643961	0.151916
30	1	0	-2.702500	2.480192	-0.082312
31	1	0	-1.871982	-0.223780	-1.032539
32	6	0	2.153900	-2.104297	0.561596
33	1	0	2.878885	-2.733680	1.065782
34	8	0	3.645329	-0.244537	0.591167

Most stable energy, Gibbs free energy (Ha), and geometry for protomer ./metaOH_PyN3//1_5
E: -724.212187
G: -723.953252
Geometry:

Input orientation:

Center Number	Atomic Number	Atomic Type	Coordinates (Angstroms) X	Y	Z
1	6	0	-0.435514	1.612539	-0.312120
2	6	0	0.371515	2.628686	0.167473
3	6	0	2.321442	1.155702	0.171720
4	6	0	1.441731	0.149319	-0.306820
5	7	0	0.151629	0.425061	-0.528615
6	1	0	-0.071601	3.599492	0.349944
7	6	0	1.964163	-1.230100	-0.581052
8	1	0	2.747615	-1.129769	-1.336281
9	1	0	2.471319	-1.581982	0.327831
10	6	0	-1.886978	1.852158	-0.678968
11	1	0	-1.873872	2.432046	-1.605966
12	1	0	-2.308227	2.508836	0.084377
13	7	0	0.955807	-2.169261	-1.047792
14	1	0	1.371766	-2.752707	-1.760984
15	7	0	-2.786489	0.731351	-0.858356
16	1	0	-2.397669	0.043671	-1.493472
17	6	0	0.430796	-3.039835	0.007186

18	1	0	1.239704	-3.544144	0.551053
19	1	0	-0.178174	-3.810647	-0.468891
20	6	0	-0.416659	-2.283047	1.012551
21	1	0	-0.701058	-2.965771	1.822646
22	1	0	0.180816	-1.490551	1.474410
23	6	0	-3.270526	0.086321	0.358390
24	1	0	-3.741075	0.849147	0.981769
25	1	0	-4.054530	-0.615699	0.064869
26	6	0	-2.239359	-0.664026	1.189489
27	1	0	-2.737489	-1.097384	2.067169
28	1	0	-1.483338	0.028553	1.570208
29	7	0	-1.566408	-1.661531	0.370534
30	1	0	-2.229700	-2.378149	0.091729
31	1	0	-0.439817	-0.385896	-0.774189
32	6	0	1.714934	2.411858	0.406710
33	1	0	2.337298	3.214754	0.784940
34	8	0	3.565960	0.906287	0.371437

Most stable energy, Gibbs free energy (Ha), and geometry for protomer ./metaOH_PyN3//2_7
E: -724.675486
G: -724.405312
Geometry:
Input orientation:

Center Number	Atomic Number	Atomic Type	Coordinates (Angstroms) X	Y	Z
1	6	0	0.281106	-1.668389	0.093211
2	6	0	1.468111	-2.270615	-0.281363
3	6	0	2.547752	-0.138907	-0.237250
4	6	0	1.347556	0.445157	0.149104
5	7	0	0.272579	-0.342976	0.299557
6	1	0	1.489080	-3.340289	-0.437897
7	6	0	1.280635	1.927883	0.393700
8	1	0	2.131999	2.170873	1.031103
9	1	0	1.472672	2.427771	-0.566130
10	6	0	-0.979955	-2.481016	0.252487
11	1	0	-0.699182	-3.376431	0.807186
12	1	0	-1.276364	-2.821240	-0.748312
13	7	0	0.058751	2.391189	1.025041
14	1	0	0.309167	3.087720	1.713843
15	7	0	-2.066820	-1.803892	0.937830
16	1	0	-2.472348	-2.447775	1.603363
17	6	0	-0.903517	2.991923	0.099722
18	1	0	-0.443616	3.774856	-0.515616
19	1	0	-1.687620	3.457992	0.698720
20	6	0	-1.528712	1.965108	-0.823917
21	1	0	-2.258654	2.461921	-1.473197
22	1	0	-0.766453	1.532900	-1.482132
23	6	0	-3.130178	-1.332981	0.048419
24	1	0	-3.517443	-2.138608	-0.587364
25	1	0	-3.951714	-0.980051	0.674224
26	6	0	-2.665702	-0.201710	-0.847918
27	1	0	-3.500018	0.123445	-1.479881
28	1	0	-1.878699	-0.553435	-1.524425
29	7	0	-2.110542	0.879901	-0.048759
30	1	0	-2.811637	1.240802	0.591748
31	1	0	-0.675646	0.136157	0.480780
32	6	0	2.606764	-1.507298	-0.450645

33	1	0	3.546231	-1.962598	-0.741041
34	8	0	3.611087	0.689019	-0.378861
35	1	0	4.395093	0.183303	-0.634140

Most stable energy, Gibbs free energy (Ha), and geometry for protomer ./metaOH_PyN3//2_1
E: -724.685855
G: -724.413198
Geometry:
Input orientation:

Center Number	Atomic Number	Atomic Type	Coordinates (Angstroms) X	Y	Z
1	6	0	0.275606	-1.604445	-0.307793
2	6	0	1.291367	-2.159272	0.457041
3	6	0	2.375605	-0.038406	0.456868
4	6	0	1.308979	0.465892	-0.290600
5	7	0	0.310234	-0.320834	-0.675724
6	1	0	1.239505	-3.197678	0.757425
7	6	0	1.307035	1.928284	-0.686895
8	1	0	1.600847	2.524619	0.179566
9	1	0	2.104466	2.062800	-1.424022
10	6	0	-0.881269	-2.468269	-0.764590
11	1	0	-1.004519	-3.278661	-0.035575
12	1	0	-0.594654	-2.934717	-1.709879
13	7	0	0.066392	2.467758	-1.215455
14	1	0	-0.361182	1.799479	-1.846910
15	7	0	-2.127688	-1.748500	-0.992709
16	1	0	-2.686556	-2.281772	-1.645325
17	6	0	-0.890878	2.919709	-0.212007
18	1	0	-1.762021	3.321508	-0.732313
19	1	0	-0.441677	3.743814	0.344255
20	6	0	-1.359841	1.903245	0.818900
21	1	0	-0.523721	1.449682	1.350573
22	1	0	-2.020281	2.372354	1.546317
23	6	0	-2.927980	-1.514533	0.205815
24	1	0	-3.939922	-1.263823	-0.113763
25	1	0	-2.989137	-2.397741	0.852786
26	6	0	-2.376775	-0.373483	1.042642
27	1	0	-1.426815	-0.633491	1.508409
28	1	0	-3.078051	-0.077598	1.819732
29	7	0	-2.113209	0.799110	0.168921
30	1	0	-1.540519	0.431989	-0.611368
31	1	0	-2.990876	1.156040	-0.217367
32	6	0	2.363394	-1.368260	0.835972
33	1	0	3.176420	-1.766709	1.432146
34	8	0	3.378682	0.824555	0.790579
35	1	0	4.039385	0.365157	1.326911

Most stable energy, Gibbs free energy (Ha), and geometry for protomer ./metaOH_PyN3//2_3
E: -724.673711
G: -724.397540
Geometry:
Input orientation:

Center Number	Atomic Number	Atomic Type	Coordinates (Angstroms) X	Y	Z
1	6	0	0.096180	-1.797698	-0.194708
2	6	0	1.019582	-2.155997	0.787234

3	6	0	2.139939	-0.024252	0.515310
4	6	0	1.181558	0.209941	-0.503262
5	7	0	0.212319	-0.639176	-0.836226
6	1	0	0.909032	-3.105140	1.298859
7	6	0	1.165313	1.563930	-1.135151
8	1	0	2.120277	1.820230	-1.592401
9	1	0	0.379569	1.668065	-1.879668
10	6	0	-1.092834	-2.671048	-0.534188
11	1	0	-1.308712	-3.313012	0.321736
12	1	0	-0.825332	-3.332419	-1.362123
13	7	0	0.948543	2.589788	-0.060263
14	1	0	1.789308	2.519858	0.535075
15	7	0	-2.319055	-1.979020	-0.908089
16	1	0	-2.149076	-1.395745	-1.719718
17	6	0	-0.241605	2.391453	0.820606
18	1	0	-0.252042	3.244365	1.494634
19	1	0	-0.062155	1.499406	1.416369
20	6	0	-1.569895	2.357027	0.088957
21	1	0	-2.341084	2.719812	0.764543
22	1	0	-1.551645	3.002182	-0.788817
23	6	0	-2.992345	-1.225889	0.140813
24	1	0	-3.969606	-0.925106	-0.242502
25	1	0	-3.172652	-1.896113	0.982925
26	6	0	-2.322972	0.021898	0.718387
27	1	0	-1.389369	-0.202961	1.228428
28	1	0	-3.001934	0.507339	1.418094
29	7	0	-2.026302	1.005917	-0.364741
30	1	0	-1.326838	0.553785	-0.980752
31	1	0	0.913984	3.521223	-0.479897
32	1	0	-2.877210	1.143585	-0.917888
33	6	0	2.039458	-1.292304	1.132842
34	1	0	2.744591	-1.549324	1.915223
35	8	0	2.987358	0.894653	0.857236

Most stable energy, Gibbs free energy (Ha), and geometry for protomer ./metaOH_PyN3//2_10
E: -724.672622
G: -724.398059
Geometry:
Input orientation:

Center Number	Atomic Number	Atomic Type	Coordinates (Angstroms) X	Y	Z
1	6	0	0.133787	-1.534056	0.554605
2	6	0	1.133019	-2.353163	0.071564
3	6	0	2.559045	-0.410810	-0.368373
4	6	0	1.486595	0.376629	0.159859
5	7	0	0.379339	-0.209994	0.583775
6	1	0	0.969469	-3.422962	0.049658
7	6	0	1.582909	1.869494	0.239880
8	1	0	2.504914	2.106388	0.774612
9	1	0	1.719312	2.253098	-0.780817
10	6	0	-1.208327	-1.991234	1.013839
11	1	0	-1.666726	-1.283903	1.700559
12	1	0	-1.137320	-2.961043	1.499870
13	7	0	0.449571	2.481502	0.917875
14	1	0	0.794664	3.233879	1.497856
15	7	0	-2.160249	-2.174338	-0.135216
16	1	0	-3.059247	-2.479099	0.249903

17	6	0	-0.577643	3.027613	0.028380
18	1	0	-0.139979	3.664722	-0.749768
19	1	0	-1.234260	3.653102	0.635954
20	6	0	-1.416297	1.967265	-0.661377
21	1	0	-2.129862	2.472136	-1.323710
22	1	0	-0.780815	1.348903	-1.302971
23	6	0	-2.395033	-0.994236	-1.030254
24	1	0	-1.437287	-0.741688	-1.482390
25	1	0	-3.048927	-1.364318	-1.816200
26	6	0	-3.048553	0.188053	-0.332684
27	1	0	-3.745861	-0.178719	0.423199
28	1	0	-3.643197	0.708008	-1.092444
29	7	0	-2.102735	1.105161	0.304499
30	1	0	-2.621252	1.700353	0.942999
31	1	0	-0.400983	0.405510	0.883812
32	1	0	-1.812050	-2.942388	-0.716000
33	6	0	2.323675	-1.807284	-0.377370
34	1	0	3.108917	-2.451936	-0.753461
35	8	0	3.627372	0.145948	-0.784542

Most stable energy, Gibbs free energy (Ha), and geometry for protomer ./metaOH_PyN3//2_2
E: -724.680447
G: -724.407643
Geometry:
Input orientation:

Center Number	Atomic Number	Atomic Type	Coordinates (Angstroms) X	Y	Z
1	6	0	0.506692	-1.654546	-0.270111
2	6	0	1.572933	-2.041792	0.536718
3	6	0	2.489346	0.149373	0.295492
4	6	0	1.385644	0.465195	-0.488058
5	7	0	0.446393	-0.428286	-0.776699
6	1	0	1.605327	-3.041997	0.949141
7	6	0	1.153832	1.855067	-1.018259
8	1	0	1.693418	2.607321	-0.448958
9	1	0	1.433515	1.931785	-2.068330
10	6	0	-0.605936	-2.613604	-0.635552
11	1	0	-0.675058	-3.384821	0.132509
12	1	0	-0.303822	-3.120856	-1.556609
13	7	0	-0.302013	2.142817	-0.914039
14	1	0	-0.551485	2.938889	-1.502961
15	7	0	-1.927303	-2.038442	-0.844209
16	1	0	-1.825878	-1.214415	-1.429682
17	6	0	-0.769955	2.381348	0.483349
18	1	0	-0.528337	3.407358	0.749170
19	1	0	-0.205134	1.709604	1.127568
20	6	0	-2.260494	2.112897	0.570339
21	1	0	-2.560124	2.250329	1.616432
22	1	0	-2.794573	2.852357	-0.028025
23	6	0	-2.631308	-1.645079	0.377813
24	1	0	-3.703656	-1.625437	0.160348
25	1	0	-2.478150	-2.425363	1.125083
26	6	0	-2.266444	-0.298023	0.986361
27	1	0	-1.204328	-0.264132	1.231413
28	1	0	-2.817617	-0.183628	1.930178
29	7	0	-2.580212	0.786694	0.055742
30	1	0	-3.572920	0.750247	-0.149806

31	1	0	-0.816134	1.313184	-1.251057
32	6	0	2.580140	-1.135833	0.813486
33	1	0	3.422652	-1.401382	1.441575
34	8	0	3.416828	1.117894	0.530053
35	1	0	4.110363	0.773382	1.109083

Most stable energy, Gibbs free energy (Ha), and geometry for protomer ./metaOH_PyN3//2_5
E: -724.672674
G: -724.396325
Geometry:
Input orientation:

Center Number	Atomic Number	Atomic Type	Coordinates (Angstroms) X	Y	Z
1	6	0	0.384199	-1.556995	0.472724
2	6	0	1.481510	-2.191311	-0.083391
3	6	0	2.514381	-0.021623	-0.462572
4	6	0	1.331175	0.531172	0.137530
5	7	0	0.341800	-0.218996	0.579963
6	1	0	1.501085	-3.272772	-0.152277
7	6	0	1.250907	2.032634	0.253679
8	1	0	2.139414	2.366764	0.794054
9	1	0	1.345652	2.458800	-0.755339
10	6	0	-0.828849	-2.294577	0.932772
11	1	0	-1.355266	-1.754802	1.717431
12	1	0	-0.588689	-3.292233	1.292729
13	7	0	0.066761	2.539502	0.940877
14	1	0	0.342343	3.342962	1.489527
15	7	0	-1.818324	-2.505478	-0.188827
16	1	0	-2.645292	-2.969913	0.198311
17	6	0	-1.011781	2.969334	0.056511
18	1	0	-0.660511	3.631371	-0.744645
19	1	0	-1.732744	3.527305	0.655968
20	6	0	-1.724857	1.811327	-0.611781
21	1	0	-2.601272	2.161734	-1.153671
22	1	0	-1.067537	1.287620	-1.305539
23	6	0	-2.259354	-1.297539	-0.952895
24	1	0	-1.380517	-0.883103	-1.440912
25	1	0	-2.925967	-1.672818	-1.724877
26	6	0	-3.011129	-0.282264	-0.108607
27	1	0	-3.487049	-0.764065	0.744667
28	1	0	-3.789688	0.171807	-0.717107
29	7	0	-2.165979	0.826616	0.412978
30	1	0	-2.682098	1.316781	1.146170
31	1	0	-1.400856	-3.155422	-0.860621
32	1	0	-1.260415	0.449646	0.813831
33	6	0	2.543360	-1.430767	-0.544104
34	1	0	3.416461	-1.904891	-0.977630
35	8	0	3.462618	0.739404	-0.881454

Most stable energy, Gibbs free energy (Ha), and geometry for protomer ./metaOH_PyN3//2_8
E: -724.678200
G: -724.403516
Geometry:
Input orientation:

Center Number	Atomic Number	Atomic Type	Coordinates (Angstroms) X	Y	Z

1	6	0	0.417325	-1.782566	-0.379958
2	6	0	1.505144	-2.369994	0.234150
3	6	0	2.720623	-0.235754	0.223041
4	6	0	1.553531	0.313408	-0.375047
5	7	0	0.523064	-0.476776	-0.662482
6	1	0	1.456426	-3.418715	0.496114
7	6	0	1.431199	1.773911	-0.692296
8	1	0	1.848661	2.331300	0.153503
9	1	0	2.074149	1.991614	-1.548831
10	6	0	-0.877974	-2.457096	-0.739713
11	1	0	-1.008130	-3.317994	-0.075203
12	1	0	-0.823870	-2.836608	-1.761667
13	7	0	0.063845	2.186073	-0.999030
14	1	0	0.089401	2.846190	-1.763182
15	7	0	-1.982704	-1.505333	-0.666773
16	1	0	-2.775900	-1.876149	-1.177710
17	6	0	-0.657093	2.791820	0.122256
18	1	0	-1.366349	3.517904	-0.277843
19	1	0	0.016974	3.329405	0.796056
20	6	0	-1.415921	1.773940	0.950419
21	1	0	-0.774240	0.955518	1.271850
22	1	0	-1.857924	2.237087	1.829869
23	6	0	-2.383774	-1.214113	0.707957
24	1	0	-2.951051	-2.031040	1.164471
25	1	0	-1.484196	-1.080487	1.311464
26	6	0	-3.241802	0.031930	0.764035
27	1	0	-3.490494	0.298044	1.789090
28	1	0	-4.160253	-0.106199	0.195855
29	7	0	-2.539264	1.200202	0.151398
30	1	0	-2.183929	0.911372	-0.767181
31	1	0	-0.280917	-0.024540	-1.101577
32	1	0	-3.218926	1.944936	-0.022115
33	6	0	2.633602	-1.619436	0.518882
34	1	0	3.483625	-2.085265	1.003651
35	8	0	3.741102	0.495977	0.474972

Most stable energy, Gibbs free energy (Ha), and geometry for protomer ./metaOH_PyN3//2_4
E: -724.681675
G: -724.409471
Geometry:
Input orientation:

Center Number	Atomic Number	Atomic Type	Coordinates (Angstroms) X	Y	Z
1	6	0	0.520437	1.677784	-0.395279
2	6	0	1.615423	2.220257	0.249293
3	6	0	2.539826	0.022847	0.334578
4	6	0	1.386443	-0.455036	-0.300804
5	7	0	0.428436	0.377165	-0.672995
6	1	0	1.654688	3.277501	0.475237
7	6	0	1.243336	-1.930285	-0.595100
8	1	0	1.996805	-2.175490	-1.351296
9	1	0	1.529239	-2.492962	0.295619
10	6	0	-0.660249	2.513165	-0.815231
11	1	0	-0.579500	2.817961	-1.858186
12	1	0	-0.770468	3.398086	-0.192498
13	7	0	-0.066390	-2.382710	-1.040485
14	1	0	-0.506539	-1.628038	-1.558859

15	7	0	-1.891381	1.689972	-0.691180
16	1	0	-1.708752	0.769548	-1.121609
17	6	0	-0.970231	-2.776250	0.042472
18	1	0	-0.416633	-3.424814	0.722563
19	1	0	-1.771778	-3.384642	-0.386722
20	6	0	-1.616687	-1.656334	0.843528
21	1	0	-2.094983	-2.088954	1.732992
22	1	0	-0.855331	-0.958320	1.196536
23	6	0	-2.299237	1.397547	0.712855
24	1	0	-1.388000	1.209400	1.277849
25	1	0	-2.789371	2.280520	1.115271
26	6	0	-3.216198	0.188419	0.715877
27	1	0	-4.142352	0.439992	0.196980
28	1	0	-3.468417	-0.038842	1.758454
29	7	0	-2.589735	-0.932517	0.024121
30	1	0	-3.313389	-1.586516	-0.253691
31	1	0	-2.666004	2.131073	-1.189813
32	6	0	2.654767	1.372810	0.608830
33	1	0	3.536381	1.747920	1.115451
34	8	0	3.494736	-0.890911	0.663813
35	1	0	4.232582	-0.450036	1.106579

Most stable energy, Gibbs free energy (Ha), and geometry for protomer ./metaOH_PyN3//2_9
E: -724.673181
G: -724.398094
Geometry:
Input orientation:

Center Number	Atomic Number	Atomic Type	Coordinates (Angstroms) X	Y	Z
1	6	0	-1.065942	1.579793	-0.089816
2	6	0	-0.401130	2.600295	0.585628
3	6	0	1.762029	1.612804	0.001313
4	6	0	1.021554	0.646932	-0.711043
5	7	0	-0.317631	0.685498	-0.719381
6	1	0	-0.984769	3.349173	1.104998
7	6	0	1.700216	-0.514946	-1.341653
8	1	0	1.011569	-1.160835	-1.877596
9	1	0	2.493577	-0.187172	-2.011498
10	6	0	-2.555653	1.361322	-0.112310
11	1	0	-3.013043	2.124248	-0.744143
12	1	0	-2.932464	1.518383	0.905915
13	7	0	2.367154	-1.324990	-0.272066
14	1	0	2.849843	-2.112503	-0.712025
15	7	0	-2.894347	0.046422	-0.637133
16	1	0	-3.786845	0.109809	-1.107552
17	6	0	1.480587	-1.842666	0.819715
18	1	0	1.091969	-0.976854	1.353622
19	1	0	2.142680	-2.383044	1.492141
20	6	0	0.378088	-2.769604	0.335741
21	1	0	0.772541	-3.414856	-0.452292
22	1	0	0.130034	-3.416945	1.186271
23	6	0	-2.979238	-1.013194	0.373745
24	1	0	-3.568121	-0.687414	1.239900
25	1	0	-3.505395	-1.852917	-0.084183
26	6	0	-1.638392	-1.511993	0.891417
27	1	0	-1.844941	-2.247941	1.679755
28	1	0	-1.090659	-0.695149	1.370011

29	7	0	-0.814508	-2.089131	-0.172097
30	1	0	-1.377832	-2.781847	-0.655650
31	1	0	-0.819806	-0.113613	-1.124894
32	1	0	3.077687	-0.711854	0.147968
33	6	0	0.973552	2.628005	0.619393
34	1	0	1.490184	3.409060	1.164623
35	8	0	3.036055	1.524049	0.099274

Most stable energy, Gibbs free energy (Ha), and geometry for protomer ./metaOH_PyN3//3_1
E: -725.138657
G: -724.850957
Geometry:
Input orientation:

Center Number	Atomic Number	Atomic Type	Coordinates (Angstroms) X	Y	Z
1	6	0	0.373310	-1.566477	0.416404
2	6	0	1.437586	-2.116709	-0.286109
3	6	0	2.434031	0.042577	-0.394070
4	6	0	1.327616	0.527011	0.298735
5	7	0	0.341486	-0.262108	0.703682
6	1	0	1.439235	-3.173093	-0.520024
7	6	0	1.259640	1.999869	0.602081
8	1	0	2.007217	2.266509	1.348468
9	1	0	1.445307	2.597273	-0.290112
10	6	0	-0.750134	-2.454260	0.903024
11	1	0	-0.509781	-2.764291	1.922437
12	1	0	-0.755016	-3.359912	0.285619
13	7	0	-0.071796	2.382774	1.146641
14	1	0	-0.419893	1.603152	1.717006
15	7	0	-2.058296	-1.812682	0.937887
16	1	0	-2.641053	-2.308420	1.598885
17	6	0	-1.095524	2.795753	0.130983
18	1	0	-0.686370	3.665619	-0.376967
19	1	0	-1.983874	3.093577	0.685474
20	6	0	-1.416689	1.740903	-0.900030
21	1	0	-2.013133	2.201934	-1.684222
22	1	0	-0.523119	1.322478	-1.358168
23	6	0	-2.752136	-1.760057	-0.346371
24	1	0	-2.634687	-2.678719	-0.931359
25	1	0	-3.814784	-1.621462	-0.146836
26	6	0	-2.250581	-0.600946	-1.193064
27	1	0	-2.899785	-0.407883	-2.043482
28	1	0	-1.235905	-0.766981	-1.551279
29	7	0	-2.209852	0.617558	-0.336216
30	1	0	-3.163951	0.932936	-0.139734
31	1	0	0.053470	3.175613	1.780201
32	1	0	-1.805324	0.286799	0.554920
33	6	0	2.486100	-1.308511	-0.689508
34	1	0	3.330145	-1.708711	-1.238737
35	8	0	3.393388	0.937768	-0.747306
36	1	0	4.097526	0.493665	-1.239874

Most stable energy, Gibbs free energy (Ha), and geometry for protomer ./metaOH_PyN3//3_7
E: -725.122710
G: -724.832251
Geometry:
Input orientation:

Center Number	Atomic Number	Atomic Type	Coordinates (Angstroms) X	Y	Z
1	6	0	-1.358037	1.327455	-0.288889
2	6	0	-0.987173	2.198001	0.727559
3	6	0	1.376580	1.700843	0.341723
4	6	0	0.936141	0.876610	-0.721071
5	7	0	-0.373701	0.756696	-0.984559
6	1	0	-1.764815	2.679339	1.305379
7	6	0	1.913511	0.067105	-1.501312
8	1	0	1.433880	-0.562063	-2.249233
9	1	0	2.643016	0.716557	-1.983933
10	6	0	-2.750722	0.853749	-0.599050
11	1	0	-3.112466	1.337189	-1.507775
12	1	0	-3.403362	1.157463	0.225452
13	7	0	2.698660	-0.819533	-0.579229
14	1	0	3.553548	-1.091787	-1.071219
15	7	0	-2.749492	-0.593432	-0.820306
16	1	0	-3.534043	-0.831585	-1.413062
17	6	0	2.050842	-2.083038	-0.102868
18	1	0	2.848167	-2.654967	0.363191
19	1	0	1.713925	-2.622410	-0.987416
20	6	0	0.932213	-1.879694	0.909217
21	1	0	1.018709	-2.618706	1.701049
22	1	0	0.975302	-0.894210	1.369146
23	6	0	-2.834216	-1.393370	0.401807
24	1	0	-3.585027	-1.004495	1.098695
25	1	0	-3.132543	-2.403846	0.120739
26	6	0	-1.525165	-1.457321	1.167295
27	1	0	-1.629028	-2.080712	2.052471
28	1	0	-1.180588	-0.473004	1.478919
29	7	0	-0.438433	-2.055040	0.330635
30	1	0	-0.621505	-3.056622	0.212533
31	1	0	-0.658137	0.136971	-1.745004
32	1	0	2.991623	-0.224045	0.213532
33	1	0	-0.495132	-1.659995	-0.611719
34	6	0	0.342312	2.398979	1.025569
35	1	0	0.626085	3.061131	1.834625
36	8	0	2.616020	1.746276	0.651148

Most stable energy, Gibbs free energy (Ha), and geometry for protomer ./metaOH_PyN3//3_5
E: -725.137238
G: -724.851778
Geometry:

Input orientation:

Center Number	Atomic Number	Atomic Type	Coordinates (Angstroms) X	Y	Z
1	6	0	0.626378	-1.720148	-0.345825
2	6	0	1.784660	-2.185777	0.238080
3	6	0	2.660411	0.058989	0.259051
4	6	0	1.474750	0.504701	-0.311029
5	7	0	0.554967	-0.409813	-0.607671
6	1	0	1.877952	-3.235833	0.475961
7	6	0	1.159558	1.947020	-0.590642
8	1	0	1.431144	2.522571	0.300405
9	1	0	1.822843	2.281512	-1.391005
10	6	0	-0.584191	-2.540033	-0.700040

11	1	0	-0.626232	-3.395164	-0.017769
12	1	0	-0.459898	-2.930127	-1.711708
13	7	0	-0.229972	2.153647	-0.977302
14	1	0	-0.257481	2.756001	-1.787471
15	7	0	-1.780808	-1.709357	-0.663423
16	1	0	-2.514563	-2.156808	-1.201324
17	6	0	-1.092636	2.713077	0.066012
18	1	0	-1.862750	3.317189	-0.415551
19	1	0	-0.537338	3.368388	0.743151
20	6	0	-1.760461	1.644956	0.908939
21	1	0	-1.035553	0.935858	1.304862
22	1	0	-2.301193	2.087233	1.742756
23	6	0	-2.252336	-1.463894	0.698766
24	1	0	-2.701907	-2.351686	1.153899
25	1	0	-1.396036	-1.192094	1.319167
26	6	0	-3.289492	-0.361460	0.717772
27	1	0	-3.593900	-0.121937	1.734323
28	1	0	-4.166073	-0.646866	0.138252
29	7	0	-2.763157	0.892113	0.095788
30	1	0	-2.352762	0.645865	-0.811958
31	1	0	-0.305720	-0.057422	-1.034826
32	1	0	-3.546128	1.519656	-0.103441
33	6	0	2.808632	-1.295203	0.528672
34	1	0	3.724855	-1.643531	0.990599
35	8	0	3.592284	0.997133	0.531619
36	1	0	4.378415	0.589397	0.921202

Most stable energy, Gibbs free energy (Ha), and geometry for protomer ./metaOH_PyN3//3_2
E: -725.138258
G: -724.849526
Geometry:
Input orientation:

Center Number	Atomic Number	Atomic Type	Coordinates (Angstroms) X	Y	Z
1	6	0	0.529418	-1.656173	-0.048137
2	6	0	1.747579	-2.090288	0.446738
3	6	0	2.546344	0.137388	0.188869
4	6	0	1.288966	0.506191	-0.299016
5	7	0	0.315706	-0.394718	-0.417535
6	1	0	1.886330	-3.125396	0.729867
7	6	0	1.083447	1.950359	-0.696735
8	1	0	1.448826	2.576681	0.126632
9	1	0	1.748002	2.150043	-1.540390
10	6	0	-0.597265	-2.652547	-0.167653
11	1	0	-0.925195	-2.995227	0.813749
12	1	0	-0.266658	-3.519801	-0.735104
13	7	0	-0.275033	2.313976	-1.074404
14	1	0	-0.232486	2.966209	-1.845139
15	7	0	-1.786322	-2.089765	-0.871597
16	1	0	-2.193345	-2.824437	-1.454728
17	6	0	-1.055324	2.922747	0.000704
18	1	0	-1.916460	3.420128	-0.446598
19	1	0	-0.487992	3.669748	0.566088
20	6	0	-1.537745	1.866369	0.975117
21	1	0	-0.707078	1.387786	1.493088
22	1	0	-2.232999	2.268784	1.707495
23	6	0	-2.883620	-1.579284	0.011735

24	1	0	-3.697626	-1.270694	-0.642067
25	1	0	-3.212529	-2.423967	0.612176
26	6	0	-2.460724	-0.460106	0.930020
27	1	0	-1.551694	-0.698365	1.479757
28	1	0	-3.257940	-0.281899	1.648130
29	7	0	-2.226163	0.804819	0.190512
30	1	0	-1.592793	0.639826	-0.608110
31	1	0	-1.466810	-1.355610	-1.512822
32	1	0	-3.110510	1.166079	-0.178314
33	6	0	2.776270	-1.174303	0.564964
34	1	0	3.750166	-1.464150	0.941650
35	8	0	3.497798	1.107628	0.263122
36	1	0	4.318751	0.738536	0.617379

Most stable energy, Gibbs free energy (Ha), and geometry for protomer ./metaOH_PyN3//3_9

E: -725.118244

G: -724.829171

Geometry:

Input orientation:

Center Number	Atomic Number	Atomic Type	Coordinates (Angstroms) X	Y	Z
1	6	0	0.120049	-1.650288	-0.559773
2	6	0	0.987017	-2.396783	0.213215
3	6	0	2.523529	-0.489511	0.412495
4	6	0	1.576585	0.227762	-0.383143
5	7	0	0.496961	-0.387857	-0.835952
6	1	0	0.721357	-3.415845	0.463846
7	6	0	1.710362	1.686328	-0.710170
8	1	0	2.032245	2.198998	0.203485
9	1	0	2.514947	1.812472	-1.437779
10	6	0	-1.250109	-2.049923	-0.983094
11	1	0	-1.277076	-3.056800	-1.391633
12	1	0	-1.655863	-1.354334	-1.715058
13	7	0	0.468033	2.237779	-1.245929
14	1	0	0.684543	2.863147	-2.009725
15	7	0	-2.199788	-2.062370	0.193812
16	1	0	-1.977468	-2.892161	0.752056
17	6	0	-0.347010	2.958486	-0.266894
18	1	0	-1.036687	3.607610	-0.807892
19	1	0	0.266123	3.595080	0.379455
20	6	0	-1.136685	2.045877	0.651216
21	1	0	-0.504679	1.286232	1.107239
22	1	0	-1.601601	2.621954	1.448233
23	6	0	-2.198741	-0.903439	1.146970
24	1	0	-2.693455	-1.282970	2.038197
25	1	0	-1.166196	-0.697424	1.420450
26	6	0	-2.987542	0.318739	0.716001
27	1	0	-3.324928	0.823269	1.619025
28	1	0	-3.863045	0.035103	0.134061
29	7	0	-2.258144	1.364023	-0.072207
30	1	0	-1.946715	0.983044	-0.969672
31	1	0	-0.084637	0.197509	-1.441896
32	1	0	-3.149635	-2.204233	-0.165089
33	1	0	-2.946067	2.083340	-0.319163
34	6	0	2.165998	-1.838589	0.675785
35	1	0	2.841821	-2.430350	1.281098
36	8	0	3.580351	0.075371	0.833377

Most stable energy, Gibbs free energy (Ha), and geometry for protomer ./metaOH_PyN3//3_6
E: -725.127985
G: -724.840875
Geometry:
Input orientation:

Center Number	Atomic Number	Atomic Type	Coordinates (Angstroms) X	Y	Z
1	6	0	-0.043906	-1.799590	-0.009560
2	6	0	1.066631	-2.440867	-0.550664
3	6	0	2.402835	-0.500890	-0.068979
4	6	0	1.284292	0.103700	0.469268
5	7	0	0.126200	-0.579524	0.481238
6	1	0	0.956430	-3.439869	-0.949416
7	6	0	1.272536	1.487967	1.030310
8	1	0	0.451138	1.619950	1.728865
9	1	0	2.210190	1.699849	1.538553
10	6	0	-1.421706	-2.401468	0.031193
11	1	0	-1.327835	-3.388970	0.483785
12	1	0	-1.739935	-2.560843	-1.007109
13	7	0	1.121435	2.514812	-0.047347
14	1	0	1.128943	3.436239	0.401347
15	7	0	-2.368426	-1.599633	0.785492
16	1	0	-2.943677	-2.218884	1.339652
17	6	0	-0.104668	2.413756	-0.907419
18	1	0	-0.035355	1.477196	-1.458286
19	1	0	-0.016035	3.230015	-1.619748
20	6	0	-1.402317	2.546557	-0.126687
21	1	0	-1.254441	3.238326	0.705209
22	1	0	-2.130661	3.007166	-0.802397
23	6	0	-3.246616	-0.753037	-0.025027
24	1	0	-3.711433	-1.317115	-0.842357
25	1	0	-4.045227	-0.397941	0.628350
26	6	0	-2.543220	0.447957	-0.631046
27	1	0	-3.276456	1.018407	-1.212427
28	1	0	-1.773513	0.117619	-1.336317
29	7	0	-1.915212	1.279464	0.401043
30	1	0	-2.614495	1.489793	1.106894
31	1	0	-0.718215	-0.038832	0.798893
32	1	0	1.945259	2.476128	-0.655976
33	6	0	2.282739	-1.799588	-0.576413
34	1	0	3.157069	-2.284355	-0.994006
35	8	0	3.548305	0.205977	-0.084989
36	1	0	4.267612	-0.312231	-0.474301

Most stable energy, Gibbs free energy (Ha), and geometry for protomer ./metaOH_PyN3//3_8
E: -725.129151
G: -724.842228
Geometry:
Input orientation:

Center Number	Atomic Number	Atomic Type	Coordinates (Angstroms) X	Y	Z
1	6	0	0.242855	-1.535613	0.517370
2	6	0	1.327345	-2.275135	0.110197
3	6	0	2.526096	-0.235138	-0.296202
4	6	0	1.410489	0.487854	0.139316

5	7	0	0.339659	-0.194696	0.524842
6	1	0	1.272655	-3.354672	0.115681
7	6	0	1.389731	1.987832	0.185640
8	1	0	2.307140	2.302287	0.685327
9	1	0	1.462196	2.353522	-0.847650
10	6	0	-1.058054	-2.124036	0.963720
11	1	0	-1.565490	-1.481204	1.678506
12	1	0	-0.887227	-3.094758	1.421865
13	7	0	0.236783	2.516880	0.891995
14	1	0	0.545235	3.284178	1.473033
15	7	0	-1.992617	-2.354551	-0.183467
16	1	0	-2.852844	-2.757203	0.202155
17	6	0	-0.850384	2.996008	0.036843
18	1	0	-0.486025	3.674013	-0.743995
19	1	0	-1.535528	3.559185	0.672417
20	6	0	-1.620243	1.878316	-0.641162
21	1	0	-2.429623	2.321109	-1.231529
22	1	0	-0.970823	1.347833	-1.345224
23	6	0	-2.357881	-1.168535	-1.026778
24	1	0	-1.448110	-0.832036	-1.521206
25	1	0	-3.025867	-1.562965	-1.788404
26	6	0	-3.054535	-0.062916	-0.248704
27	1	0	-3.654755	-0.503747	0.549507
28	1	0	-3.746935	0.425798	-0.941286
29	7	0	-2.137586	0.917787	0.339733
30	1	0	-2.635212	1.432354	1.060179
31	1	0	-0.556148	0.339319	0.750246
32	1	0	-1.584116	-3.066399	-0.796459
33	6	0	2.480833	-1.618300	-0.300761
34	1	0	3.350883	-2.176503	-0.624963
35	8	0	3.591716	0.490207	-0.685211
36	1	0	4.316701	-0.087454	-0.965635

Most stable energy, Gibbs free energy (Ha), and geometry for protomer ./metaOH_PyN3//4_2
E: -725.572065
G: -725.268470
Geometry:

Input orientation:

Center Number	Atomic Number	Atomic Type	Coordinates (Angstroms) X	Y	Z
1	6	0	0.065966	-1.784937	-0.450313
2	6	0	0.943832	-2.418337	0.415220
3	6	0	2.388996	-0.498423	0.343417
4	6	0	1.511641	0.102174	-0.544409
5	7	0	0.416017	-0.583894	-0.914909
6	1	0	0.687414	-3.395458	0.799192
7	6	0	1.715605	1.482238	-1.072853
8	1	0	2.778675	1.695672	-1.140802
9	1	0	1.258585	1.599706	-2.053277
10	6	0	-1.284557	-2.308575	-0.848018
11	1	0	-1.487361	-3.198781	-0.245997
12	1	0	-1.261158	-2.606464	-1.897377
13	7	0	1.123170	2.538546	-0.180358
14	1	0	1.430073	2.390372	0.788749
15	7	0	-2.279474	-1.253410	-0.681454
16	1	0	-3.129056	-1.519955	-1.166901
17	6	0	-0.365844	2.694699	-0.240918

18	1	0	-0.651378	2.498302	-1.274146
19	1	0	-0.581043	3.735763	-0.017121
20	6	0	-1.102903	1.776103	0.730719
21	1	0	-0.563707	0.853018	0.928272
22	1	0	-1.284737	2.267662	1.682932
23	6	0	-2.574428	-0.979908	0.725623
24	1	0	-3.246702	-1.718333	1.170816
25	1	0	-1.640962	-1.024185	1.289656
26	6	0	-3.211567	0.387404	0.888704
27	1	0	-3.262716	0.683841	1.933715
28	1	0	-4.210031	0.408250	0.456300
29	7	0	-2.427238	1.424265	0.147282
30	1	0	-2.283596	1.051277	-0.800277
31	1	0	-0.247437	-0.121927	-1.543366
32	1	0	1.537454	3.428757	-0.473607
33	1	0	-2.990953	2.274150	0.060555
34	6	0	2.107208	-1.787814	0.800177
35	1	0	2.796703	-2.265278	1.486102
36	8	0	3.459125	0.213647	0.726939
37	1	0	4.016291	-0.297121	1.332631

Most stable energy, Gibbs free energy (Ha), and geometry for protomer ./metaOH_PyN3//4_3
E: -725.572172
G: -725.268284
Geometry:
Input orientation:

Center Number	Atomic Number	Atomic Type	Coordinates (Angstroms) X	Y	Z
1	6	0	0.434770	-1.746878	0.367600
2	6	0	1.457266	-2.203871	-0.433305
3	6	0	2.537939	-0.062328	-0.263212
4	6	0	1.476986	0.388552	0.521696
5	7	0	0.514929	-0.485389	0.824535
6	1	0	1.420705	-3.214984	-0.812817
7	6	0	1.308070	1.802238	1.002068
8	1	0	1.615499	1.860058	2.047841
9	1	0	1.977200	2.441589	0.421462
10	6	0	-0.753889	-2.575511	0.734889
11	1	0	-1.107978	-2.351024	1.739070
12	1	0	-0.479779	-3.625075	0.676185
13	7	0	-0.094345	2.187472	0.891414
14	1	0	-0.261459	2.995962	1.480592
15	7	0	-1.925884	-2.397827	-0.194751
16	1	0	-2.527641	-3.213941	-0.049122
17	6	0	-0.469515	2.497959	-0.489840
18	1	0	-0.022769	1.748764	-1.146101
19	1	0	-0.091676	3.469425	-0.820725
20	6	0	-1.976254	2.508395	-0.656650
21	1	0	-2.424353	3.338138	-0.113506
22	1	0	-2.261286	2.565772	-1.704610
23	6	0	-2.775946	-1.178935	0.007271
24	1	0	-3.791649	-1.458397	-0.256750
25	1	0	-2.746138	-0.955147	1.073476
26	6	0	-2.308883	0.010953	-0.825405
27	1	0	-2.837908	0.069815	-1.772872
28	1	0	-1.244276	-0.029895	-1.040243
29	7	0	-2.582909	1.268571	-0.078377

30	1	0	-3.594836	1.395945	0.006949
31	1	0	-0.244169	-0.118696	1.405804
32	1	0	-1.604973	-2.455014	-1.168575
33	1	0	-2.205537	1.175100	0.873082
34	6	0	2.517549	-1.366110	-0.740460
35	1	0	3.330541	-1.713984	-1.366253
36	8	0	3.517861	0.815583	-0.526326
37	1	0	4.188986	0.419121	-1.100952

Most stable energy, Gibbs free energy (Ha), and geometry for protomer ./H_Py2N2//0_1

E: -762.105531

G: -761.854217

Geometry:

Input orientation:

Center Number	Atomic Number	Atomic Type	Coordinates (Angstroms) X	Y	Z
1	1	0	-0.001853	2.226943	-3.029451
2	1	0	0.001921	-2.246387	-3.014791
3	6	0	-0.001589	1.997329	-1.971110
4	6	0	1.192732	1.823365	-1.288912
5	6	0	1.148807	1.527751	0.069151
6	7	0	-0.000896	1.442388	0.737084
7	6	0	-1.150886	1.526689	0.069500
8	6	0	-1.195541	1.822315	-1.288528
9	6	0	-2.405636	1.233937	0.864310
10	7	0	-2.373518	0.003642	1.658428
11	6	0	-2.404248	-1.231305	0.871615
12	6	0	-1.148772	-1.527770	0.079361
13	7	0	0.000882	-1.438033	0.746809
14	6	0	1.150852	-1.526707	0.079753
15	6	0	1.195534	-1.830981	-1.276343
16	6	0	0.001633	-2.010282	-1.957875
17	6	0	-1.192665	-1.832028	-1.276773
18	6	0	2.405717	-1.228784	0.872388
19	7	0	2.373484	0.006584	1.658469
20	6	0	2.404133	1.236456	0.863626
21	1	0	2.145596	1.888806	-1.798265
22	1	0	-2.148629	1.886901	-1.797566
23	1	0	-3.259728	1.190142	0.187735
24	1	0	-2.577426	2.067833	1.548982
25	1	0	-3.257886	-1.192133	0.194180
26	1	0	-2.575926	-2.061372	1.560965
27	1	0	2.148640	-1.898649	-1.784959
28	1	0	-2.145525	-1.900549	-1.785739
29	1	0	2.577850	-2.058302	1.562282
30	1	0	3.259604	-1.189200	0.195291
31	1	0	1.506563	0.007829	2.190133
32	1	0	3.258006	1.193071	0.186747
33	1	0	2.575438	2.070896	1.547760
34	1	0	-1.506610	0.005622	2.190110

Most stable energy, Gibbs free energy (Ha), and geometry for protomer ./H_Py2N2//1_3

E: -762.564206

G: -762.303442

Geometry:

Input orientation:

Center Atomic Atomic Coordinates (Angstroms)

Number	Number	Type	X	Y	Z
1	1	0	5.026841	-0.410032	0.781374
2	1	0	-5.006536	0.415910	0.794408
3	6	0	3.980805	-0.297057	0.525989
4	6	0	3.480757	0.941290	0.172791
5	6	0	2.132721	1.046201	-0.167156
6	7	0	1.336377	-0.013109	-0.141285
7	6	0	1.804454	-1.215122	0.212755
8	6	0	3.132553	-1.400938	0.547468
9	6	0	0.778985	-2.323715	0.264826
10	7	0	-0.125289	-2.303424	-0.889139
11	6	0	-1.536198	-2.391425	-0.572395
12	6	0	-2.142405	-1.072275	-0.175136
13	7	0	-1.371732	0.015885	-0.157376
14	6	0	-1.796334	1.239053	0.207474
15	6	0	-3.118083	1.406695	0.549956
16	6	0	-3.963569	0.300182	0.529669
17	6	0	-3.477460	-0.944443	0.176955
18	6	0	-0.751085	2.320964	0.263063
19	7	0	0.132525	2.320601	-0.902678
20	6	0	1.550666	2.377260	-0.582681
21	1	0	4.113410	1.819285	0.148237
22	1	0	3.494487	-2.383711	0.818524
23	1	0	0.193714	-2.186770	1.179660
24	1	0	1.288916	-3.283122	0.359769
25	1	0	-1.761607	-3.103766	0.231130
26	1	0	-2.074326	-2.737987	-1.456072
27	1	0	-3.476683	2.386367	0.832037
28	1	0	-4.112970	-1.819149	0.165063
29	1	0	-1.260421	3.276323	0.390826
30	1	0	-0.164140	2.152463	1.171118
31	1	0	-0.095031	3.116686	-1.482723
32	1	0	1.777906	3.104955	0.206867
33	1	0	2.085822	2.710349	-1.473431
34	1	0	0.114071	-3.054871	-1.520527
35	1	0	-0.326192	-0.056015	-0.361565

Most stable energy, Gibbs free energy (Ha), and geometry for protomer ./H_Py2N2//1_2
E: -762.565366
G: -762.302993
Geometry:
Input orientation:

Center Number	Atomic Number	Atomic Type	Coordinates (Angstroms) X	Y	Z
1	1	0	-0.374731	2.671449	-2.748065
2	1	0	-0.070947	-1.836153	-3.058492
3	6	0	-0.227366	2.269183	-1.754008
4	6	0	1.049130	1.963269	-1.303672
5	6	0	1.181960	1.465643	-0.017574
6	7	0	0.148645	1.280134	0.799091
7	6	0	-1.085019	1.501623	0.344872
8	6	0	-1.309668	2.020963	-0.927490
9	6	0	-2.248853	1.115662	1.229593
10	7	0	-3.011731	-0.058738	0.793095
11	6	0	-2.348851	-1.351805	0.981187
12	6	0	-1.101911	-1.569413	0.155309

13	7	0	0.067075	-1.597643	0.786522
14	6	0	1.187197	-1.624708	0.058364
15	6	0	1.194009	-1.717036	-1.320378
16	6	0	-0.030543	-1.758277	-1.979647
17	6	0	-1.189559	-1.662116	-1.235488
18	6	0	2.453600	-1.422138	0.838543
19	7	0	2.428041	-0.063161	1.475182
20	6	0	2.536073	1.093728	0.535090
21	1	0	1.919351	2.092102	-1.933484
22	1	0	-2.323097	2.208531	-1.259963
23	1	0	-2.941098	1.957620	1.276647
24	1	0	-1.880407	0.927202	2.237730
25	1	0	-3.075116	-2.128005	0.735919
26	1	0	-2.090269	-1.453548	2.034795
27	1	0	2.130405	-1.730132	-1.862124
28	1	0	-2.161861	-1.645643	-1.712673
29	1	0	2.534289	-2.139716	1.652762
30	1	0	3.337834	-1.472174	0.208899
31	1	0	3.185105	-0.009437	2.158354
32	1	0	3.230983	0.826026	-0.257395
33	1	0	2.952914	1.923206	1.105513
34	1	0	-3.263734	0.050887	-0.185477
35	1	0	1.536283	0.037649	1.981323

Most stable energy, Gibbs free energy (Ha), and geometry for protomer ./H_Py2N2//1_1

E: -762.565366

G: -762.303001

Geometry:

Input orientation:

Center Number	Atomic Number	Atomic Type	Coordinates (Angstroms) X	Y	Z
1	1	0	0.073126	1.833358	-3.060437
2	1	0	0.371914	-2.674074	-2.745553
3	6	0	0.032497	1.756441	-1.981532
4	6	0	1.191310	1.659810	-1.237124
5	6	0	1.103363	1.568355	0.153747
6	7	0	-0.065682	1.598247	0.784745
7	6	0	-1.185678	1.625741	0.056399
8	6	0	-1.192198	1.716900	-1.322416
9	6	0	-2.452385	1.425165	0.836594
10	7	0	-2.428047	0.067059	1.475105
11	6	0	-2.537062	-1.091049	0.536636
12	6	0	-1.183357	-1.464671	-0.015841
13	7	0	-0.149793	-1.279504	0.800603
14	6	0	1.083604	-1.502490	0.346408
15	6	0	1.307639	-2.023037	-0.925583
16	6	0	0.225031	-2.270905	-1.751790
17	6	0	-1.051130	-1.963423	-1.301559
18	6	0	2.247911	-1.116872	1.230643
19	7	0	3.011775	0.056519	0.793102
20	6	0	2.349978	1.350314	0.980008
21	1	0	2.163663	1.642044	-1.714155
22	1	0	-2.128489	1.730382	-1.864331
23	1	0	-3.336445	1.475030	0.206688
24	1	0	-2.532709	2.143917	1.649823
25	1	0	-3.231987	-0.823954	-0.256041
26	1	0	-2.954308	-1.919443	1.108345

27	1	0	2.320863	-2.211785	-1.258007
28	1	0	-1.921534	-2.091924	-1.931184
29	1	0	1.879822	-0.927323	2.238706
30	1	0	2.939457	-1.959373	1.278185
31	1	0	3.263641	-0.054223	-0.185386
32	1	0	3.076960	2.125671	0.734191
33	1	0	2.091335	1.453175	2.033495
34	1	0	-3.185262	0.014942	2.158239
35	1	0	-1.536469	-0.033766	1.981530

Most stable energy, Gibbs free energy (Ha), and geometry for protomer ./H_Py2N2//1_4
E: -762.564205
G: -762.303441
Geometry:
Input orientation:

Center Number	Atomic Number	Atomic Type	Coordinates (Angstroms) X	Y	Z
1	1	0	-5.006630	0.412605	0.794882
2	1	0	5.026994	-0.406715	0.781718
3	6	0	-3.963630	0.297571	0.529966
4	6	0	-3.118877	1.404646	0.550131
5	6	0	-1.797071	1.237895	0.207440
6	7	0	-1.371719	0.015015	-0.157500
7	6	0	-2.141668	-1.073657	-0.175161
8	6	0	-3.476752	-0.946725	0.177153
9	6	0	-1.534650	-2.392383	-0.572585
10	7	0	-0.123792	-2.303470	-0.889313
11	6	0	0.780480	-2.323225	0.264664
12	6	0	1.805216	-1.213944	0.212689
13	7	0	1.336399	-0.012249	-0.141447
14	6	0	2.132045	1.047584	-0.167248
15	6	0	3.480104	0.943575	0.172893
16	6	0	3.980920	-0.294435	0.526185
17	6	0	3.133394	-1.398876	0.547573
18	6	0	1.549153	2.378245	-0.582885
19	7	0	0.131039	2.320670	-0.902841
20	6	0	-0.752543	2.320510	0.262914
21	1	0	-3.478090	2.384072	0.832286
22	1	0	-4.111682	-1.821853	0.165353
23	1	0	-2.072556	-2.739157	-1.456317
24	1	0	-1.759631	-3.104976	0.230837
25	1	0	1.291054	-3.282296	0.359550
26	1	0	0.195107	-2.186746	1.179502
27	1	0	4.112179	1.821988	0.148410
28	1	0	3.495943	-2.381402	0.818703
29	1	0	1.775958	3.106163	0.206581
30	1	0	2.084075	2.711580	-1.473685
31	1	0	-0.097040	3.116576	-1.482927
32	1	0	-1.262521	3.275536	0.390629
33	1	0	-0.165469	2.152479	1.170974
34	1	0	0.116049	-3.054757	-1.520712
35	1	0	-0.326169	-0.056216	-0.361799

Most stable energy, Gibbs free energy (Ha), and geometry for protomer ./H_Py2N2//2_1
E: -763.024367
G: -762.746398
Geometry:
Input orientation:

Center Number	Atomic Number	Atomic Type	Coordinates (Angstroms) X	Y	Z
1	1	0	1.897403	1.398333	-3.009535
2	1	0	-1.897654	-1.398224	-3.009446
3	6	0	1.644344	1.286012	-1.963509
4	6	0	0.587299	1.994434	-1.418737
5	6	0	0.301862	1.819169	-0.070071
6	7	0	1.007291	1.012520	0.710847
7	6	0	1.974262	0.275058	0.165684
8	6	0	2.341692	0.389768	-1.164025
9	6	0	2.584831	-0.755672	1.071336
10	7	0	1.516476	-1.665002	1.599285
11	6	0	0.852321	-2.537884	0.582228
12	6	0	-0.301873	-1.819288	-0.070144
13	7	0	-1.007239	-1.012700	0.710896
14	6	0	-1.974252	-0.275196	0.165868
15	6	0	-2.341793	-0.389804	-1.163820
16	6	0	-1.644510	-1.285985	-1.963431
17	6	0	-0.587420	-1.994448	-1.418800
18	6	0	-2.584733	0.755479	1.071642
19	7	0	-1.516321	1.664778	1.599531
20	6	0	-0.852280	2.537718	0.582449
21	1	0	-0.019073	2.657310	-2.021390
22	1	0	3.133349	-0.228269	-1.565302
23	1	0	3.048933	-0.289893	1.938939
24	1	0	3.312232	-1.372954	0.551495
25	1	0	0.495056	-3.421498	1.109920
26	1	0	1.599775	-2.839186	-0.147704
27	1	0	-3.133483	0.228264	-1.564984
28	1	0	0.018906	-2.657274	-2.021554
29	1	0	-3.312186	1.372790	0.551908
30	1	0	-3.048747	0.289652	1.939266
31	1	0	-0.784530	1.092092	2.042886
32	1	0	-0.494979	3.421316	1.110142
33	1	0	-1.599810	2.839039	-0.147395
34	1	0	0.784733	-1.092345	2.042757
35	1	0	-1.927219	2.253834	2.326323
36	1	0	1.927455	-2.254102	2.325996

Most stable energy, Gibbs free energy (Ha), and geometry for protomer ./H_Py2N2//2_3

E: -763.013986

G: -762.736249

Geometry:

Input orientation:

Center Number	Atomic Number	Atomic Type	Coordinates (Angstroms) X	Y	Z
1	1	0	-2.129021	1.564267	-2.801785
2	1	0	1.888498	-1.334908	-3.056899
3	6	0	-1.770255	1.415219	-1.791692
4	6	0	-0.625376	2.056276	-1.353905
5	6	0	-0.213338	1.831111	-0.046385
6	7	0	-0.866848	1.037320	0.790410
7	6	0	-1.932796	0.360723	0.348886
8	6	0	-2.426305	0.540942	-0.934318
9	6	0	-2.511209	-0.679985	1.272694

10	7	0	-1.519697	-1.725573	1.568793
11	6	0	-1.094480	-2.469635	0.392025
12	6	0	0.125784	-1.843758	-0.231703
13	7	0	0.889120	-1.103221	0.570653
14	6	0	1.933384	-0.359497	0.161272
15	6	0	2.321874	-0.444396	-1.151875
16	6	0	1.598088	-1.266660	-2.016986
17	6	0	0.486985	-1.952195	-1.565509
18	6	0	2.564543	0.563064	1.157010
19	7	0	1.631632	1.657738	1.582044
20	6	0	1.042929	2.480870	0.483117
21	1	0	-0.051184	2.703910	-2.003071
22	1	0	-3.292316	-0.018522	-1.261600
23	1	0	-3.408923	-1.098671	0.808317
24	1	0	-2.795685	-0.215121	2.216962
25	1	0	-1.864761	-2.541349	-0.383495
26	1	0	-0.831823	-3.488378	0.683573
27	1	0	3.157949	0.145364	-1.498923
28	1	0	-0.121685	-2.549593	-2.229601
29	1	0	2.854696	0.027784	2.059779
30	1	0	3.434920	1.036951	0.713206
31	1	0	2.152819	2.267869	2.216731
32	1	0	1.791347	2.598697	-0.298294
33	1	0	0.818352	3.458567	0.907664
34	1	0	-1.919695	-2.368115	2.240298
35	1	0	0.530064	-1.011786	1.527477
36	1	0	0.846733	1.263857	2.119155

Most stable energy, Gibbs free energy (Ha), and geometry for protomer ./H_Py2N2//2_6
E: -763.015934
G: -762.739677
Geometry:

Input orientation:

Center Number	Atomic Number	Atomic Type	Coordinates (Angstroms) X	Y	Z
1	1	0	0.003786	2.362972	2.949293
2	1	0	-0.004091	-2.726076	2.938775
3	6	0	0.003423	2.120263	1.894854
4	6	0	1.208676	1.935021	1.230303
5	6	0	1.192467	1.612767	-0.109867
6	7	0	0.002541	1.522596	-0.723067
7	6	0	-1.187126	1.616629	-0.109935
8	6	0	-1.202392	1.938876	1.230242
9	6	0	-2.396069	1.275053	-0.933648
10	7	0	-2.202118	-0.004205	-1.615372
11	6	0	-2.422095	-1.167077	-0.762787
12	6	0	-1.192694	-1.541493	0.020786
13	7	0	-0.002334	-1.353059	-0.568545
14	6	0	1.187607	-1.545349	0.020491
15	6	0	1.202288	-2.058584	1.300195
16	6	0	-0.003606	-2.330308	1.931759
17	6	0	-1.208752	-2.054821	1.300479
18	6	0	2.418005	-1.175274	-0.763543
19	7	0	2.202050	-0.011247	-1.615589
20	6	0	2.400360	1.267116	-0.933448
21	1	0	2.156475	2.006087	1.744209
22	1	0	-2.149995	2.013002	1.744084

23	1	0	-2.533459	2.049023	-1.690482
24	1	0	-3.268328	1.280112	-0.274533
25	1	0	-2.680931	-2.015806	-1.398918
26	1	0	-3.240257	-1.026228	-0.050290
27	1	0	2.149538	-2.216199	1.795791
28	1	0	-2.156405	-2.209447	1.796241
29	1	0	3.237045	-1.037858	-0.051374
30	1	0	2.673292	-2.024723	-1.400155
31	1	0	2.831841	-0.060755	-2.405591
32	1	0	2.540601	2.040792	-1.690054
33	1	0	3.272503	1.269010	-0.274154
34	1	0	-2.832136	-0.051837	-2.405311
35	1	0	0.002175	1.264598	-1.711077
36	1	0	-0.001912	-0.962853	-1.512644

Most stable energy, Gibbs free energy (Ha), and geometry for protomer ./H_Py2N2//2_2
E: -763.013987
G: -762.736260
Geometry:
Input orientation:

Center Number	Atomic Number	Atomic Type	Coordinates (Angstroms) X	Y	Z
1	1	0	2.125346	-1.567159	-2.801935
2	1	0	-1.887391	1.340931	-3.056456
3	6	0	1.767146	-1.417948	-1.791670
4	6	0	2.425232	-0.545619	-0.933908
5	6	0	1.932318	-0.365063	0.349508
6	7	0	0.865125	-1.039726	0.790915
7	6	0	0.209737	-1.831656	-0.046248
8	6	0	0.620954	-2.056805	-1.353997
9	6	0	-1.047661	-2.479157	0.483281
10	7	0	-1.635034	-1.654694	1.581920
11	6	0	-2.565625	-0.558234	1.156550
12	6	0	-1.932482	0.363166	0.161000
13	7	0	-0.886320	1.104273	0.570313
14	6	0	-0.121913	1.843822	-0.231913
15	6	0	-0.483736	1.954119	-1.565412
16	6	0	-1.596514	1.271248	-2.016773
17	6	0	-2.321508	0.449813	-1.151872
18	6	0	1.100106	2.466645	0.391498
19	7	0	1.523233	1.722194	1.568802
20	6	0	2.512688	0.674316	1.273570
21	1	0	3.292390	0.012198	-1.260966
22	1	0	0.045155	-2.702705	-2.003461
23	1	0	-0.824764	-3.457072	0.908240
24	1	0	-1.796246	-2.595969	-0.298119
25	1	0	-2.854998	-0.022352	2.059224
26	1	0	-3.436813	-1.030419	0.712537
27	1	0	0.125750	2.550822	-2.229378
28	1	0	-3.159102	-0.137838	-1.498829
29	1	0	1.870665	2.535535	-0.384011
30	1	0	0.840401	3.486390	0.682194
31	1	0	1.924339	2.364171	2.240197
32	1	0	2.795407	0.208972	2.218120
33	1	0	3.411630	1.090958	0.809753
34	1	0	-0.849405	-1.262436	2.119149
35	1	0	-0.526937	1.011669	1.526929

36	1	0	-2.157598	-2.263712	2.216570

Most stable energy, Gibbs free energy (Ha), and geometry for protomer ./H_Py2N2//2_5
E: -763.013982
G: -762.736210
Geometry:
Input orientation:

Center Number	Atomic Number	Atomic Type	Coordinates (Angstroms) X	Y	Z
1	1	0	1.901137	-1.323183	-3.052610
2	1	0	-2.139938	1.545787	-2.806364
3	6	0	1.609606	-1.256374	-2.012920
4	6	0	2.326243	-0.427562	-1.148143
5	6	0	1.936307	-0.344469	0.164721
6	7	0	0.897974	-1.096365	0.574140
7	6	0	0.140988	-1.843677	-0.227980
8	6	0	0.503701	-1.950339	-1.561493
9	6	0	-1.074297	-2.478943	0.395871
10	7	0	-1.507941	-1.735356	1.569883
11	6	0	-2.507248	-0.698493	1.269439
12	6	0	-1.935704	0.345510	0.345033
13	7	0	-0.875312	1.030734	0.786717
14	6	0	-0.227494	1.828916	-0.050311
15	6	0	-0.640790	2.050264	-1.358126
16	6	0	-1.780376	1.399998	-1.796082
17	6	0	-2.429852	0.520957	-0.938547
18	6	0	1.023394	2.489110	0.478994
19	7	0	1.617063	1.673533	1.580889
20	6	0	2.558815	0.584731	1.159799
21	1	0	3.157664	0.168687	-1.495299
22	1	0	-0.099855	-2.553081	-2.225419
23	1	0	-0.802482	-3.494259	0.691018
24	1	0	-1.842734	-2.560412	-0.380463
25	1	0	-2.797706	-0.234308	2.212235
26	1	0	-3.400498	-1.125344	0.803857
27	1	0	-0.071513	2.702172	-2.007344
28	1	0	-3.291299	-0.045452	-1.265901
29	1	0	1.771860	2.610186	-0.301883
30	1	0	0.791285	3.466387	0.900400
31	1	0	0.834974	1.275014	2.118654
32	1	0	2.851398	0.053518	2.064181
33	1	0	3.426367	1.064244	0.716443
34	1	0	-1.904109	-2.379499	2.242127
35	1	0	0.537804	-1.006918	1.530689
36	1	0	2.133176	2.289453	2.214104

Most stable energy, Gibbs free energy (Ha), and geometry for protomer ./H_Py2N2//2_4
E: -763.013995
G: -762.736347
Geometry:
Input orientation:

Center Number	Atomic Number	Atomic Type	Coordinates (Angstroms) X	Y	Z
1	1	0	-1.870924	1.376518	-3.055038
2	1	0	2.097688	-1.594283	-2.803912
3	6	0	-1.579472	1.299780	-2.016011

4	6	0	-0.455592	1.964260	-1.564810
5	6	0	-0.093235	1.845090	-0.232179
6	7	0	-0.867752	1.115605	0.569504
7	6	0	-1.926161	0.392061	0.160174
8	6	0	-2.315833	0.487656	-1.151868
9	6	0	-2.573365	-0.519933	1.155251
10	7	0	-1.659090	-1.629553	1.581559
11	6	0	-1.084624	-2.464428	0.483994
12	6	0	0.182054	-1.835817	-0.046161
13	7	0	0.850292	-1.055472	0.791778
14	6	0	1.927003	-0.396151	0.350375
15	6	0	2.415564	-0.581581	-0.934065
16	6	0	1.743218	-1.441948	-1.792795
17	6	0	0.588041	-2.064306	-1.354907
18	6	0	2.523070	0.633515	1.275243
19	7	0	1.549689	1.696876	1.568821
20	6	0	1.139724	2.446972	0.390461
21	1	0	0.161920	2.553160	-2.228331
22	1	0	-3.163231	-0.085844	-1.498697
23	1	0	-3.451271	-0.979129	0.710975
24	1	0	-2.855236	0.020451	2.057628
25	1	0	-1.834820	-2.570983	-0.297318
26	1	0	-0.876143	-3.444952	0.910285
27	1	0	3.290544	-0.036090	-1.261147
28	1	0	0.001514	-2.699841	-2.004991
29	1	0	2.797070	0.163757	2.220151
30	1	0	3.429009	1.035987	0.812583
31	1	0	1.959878	2.332873	2.240410
32	1	0	1.911408	2.500521	-0.385188
33	1	0	0.898486	3.471731	0.679315
34	1	0	-2.189873	-2.229366	2.218195
35	1	0	-0.508619	1.015889	1.525513
36	1	0	-0.866606	-1.248365	2.116794

Most stable energy, Gibbs free energy (Ha), and geometry for protomer ./OH_Py2N2//0_1
E: -911.650758
G: -911.422173
Geometry:

Input orientation:

Center Number	Atomic Number	Atomic Type	Coordinates (Angstroms) X	Y	Z
1	6	0	-1.708498	0.226477	1.999398
2	6	0	-1.057842	-1.015131	1.765142
3	6	0	0.297216	-1.060761	1.492714
4	7	0	1.078434	0.023367	1.460814
5	6	0	0.472636	1.215788	1.565343
6	6	0	-0.866828	1.366366	1.854065
7	6	0	1.347498	2.411110	1.256667
8	7	0	2.007381	2.368913	-0.054825
9	6	0	1.086824	2.386846	-1.197998
10	6	0	0.412281	1.063936	-1.511031
11	7	0	1.206028	-0.012216	-1.474690
12	6	0	0.608918	-1.209606	-1.572109
13	6	0	-0.728637	-1.374391	-1.862917
14	6	0	-1.581830	-0.243992	-2.010500
15	6	0	-0.941503	1.004771	-1.782683
16	6	0	1.480049	-2.392119	-1.216668

17	7	0	2.012630	-2.353249	0.153175
18	6	0	0.986649	-2.383287	1.201740
19	1	0	-1.645558	-1.926886	1.762508
20	1	0	-1.303702	2.357040	1.917439
21	1	0	2.133139	2.479515	2.012852
22	1	0	0.754768	3.324761	1.317503
23	1	0	1.655132	2.693145	-2.080852
24	1	0	0.328563	3.152359	-1.026552
25	1	0	-1.156369	-2.369588	-1.917536
26	1	0	-1.538204	1.910577	-1.779415
27	1	0	0.917211	-3.317766	-1.343302
28	1	0	2.333925	-2.427759	-1.896575
29	1	0	2.493541	-1.462122	0.253207
30	1	0	1.461479	-2.722072	2.127244
31	1	0	0.237600	-3.132486	0.940417
32	8	0	-2.963483	0.320077	2.265895
33	8	0	-2.836986	-0.350086	-2.269160
34	1	0	2.503703	1.482015	-0.104649

Most stable energy, Gibbs free energy (Ha), and geometry for protomer ./OH_Py2N2//1_6
E: -912.128707
G: -911.884783
Geometry:

Input orientation:

Center Number	Atomic Number	Atomic Type	Coordinates (Angstroms) X	Y	Z
1	6	0	0.003266	-2.140505	1.489617
2	6	0	1.216663	-1.904971	0.757361
3	6	0	1.193183	-1.503789	-0.542538
4	7	0	0.003474	-1.368382	-1.176558
5	6	0	-1.186475	-1.505589	-0.543355
6	6	0	-1.209985	-1.906633	0.756583
7	6	0	-2.436094	-1.172702	-1.328631
8	7	0	-3.126901	0.059560	-0.957287
9	6	0	-2.433859	1.303067	-1.315113
10	6	0	-1.146417	1.575283	-0.566073
11	7	0	-0.003615	1.482443	-1.266062
12	6	0	1.139456	1.577125	-0.566765
13	6	0	1.188788	1.836449	0.787646
14	6	0	-0.003292	1.990663	1.546424
15	6	0	-1.195568	1.834546	0.788364
16	6	0	2.427216	1.308378	-1.316778
17	7	0	3.126652	0.069041	-0.956934
18	6	0	2.442475	-1.167408	-1.326604
19	1	0	2.168236	-2.027572	1.259860
20	1	0	-2.161669	-2.030747	1.258523
21	1	0	-3.133003	-2.000398	-1.197442
22	1	0	-2.188571	-1.117753	-2.390143
23	1	0	-3.133711	2.120833	-1.136507
24	1	0	-2.221439	1.273361	-2.384148
25	1	0	2.147766	1.901396	1.292140
26	1	0	-2.154398	1.897790	1.293328
27	1	0	2.213544	1.275147	-2.385463
28	1	0	3.123545	2.129808	-1.141142
29	1	0	3.294384	0.063769	0.045465
30	1	0	3.143036	-1.991493	-1.192305
31	1	0	2.196386	-1.116070	-2.388631

32	8	0	0.003106	-2.516866	2.691792
33	8	0	-0.003072	2.235954	2.806771
34	1	0	-3.294755	0.051954	0.045080
35	1	0	0.003520	-0.972072	-2.109228

Most stable energy, Gibbs free energy (Ha), and geometry for protomer ./OH_Py2N2//1_3
E: -912.118233
G: -911.875107
Geometry:

Input orientation:

Center Number	Atomic Number	Atomic Type	Coordinates (Angstroms) X	Y	Z
1	6	0	-2.180346	-1.503294	-0.490539
2	6	0	-2.389717	-0.342653	0.305483
3	6	0	-1.476142	0.036654	1.267806
4	7	0	-0.378709	-0.679599	1.555175
5	6	0	-0.124438	-1.732233	0.771906
6	6	0	-0.954613	-2.181645	-0.228045
7	6	0	1.192099	-2.423907	1.043833
8	7	0	2.216446	-1.428160	1.488081
9	6	0	2.994428	-0.759834	0.392030
10	6	0	2.084823	0.192593	-0.331780
11	7	0	1.600896	1.179294	0.437877
12	6	0	0.606971	1.907240	-0.085699
13	6	0	0.159955	1.759064	-1.386271
14	6	0	0.716570	0.777254	-2.247757
15	6	0	1.712124	-0.041274	-1.631938
16	6	0	-0.088532	2.885525	0.838747
17	7	0	-1.474352	2.563943	1.197934
18	6	0	-1.659024	1.348679	2.002477
19	1	0	-3.266932	0.268866	0.120900
20	1	0	-0.670924	-3.030751	-0.839070
21	1	0	1.570202	-2.936832	0.162381
22	1	0	1.087077	-3.144100	1.854734
23	1	0	3.387165	-1.539544	-0.255226
24	1	0	3.813473	-0.233488	0.878670
25	1	0	-0.650173	2.381756	-1.752308
26	1	0	2.133970	-0.873813	-2.182948
27	1	0	0.489334	2.962683	1.760018
28	1	0	-0.100668	3.871595	0.370659
29	1	0	-2.018131	2.471264	0.344253
30	1	0	-2.670169	1.382652	2.412470
31	1	0	-0.960089	1.387652	2.838448
32	8	0	-3.005040	-1.888809	-1.392682
33	8	0	0.339087	0.605146	-3.459661
34	1	0	2.867706	-1.872743	2.134757
35	1	0	1.695593	-0.693704	1.994909

Most stable energy, Gibbs free energy (Ha), and geometry for protomer ./OH_Py2N2//1_2
E: -912.123470
G: -911.882419
Geometry:

Input orientation:

Center Number	Atomic Number	Atomic Type	Coordinates (Angstroms) X	Y	Z
1	6	0	-2.320106	0.152132	-1.247533

2	6	0	-1.396162	1.135377	-1.573750
3	6	0	-0.058936	0.774287	-1.666818
4	7	0	0.360569	-0.478398	-1.493686
5	6	0	-0.520248	-1.399219	-1.098302
6	6	0	-1.876781	-1.136530	-0.981260
7	6	0	0.030045	-2.777450	-0.780950
8	7	0	1.331785	-2.824779	-0.115613
9	6	0	1.302194	-2.452580	1.307007
10	6	0	0.959303	-1.000794	1.544477
11	7	0	1.826618	-0.120748	1.025042
12	6	0	1.428386	1.158070	0.972386
13	6	0	0.258277	1.619392	1.537740
14	6	0	-0.642418	0.720792	2.175396
15	6	0	-0.233632	-0.641747	2.136284
16	6	0	2.279169	2.056871	0.106896
17	7	0	2.288275	1.666682	-1.311320
18	6	0	0.999379	1.814035	-1.986646
19	1	0	-1.709736	2.162648	-1.717009
20	1	0	-2.571545	-1.900233	-0.656420
21	1	0	0.114285	-3.321308	-1.726102
22	1	0	-0.695476	-3.317479	-0.170945
23	1	0	2.290923	-2.660862	1.720253
24	1	0	0.584788	-3.097283	1.815863
25	1	0	-0.017824	2.663946	1.441903
26	1	0	-0.902811	-1.404972	2.518609
27	1	0	1.930792	3.087823	0.177259
28	1	0	3.311352	2.027293	0.460761
29	1	0	2.533359	0.679865	-1.343763
30	1	0	1.176312	1.777740	-3.065358
31	1	0	0.600041	2.805249	-1.767028
32	8	0	-3.649465	0.410718	-1.133718
33	8	0	-1.757417	1.100196	2.688560
34	1	0	1.932772	-2.150817	-0.584158
35	1	0	-3.820128	1.343020	-1.326699

Most stable energy, Gibbs free energy (Ha), and geometry for protomer ./OH_Py2N2//1_1
E: -912.123465
G: -911.882561
Geometry:
Input orientation:

Center Number	Atomic Number	Atomic Type	Coordinates (Angstroms) X	Y	Z
1	6	0	0.627763	0.725391	2.175994
2	6	0	-0.273718	1.618064	1.531157
3	6	0	-1.438915	1.149833	0.961260
4	7	0	-1.831631	-0.130591	1.015518
5	6	0	-0.963082	-1.005162	1.542183
6	6	0	0.225032	-0.639004	2.139189
7	6	0	-1.297001	-2.459142	1.305923
8	7	0	-1.310084	-2.835410	-0.115910
9	6	0	-0.002395	-2.778447	-0.768709
10	6	0	0.537311	-1.396573	-1.088694
11	7	0	-0.348899	-0.485371	-1.493822
12	6	0	0.060898	0.770131	-1.670570
13	6	0	1.394311	1.143144	-1.571469
14	6	0	2.324648	0.169550	-1.234435
15	6	0	1.890835	-1.121456	-0.964365

16	6	0	-1.004628	1.799195	-2.000669
17	7	0	-2.293913	1.647089	-1.327150
18	6	0	-2.289893	2.042571	0.089565
19	1	0	-0.001764	2.663535	1.433482
20	1	0	0.895664	-1.398080	2.527202
21	1	0	-2.288932	-2.670970	1.709497
22	1	0	-0.581731	-3.098966	1.823859
23	1	0	-0.071365	-3.328552	-1.711498
24	1	0	0.722516	-3.307488	-0.148409
25	1	0	1.700026	2.172390	-1.717631
26	1	0	2.590098	-1.877476	-0.631282
27	1	0	-0.613478	2.795070	-1.787522
28	1	0	-1.179018	1.753463	-3.079420
29	1	0	-2.533691	0.658872	-1.356437
30	1	0	-3.322951	2.011209	0.440776
31	1	0	-1.944952	3.074876	0.157046
32	8	0	1.738793	1.111013	2.693119
33	8	0	3.650907	0.440499	-1.113424
34	1	0	-1.912278	-2.167804	-0.591977
35	1	0	3.814353	1.373352	-1.309931

Most stable energy, Gibbs free energy (Ha), and geometry for protomer ./OH_Py2N2//1_4
E: -912.118230
G: -911.875064
Geometry:
Input orientation:

Center Number	Atomic Number	Atomic Type	Coordinates (Angstroms) X	Y	Z
1	6	0	0.732138	0.786251	-2.247997
2	6	0	1.712624	-0.052023	-1.634600
3	6	0	2.083295	0.166247	-0.331087
4	7	0	1.610402	1.153658	0.444428
5	6	0	0.629361	1.900230	-0.077287
6	6	0	0.186353	1.769429	-1.381060
7	6	0	-0.055870	2.881130	0.852082
8	7	0	-1.446487	2.574900	1.206371
9	6	0	-1.648327	1.359037	2.005852
10	6	0	-1.478581	0.047622	1.267024
11	7	0	-0.392117	-0.683844	1.557470
12	6	0	-0.147222	-1.736433	0.770922
13	6	0	-0.977842	-2.171057	-0.235033
14	6	0	-2.193442	-1.475915	-0.501271
15	6	0	-2.391744	-0.315730	0.298140
16	6	0	1.159710	-2.444563	1.046093
17	7	0	2.196863	-1.460262	1.486272
18	6	0	2.980012	-0.802219	0.387760
19	1	0	2.124943	-0.885980	-2.190627
20	1	0	-0.612706	2.407048	-1.745688
21	1	0	-0.054664	3.870141	0.390090
22	1	0	0.520885	2.945383	1.775025
23	1	0	-2.660378	1.403656	2.412584
24	1	0	-0.951828	1.386546	2.844302
25	1	0	-0.701791	-3.021158	-0.848156
26	1	0	-3.259869	0.307975	0.111343
27	1	0	1.045059	-3.159447	1.860354
28	1	0	1.531348	-2.966373	0.167109
29	1	0	2.844470	-1.911803	2.131810

30	1	0	3.357976	-1.586629	-0.262595
31	1	0	3.809392	-0.289566	0.871610
32	8	0	0.357691	0.630346	-3.462992
33	8	0	-3.018062	-1.847190	-1.409302
34	1	0	-1.988912	2.492022	0.350812
35	1	0	1.686210	-0.719133	1.993582

Most stable energy, Gibbs free energy (Ha), and geometry for protomer ./OH_Py2N2//1_5

E: -912.132413

G: -911.889478

Geometry:

Input orientation:

Center Number	Atomic Number	Atomic Type	Coordinates (Angstroms) X	Y	Z
1	6	0	0.008569	2.437429	0.268869
2	6	0	1.202084	1.912246	-0.306344
3	6	0	1.141495	1.049492	-1.379610
4	7	0	-0.001441	0.669337	-1.965853
5	6	0	-1.139671	1.057404	-1.375656
6	6	0	-1.190568	1.920454	-0.302110
7	6	0	-2.385601	0.372058	-1.877106
8	7	0	-2.301086	-1.074468	-1.631062
9	6	0	-2.459700	-1.440360	-0.231642
10	6	0	-1.189272	-1.379782	0.585251
11	7	0	-0.004933	-1.486943	-0.062635
12	6	0	1.182265	-1.387931	0.581324
13	6	0	1.210215	-1.273680	1.937608
14	6	0	0.000607	-1.227259	2.706967
15	6	0	-1.211854	-1.265430	1.941659
16	6	0	2.449289	-1.457236	-0.240070
17	7	0	2.287835	-1.090180	-1.638823
18	6	0	2.381015	0.355715	-1.885374
19	1	0	2.158939	2.171740	0.132596
20	1	0	-2.144061	2.186511	0.140205
21	1	0	-2.475581	0.516721	-2.954369
22	1	0	-3.266892	0.811338	-1.395801
23	1	0	-2.815423	-2.471824	-0.192282
24	1	0	-3.205215	-0.823865	0.284782
25	1	0	2.162570	-1.193279	2.445851
26	1	0	-2.161919	-1.178537	2.453112
27	1	0	3.200984	-0.845992	0.273618
28	1	0	2.797960	-2.491161	-0.202135
29	1	0	3.019862	-1.549146	-2.165838
30	1	0	2.468145	0.499624	-2.962975
31	1	0	3.266916	0.789198	-1.407280
32	8	0	0.013140	3.265372	1.245739
33	8	0	0.003076	-1.129730	3.964791
34	1	0	-3.038318	-1.528472	-2.155121
35	1	0	-0.006260	-1.439592	-1.079728

Most stable energy, Gibbs free energy (Ha), and geometry for protomer ./OH_Py2N2//2_7

E: -912.584061

G: -912.327747

Geometry:

Input orientation:

Center Atomic Atomic Coordinates (Angstroms)

Number Number Type X Y Z

1	6	0	-0.049029	1.876884	1.639359
2	6	0	-1.200840	1.696651	0.823553
3	6	0	-1.087212	1.543122	-0.541910
4	7	0	0.084675	1.570762	-1.198840
5	6	0	1.192761	1.689371	-0.447589
6	6	0	1.178615	1.855965	0.921341
7	6	0	2.512827	1.502294	-1.157929
8	7	0	3.125465	0.179378	-0.974850
9	6	0	2.341876	-0.942531	-1.476337
10	6	0	1.134655	-1.347889	-0.658025
11	7	0	-0.082168	-1.254507	-1.226402
12	6	0	-1.239000	-1.513975	-0.591065
13	6	0	-1.191302	-1.994563	0.692952
14	6	0	0.050661	-2.155320	1.310434
15	6	0	1.220977	-1.818336	0.627569
16	6	0	-2.525200	-1.192327	-1.312697
17	7	0	-3.120249	0.098452	-0.984807
18	6	0	-2.330515	1.273974	-1.368468
19	1	0	-2.177403	1.665607	1.296343
20	1	0	2.113034	1.939979	1.466664
21	1	0	3.234255	2.237120	-0.800429
22	1	0	2.368457	1.659743	-2.226967
23	1	0	2.989409	-1.818875	-1.527526
24	1	0	2.020761	-0.713638	-2.493460
25	1	0	-2.107478	-2.222342	1.222619
26	1	0	2.184384	-1.919966	1.108734
27	1	0	-2.343616	-1.227586	-2.387968
28	1	0	-3.243761	-1.972596	-1.067199
29	1	0	-3.314404	0.130838	0.011824
30	1	0	-2.991212	2.140623	-1.306075
31	1	0	-2.038471	1.158098	-2.412796
32	8	0	-0.114081	2.027792	2.910830
33	8	0	0.175345	-2.619281	2.555555
34	1	0	3.323159	0.038485	0.012034
35	1	0	-0.136357	-0.843859	-2.155771
36	1	0	-0.690758	-2.820998	2.940613

Most stable energy, Gibbs free energy (Ha), and geometry for protomer ./OH_Py2N2//2_1

E: -912.593090

G: -912.340050

Geometry:

Input orientation:

Center Number	Atomic Number	Atomic Type	Coordinates (Angstroms) X	Y	Z
1	6	0	1.473976	0.053499	1.805648
2	6	0	0.761863	1.243304	1.718076
3	6	0	-0.616655	1.175048	1.602899
4	7	0	-1.289806	0.022333	1.621546
5	6	0	-0.594558	-1.115047	1.626750
6	6	0	0.787151	-1.152131	1.740992
7	6	0	-1.389426	-2.369955	1.355018
8	7	0	-2.045283	-2.376837	0.042069
9	6	0	-1.130560	-2.437882	-1.097330
10	6	0	-0.348186	-1.175359	-1.408164
11	7	0	-1.001242	-0.018721	-1.278314
12	6	0	-0.324612	1.118775	-1.429269

13	6	0	1.005436	1.151483	-1.824887
14	6	0	1.656725	-0.056149	-2.036536
15	6	0	0.978048	-1.245812	-1.804027
16	6	0	-1.078008	2.406874	-1.150718
17	7	0	-2.038270	2.376749	-0.048352
18	6	0	-1.433675	2.404710	1.289482
19	1	0	1.280917	2.192547	1.687902
20	1	0	1.323034	-2.093682	1.730775
21	1	0	-2.165799	-2.468238	2.115455
22	1	0	-0.740940	-3.243525	1.423365
23	1	0	-1.717730	-2.687315	-1.985208
24	1	0	-0.431079	-3.259804	-0.940014
25	1	0	1.533670	2.090975	-1.936642
26	1	0	1.487079	-2.196265	-1.898571
27	1	0	-0.359346	3.206827	-0.967951
28	1	0	-1.619295	2.672017	-2.063051
29	1	0	-2.564391	1.509822	-0.125879
30	1	0	-2.241513	2.491702	2.017414
31	1	0	-0.811019	3.295860	1.369620
32	8	0	2.825298	0.121376	1.893549
33	8	0	2.957229	-0.124058	-2.420844
34	1	0	-2.576854	-1.513422	-0.036268
35	1	0	3.201828	-0.769399	1.910380
36	1	0	3.313181	0.768188	-2.534744

Most stable energy, Gibbs free energy (Ha), and geometry for protomer ./OH_Py2N2//2_3
E: -912.586896
G: -912.330579
Geometry:

Input orientation:

Center Number	Atomic Number	Atomic Type	Coordinates (Angstroms) X	Y	Z
1	6	0	-0.710473	1.087818	-2.273173
2	6	0	-0.153840	1.983769	-1.370987
3	6	0	-0.477463	1.862449	-0.026030
4	7	0	-1.343734	0.949524	0.416782
5	6	0	-1.821959	0.058868	-0.449527
6	6	0	-1.546170	0.080110	-1.802898
7	6	0	-2.711584	-1.012878	0.132332
8	7	0	-2.278739	-1.372883	1.516492
9	6	0	-1.205676	-2.421200	1.606169
10	6	0	0.078113	-1.826980	1.101414
11	7	0	0.523310	-0.788773	1.826233
12	6	0	1.515317	-0.071093	1.284600
13	6	0	2.143180	-0.412915	0.101550
14	6	0	1.736698	-1.549319	-0.646457
15	6	0	0.626302	-2.250022	-0.083298
16	6	0	1.893625	1.218377	1.983936
17	7	0	1.619932	2.454905	1.243184
18	6	0	0.203644	2.745260	0.996686
19	1	0	0.539168	2.746174	-1.707922
20	1	0	-1.944411	-0.668535	-2.474723
21	1	0	-2.700667	-1.914906	-0.474769
22	1	0	-3.736062	-0.649736	0.207516
23	1	0	-1.534412	-3.275296	1.020092
24	1	0	-1.135596	-2.693584	2.657528
25	1	0	2.939764	0.213761	-0.286832

26	1	0	0.190220	-3.082257	-0.623534
27	1	0	1.360075	1.268143	2.933237
28	1	0	2.962739	1.207491	2.203199
29	1	0	2.107411	2.419705	0.352013
30	1	0	0.133795	3.781043	0.660827
31	1	0	-0.331407	2.661956	1.942514
32	8	0	-0.452720	1.129826	-3.601721
33	8	0	2.281894	-1.898245	-1.750694
34	1	0	-3.090529	-1.682409	2.052007
35	1	0	-1.911894	-0.514055	1.952693
36	1	0	0.160197	1.851647	-3.799776

Most stable energy, Gibbs free energy (Ha), and geometry for protomer ./OH_Py2N2//2_12
E: -912.583746
G: -912.330443
Geometry:
Input orientation:

Center Number	Atomic Number	Atomic Type	Coordinates (Angstroms) X	Y	Z
1	6	0	-3.973720	0.086701	0.443119
2	6	0	-3.200146	1.247883	0.503522
3	6	0	-1.857832	1.158344	0.228961
4	7	0	-1.326273	-0.028730	-0.101605
5	6	0	-2.035261	-1.165028	-0.151344
6	6	0	-3.381653	-1.134547	0.117774
7	6	0	-1.279857	-2.417197	-0.505434
8	7	0	-0.044720	-2.564472	0.258973
9	6	0	1.218483	-2.408947	-0.474412
10	6	0	1.963666	-1.134659	-0.152104
11	7	0	1.337400	0.021373	-0.464030
12	6	0	1.983808	1.171798	-0.213352
13	6	0	3.235489	1.219780	0.359441
14	6	0	3.928532	0.025894	0.701290
15	6	0	3.216774	-1.177579	0.403324
16	6	0	1.340478	2.468581	-0.678452
17	7	0	-0.094529	2.475792	-0.907393
18	6	0	-0.907580	2.316432	0.301502
19	1	0	-3.645628	2.198969	0.762830
20	1	0	-3.971640	-2.039622	0.087653
21	1	0	-1.078528	-2.390607	-1.582012
22	1	0	-1.941144	-3.261958	-0.322252
23	1	0	1.035314	-2.436719	-1.553558
24	1	0	1.867041	-3.250242	-0.235072
25	1	0	3.709623	2.177688	0.541764
26	1	0	3.678851	-2.131123	0.630012
27	1	0	1.585843	3.256387	0.034514
28	1	0	1.825893	2.743542	-1.617757
29	1	0	-0.330609	1.770655	-1.595024
30	1	0	-1.488179	3.216280	0.504941
31	1	0	-0.262425	2.147656	1.170503
32	8	0	-5.286564	0.092860	0.697284
33	8	0	5.089606	0.022328	1.231531
34	1	0	-0.051572	-3.472244	0.699566
35	1	0	-0.262497	-0.049729	-0.318052
36	1	0	-5.590752	0.986433	0.916120

Most stable energy, Gibbs free energy (Ha), and geometry for protomer ./OH_Py2N2//2_6
E: -912.587092

G: -912.328721
Geometry:
Input orientation:

Center Number	Atomic Number	Atomic Type	Coordinates (Angstroms) X	Y	Z
1	6	0	-1.968108	-1.673530	0.706911
2	6	0	-2.355443	-0.790735	-0.348941
3	6	0	-1.982177	0.529395	-0.316314
4	7	0	-1.286619	1.093930	0.681743
5	6	0	-0.835819	0.267479	1.628844
6	6	0	-1.135205	-1.073722	1.696468
7	6	0	0.069366	0.903965	2.657241
8	7	0	0.932011	1.944537	2.014554
9	6	0	2.241877	1.455069	1.471547
10	6	0	1.981849	0.534909	0.312425
11	7	0	1.285820	1.091416	-0.689826
12	6	0	0.835817	0.257532	-1.630771
13	6	0	1.136447	-1.083861	-1.688453
14	6	0	1.969961	-1.675508	-0.694516
15	6	0	2.356455	-0.784559	0.354760
16	6	0	-0.069990	0.885498	-2.663840
17	7	0	-0.934783	1.928851	-2.028603
18	6	0	-2.243708	1.440822	-1.481990
19	1	0	-2.904644	-1.184035	-1.195928
20	1	0	-0.716258	-1.689449	2.483523
21	1	0	0.713451	0.170826	3.137288
22	1	0	-0.519350	1.413149	3.419117
23	1	0	2.768309	0.959608	2.282845
24	1	0	2.792192	2.341381	1.159788
25	1	0	0.717993	-1.705824	-2.470850
26	1	0	2.905922	-1.171100	1.204683
27	1	0	0.518208	1.390690	-3.428764
28	1	0	-0.712492	0.148037	-3.139338
29	1	0	-1.118118	2.675279	-2.700051
30	1	0	-2.769324	0.938625	-2.289671
31	1	0	-2.795595	2.328374	-1.176569
32	8	0	-2.293128	-2.905774	0.728306
33	8	0	2.296216	-2.907556	-0.706660
34	1	0	1.113834	2.696215	2.680503
35	1	0	-0.386269	2.323247	-1.248924
36	1	0	0.382668	2.332055	1.231987

Most stable energy, Gibbs free energy (Ha), and geometry for protomer ./OH_Py2N2//2_10
E: -912.590672
G: -912.333194
Geometry:
Input orientation:

Center Number	Atomic Number	Atomic Type	Coordinates (Angstroms) X	Y	Z
1	6	0	-2.763072	0.257924	0.103008
2	6	0	-1.939287	1.278573	0.656018
3	6	0	-0.782250	0.940428	1.319677
4	7	0	-0.345100	-0.308626	1.505237
5	6	0	-1.066444	-1.289502	0.930572
6	6	0	-2.245587	-1.061882	0.259095

7	6	0	-0.527628	-2.701251	1.060961
8	7	0	0.896044	-2.907697	0.806995
9	6	0	1.339514	-2.642723	-0.558543
10	6	0	1.289350	-1.197431	-0.990487
11	7	0	1.999113	-0.327299	-0.229610
12	6	0	1.827765	1.015372	-0.331499
13	6	0	1.107146	1.540631	-1.349445
14	6	0	0.451047	0.680087	-2.306832
15	6	0	0.547815	-0.728270	-2.027853
16	6	0	2.350338	1.825513	0.811448
17	7	0	1.498189	1.595627	2.027074
18	6	0	0.075124	2.037289	1.913219
19	1	0	-2.216623	2.318934	0.532182
20	1	0	-2.779934	-1.890282	-0.191849
21	1	0	-1.089880	-3.351701	0.389722
22	1	0	-0.723525	-3.045446	2.079247
23	1	0	0.724650	-3.222566	-1.245778
24	1	0	2.369198	-2.992900	-0.656092
25	1	0	0.962710	2.610924	-1.412115
26	1	0	-0.025451	-1.421589	-2.629224
27	1	0	3.365082	1.535857	1.080291
28	1	0	2.314061	2.886766	0.584367
29	1	0	1.940740	2.070994	2.815735
30	1	0	0.046196	2.942749	1.311058
31	1	0	-0.255063	2.274230	2.923814
32	8	0	-3.856449	0.504342	-0.512386
33	8	0	-0.210602	1.136546	-3.266574
34	1	0	1.436038	-2.358254	1.467244
35	1	0	2.520791	-0.687670	0.561712
36	1	0	1.464260	0.587714	2.242677

Most stable energy, Gibbs free energy (Ha), and geometry for protomer ./OH_Py2N2//2_2

E: -912.586892

G: -912.330443

Geometry:

Input orientation:

Center Number	Atomic Number	Atomic Type	Coordinates (Angstroms) X	Y	Z
1	6	0	-1.762497	-1.508367	-0.668210
2	6	0	-2.150861	-0.368060	0.083510
3	6	0	-1.524591	-0.046029	1.272923
4	7	0	-0.551353	-0.787074	1.817223
5	6	0	-0.122560	-1.830153	1.089565
6	6	0	-0.671049	-2.235082	-0.101377
7	6	0	1.144689	-2.454681	1.599433
8	7	0	2.241645	-1.430380	1.521780
9	6	0	2.688721	-1.070767	0.142073
10	6	0	1.825742	0.023623	-0.437693
11	7	0	1.361870	0.919144	0.431363
12	6	0	0.517187	1.852430	-0.010729
13	6	0	0.203616	1.988463	-1.356652
14	6	0	0.745770	1.086018	-2.261139
15	6	0	1.557602	0.058325	-1.792261
16	6	0	-0.151506	2.742936	1.013481
17	7	0	-1.575611	2.482092	1.247572
18	6	0	-1.881507	1.246707	1.977576
19	1	0	-2.931560	0.277114	-0.306817

20	1	0	-0.248549	-3.073289	-0.643169
21	1	0	1.458026	-3.313086	1.011208
22	1	0	1.062131	-2.730535	2.648994
23	1	0	2.661175	-1.968535	-0.470827
24	1	0	3.720524	-0.730575	0.224486
25	1	0	-0.471723	2.766984	-1.692679
26	1	0	1.943050	-0.695042	-2.466261
27	1	0	0.374648	2.641036	1.962484
28	1	0	-0.056403	3.779312	0.685669
29	1	0	-2.057058	2.463607	0.352617
30	1	0	-2.952398	1.257018	2.187988
31	1	0	-1.354960	1.279040	2.931520
32	8	0	-2.307210	-1.839832	-1.778066
33	8	0	0.495406	1.140194	-3.590728
34	1	0	3.043958	-1.762473	2.058130
35	1	0	1.892933	-0.566892	1.963300
36	1	0	-0.104722	1.872968	-3.787821

Most stable energy, Gibbs free energy (Ha), and geometry for protomer ./OH_Py2N2//2_5
E: -912.586895
G: -912.330456
Geometry:
Input orientation:

Center Number	Atomic Number	Atomic Type	Coordinates (Angstroms) X	Y	Z
1	6	0	-0.731961	-1.090050	-2.263311
2	6	0	-1.553550	-0.069903	-1.794861
3	6	0	-1.824542	-0.038986	-0.440756
4	7	0	-1.354196	-0.931231	0.428292
5	6	0	-0.500506	-1.856510	-0.013233
6	6	0	-0.183339	-1.988483	-1.358771
7	6	0	0.174237	-2.742341	1.011105
8	7	0	1.595306	-2.468490	1.248803
9	6	0	1.888138	-1.231210	1.980805
10	6	0	1.521546	0.058909	1.276379
11	7	0	0.540965	0.791128	1.819233
12	6	0	0.104717	1.831110	1.091482
13	6	0	0.652089	2.241597	-0.098026
14	6	0	1.750672	1.524396	-0.663392
15	6	0	2.147278	0.386999	0.088305
16	6	0	-1.169126	2.443956	1.599023
17	7	0	-2.256196	1.409231	1.519130
18	6	0	-2.698484	1.046837	0.138534
19	1	0	-1.944522	0.680665	-2.468792
20	1	0	0.499154	-2.760935	-1.694287
21	1	0	0.089703	-3.779031	0.681395
22	1	0	-0.354922	-2.647041	1.959122
23	1	0	2.958505	-1.232098	2.194229
24	1	0	1.359303	-1.269114	2.933283
25	1	0	0.223438	3.076443	-0.640177
26	1	0	2.934055	-0.251417	-0.300930
27	1	0	-1.091262	2.720540	2.648736
28	1	0	-1.489382	3.299401	1.010153
29	1	0	-3.062109	1.733123	2.055095
30	1	0	-2.678513	1.945413	-0.473411
31	1	0	-3.727144	0.696969	0.219486
32	8	0	-0.479172	-1.141068	-3.592554

33	8	0	2.294381	1.861140	-1.772195
34	1	0	2.078639	-2.444800	0.354995
35	1	0	-1.899672	0.548662	1.960087
36	1	0	0.126521	-1.869335	-3.789290

Most stable energy, Gibbs free energy (Ha), and geometry for protomer ./OH_Py2N2//2_8
E: -912.600876
G: -912.345142
Geometry:
Input orientation:

Center Number	Atomic Number	Atomic Type	Coordinates (Angstroms) X	Y	Z
1	6	0	1.708499	-1.942910	-0.076936
2	6	0	2.196009	-0.770599	-0.638801
3	6	0	1.329807	0.007782	-1.390887
4	7	0	0.063935	-0.347142	-1.633363
5	6	0	-0.409108	-1.451817	-1.054699
6	6	0	0.377042	-2.285583	-0.273156
7	6	0	-1.868696	-1.787806	-1.301385
8	7	0	-2.777485	-0.657825	-1.466633
9	6	0	-3.152685	0.010194	-0.219159
10	6	0	-1.990559	0.528724	0.587217
11	7	0	-1.281995	1.549118	0.037354
12	6	0	-0.074961	1.934181	0.513985
13	6	0	0.395525	1.394428	1.671084
14	6	0	-0.336560	0.378924	2.373756
15	6	0	-1.564924	-0.035482	1.746315
16	6	0	0.720109	2.914222	-0.319511
17	7	0	1.956477	2.397725	-0.899802
18	6	0	1.792886	1.349765	-1.913748
19	1	0	3.219933	-0.462909	-0.466931
20	1	0	-0.040501	-3.171091	0.191343
21	1	0	-2.232949	-2.413481	-0.485232
22	1	0	-1.913830	-2.395736	-2.208767
23	1	0	-3.707719	-0.691661	0.401651
24	1	0	-3.816675	0.839126	-0.468279
25	1	0	1.360803	1.706570	2.050654
26	1	0	-2.125841	-0.854433	2.178066
27	1	0	0.090755	3.287508	-1.129173
28	1	0	0.977974	3.763136	0.313468
29	1	0	2.552998	2.049348	-0.154414
30	1	0	2.757192	1.222056	-2.406979
31	1	0	1.078503	1.704605	-2.656486
32	8	0	2.555429	-2.696584	0.667846
33	8	0	0.082391	-0.143342	3.438230
34	1	0	-2.337706	0.016533	-2.085886
35	1	0	-1.567945	1.897229	-0.871385
36	1	0	2.090921	-3.467289	1.022758

Most stable energy, Gibbs free energy (Ha), and geometry for protomer ./OH_Py2N2//2_4
E: -912.586893
G: -912.330471
Geometry:
Input orientation:

Center Number	Atomic Number	Atomic Type	Coordinates (Angstroms) X	Y	Z

1	6	0	-1.753631	1.521007	-0.663118
2	6	0	-0.655936	2.240112	-0.098489
3	6	0	-0.107567	1.831061	1.091057
4	7	0	-0.541780	0.790631	1.819367
5	6	0	-1.521255	0.056450	1.277044
6	6	0	-2.148061	0.383200	0.089170
7	6	0	-1.885323	-1.234133	1.981956
8	7	0	-1.590294	-2.471195	1.250297
9	6	0	-0.168725	-2.742258	1.012275
10	6	0	0.503865	-1.855763	-0.012874
11	7	0	1.355796	-0.928512	0.427911
12	6	0	1.824024	-0.035690	-0.441618
13	6	0	1.552770	-0.067872	-1.795628
14	6	0	0.733024	-1.089809	-2.263361
15	6	0	0.186473	-1.988940	-1.358222
16	6	0	2.696070	1.052148	0.136936
17	7	0	2.254377	1.414116	1.517805
18	6	0	1.165180	2.446412	1.598304
19	1	0	-0.228881	3.075496	-0.641075
20	1	0	-2.934072	-0.256503	-0.299511
21	1	0	-2.955657	-1.236975	2.195403
22	1	0	-1.356304	-1.270782	2.934373
23	1	0	-0.082057	-3.779001	0.683314
24	1	0	0.360592	-2.645075	1.960012
25	1	0	1.942122	0.683054	-2.470119
26	1	0	-0.494784	-2.762734	-1.693188
27	1	0	3.725563	0.704511	0.217064
28	1	0	2.673722	1.950483	-0.475336
29	1	0	3.060111	1.739902	2.052859
30	1	0	1.483417	3.302703	1.009632
31	1	0	1.086910	2.722441	2.648144
32	8	0	-2.298419	1.856482	-1.771725
33	8	0	0.479916	-1.141758	-3.592491
34	1	0	-2.073918	-2.448556	0.356617
35	1	0	1.900012	0.552936	1.959325
36	1	0	-0.124318	-1.871307	-3.788967

Most stable energy, Gibbs free energy (Ha), and geometry for protomer ./OH_Py2N2//2_14
E: -912.590672
G: -912.333067
Geometry:
Input orientation:

Center Number	Atomic Number	Atomic Type	Coordinates (Angstroms) X	Y	Z
1	6	0	-0.434342	0.678548	2.309082
2	6	0	-1.094186	1.543009	1.357808
3	6	0	-1.823234	1.021967	0.343723
4	7	0	-1.999900	-0.319936	0.240631
5	6	0	-1.287879	-1.193781	0.995118
6	6	0	-0.537701	-0.728960	2.028215
7	6	0	-1.345806	-2.638226	0.561321
8	7	0	-0.912784	-2.903022	-0.807568
9	6	0	0.509618	-2.701003	-1.071790
10	6	0	1.055148	-1.291935	-0.940498
11	7	0	0.334408	-0.305936	-1.507223
12	6	0	0.778241	0.940523	-1.320365
13	6	0	1.941029	1.271458	-0.663044

14	6	0	2.763823	0.245401	-0.118812
15	6	0	2.239544	-1.071621	-0.275998
16	6	0	-0.078069	2.043233	-1.904692
17	7	0	-1.502901	1.606521	-2.014664
18	6	0	-2.349504	1.835630	-0.794995
19	1	0	-0.945712	2.612697	1.421403
20	1	0	0.037330	-1.425269	2.624429
21	1	0	-2.375927	-2.985182	0.665581
22	1	0	-0.728095	-3.221066	1.243479
23	1	0	0.696208	-3.042400	-2.092755
24	1	0	1.074577	-3.355935	-0.407202
25	1	0	2.223707	2.310196	-0.537655
26	1	0	2.773252	-1.903805	0.168686
27	1	0	-0.043668	2.945419	-1.297891
28	1	0	0.248705	2.284479	-2.915364
29	1	0	-1.472636	0.598947	-2.232774
30	1	0	-3.365954	1.548448	-1.060007
31	1	0	-2.310154	2.896347	-0.565930
32	8	0	0.235501	1.131020	3.265019
33	8	0	3.862255	0.484679	0.490348
34	1	0	-1.456066	-2.351978	-1.463672
35	1	0	-2.527571	-0.676944	-0.548255
36	1	0	-1.946977	2.085282	-2.800376

Most stable energy, Gibbs free energy (Ha), and geometry for protomer ./OH_Py2N2//2_11
E: -912.600876
G: -912.345088
Geometry:
Input orientation:

Center Number	Atomic Number	Atomic Type	Coordinates (Angstroms) X	Y	Z
1	6	0	-0.338405	0.374684	-2.373531
2	6	0	0.389622	1.394012	-1.672189
3	6	0	-0.082937	1.933283	-0.515706
4	7	0	-1.288394	1.544010	-0.038513
5	6	0	-1.992942	0.520161	-0.587137
6	6	0	-1.565124	-0.043780	-1.745563
7	6	0	-3.152851	-0.002067	0.220031
8	7	0	-2.774607	-0.667606	1.467935
9	6	0	-1.861403	-1.794113	1.303257
10	6	0	-0.403179	-1.452545	1.056172
11	7	0	0.065474	-0.345414	1.633705
12	6	0	1.329881	0.014383	1.390729
13	6	0	2.199082	-0.761180	0.639215
14	6	0	1.716188	-1.935890	0.078367
15	6	0	0.386211	-2.283899	0.275293
16	6	0	1.787671	1.358764	1.912111
17	7	0	1.946947	2.406288	0.897013
18	6	0	0.708450	2.917384	0.316479
19	1	0	1.353632	1.709517	-2.052203
20	1	0	-2.122793	-0.865496	-2.176271
21	1	0	-3.705453	-0.706559	-0.399957
22	1	0	-3.819858	0.824565	0.468720
23	1	0	-2.223303	-2.421663	0.487498
24	1	0	-1.904048	-2.401702	2.210988
25	1	0	3.221694	-0.449448	0.466809
26	1	0	-0.027842	-3.171484	-0.188366

27	1	0	1.072048	1.711586	2.654612
28	1	0	2.752557	1.235389	2.405304
29	1	0	2.544566	2.059390	0.151813
30	1	0	0.962913	3.766539	-0.317555
31	1	0	0.077906	3.289202	1.125880
32	8	0	0.082634	-0.147329	-3.437306
33	8	0	2.566045	-2.686504	-0.666178
34	1	0	-2.337253	0.009013	2.086446
35	1	0	-1.575766	1.892097	0.869803
36	1	0	2.104487	-3.458899	-1.021230

Most stable energy, Gibbs free energy (Ha), and geometry for protomer ./OH_Py2N2//2_15
E: -912.609687
G: -912.354084
Geometry:
Input orientation:

Center Number	Atomic Number	Atomic Type	Coordinates (Angstroms) X	Y	Z
1	6	0	-2.034677	-0.040272	1.817887
2	6	0	-1.900929	1.166804	1.044620
3	6	0	-1.636515	1.121306	-0.286847
4	7	0	-1.525935	-0.077601	-0.908862
5	6	0	-1.524250	-1.251735	-0.233134
6	6	0	-1.785177	-1.262620	1.099963
7	6	0	-1.211745	-2.482060	-1.043296
8	7	0	-0.091382	-2.265830	-1.956885
9	6	0	1.218959	-2.376542	-1.317244
10	6	0	1.567602	-1.134574	-0.542065
11	7	0	1.357203	0.044112	-1.177041
12	6	0	1.461170	1.239169	-0.547424
13	6	0	1.936119	1.291869	0.724259
14	6	0	2.305695	0.092578	1.429373
15	6	0	2.045354	-1.138474	0.729184
16	6	0	1.008837	2.442308	-1.331665
17	7	0	-0.260798	2.201174	-2.015237
18	6	0	-1.430988	2.337898	-1.149230
19	1	0	-1.978003	2.123942	1.543146
20	1	0	-1.772045	-2.199625	1.640789
21	1	0	-2.089415	-2.729545	-1.643398
22	1	0	-1.036196	-3.308994	-0.348065
23	1	0	1.963677	-2.515232	-2.103205
24	1	0	1.284160	-3.230000	-0.635106
25	1	0	2.008256	2.245488	1.230297
26	1	0	2.202427	-2.079468	1.239619
27	1	0	0.964671	3.295143	-0.646988
28	1	0	1.761358	2.660581	-2.091618
29	1	0	-0.346748	2.864703	-2.774306
30	1	0	-2.307067	2.459306	-1.789001
31	1	0	-1.369571	3.211012	-0.492018
32	8	0	-2.300389	-0.024577	3.045336
33	8	0	2.776047	0.115314	2.593546
34	1	0	-0.144379	-2.953811	-2.696922
35	1	0	-1.264355	-0.090386	-1.888268
36	1	0	0.943337	0.025850	-2.102556

Most stable energy, Gibbs free energy (Ha), and geometry for protomer ./OH_Py2N2//2_9
E: -912.592018
G: -912.334093

Geometry:

Input orientation:

Center Number	Atomic Number	Atomic Type	Coordinates (Angstroms) X	Y	Z
1	6	0	-2.224258	-1.520875	-0.053304
2	6	0	-2.482590	-0.157960	0.287652
3	6	0	-1.825201	0.869807	-0.346686
4	7	0	-0.942471	0.672415	-1.343830
5	6	0	-0.629370	-0.594564	-1.623174
6	6	0	-1.210461	-1.695833	-1.036856
7	6	0	0.459920	-0.776682	-2.657205
8	7	0	1.377107	0.402317	-2.632573
9	6	0	2.519009	0.322904	-1.661864
10	6	0	2.038108	0.128599	-0.257752
11	7	0	1.338541	1.151084	0.294883
12	6	0	0.651048	1.005749	1.448703
13	6	0	0.791756	-0.129764	2.185727
14	6	0	1.635130	-1.202978	1.740274
15	6	0	2.224012	-1.021770	0.432935
16	6	0	-0.249205	2.159989	1.820203
17	7	0	-0.753712	2.855480	0.640931
18	6	0	-2.008437	2.293485	0.117598
19	1	0	-3.178299	0.056790	1.090335
20	1	0	-0.885626	-2.695036	-1.302491
21	1	0	0.039304	-0.822367	-3.660793
22	1	0	1.045494	-1.675265	-2.475063
23	1	0	3.078009	1.251701	-1.765231
24	1	0	3.139622	-0.515655	-1.963865
25	1	0	0.233811	-0.243203	3.106114
26	1	0	2.780117	-1.835039	-0.013669
27	1	0	-1.044604	1.772422	2.466023
28	1	0	0.337128	2.866430	2.410620
29	1	0	-0.921028	3.823058	0.886335
30	1	0	-2.322105	2.916540	-0.721388
31	1	0	-2.799637	2.314980	0.874333
32	8	0	-2.820374	-2.497975	0.512914
33	8	0	1.809321	-2.252713	2.404044
34	1	0	0.777810	1.212122	-2.408788
35	1	0	1.091596	1.967590	-0.258201
36	1	0	1.773310	0.550242	-3.562169

Most stable energy, Gibbs free energy (Ha), and geometry for protomer ./OH_Py2N2//3_19

E: -913.055254

G: -912.783286

Geometry:

Input orientation:

Center Number	Atomic Number	Atomic Type	Coordinates (Angstroms) X	Y	Z
1	6	0	-0.353333	-2.176476	-1.627008
2	6	0	0.993125	-1.873000	-1.228320
3	6	0	1.280852	-1.470048	0.038140
4	7	0	0.289938	-1.363784	0.953338
5	6	0	-1.013657	-1.554559	0.633021
6	6	0	-1.356879	-1.973287	-0.609744
7	6	0	-2.025549	-1.254726	1.701969

8	7	0	-2.804970	-0.002731	1.414511
9	6	0	-2.123966	1.303729	1.697743
10	6	0	-1.006220	1.568914	0.729946
11	7	0	0.257596	1.532842	1.220206
12	6	0	1.342624	1.583182	0.402586
13	6	0	1.180706	1.805734	-0.925763
14	6	0	-0.128431	1.946485	-1.510412
15	6	0	-1.226557	1.790733	-0.589435
16	6	0	2.665084	1.265759	1.038896
17	7	0	2.657392	-0.103389	1.562318
18	6	0	2.668744	-1.126664	0.521186
19	1	0	1.788053	-1.942502	-1.959265
20	1	0	-2.399388	-2.132532	-0.855129
21	1	0	-1.559963	-1.124207	2.677239
22	1	0	-2.756490	-2.057824	1.755380
23	1	0	-1.771075	1.263167	2.725925
24	1	0	-2.891249	2.069446	1.608851
25	1	0	2.048829	1.826179	-1.571217
26	1	0	-2.241302	1.821032	-0.966556
27	1	0	3.447852	1.422238	0.291277
28	1	0	2.841819	1.945603	1.873402
29	1	0	3.474523	-0.224150	2.146917
30	1	0	3.109840	-2.032086	0.941461
31	1	0	3.266507	-0.841142	-0.350609
32	8	0	-0.648064	-2.564024	-2.781894
33	8	0	-0.303500	2.147004	-2.731931
34	1	0	-3.650178	-0.030722	1.993446
35	1	0	0.544594	-1.020682	1.874522
36	1	0	0.401929	1.317099	2.201319
37	1	0	-3.123833	-0.017167	0.438627

Most stable energy, Gibbs free energy (Ha), and geometry for protomer ./OH_Py2N2//3_16
E: -913.043826
G: -912.771397
Geometry:

Input orientation:

Center Number	Atomic Number	Atomic Type	Coordinates (Angstroms) X	Y	Z
1	6	0	-0.734258	-0.620061	2.261851
2	6	0	-1.767268	0.076540	1.532011
3	6	0	-2.090462	-0.278978	0.265857
4	7	0	-1.484464	-1.339008	-0.328607
5	6	0	-0.438318	-1.970098	0.255843
6	6	0	-0.064132	-1.659200	1.522219
7	6	0	0.295446	-2.979190	-0.578841
8	7	0	1.647352	-2.484404	-0.991668
9	6	0	1.684042	-1.311139	-1.935813
10	6	0	1.446626	-0.002696	-1.225195
11	7	0	0.282796	0.610528	-1.488434
12	6	0	0.014328	1.705674	-0.770783
13	6	0	0.864523	2.261167	0.155833
14	6	0	2.138442	1.671683	0.401145
15	6	0	2.385537	0.478384	-0.344254
16	6	0	-1.333648	2.338895	-1.032963
17	7	0	-2.282345	1.330440	-1.593950
18	6	0	-3.033779	0.511978	-0.584816
19	1	0	-2.253852	0.926673	1.991129

20	1	0	0.768522	-2.180283	1.977860
21	1	0	-0.246797	-3.221079	-1.490155
22	1	0	0.464086	-3.888924	-0.007663
23	1	0	0.928389	-1.491822	-2.696045
24	1	0	2.672673	-1.335604	-2.388965
25	1	0	0.566572	3.140104	0.715334
26	1	0	3.315604	-0.057745	-0.189013
27	1	0	-1.768611	2.752198	-0.125466
28	1	0	-1.247228	3.131469	-1.775122
29	1	0	-1.721671	0.708547	-2.195304
30	1	0	-3.707063	-0.136056	-1.143532
31	1	0	-3.607007	1.202641	0.026549
32	8	0	-0.420559	-0.315896	3.430950
33	8	0	2.978669	2.153028	1.229219
34	1	0	2.123368	-3.269265	-1.445555
35	1	0	-1.696659	-1.549906	-1.298195
36	1	0	-2.971128	1.801810	-2.183472
37	1	0	2.196130	-2.258716	-0.154461

Most stable energy, Gibbs free energy (Ha), and geometry for protomer ./OH_Py2N2//3_1
E: -913.052389
G: -912.784176
Geometry:

Input orientation:

Center Number	Atomic Number	Atomic Type	Coordinates (Angstroms) X	Y	Z
1	6	0	-1.828043	1.640206	-0.809012
2	6	0	-2.281291	0.724867	0.131167
3	6	0	-1.458125	0.406882	1.200807
4	7	0	-0.269594	0.988843	1.382854
5	6	0	0.175573	1.821968	0.446424
6	6	0	-0.557244	2.188614	-0.666595
7	6	0	1.570557	2.360024	0.653370
8	7	0	2.439126	1.339348	1.316044
9	6	0	3.092016	0.346689	0.398239
10	6	0	2.036292	-0.556632	-0.169667
11	7	0	1.388153	-1.299418	0.733932
12	6	0	0.298483	-1.951065	0.336836
13	6	0	-0.124855	-1.961690	-0.988952
14	6	0	0.598095	-1.242833	-1.925699
15	6	0	1.701012	-0.500089	-1.504514
16	6	0	-0.541908	-2.630864	1.394943
17	7	0	-1.863285	-2.043289	1.627392
18	6	0	-1.868064	-0.680600	2.169506
19	1	0	-3.246918	0.249919	0.010221
20	1	0	-0.151255	2.859036	-1.413887
21	1	0	1.545929	3.226710	1.313025
22	1	0	2.035434	2.645343	-0.287752
23	1	0	3.802941	-0.210136	1.005265
24	1	0	3.616889	0.906765	-0.370853
25	1	0	-1.016732	-2.505159	-1.280762
26	1	0	2.243711	0.127284	-2.198918
27	1	0	-0.694821	-3.672845	1.110229
28	1	0	0.009926	-2.616391	2.334254
29	1	0	-2.395514	-2.059655	0.761700
30	1	0	-1.201835	-0.653441	3.031406
31	1	0	-2.879737	-0.470460	2.519479

32	8	0	-2.633193	1.938231	-1.854346
33	8	0	0.262521	-1.191137	-3.234166
34	1	0	1.845385	0.821764	1.980446
35	1	0	3.168142	1.816339	1.849203
36	1	0	-2.196796	2.569245	-2.444003
37	1	0	-0.522939	-1.732074	-3.398242

Most stable energy, Gibbs free energy (Ha), and geometry for protomer ./OH_Py2N2//3_7
E: -913.056781
G: -912.786508
Geometry:

Input orientation:

Center Number	Atomic Number	Atomic Type	Coordinates (Angstroms) X	Y	Z
1	6	0	-2.429290	-0.975848	-0.324066
2	6	0	-2.452831	0.403667	-0.130444
3	6	0	-1.462302	1.171788	-0.710427
4	7	0	-0.506429	0.639418	-1.484021
5	6	0	-0.461611	-0.679716	-1.611619
6	6	0	-1.396225	-1.540569	-1.058374
7	6	0	0.692055	-1.235539	-2.412850
8	7	0	1.856133	-0.302337	-2.364320
9	6	0	2.790223	-0.481529	-1.200191
10	6	0	2.065008	-0.344707	0.101624
11	7	0	1.599582	0.891775	0.412536
12	6	0	0.708905	1.087188	1.412156
13	6	0	0.394873	0.065454	2.252360
14	6	0	0.964887	-1.242632	2.079096
15	6	0	1.797945	-1.399592	0.908213
16	6	0	0.107226	2.468205	1.487850
17	7	0	-0.101867	3.033326	0.158175
18	6	0	-1.385676	2.653977	-0.442220
19	1	0	-3.214425	0.851888	0.493427
20	1	0	-1.314617	-2.613890	-1.178235
21	1	0	0.412035	-1.337040	-3.460621
22	1	0	1.010943	-2.205323	-2.036536
23	1	0	3.566726	0.274027	-1.308571
24	1	0	3.223509	-1.473398	-1.287697
25	1	0	-0.325196	0.225401	3.044160
26	1	0	2.168510	-2.382702	0.650558
27	1	0	-0.812801	2.405105	2.078879
28	1	0	0.803204	3.113239	2.027182
29	1	0	-0.077039	4.042603	0.231040
30	1	0	-1.483999	3.193252	-1.385628
31	1	0	-2.229341	2.932166	0.197943
32	8	0	-3.402130	-1.710981	0.255749
33	8	0	0.712945	-2.199409	2.846782
34	1	0	1.470421	0.652340	-2.358969
35	1	0	1.694989	1.655696	-0.250907
36	1	0	2.407301	-0.406234	-3.219087
37	1	0	-3.269134	-2.651863	0.073551

Most stable energy, Gibbs free energy (Ha), and geometry for protomer ./OH_Py2N2//3_14
E: -913.056785
G: -912.786559
Geometry:

Input orientation:

Center Number	Atomic Number	Atomic Type	Coordinates (Angstroms) X	Y	Z
1	6	0	-0.962227	1.241933	-2.081797
2	6	0	-1.794102	1.403779	-0.910744
3	6	0	-2.063071	0.351525	-0.101344
4	7	0	-1.600654	-0.886749	-0.409424
5	6	0	-0.711410	-1.086864	-1.409371
6	6	0	-0.395565	-0.067952	-2.252344
7	6	0	-0.113578	-2.469732	-1.482482
8	7	0	0.092775	-3.033679	-0.151904
9	6	0	1.377635	-2.658021	0.448713
10	6	0	1.459111	-1.175548	0.713653
11	7	0	0.505819	-0.638749	1.487288
12	6	0	0.464385	0.680882	1.611275
13	6	0	1.400049	1.537974	1.053976
14	6	0	2.430403	0.968673	0.319264
15	6	0	2.450516	-0.411458	0.129896
16	6	0	-0.687044	1.241285	2.412508
17	7	0	-1.854409	0.312073	2.364786
18	6	0	-2.787665	0.493068	1.200268
19	1	0	-2.162206	2.388385	-0.655294
20	1	0	0.323427	-0.231593	-3.044372
21	1	0	0.807088	-2.410166	-2.072881
22	1	0	-0.810965	-3.113455	-2.021559
23	1	0	2.220682	-2.940641	-0.190312
24	1	0	1.473203	-3.195700	1.393296
25	1	0	1.321006	2.611811	1.170719
26	1	0	3.209866	-0.863237	-0.494142
27	1	0	-0.406525	1.342348	3.460183
28	1	0	-1.002672	2.211927	2.035664
29	1	0	-2.405420	0.419060	3.219275
30	1	0	-3.217172	1.486715	1.286233
31	1	0	-3.567063	-0.259340	1.309787
32	8	0	-0.708485	2.196256	-2.851967
33	8	0	3.403555	1.699542	-0.265349
34	1	0	0.064332	-4.042962	-0.223318
35	1	0	-1.697813	-1.649133	0.255512
36	1	0	-1.472119	-0.643983	2.360932
37	1	0	3.272505	2.641540	-0.087697

Most stable energy, Gibbs free energy (Ha), and geometry for protomer ./OH_Py2N2//3_5
E: -913.053017
G: -912.786836
Geometry:
Input orientation:

Center Number	Atomic Number	Atomic Type	Coordinates (Angstroms) X	Y	Z
1	6	0	-3.966494	0.091965	-0.434581
2	6	0	-3.178198	1.243068	-0.478349
3	6	0	-1.852989	1.132522	-0.115200
4	7	0	-1.326500	-0.025870	0.297817
5	6	0	-2.068760	-1.127980	0.327782
6	6	0	-3.405903	-1.113405	-0.040234
7	6	0	-1.421111	-2.419610	0.774420
8	7	0	-0.000797	-2.294378	1.063457
9	6	0	0.866756	-2.283827	-0.114808

10	6	0	1.849325	-1.141883	-0.098661
11	7	0	1.350944	0.056250	0.263282
12	6	0	2.062130	1.186109	0.264546
13	6	0	3.389931	1.142661	-0.104136
14	6	0	3.946742	-0.079839	-0.465825
15	6	0	3.164080	-1.241489	-0.462852
16	6	0	1.394973	2.471028	0.712092
17	7	0	-0.028446	2.441346	0.962456
18	6	0	-0.894465	2.294193	-0.212304
19	1	0	-3.597518	2.190156	-0.793622
20	1	0	-4.003367	-2.015404	-0.014506
21	1	0	-1.633355	-3.179599	0.012159
22	1	0	-1.923530	-2.745864	1.686487
23	1	0	0.260763	-2.172140	-1.019032
24	1	0	1.425494	-3.213558	-0.221327
25	1	0	3.986776	2.045810	-0.106603
26	1	0	3.592505	-2.192826	-0.744933
27	1	0	1.909246	2.781489	1.623865
28	1	0	1.616009	3.232541	-0.037064
29	1	0	-0.257761	1.760839	1.676892
30	1	0	-0.282335	2.135797	-1.106976
31	1	0	-1.468419	3.205724	-0.383277
32	8	0	-5.276142	0.105617	-0.767505
33	8	0	5.229175	-0.206530	-0.826528
34	1	0	0.271420	-3.061457	1.662748
35	1	0	0.300900	0.060298	0.463958
36	1	0	-5.550422	1.000462	-1.012801
37	1	0	5.685661	0.647079	-0.789376

Most stable energy, Gibbs free energy (Ha), and geometry for protomer ./OH_Py2N2//3_2
E: -913.052402
G: -912.784048
Geometry:

Input orientation:

Center Number	Atomic Number	Atomic Type	Coordinates (Angstroms) X	Y	Z
1	6	0	1.922605	-1.534492	-0.836241
2	6	0	0.679987	-2.146080	-0.705995
3	6	0	-0.076523	-1.823575	0.405400
4	7	0	0.322276	-0.976862	1.349787
5	6	0	1.482218	-0.335686	1.180078
6	6	0	2.325890	-0.605384	0.113728
7	6	0	1.832255	0.762572	2.160173
8	7	0	1.767602	2.127775	1.627687
9	6	0	0.421322	2.659088	1.402611
10	6	0	-0.398821	1.936634	0.357162
11	7	0	-1.453303	1.238159	0.769496
12	6	0	-2.076109	0.461528	-0.123686
13	6	0	-1.752365	0.413377	-1.461791
14	6	0	-0.687485	1.201413	-1.898239
15	6	0	0.011303	1.957423	-0.972692
16	6	0	-3.074836	-0.494829	0.459498
17	7	0	-2.346946	-1.485064	1.322965
18	6	0	-1.444962	-2.432097	0.599177
19	1	0	0.311857	-2.830544	-1.460136
20	1	0	3.268469	-0.084194	0.002016
21	1	0	2.849685	0.597119	2.517419

22	1	0	1.160780	0.697663	3.015895
23	1	0	0.529472	3.704275	1.109405
24	1	0	-0.121550	2.628625	2.346766
25	1	0	-2.273235	-0.241795	-2.147267
26	1	0	0.875404	2.537902	-1.276495
27	1	0	-3.781066	0.017677	1.109236
28	1	0	-3.607618	-1.055913	-0.303389
29	1	0	-3.035765	-2.018168	1.856372
30	1	0	-1.908001	-2.688223	-0.351422
31	1	0	-1.374411	-3.330596	1.210953
32	8	0	2.748417	-1.783519	-1.878283
33	8	0	-0.361706	1.156118	-3.209350
34	1	0	2.296525	2.173423	0.761035
35	1	0	-1.760731	-0.961740	1.989888
36	1	0	2.346921	-2.428806	-2.476971
37	1	0	0.394170	1.734430	-3.384532

Most stable energy, Gibbs free energy (Ha), and geometry for protomer ./OH_Py2N2//3_11
E: -913.053018
G: -912.786840
Geometry:
Input orientation:

Center Number	Atomic Number	Atomic Type	Coordinates (Angstroms) X	Y	Z
1	6	0	-3.946470	-0.079249	0.466915
2	6	0	-3.389616	1.143152	0.104924
3	6	0	-2.061926	1.186406	-0.264145
4	7	0	-1.350860	0.056463	-0.262974
5	6	0	-1.849277	-1.141571	0.099195
6	6	0	-3.163939	-1.240988	0.463783
7	6	0	-0.866906	-2.283691	0.115151
8	7	0	0.000575	-2.294316	-1.063206
9	6	0	1.420919	-2.419653	-0.774224
10	6	0	2.068702	-1.128062	-0.327690
11	7	0	1.326543	-0.025887	-0.297735
12	6	0	1.853134	1.132463	0.115281
13	6	0	3.178342	1.242879	0.478468
14	6	0	3.966508	0.091689	0.434807
15	6	0	3.405826	-1.113619	0.040401
16	6	0	0.894765	2.294271	0.212285
17	7	0	0.028679	2.441394	-0.962418
18	6	0	-1.394719	2.471190	-0.712024
19	1	0	-3.986347	2.046378	0.107454
20	1	0	-3.592411	-2.192243	0.746075
21	1	0	-1.425833	-3.213314	0.221552
22	1	0	-0.260826	-2.172252	1.019343
23	1	0	1.923261	-2.746019	-1.686293
24	1	0	1.633083	-3.179616	-0.011923
25	1	0	3.597740	2.189932	0.793750
26	1	0	4.003212	-2.015674	0.014731
27	1	0	0.282657	2.136078	1.107007
28	1	0	1.468865	3.205738	0.383119
29	1	0	0.257964	1.760940	-1.676908
30	1	0	-1.908997	2.781428	-1.623872
31	1	0	-1.615723	3.232902	0.036937
32	8	0	-5.228797	-0.205753	0.827976
33	8	0	5.276093	0.105186	0.767938

34	1	0	-0.271691	-3.061479	-1.662373
35	1	0	-0.300854	0.060399	-0.463834
36	1	0	-5.685221	0.647890	0.790834
37	1	0	5.550419	1.000010	1.013262

Most stable energy, Gibbs free energy (Ha), and geometry for protomer ./OH_Py2N2//3_15
E: -913.042675
G: -912.771630
Geometry:
Input orientation:

Center Number	Atomic Number	Atomic Type	Coordinates (Angstroms) X	Y	Z
1	6	0	-2.029629	-0.728159	-1.576723
2	6	0	-2.328027	-0.863604	-0.209535
3	6	0	-1.861149	0.077736	0.660539
4	7	0	-1.162313	1.126253	0.178785
5	6	0	-0.788143	1.247051	-1.097768
6	6	0	-1.232542	0.323822	-2.016444
7	6	0	0.116234	2.410728	-1.419122
8	7	0	0.814714	2.883176	-0.232355
9	6	0	2.120250	2.232728	-0.018400
10	6	0	1.939353	0.751236	0.194100
11	7	0	1.300149	0.382431	1.320316
12	6	0	0.927229	-0.899329	1.397780
13	6	0	1.223016	-1.863925	0.464377
14	6	0	1.983909	-1.523525	-0.691731
15	6	0	2.308630	-0.135710	-0.789578
16	6	0	0.080792	-1.247883	2.599206
17	7	0	-0.811814	-0.094171	2.928392
18	6	0	-2.083208	0.001307	2.141817
19	1	0	-2.889725	-1.714999	0.147103
20	1	0	-0.940882	0.406004	-3.055280
21	1	0	-0.511321	3.214792	-1.808077
22	1	0	0.785750	2.098439	-2.227371
23	1	0	2.572280	2.686030	0.863856
24	1	0	2.786001	2.392216	-0.872087
25	1	0	0.856477	-2.876320	0.586142
26	1	0	2.811782	0.221673	-1.680385
27	1	0	-0.541474	-2.121942	2.421634
28	1	0	0.698653	-1.417622	3.480014
29	1	0	-0.240588	0.753469	2.800454
30	1	0	-2.602286	0.891856	2.494314
31	1	0	-2.672843	-0.880493	2.375677
32	8	0	-2.501851	-1.665367	-2.395013
33	8	0	2.300799	-2.371441	-1.588393
34	1	0	0.961211	3.880956	-0.316158
35	1	0	-0.739261	1.815035	0.806275
36	1	0	-1.075868	-0.138762	3.915369
37	1	0	-2.226259	-1.504705	-3.310947

Most stable energy, Gibbs free energy (Ha), and geometry for protomer ./OH_Py2N2//3_9
E: -913.056784
G: -912.786540
Geometry:
Input orientation:

Center Number	Atomic Number	Atomic Type	Coordinates (Angstroms) X	Y	Z

1	6	0	-2.429973	-0.969855	-0.329235
2	6	0	-1.396890	-1.537197	-1.061498
3	6	0	-0.459254	-0.678682	-1.613250
4	7	0	-0.501204	0.640599	-1.486147
5	6	0	-1.457385	1.175432	-0.714655
6	6	0	-2.450802	0.409799	-0.136315
7	6	0	-1.377684	2.657494	-0.446651
8	7	0	-0.094276	3.033692	0.156582
9	6	0	0.110088	2.468772	1.487078
10	6	0	0.707973	1.085978	1.413642
11	7	0	1.600328	0.887406	0.416131
12	6	0	2.063201	-0.350519	0.107220
13	6	0	1.791475	-1.404102	0.913962
14	6	0	0.956188	-1.244037	2.082836
15	6	0	0.389296	0.065705	2.253879
16	6	0	2.791556	-0.490141	-1.192512
17	7	0	1.861608	-0.307852	-2.359504
18	6	0	0.694726	-1.237303	-2.412030
19	1	0	-1.317429	-2.610726	-1.180830
20	1	0	-3.212523	0.859979	0.485988
21	1	0	-2.222092	2.938008	0.191520
22	1	0	-1.472483	3.196778	-1.390410
23	1	0	-0.811484	2.408760	2.076004
24	1	0	0.806694	3.112103	2.027630
25	1	0	2.160071	-2.388377	0.657931
26	1	0	-0.332042	0.228158	3.044010
27	1	0	3.571055	0.262629	-1.298862
28	1	0	3.221564	-1.483558	-1.278584
29	1	0	2.415233	-0.413473	-3.212470
30	1	0	1.009604	-2.208469	-2.035906
31	1	0	0.417146	-1.336773	-3.460648
32	8	0	-3.405397	-1.702525	0.249365
33	8	0	0.700046	-2.199550	2.850698
34	1	0	-0.066804	4.042938	0.228920
35	1	0	1.699429	1.650698	-0.247468
36	1	0	1.479000	0.648062	-2.355421
37	1	0	-3.273810	-2.643877	0.068566

Most stable energy, Gibbs free energy (Ha), and geometry for protomer ./OH_Py2N2//3_6
E: -913.042665
G: -912.771362
Geometry:

Input orientation:

Center Number	Atomic Number	Atomic Type	Coordinates (Angstroms) X	Y	Z
1	6	0	-1.990366	1.490918	-0.755453
2	6	0	-2.311563	0.099430	-0.798372
3	6	0	-1.939062	-0.747157	0.218950
4	7	0	-1.299875	-0.332142	1.329101
5	6	0	-0.929560	0.952307	1.355659
6	6	0	-1.228418	1.878520	0.384978
7	6	0	-0.082238	1.349439	2.541744
8	7	0	0.803372	0.206279	2.922666
9	6	0	2.078552	0.075195	2.147379
10	6	0	1.861659	-0.053590	0.669535
11	7	0	1.163025	-1.117650	0.223825

12	6	0	0.791096	-1.283303	-1.048284
13	6	0	1.236181	-0.392258	-1.998060
14	6	0	2.033664	0.673590	-1.593983
15	6	0	2.330793	0.856237	-0.232003
16	6	0	-0.110745	-2.459588	-1.328879
17	7	0	-0.807861	-2.890918	-0.125652
18	6	0	-2.115293	-2.236566	0.064981
19	1	0	-2.814339	-0.294314	-1.673926
20	1	0	-0.863867	2.895691	0.466080
21	1	0	-0.700090	1.562805	3.412995
22	1	0	0.545402	2.210959	2.325557
23	1	0	2.591225	-0.804339	2.534841
24	1	0	2.671780	0.961952	2.351481
25	1	0	0.946101	-0.509590	-3.033971
26	1	0	2.892759	1.719090	0.095512
27	1	0	-0.780909	-2.178168	-2.147855
28	1	0	0.519051	-3.275312	-1.688836
29	1	0	-0.951836	-3.891438	-0.172980
30	1	0	-2.564444	-2.656672	0.964950
31	1	0	-2.781699	-2.431528	-0.780776
32	8	0	-2.310894	2.302104	-1.684243
33	8	0	2.508288	1.581627	-2.443089
34	1	0	0.229083	-0.643281	2.823048
35	1	0	0.737200	-1.784070	0.873470
36	1	0	1.061377	0.287964	3.908843
37	1	0	2.235748	1.389159	-3.353635

Most stable energy, Gibbs free energy (Ha), and geometry for protomer ./OH_Py2N2//3_18
E: -913.062607
G: -912.792982
Geometry:

Input orientation:

Center Number	Atomic Number	Atomic Type	Coordinates (Angstroms) X	Y	Z
1	6	0	-2.722137	-0.180385	-0.346723
2	6	0	-1.981440	-1.286240	-0.767893
3	6	0	-0.744795	-1.083720	-1.324142
4	7	0	-0.293634	0.174493	-1.466235
5	6	0	-0.949657	1.262158	-1.020483
6	6	0	-2.189579	1.106062	-0.459454
7	6	0	-0.277826	2.600919	-1.182197
8	7	0	1.174930	2.515599	-1.126506
9	6	0	1.709773	2.541447	0.239325
10	6	0	1.470995	1.212235	0.895724
11	7	0	2.033808	0.146050	0.273204
12	6	0	1.690707	-1.132258	0.572123
13	6	0	0.880502	-1.383950	1.630897
14	6	0	0.337594	-0.309547	2.425428
15	6	0	0.654796	1.019015	1.962318
16	6	0	2.144468	-2.171210	-0.411946
17	7	0	1.554054	-1.890494	-1.725666
18	6	0	0.135541	-2.207994	-1.804291
19	1	0	-2.360859	-2.291002	-0.637032
20	1	0	-2.733611	1.961795	-0.085531
21	1	0	-0.695954	3.272803	-0.425345
22	1	0	-0.562783	2.991543	-2.161108
23	1	0	1.252262	3.322152	0.853291

24	1	0	2.782051	2.730966	0.177290
25	1	0	0.593060	-2.402711	1.854893
26	1	0	0.191424	1.870355	2.443174
27	1	0	3.229370	-2.130176	-0.515281
28	1	0	1.866562	-3.157843	-0.031149
29	1	0	2.046833	-2.432084	-2.424063
30	1	0	-0.134992	-3.103563	-1.234997
31	1	0	-0.119472	-2.394782	-2.849362
32	8	0	-3.930725	-0.303306	0.202559
33	8	0	-0.409002	-0.516725	3.409195
34	1	0	1.564540	3.294785	-1.641277
35	1	0	0.642989	0.304834	-1.848987
36	1	0	2.586675	0.314491	-0.560710
37	1	0	-4.197062	-1.234151	0.248941

Most stable energy, Gibbs free energy (Ha), and geometry for protomer ./OH_Py2N2//3_10
E: -913.043820
G: -912.771428
Geometry:
Input orientation:

Center Number	Atomic Number	Atomic Type	Coordinates (Angstroms) X	Y	Z
1	6	0	2.148237	1.656142	0.411435
2	6	0	0.879003	2.254989	0.164302
3	6	0	0.027666	1.707990	-0.766273
4	7	0	0.290811	0.613195	-1.486383
5	6	0	1.449622	-0.008726	-1.221644
6	6	0	2.389400	0.463344	-0.336738
7	6	0	1.679375	-1.317114	-1.934813
8	7	0	1.635570	-2.491463	-0.992343
9	6	0	0.280686	-2.980163	-0.581999
10	6	0	-0.450235	-1.967685	0.251077
11	7	0	-1.490103	-1.329108	-0.336517
12	6	0	-2.092441	-0.266879	0.257768
13	6	0	-1.772581	0.083955	1.526071
14	6	0	-0.746659	-0.620094	2.258699
15	6	0	-0.079374	-1.661418	1.519569
16	6	0	-3.027965	0.531447	-0.594645
17	7	0	-2.268262	1.349481	-1.597990
18	6	0	-1.315691	2.350303	-1.030076
19	1	0	0.585366	3.134148	0.725740
20	1	0	3.315272	-0.079634	-0.180229
21	1	0	2.667841	-1.346633	-2.388014
22	1	0	0.922596	-1.491943	-2.695297
23	1	0	0.444299	-3.890375	-0.010146
24	1	0	-0.260840	-3.220069	-1.494252
25	1	0	-2.256443	0.935693	1.985088
26	1	0	0.748168	-2.188486	1.977608
27	1	0	-3.701994	-0.111427	-1.158394
28	1	0	-3.600465	1.222989	0.016429
29	1	0	-2.952338	1.826861	-2.188164
30	1	0	-1.750991	2.762523	-0.122251
31	1	0	-1.222358	3.145328	-1.768728
32	8	0	2.989237	2.129341	1.243442
33	8	0	-0.436409	-0.320522	3.429902
34	1	0	2.183876	-2.269284	-0.153880
35	1	0	-1.699520	-1.536496	-1.307493

36	1	0	-1.708922	0.726475	-2.199490
37	1	0	2.108563	-3.278123	-1.446273

Most stable energy, Gibbs free energy (Ha), and geometry for protomer ./OH_Py2N2//3_8
E: -913.042571
G: -912.771630
Geometry:
Input orientation:

Center Number	Atomic Number	Atomic Type	Coordinates (Angstroms) X	Y	Z
1	6	0	-1.983448	-1.573077	0.596880
2	6	0	-1.213134	-1.842478	-0.571470
3	6	0	-0.912297	-0.822960	-1.443081
4	7	0	-1.286765	0.451342	-1.290436
5	6	0	-1.934597	0.751162	-0.149047
6	6	0	-2.310461	-0.193920	0.776391
7	6	0	-2.125285	2.217334	0.145903
8	7	0	-0.824538	2.867984	0.383912
9	6	0	-0.117114	2.344015	1.543229
10	6	0	0.788422	1.198847	1.164107
11	7	0	1.171840	1.147682	-0.114804
12	6	0	1.868271	0.123267	-0.649288
13	6	0	2.322888	-0.868046	0.170169
14	6	0	2.011964	-0.809346	1.539701
15	6	0	1.220782	0.223220	2.032977
16	6	0	2.097366	0.122716	-2.131587
17	7	0	0.829096	0.068498	-2.927336
18	6	0	-0.064477	-1.100795	-2.661693
19	1	0	-0.845448	-2.845675	-0.751892
20	1	0	-2.822236	0.107887	1.682695
21	1	0	-2.785473	2.323958	1.012088
22	1	0	-2.589419	2.713155	-0.706812
23	1	0	-0.781227	1.992888	2.339998
24	1	0	0.510007	3.131739	1.964999
25	1	0	2.880608	-1.702529	-0.229946
26	1	0	0.919749	0.247298	3.072125
27	1	0	2.619191	1.029044	-2.436623
28	1	0	2.686237	-0.747678	-2.406653
29	1	0	1.097262	0.074269	-3.914232
30	1	0	0.557564	-1.983757	-2.535247
31	1	0	-0.682710	-1.220201	-3.550440
32	8	0	-2.307993	-2.474653	1.436945
33	8	0	2.463695	-1.798317	2.306804
34	1	0	-0.979336	3.859040	0.517836
35	1	0	0.754321	1.869017	-0.708162
36	1	0	0.259212	0.909820	-2.759174
37	1	0	2.167207	-1.692584	3.224018

Most stable energy, Gibbs free energy (Ha), and geometry for protomer ./OH_Py2N2//3_3
E: -913.050726
G: -912.780231
Geometry:
Input orientation:

Center Number	Atomic Number	Atomic Type	Coordinates (Angstroms) X	Y	Z
1	6	0	1.789214	1.742241	0.677087

2	6	0	1.059036	1.053478	1.691176
3	6	0	0.911026	-0.311914	1.631263
4	7	0	1.422023	-1.088001	0.670195
5	6	0	2.025455	-0.449987	-0.343356
6	6	0	2.246565	0.903879	-0.386814
7	6	0	2.330354	-1.318036	-1.529925
8	7	0	1.036372	-1.836932	-2.090983
9	6	0	0.124547	-0.800526	-2.662083
10	6	0	-0.798472	-0.252669	-1.600997
11	7	0	-1.156204	-1.095294	-0.641485
12	6	0	-1.894089	-0.623372	0.366305
13	6	0	-2.392545	0.661048	0.404049
14	6	0	-2.070721	1.515038	-0.650071
15	6	0	-1.232043	1.062399	-1.659553
16	6	0	-2.096497	-1.576936	1.509832
17	7	0	-0.772053	-2.069252	2.003571
18	6	0	0.093542	-1.042356	2.668252
19	1	0	0.594125	1.619667	2.489428
20	1	0	2.725028	1.353115	-1.248746
21	1	0	2.912272	-2.194019	-1.249620
22	1	0	2.833191	-0.773813	-2.324569
23	1	0	-0.456430	-1.283625	-3.446654
24	1	0	0.730804	-0.013963	-3.105459
25	1	0	-2.982595	1.013762	1.238977
26	1	0	-0.910214	1.723997	-2.454232
27	1	0	-2.606341	-1.106420	2.346046
28	1	0	-2.652842	-2.455698	1.187058
29	1	0	-0.233973	-2.432740	1.202753
30	1	0	0.741315	-1.582576	3.357128
31	1	0	-0.547509	-0.368374	3.231492
32	8	0	1.967399	3.002539	0.686613
33	8	0	-2.554790	2.772970	-0.614525
34	1	0	0.524644	-2.308229	-1.331212
35	1	0	-0.937264	-2.843292	2.648872
36	1	0	1.246383	-2.533022	-2.808315
37	1	0	-2.241140	3.276088	-1.379687

Most stable energy, Gibbs free energy (Ha), and geometry for protomer ./OH_Py2N2//3_4
E: -913.050730
G: -912.780262
Geometry:
Input orientation:

Center Number	Atomic Number	Atomic Type	Coordinates (Angstroms) X	Y	Z
1	6	0	2.073446	-1.515755	-0.642092
2	6	0	1.233990	-1.070355	-1.654115
3	6	0	0.798432	0.244379	-1.603295
4	7	0	1.155058	1.093239	-0.648876
5	6	0	1.893695	0.628382	0.361623
6	6	0	2.393985	-0.655072	0.406960
7	6	0	2.094857	1.588977	1.499455
8	7	0	0.769812	2.082791	1.990053
9	6	0	-0.094364	1.059131	2.661490
10	6	0	-0.911045	0.320736	1.629530
11	7	0	-1.422591	1.089543	0.662936
12	6	0	-2.025873	0.443929	-0.345855
13	6	0	-2.246454	-0.910313	-0.379417

14	6	0	-1.789178	-1.740585	0.690871
15	6	0	-1.058628	-1.044211	1.699486
16	6	0	-2.332281	1.303643	-1.538096
17	7	0	-1.039502	1.822225	-2.102089
18	6	0	-0.125484	0.784652	-2.667489
19	1	0	0.913266	-1.737197	-2.444839
20	1	0	2.984595	-1.001980	1.243918
21	1	0	2.650251	2.466392	1.171424
22	1	0	2.605166	1.124064	2.338522
23	1	0	-0.742894	1.603013	3.346780
24	1	0	0.547633	0.389859	3.229248
25	1	0	-2.725128	-1.366001	-1.237828
26	1	0	-0.593674	-1.604369	2.501955
27	1	0	-2.833636	0.753193	-2.329387
28	1	0	-2.916481	2.179920	-1.263501
29	1	0	-0.528708	2.298946	-1.345055
30	1	0	0.454690	1.264763	-3.454483
31	1	0	-0.730111	-0.005441	-3.106775
32	8	0	2.559534	-2.772674	-0.599248
33	8	0	-1.968070	-3.000660	0.710642
34	1	0	0.231240	2.440408	1.186935
35	1	0	-1.251168	2.513741	-2.823342
36	1	0	0.934132	2.861243	2.630251
37	1	0	2.246804	-3.280677	-1.361561

Most stable energy, Gibbs free energy (Ha), and geometry for protomer ./OH_Py2N2//3_20
E: -913.055253
G: -912.783295
Geometry:

Input orientation:

Center Number	Atomic Number	Atomic Type	Coordinates (Angstroms) X	Y	Z
1	6	0	0.344840	-2.180513	-1.623199
2	6	0	1.349370	-1.979274	-0.606516
3	6	0	1.007983	-1.557327	0.635662
4	7	0	-0.294859	-1.361405	0.956018
5	6	0	-1.286402	-1.465707	0.041259
6	6	0	-1.000445	-1.871726	-1.224619
7	6	0	-2.672915	-1.116589	0.524133
8	7	0	-2.657561	-0.091042	1.562966
9	6	0	-2.660289	1.276943	1.036475
10	6	0	-1.336669	1.588256	0.399532
11	7	0	-0.251773	1.534930	1.217132
12	6	0	1.012124	1.565784	0.726733
13	6	0	1.233183	1.784864	-0.592990
14	6	0	0.135584	1.943470	-1.514091
15	6	0	-1.174012	1.808369	-0.929142
16	6	0	2.128990	1.298369	1.694892
17	7	0	2.805396	-0.010986	1.414005
18	6	0	2.021327	-1.259427	1.703794
19	1	0	2.391225	-2.142679	-0.851923
20	1	0	-1.795829	-1.939601	-1.955220
21	1	0	-3.116872	-2.019586	0.946605
22	1	0	-3.270090	-0.830968	-0.348033
23	1	0	-2.834589	1.959292	1.869451
24	1	0	-3.442470	1.434600	0.288492
25	1	0	2.248013	1.810955	-0.970183

26	1	0	-2.042100	1.831228	-1.574557
27	1	0	2.898957	2.061205	1.604595
28	1	0	1.776020	1.260861	2.723164
29	1	0	3.650474	-0.040976	1.993032
30	1	0	1.556535	-1.125426	2.678970
31	1	0	2.749169	-2.065241	1.758406
32	8	0	0.637934	-2.570820	-2.777569
33	8	0	0.311273	2.141869	-2.735867
34	1	0	-3.474970	-0.207473	2.148053
35	1	0	-0.548020	-1.015779	1.876674
36	1	0	-0.396791	1.321516	2.198646
37	1	0	3.124189	-0.028471	0.438157

Most stable energy, Gibbs free energy (Ha), and geometry for protomer ./OH_Py2N2//3_12

E: -913.056780

G: -912.786495

Geometry:

Input orientation:

Center Number	Atomic Number	Atomic Type	Coordinates (Angstroms) X	Y	Z
1	6	0	0.975363	-1.241491	2.074698
2	6	0	0.404673	0.065991	2.250199
3	6	0	0.713786	1.087869	1.408349
4	7	0	1.600281	0.893244	0.404874
5	6	0	2.065801	-0.342769	0.092223
6	6	0	1.803512	-1.397750	0.900239
7	6	0	2.785278	-0.479081	-1.212847
8	7	0	1.845584	-0.301504	-2.372679
9	6	0	0.682351	-1.236120	-2.415138
10	6	0	-0.468307	-0.680843	-1.609177
11	7	0	-0.513891	0.638370	-1.482571
12	6	0	-1.466599	1.170505	-0.704920
13	6	0	-2.453638	0.401938	-0.119668
14	6	0	-2.429608	-0.977736	-0.312078
15	6	0	-1.399434	-1.542157	-1.050734
16	6	0	-1.389883	2.652887	-0.437926
17	7	0	-0.104029	3.033188	0.157518
18	6	0	0.110707	2.468168	1.486335
19	1	0	-0.312158	0.225312	3.045061
20	1	0	2.174007	-2.380509	0.641161
21	1	0	3.219504	-1.470401	-1.302002
22	1	0	3.560270	0.277471	-1.324905
23	1	0	1.004041	-2.205200	-2.039407
24	1	0	0.397739	-1.338902	-3.461542
25	1	0	-3.212709	0.849931	0.507431
26	1	0	-1.317280	-2.615523	-1.169842
27	1	0	-1.491980	3.191604	-1.381250
28	1	0	-2.231287	2.930944	0.205257
29	1	0	-0.079702	4.042482	0.230316
30	1	0	-0.807028	2.403863	2.080795
31	1	0	0.807891	3.114007	2.023139
32	8	0	0.727727	-2.198432	2.843590
33	8	0	-3.398990	-1.713061	0.273225
34	1	0	2.392737	-0.405181	-3.230049
35	1	0	1.691763	1.657114	-0.259205
36	1	0	1.458808	0.652741	-2.365921
37	1	0	-3.266089	-2.654066	0.091726

Most stable energy, Gibbs free energy (Ha), and geometry for protomer ./OH_Py2N2//3_17
E: -913.062637
G: -912.792706
Geometry:
Input orientation:

Center Number	Atomic Number	Atomic Type	Coordinates (Angstroms) X	Y	Z
1	6	0	0.325365	0.365655	2.417410
2	6	0	0.893395	1.411880	1.602927
3	6	0	1.706747	1.121632	0.556490
4	7	0	2.029993	-0.169095	0.290310
5	6	0	1.444261	-1.210641	0.933062
6	6	0	0.623351	-0.978540	1.988157
7	6	0	1.666124	-2.558953	0.310437
8	7	0	1.148543	-2.555613	-1.062098
9	6	0	-0.304341	-2.622911	-1.134640
10	6	0	-0.959603	-1.272869	-1.001950
11	7	0	-0.286495	-0.202318	-1.463751
12	6	0	-0.721857	1.064041	-1.348184
13	6	0	-1.959011	1.293736	-0.803052
14	6	0	-2.716244	0.205659	-0.364808
15	6	0	-2.200176	-1.089702	-0.451238
16	6	0	0.176483	2.166854	-1.844905
17	7	0	1.589733	1.829134	-1.757809
18	6	0	2.183438	2.128765	-0.449736
19	1	0	0.621457	2.440279	1.800691
20	1	0	0.141625	-1.810371	2.484802
21	1	0	1.187396	-3.316764	0.936782
22	1	0	2.735484	-2.768967	0.266074
23	1	0	-0.740776	-3.277298	-0.372718
24	1	0	-0.582197	-3.024931	-2.110993
25	1	0	-2.325696	2.305654	-0.692797
26	1	0	-2.757874	-1.930706	-0.064292
27	1	0	-0.073845	2.340057	-2.893437
28	1	0	-0.081477	3.075847	-1.291247
29	1	0	2.091934	2.346881	-2.467503
30	1	0	1.924369	3.129279	-0.092551
31	1	0	3.267482	2.063759	-0.549679
32	8	0	-0.426430	0.608371	3.389018
33	8	0	-3.925567	0.352030	0.177033
34	1	0	1.534116	-3.350334	-1.555748
35	1	0	2.585418	-0.366770	-0.535391
36	1	0	0.651078	-0.351468	-1.837476
37	1	0	-4.183627	1.285797	0.206631

Most stable energy, Gibbs free energy (Ha), and geometry for protomer ./OH_Py2N2//3_13
E: -913.042570
G: -912.771556
Geometry:
Input orientation:

Center Number	Atomic Number	Atomic Type	Coordinates (Angstroms) X	Y	Z
1	6	0	-2.018685	0.793757	-1.541839
2	6	0	-1.224614	-0.238949	-2.030054
3	6	0	-0.785977	-1.206860	-1.155724

4	7	0	-1.165864	-1.148332	0.123891
5	6	0	-1.864924	-0.123028	0.653238
6	6	0	-2.325635	0.860941	-0.171754
7	6	0	-2.091315	-0.114203	2.135975
8	7	0	-0.822323	-0.053510	2.929965
9	6	0	0.069050	1.115965	2.657704
10	6	0	0.914362	0.834164	1.438338
11	7	0	1.293405	-0.439607	1.292533
12	6	0	1.937912	-0.744124	0.150471
13	6	0	2.305992	0.196408	-0.782696
14	6	0	1.974005	1.575360	-0.610866
15	6	0	1.207505	1.849232	0.558964
16	6	0	2.132405	-2.211347	-0.136564
17	7	0	0.832979	-2.866762	-0.368682
18	6	0	0.122306	-2.351400	-1.529790
19	1	0	-0.926603	-0.269508	-3.069916
20	1	0	-2.885700	1.695927	0.224034
21	1	0	-2.611327	-1.019670	2.446680
22	1	0	-2.681418	0.756575	2.407127
23	1	0	0.689162	1.239715	3.544569
24	1	0	-0.554532	1.997491	2.529047
25	1	0	2.815145	-0.109175	-1.689215
26	1	0	0.836133	2.851972	0.734276
27	1	0	2.791291	-2.320852	-1.003396
28	1	0	2.599386	-2.701241	0.717986
29	1	0	0.990105	-3.858171	-0.497059
30	1	0	-0.503264	-3.143193	-1.946215
31	1	0	0.784169	-2.002779	-2.329529
32	8	0	-2.477421	1.775058	-2.314737
33	8	0	2.291250	2.472673	-1.458196
34	1	0	-0.251114	-0.894747	2.766026
35	1	0	-0.744580	-1.864537	0.720797
36	1	0	-1.089756	-0.054465	3.917092
37	1	0	-2.184338	1.663814	-3.232455

Most stable energy, Gibbs free energy (Ha), and geometry for protomer ./OH_Py2N2//4_2
E: -913.504742
G: -913.221177
Geometry:
Input orientation:

Center Number	Atomic Number	Atomic Type	Coordinates (Angstroms) X	Y	Z
1	6	0	-2.235470	-0.732973	1.170594
2	6	0	-2.340131	0.560989	0.662457
3	6	0	-1.785670	0.835640	-0.572280
4	7	0	-1.183616	-0.105585	-1.312164
5	6	0	-1.037652	-1.316586	-0.790462
6	6	0	-1.541651	-1.690909	0.443881
7	6	0	-0.254985	-2.310431	-1.614812
8	7	0	0.734208	-1.598580	-2.478304
9	6	0	2.055010	-1.278889	-1.842566
10	6	0	1.898666	-0.475054	-0.587985
11	7	0	1.402354	0.774195	-0.699892
12	6	0	1.045563	1.525456	0.347457
13	6	0	1.313306	1.073480	1.618218
14	6	0	1.913409	-0.173288	1.775653
15	6	0	2.185199	-0.970367	0.650106

16	6	0	0.336291	2.817602	0.030847
17	7	0	-0.372241	2.727787	-1.238219
18	6	0	-1.754837	2.245710	-1.104251
19	1	0	-2.819796	1.336694	1.244141
20	1	0	-1.386992	-2.688850	0.834851
21	1	0	-0.917966	-2.857963	-2.283465
22	1	0	0.281026	-3.018605	-0.986678
23	1	0	2.635624	-0.733433	-2.584979
24	1	0	2.541192	-2.222651	-1.614113
25	1	0	1.030839	1.666399	2.478360
26	1	0	2.579226	-1.969741	0.766951
27	1	0	-0.313013	3.059574	0.878714
28	1	0	1.092298	3.602186	-0.037270
29	1	0	-0.393243	3.644146	-1.667224
30	1	0	-2.212115	2.270621	-2.093705
31	1	0	-2.340284	2.884715	-0.436382
32	8	0	-2.780802	-0.982911	2.378102
33	8	0	2.205683	-0.685148	2.966124
34	1	0	0.278736	-0.735151	-2.805816
35	1	0	1.104625	1.137597	-1.608542
36	1	0	0.930674	-2.168817	-3.304642
37	1	0	-2.643840	-1.907762	2.628845
38	1	0	1.972166	-0.071288	3.679764

Most stable energy, Gibbs free energy (Ha), and geometry for protomer ./OH_Py2N2//4_7
E: -913.504732
G: -913.221241
Geometry:

Input orientation:

Center Number	Atomic Number	Atomic Type	Coordinates (Angstroms) X	Y	Z
1	6	0	-1.911825	0.180019	-1.776131
2	6	0	-2.186533	0.969891	-0.646211
3	6	0	-1.900604	0.467899	0.589344
4	7	0	-1.402268	-0.781120	0.694457
5	6	0	-1.042801	-1.525567	-0.356825
6	6	0	-1.309810	-1.066645	-1.625255
7	6	0	-0.331300	-2.818128	-0.046827
8	7	0	0.375309	-2.734172	1.223709
9	6	0	1.757183	-2.248749	1.094502
10	6	0	1.786229	-0.835792	0.570195
11	7	0	1.181235	0.100380	1.314071
12	6	0	1.033941	1.313884	0.798577
13	6	0	1.539327	1.695700	-0.432923
14	6	0	2.236145	0.742899	-1.163508
15	6	0	2.342309	-0.553549	-0.662059
16	6	0	0.248380	2.302211	1.626830
17	7	0	-0.739952	1.584543	2.486428
18	6	0	-2.059533	1.264475	1.848294
19	1	0	-2.582190	1.969230	-0.757709
20	1	0	-1.025218	-1.654045	-2.488485
21	1	0	0.319719	-3.053823	-0.895154
22	1	0	-1.085726	-3.604700	0.015675
23	1	0	2.344888	-2.882956	0.424050
24	1	0	2.212984	-2.278162	2.084514
25	1	0	1.383645	2.695453	-0.818831
26	1	0	2.824409	-1.325279	-1.246993

27	1	0	0.909650	2.848237	2.298402
28	1	0	-0.288637	3.012082	1.001449
29	1	0	-0.938562	2.151202	3.314716
30	1	0	-2.548079	2.208170	1.624609
31	1	0	-2.639007	0.713598	2.587570
32	8	0	-2.203381	0.698579	-2.963900
33	8	0	2.783152	1.000134	-2.368702
34	1	0	0.397467	-3.652879	1.647586
35	1	0	-1.104930	-1.149385	1.601276
36	1	0	-0.282999	0.720935	2.811230
37	1	0	-1.967465	0.089799	-3.681117
38	1	0	2.644767	1.925941	-2.615027

Most stable energy, Gibbs free energy (Ha), and geometry for protomer ./OH_Py2N2//4_1

E: -913.511789

G: -913.228938

Geometry:

Input orientation:

Center Number	Atomic Number	Atomic Type	Coordinates (Angstroms) X	Y	Z
1	6	0	-2.009851	0.370721	-1.606991
2	6	0	-1.410364	1.497945	-1.058755
3	6	0	-1.100900	1.476198	0.290761
4	7	0	-1.361492	0.444125	1.083555
5	6	0	-1.864313	-0.660108	0.527698
6	6	0	-2.220124	-0.747477	-0.801411
7	6	0	-1.944120	-1.853271	1.436752
8	7	0	-0.588341	-2.149406	2.003673
9	6	0	0.429528	-2.633583	1.020152
10	6	0	1.108380	-1.471361	0.340581
11	7	0	1.373026	-0.421617	1.108735
12	6	0	1.870270	0.670388	0.524688
13	6	0	2.217716	0.728516	-0.808471
14	6	0	2.003677	-0.407458	-1.587577
15	6	0	1.408605	-1.522649	-1.009991
16	6	0	1.946689	1.885250	1.404275
17	7	0	0.585212	2.199857	1.947812
18	6	0	-0.420512	2.655562	0.938904
19	1	0	-1.172621	2.356479	-1.674446
20	1	0	-2.617967	-1.661850	-1.220035
21	1	0	-2.283577	-2.743152	0.913846
22	1	0	-2.594923	-1.652705	2.286106
23	1	0	-0.070553	-3.281387	0.304502
24	1	0	1.158236	-3.216563	1.581980
25	1	0	2.611256	1.634095	-1.249750
26	1	0	1.166860	-2.394483	-1.605052
27	1	0	2.587003	1.703439	2.265600
28	1	0	2.294494	2.760936	0.863355
29	1	0	0.686400	2.920152	2.665466
30	1	0	0.089484	3.276687	0.206813
31	1	0	-1.151042	3.261505	1.473468
32	8	0	-2.354875	0.287213	-2.906067
33	8	0	2.339872	-0.352739	-2.890372
34	1	0	-0.696265	-2.848544	2.741046
35	1	0	0.208631	1.357076	2.403974
36	1	0	-0.218023	-1.292879	2.438981
37	1	0	-2.152697	1.115346	-3.364985

38 1 0 2.136706 -1.191244 -3.329668

Most stable energy, Gibbs free energy (Ha), and geometry for protomer ./OH_Py2N2//4_10

E: -913.510325

G: -913.228540

Geometry:

Input orientation:

Center Number	Atomic Number	Atomic Type	Coordinates (Angstroms) X	Y	Z
1	6	0	-2.239484	-1.534613	-0.012343
2	6	0	-2.039948	-0.841692	-1.212531
3	6	0	-1.617106	0.458225	-1.162707
4	7	0	-1.431763	1.035964	0.039650
5	6	0	-1.533152	0.385211	1.210369
6	6	0	-1.954683	-0.919039	1.209961
7	6	0	-1.143895	1.149262	2.447379
8	7	0	0.022009	1.994311	2.210187
9	6	0	1.297080	1.299772	2.370170
10	6	0	1.616342	0.463422	1.162191
11	7	0	1.430002	1.039516	-0.040804
12	6	0	1.532684	0.387664	-1.210813
13	6	0	1.956641	-0.915804	-1.208926
14	6	0	2.242427	-1.529549	0.014077
15	6	0	2.041594	-0.835647	1.213485
16	6	0	1.141782	1.149552	-2.448648
17	7	0	-0.026360	1.991828	-2.212495
18	6	0	-1.299639	1.293833	-2.371691
19	1	0	-2.188162	-1.335175	-2.162438
20	1	0	-2.036528	-1.466356	2.139536
21	1	0	-0.993503	0.428856	3.256437
22	1	0	-1.983337	1.790450	2.722343
23	1	0	1.319578	0.646914	3.247240
24	1	0	2.081983	2.049536	2.484113
25	1	0	2.039505	-1.464012	-2.137887
26	1	0	2.190635	-1.327822	2.163941
27	1	0	1.979606	1.792679	-2.723993
28	1	0	0.993461	0.428013	-3.257082
29	1	0	-0.002426	2.774857	-2.852348
30	1	0	-1.320400	0.639786	-3.247918
31	1	0	-2.086446	2.041426	-2.486741
32	8	0	-2.655626	-2.794496	-0.094134
33	8	0	2.660715	-2.788619	0.097310
34	1	0	-0.003940	2.778032	2.849116
35	1	0	-1.086658	1.994195	0.065205
36	1	0	1.082846	1.996980	-0.067316
37	1	0	-2.748730	-3.191612	0.785519
38	1	0	2.754460	-3.186640	-0.781870

Most stable energy, Gibbs free energy (Ha), and geometry for protomer ./OH_Py2N2//4_12

E: -913.503560

G: -913.218553

Geometry:

Input orientation:

Center Number	Atomic Number	Atomic Type	Coordinates (Angstroms) X	Y	Z
1	6	0	-1.079423	2.434173	0.001039

2	6	0	0.305647	2.401643	-0.147608
3	6	0	0.867761	1.409148	-0.914596
4	7	0	0.068799	0.509757	-1.503273
5	6	0	-1.267428	0.466930	-1.329995
6	6	0	-1.875023	1.440894	-0.591954
7	6	0	-2.009984	-0.679863	-1.954241
8	7	0	-2.525055	-1.648474	-0.931483
9	6	0	-1.536416	-2.621696	-0.356116
10	6	0	-0.479285	-1.927899	0.455253
11	7	0	0.772826	-1.890020	-0.067510
12	6	0	1.763840	-1.150357	0.499700
13	6	0	1.546020	-0.517870	1.679429
14	6	0	0.264674	-0.572584	2.338626
15	6	0	-0.752457	-1.313475	1.632721
16	6	0	3.005864	-0.958642	-0.321516
17	7	0	2.694080	-0.124244	-1.489808
18	6	0	2.348815	1.248644	-1.147291
19	1	0	0.935411	3.132607	0.342394
20	1	0	-2.946443	1.436749	-0.444268
21	1	0	-1.379537	-1.239801	-2.642481
22	1	0	-2.880335	-0.302821	-2.486027
23	1	0	-1.112022	-3.174202	-1.191736
24	1	0	-2.111425	-3.299042	0.270538
25	1	0	2.333908	0.083650	2.113134
26	1	0	-1.755702	-1.352684	2.038324
27	1	0	3.771831	-0.508795	0.315436
28	1	0	3.369441	-1.921434	-0.681181
29	1	0	3.494948	-0.121842	-2.108337
30	1	0	2.621211	1.900058	-1.979461
31	1	0	2.868010	1.617458	-0.256767
32	8	0	-1.707228	3.364846	0.711598
33	8	0	0.038988	0.011649	3.418292
34	1	0	-3.260211	-2.202252	-1.383532
35	1	0	0.540309	-0.214387	-2.047203
36	1	0	0.946500	-2.331447	-0.965039
37	1	0	-2.984574	-1.136715	-0.168817
38	1	0	-1.083408	4.005098	1.086727

Most stable energy, Gibbs free energy (Ha), and geometry for protomer ./OH_Py2N2//4_6
E: -913.504732
G: -913.221184
Geometry:
Input orientation:

Center Number	Atomic Number	Atomic Type	Coordinates (Angstroms) X	Y	Z
1	6	0	-1.907741	-0.192307	-1.779078
2	6	0	-1.310571	1.057023	-1.631022
3	6	0	-1.047395	1.520755	-0.363536
4	7	0	-1.406008	0.778226	0.689388
5	6	0	-1.899852	-0.472826	0.587349
6	6	0	-2.181814	-0.979584	-0.647185
7	6	0	-2.058129	-1.266313	1.848419
8	7	0	-0.738599	-1.579741	2.489925
9	6	0	0.253771	-2.296325	1.634113
10	6	0	1.037024	-1.307618	0.804159
11	7	0	1.179445	-0.092176	1.316475
12	6	0	1.782427	0.843898	0.570845

13	6	0	2.341423	0.560023	-0.659711
14	6	0	2.240096	-0.738104	-1.157822
15	6	0	1.545361	-1.691163	-0.425596
16	6	0	1.747430	2.258291	1.090881
17	7	0	0.363572	2.739196	1.215869
18	6	0	-0.340878	2.816718	-0.056264
19	1	0	-1.026491	1.642748	-2.495561
20	1	0	-2.573786	-1.980634	-0.756309
21	1	0	-2.542671	-2.212497	1.626482
22	1	0	-2.641080	-0.715551	2.585030
23	1	0	-0.279562	-3.010047	1.010018
24	1	0	0.915957	-2.837889	2.308383
25	1	0	2.821872	1.331703	-1.246066
26	1	0	1.393435	-2.692458	-0.808993
27	1	0	2.201130	2.292259	2.081707
28	1	0	2.334204	2.892562	0.419681
29	1	0	0.381650	3.659240	1.637022
30	1	0	0.310970	3.051937	-0.904083
31	1	0	-1.098106	3.600903	0.002312
32	8	0	-2.194989	-0.715817	-2.965747
33	8	0	2.789503	-0.996807	-2.361594
34	1	0	-0.936776	-2.144958	3.319308
35	1	0	-1.111621	1.150450	1.595528
36	1	0	-0.285225	-0.713766	2.813416
37	1	0	-1.959480	-0.108785	-3.684587
38	1	0	2.653924	-1.923574	-2.605842

Most stable energy, Gibbs free energy (Ha), and geometry for protomer ./OH_Py2N2//4_14
E: -913.503572
G: -913.218468
Geometry:
Input orientation:

Center Number	Atomic Number	Atomic Type	Coordinates (Angstroms) X	Y	Z
1	6	0	1.072448	-2.437452	-0.013789
2	6	0	1.869965	-1.443326	-0.602802
3	6	0	1.263992	-0.463333	-1.334092
4	7	0	-0.072697	-0.500980	-1.504938
5	6	0	-0.873313	-1.401293	-0.919962
6	6	0	-0.312794	-2.399781	-0.159598
7	6	0	-2.354272	-1.235417	-1.149535
8	7	0	-2.696372	0.140235	-1.484017
9	6	0	-3.004105	0.969219	-0.310794
10	6	0	-1.760174	1.153233	0.509306
11	7	0	-0.768481	1.893960	-0.055383
12	6	0	0.484983	1.924769	0.464582
13	6	0	0.759018	1.302479	1.637721
14	6	0	-0.258676	0.560342	2.341498
15	6	0	-1.541519	0.512913	1.684653
16	6	0	1.542270	2.619875	-0.345459
17	7	0	2.527563	1.646904	-0.927169
18	6	0	2.008899	0.684271	-1.953841
19	1	0	2.941690	-1.443361	-0.457307
20	1	0	-0.943816	-3.131679	0.327373
21	1	0	-2.873150	-1.607808	-0.260326
22	1	0	-2.629568	-1.881493	-1.984923
23	1	0	-3.770214	0.517955	0.324985

24	1	0	-3.365723	1.934870	-0.664716
25	1	0	1.763318	1.336156	2.041204
26	1	0	-2.329976	-0.089403	2.116217
27	1	0	1.117642	3.177060	-1.177862
28	1	0	2.120095	3.292972	0.283180
29	1	0	2.987169	1.130515	-0.167716
30	1	0	2.877838	0.307430	-2.488081
31	1	0	1.379142	1.248872	-2.638854
32	8	0	1.698882	-3.373895	0.690411
33	8	0	-0.032296	-0.030790	3.417256
34	1	0	-3.498153	0.143389	-2.101364
35	1	0	-0.543047	0.227775	-2.043711
36	1	0	-0.942883	2.341086	-0.949918
37	1	0	3.263284	2.201151	-1.377729
38	1	0	1.074061	-4.014175	1.063837

Most stable energy, Gibbs free energy (Ha), and geometry for protomer ./OH_Py2N2//4_3
E: -913.504705
G: -913.221608
Geometry:

Input orientation:

Center Number	Atomic Number	Atomic Type	Coordinates (Angstroms) X	Y	Z
1	6	0	-2.235346	0.767601	-1.152942
2	6	0	-1.530477	1.708577	-0.414810
3	6	0	-1.023558	1.310952	0.811118
4	7	0	-1.176749	0.093073	1.314469
5	6	0	-1.789715	-0.831669	0.562836
6	6	0	-2.347768	-0.533316	-0.664597
7	6	0	-1.768304	-2.250186	1.072229
8	7	0	-0.389289	-2.745679	1.193586
9	6	0	0.314829	-2.819555	-0.078955
10	6	0	1.034224	-1.527975	-0.374685
11	7	0	1.400319	-0.798776	0.684941
12	6	0	1.905775	0.448449	0.594373
13	6	0	2.192508	0.963857	-0.635476
14	6	0	1.910243	0.190042	-1.774616
15	6	0	1.301382	-1.054946	-1.637918
16	6	0	2.069867	1.229014	1.862703
17	7	0	0.752118	1.555181	2.501814
18	6	0	-0.229940	2.286678	1.646811
19	1	0	-1.370299	2.711352	-0.790924
20	1	0	-2.836400	-1.296031	-1.255851
21	1	0	-2.361281	-2.873440	0.396152
22	1	0	-2.222408	-2.287275	2.062764
23	1	0	-0.339142	-3.041008	-0.928875
24	1	0	1.064060	-3.611828	-0.026897
25	1	0	2.593925	1.962107	-0.735488
26	1	0	1.011394	-1.629948	-2.507679
27	1	0	2.642229	0.664448	2.597107
28	1	0	2.567872	2.170482	1.650622
29	1	0	0.954717	2.114778	3.333927
30	1	0	0.313155	2.996671	1.026659
31	1	0	-0.886936	2.833543	2.321875
32	8	0	-2.784780	1.040274	-2.353618
33	8	0	2.201722	0.722315	-2.956353
34	1	0	-0.416616	-3.668958	1.607073

35	1	0	1.101841	-1.175932	1.587748
36	1	0	0.288660	0.692983	2.820780
37	1	0	-2.641450	1.967487	-2.591544
38	1	0	1.958183	0.125627	-3.681171

Most stable energy, Gibbs free energy (Ha), and geometry for protomer ./OH_Py2N2//4_5
E: -913.505829
G: -913.219790
Geometry:
Input orientation:

Center Number	Atomic Number	Atomic Type	Coordinates (Angstroms) X	Y	Z
1	6	0	1.904719	-1.833925	0.022182
2	6	0	2.251088	-0.633623	-0.593745
3	6	0	1.289493	0.017513	-1.341008
4	7	0	0.062213	-0.471754	-1.535137
5	6	0	-0.264704	-1.598908	-0.913197
6	6	0	0.612766	-2.321473	-0.123878
7	6	0	-1.682988	-2.080631	-1.097752
8	7	0	-2.582989	-0.950091	-1.468759
9	6	0	-3.127086	-0.143974	-0.322309
10	6	0	-2.020426	0.457110	0.486343
11	7	0	-1.372628	1.520002	-0.059149
12	6	0	-0.215825	1.985542	0.470267
13	6	0	0.253848	1.493763	1.643883
14	6	0	-0.431877	0.430695	2.334798
15	6	0	-1.603564	-0.076299	1.659492
16	6	0	0.530855	3.010034	-0.332702
17	7	0	1.776123	2.436456	-0.940898
18	6	0	1.588698	1.354702	-1.968070
19	1	0	3.244100	-0.220346	-0.468209
20	1	0	0.299447	-3.229003	0.377496
21	1	0	-2.067794	-2.540672	-0.189836
22	1	0	-1.733146	-2.804511	-1.910339
23	1	0	-3.717745	-0.819813	0.288584
24	1	0	-3.768181	0.620795	-0.757125
25	1	0	1.176816	1.881341	2.056681
26	1	0	-2.125618	-0.927843	2.075578
27	1	0	-0.072661	3.397943	-1.150852
28	1	0	0.852010	3.831784	0.302865
29	1	0	2.392888	2.098137	-0.193688
30	1	0	2.519019	1.316171	-2.530436
31	1	0	0.778029	1.669568	-2.620384
32	8	0	2.843427	-2.461446	0.757156
33	8	0	-0.022227	-0.045541	3.412293
34	1	0	-3.382667	-1.319339	-1.988422
35	1	0	-1.671832	1.867787	-0.964586
36	1	0	2.270783	3.213022	-1.390061
37	1	0	-2.058977	-0.335521	-2.105500
38	1	0	2.484526	-3.273304	1.143600

Most stable energy, Gibbs free energy (Ha), and geometry for protomer ./OH_Py2N2//4_11
E: -913.503551
G: -913.218631
Geometry:
Input orientation:

Center	Atomic	Atomic	Coordinates (Angstroms)

Number	Number	Type	X	Y	Z
1	6	0	0.266000	0.582139	2.336180
2	6	0	1.547082	0.523963	1.676795
3	6	0	1.764758	1.151017	0.494131
4	7	0	0.773848	1.888471	-0.076049
5	6	0	-0.477926	1.929679	0.447253
6	6	0	-0.750996	1.320489	1.627473
7	6	0	-1.534961	2.620952	-0.366321
8	7	0	-2.524266	1.646051	-0.937648
9	6	0	-2.009945	0.673207	-1.956830
10	6	0	-1.267997	-0.471662	-1.328240
11	7	0	0.068233	-0.516206	-1.501438
12	6	0	0.866528	-1.413658	-0.908909
13	6	0	0.303751	-2.402490	-0.137635
14	6	0	-1.081310	-2.433377	0.011186
15	6	0	-1.876218	-1.442043	-0.585941
16	6	0	2.347698	-1.255235	-1.142221
17	7	0	2.693850	0.115813	-1.491153
18	6	0	3.006435	0.955197	-0.326622
19	1	0	2.334895	-0.075911	2.112898
20	1	0	-1.754043	1.362224	2.033302
21	1	0	-1.110617	3.170276	-1.204058
22	1	0	-2.109457	3.300868	0.258023
23	1	0	-1.379328	1.230119	-2.647396
24	1	0	-2.880598	0.294626	-2.487018
25	1	0	0.933045	-3.131749	0.355493
26	1	0	-2.947609	-1.436399	-0.438131
27	1	0	2.866620	-1.620133	-0.249914
28	1	0	2.619734	-1.910847	-1.971207
29	1	0	3.494736	0.109961	-2.109657
30	1	0	3.370319	1.916256	-0.690610
31	1	0	3.772422	0.507810	0.312024
32	8	0	0.040409	0.002563	3.418402
33	8	0	-1.709723	-3.360488	0.725915
34	1	0	-3.259354	2.198390	-1.391581
35	1	0	0.947515	2.326294	-0.975363
36	1	0	0.540253	0.205234	-2.048536
37	1	0	-2.983710	1.137332	-0.172861
38	1	0	-1.086272	-3.999643	1.103496

Most stable energy, Gibbs free energy (Ha), and geometry for protomer ./OH_Py2N2//4_9
E: -913.487593
G: -913.203180
Geometry:

Input orientation:

Center Number	Atomic Number	Atomic Type	Coordinates (Angstroms) X	Y	Z
1	6	0	-1.144626	-0.473515	-2.027385
2	6	0	-0.737431	-1.623227	-1.345393
3	6	0	-1.038485	-1.748528	-0.015687
4	7	0	-1.746698	-0.788452	0.600429
5	6	0	-2.079606	0.370323	-0.001111
6	6	0	-1.813884	0.542145	-1.330505
7	6	0	-2.635146	1.464153	0.858507
8	7	0	-1.553181	2.108648	1.671930
9	6	0	-0.423075	2.708591	0.899148

10	6	0	0.624437	1.661406	0.590077
11	7	0	0.613766	0.584041	1.381481
12	6	0	1.494151	-0.384321	1.081362
13	6	0	2.428454	-0.288043	0.079201
14	6	0	2.484484	0.872813	-0.753322
15	6	0	1.496446	1.856197	-0.454463
16	6	0	1.393026	-1.648704	1.897309
17	7	0	0.911656	-2.817362	1.075301
18	6	0	-0.560653	-2.898905	0.818933
19	1	0	-0.174540	-2.394242	-1.854135
20	1	0	-2.078446	1.469088	-1.822633
21	1	0	-3.373674	1.076683	1.558171
22	1	0	-3.079659	2.236612	0.237823
23	1	0	-0.006361	3.497790	1.523319
24	1	0	-0.829091	3.153401	-0.007224
25	1	0	3.122480	-1.101528	-0.102934
26	1	0	1.430385	2.749405	-1.064412
27	1	0	2.366539	-1.947257	2.279760
28	1	0	0.695559	-1.532375	2.723128
29	1	0	1.171577	-3.678103	1.566222
30	1	0	-1.055660	-2.917913	1.786879
31	1	0	-0.738222	-3.836221	0.296673
32	8	0	-0.841541	-0.379111	-3.310971
33	8	0	3.333307	1.002973	-1.692777
34	1	0	-1.137236	1.413590	2.308397
35	1	0	-1.920253	-0.883573	1.600395
36	1	0	1.416207	-2.831682	0.181041
37	1	0	-1.994749	2.832859	2.242986
38	1	0	-1.161557	0.452142	-3.695267

Most stable energy, Gibbs free energy (Ha), and geometry for protomer ./OH_Py2N2//4_8
E: -913.505812
G: -913.219753
Geometry:
Input orientation:

Center Number	Atomic Number	Atomic Type	Coordinates (Angstroms) X	Y	Z
1	6	0	0.423457	-0.424944	-2.335936
2	6	0	-0.272633	-1.483232	-1.648010
3	6	0	0.194098	-1.985374	-0.477596
4	7	0	1.356946	-1.534851	0.051345
5	6	0	2.015612	-0.477029	-0.491131
6	6	0	1.602397	0.065818	-1.661230
7	6	0	3.130057	0.109180	0.317950
8	7	0	2.595776	0.917543	1.467409
9	6	0	1.706626	2.058329	1.101076
10	6	0	0.282952	1.592072	0.917742
11	7	0	-0.054712	0.466480	1.536713
12	6	0	-1.287549	-0.009117	1.343482
13	6	0	-2.243625	0.654577	0.600166
14	6	0	-1.885736	1.852962	-0.012871
15	6	0	-0.588367	2.326348	0.132335
16	6	0	-1.600155	-1.345242	1.966306
17	7	0	-1.799742	-2.421324	0.935525
18	6	0	-0.561439	-3.005208	0.322994
19	1	0	-1.200824	-1.858527	-2.060437
20	1	0	2.133030	0.913356	-2.074612

21	1	0	3.727125	0.780543	-0.291688
22	1	0	3.763249	-0.664012	0.749471
23	1	0	2.094529	2.516888	0.193745
24	1	0	1.765764	2.779416	1.915510
25	1	0	-3.241158	0.252146	0.475179
26	1	0	-0.266582	3.232098	-0.366916
27	1	0	-0.792286	-1.671080	2.616748
28	1	0	-2.529573	-1.298965	2.529584
29	1	0	-2.414565	-2.074528	0.190577
30	1	0	-0.892021	-3.821662	-0.314596
31	1	0	0.039631	-3.401531	1.138885
32	8	0	0.016565	0.060713	-3.410265
33	8	0	-2.819450	2.492076	-0.744206
34	1	0	3.399673	1.277369	1.987179
35	1	0	1.654294	-1.889930	0.954552
36	1	0	-2.301299	-3.194527	1.382835
37	1	0	2.066265	0.306775	2.103322
38	1	0	-2.453233	3.300791	-1.130282

Most stable energy, Gibbs free energy (Ha), and geometry for protomer ./OH_Py2N2//4_15
E: -913.497841
G: -913.210439
Geometry:

Input orientation:

Center Number	Atomic Number	Atomic Type	Coordinates (Angstroms) X	Y	Z
1	6	0	0.011565	-2.010274	1.554168
2	6	0	1.213982	-1.818283	0.780865
3	6	0	1.159993	-1.527864	-0.542515
4	7	0	-0.035399	-1.445757	-1.180513
5	6	0	-1.208464	-1.563204	-0.508356
6	6	0	-1.217556	-1.857324	0.814970
7	6	0	-2.463265	-1.306508	-1.291415
8	7	0	-3.094507	0.004468	-0.924937
9	6	0	-2.389815	1.252399	-1.367884
10	6	0	-1.159867	1.527015	-0.547718
11	7	0	0.035501	1.444497	-1.185784
12	6	0	1.208433	1.562500	-0.513352
13	6	0	1.217360	1.858511	0.809573
14	6	0	-0.011786	2.012322	1.548523
15	6	0	-1.214024	1.819134	0.775269
16	6	0	2.463443	1.303901	-1.295418
17	7	0	3.094659	-0.006006	-0.925195
18	6	0	2.389728	-1.255362	-1.363753
19	1	0	2.175204	-1.886289	1.275744
20	1	0	-2.162434	-1.950548	1.335440
21	1	0	-3.204811	-2.069506	-1.068779
22	1	0	-2.277989	-1.282785	-2.363244
23	1	0	-3.105199	2.062552	-1.247375
24	1	0	-2.159639	1.130860	-2.424648
25	1	0	2.162207	1.952269	1.330025
26	1	0	-2.175334	1.887587	1.269972
27	1	0	2.278330	1.277146	-2.367205
28	1	0	3.204895	2.067588	-1.074826
29	1	0	3.246233	-0.035357	0.089809
30	1	0	3.105247	-2.065024	-1.240834
31	1	0	2.159344	-1.137184	-2.420842

32	8	0	0.033268	-2.272986	2.773128
33	8	0	-0.033638	2.276877	2.767047
34	1	0	-3.245172	0.036952	0.090117
35	1	0	-0.053635	-1.217840	-2.169631
36	1	0	0.053838	1.213687	-2.174234
37	1	0	4.025801	-0.019814	-1.354022
38	1	0	-4.025951	0.016869	-1.353134

Most stable energy, Gibbs free energy (Ha), and geometry for protomer ./OH_Py2N2//4_13
E: -913.503573
G: -913.218480
Geometry:
Input orientation:

Center Number	Atomic Number	Atomic Type	Coordinates (Angstroms) X	Y	Z
1	6	0	-0.259073	0.559081	-2.341799
2	6	0	0.758033	1.302391	-1.638415
3	6	0	0.483550	1.924970	-0.465528
4	7	0	-0.769889	1.893438	0.054472
5	6	0	-1.760989	1.151621	-0.509834
6	6	0	-1.541841	0.510900	-1.684870
7	6	0	-3.004783	0.967038	0.310349
8	7	0	-2.696379	0.139122	1.484143
9	6	0	-2.353313	-1.236521	1.150608
10	6	0	-0.872275	-1.401478	0.920889
11	7	0	-0.072180	-0.500345	1.505319
12	6	0	1.264441	-0.461780	1.334151
13	6	0	1.870989	-1.441652	0.603168
14	6	0	1.074064	-2.436619	0.014778
15	6	0	-0.311175	-2.399903	0.160879
16	6	0	2.008643	0.686614	1.953286
17	7	0	2.526461	1.649181	0.926158
18	6	0	1.540353	2.621183	0.344211
19	1	0	1.762298	1.336663	-2.041934
20	1	0	-2.329813	-0.092294	-2.116095
21	1	0	-3.770462	0.514667	-0.325164
22	1	0	-3.367277	1.932608	0.663599
23	1	0	-2.872073	-1.609948	0.261766
24	1	0	-2.627986	-1.882180	1.986526
25	1	0	2.942683	-1.440952	0.457456
26	1	0	-0.941762	-3.132462	-0.325660
27	1	0	1.378629	1.251072	2.638181
28	1	0	2.877934	0.310583	2.487532
29	1	0	2.986347	1.132839	0.166849
30	1	0	2.117585	3.294531	-0.284706
31	1	0	1.115352	3.178300	1.176470
32	8	0	-0.032244	-0.032386	-3.417279
33	8	0	1.700964	-3.372912	-0.689204
34	1	0	-3.498136	0.142123	2.101524
35	1	0	-0.944618	2.340823	0.948812
36	1	0	-0.542910	0.228340	2.043843
37	1	0	3.261827	2.204168	1.376387
38	1	0	1.076431	-4.013680	-1.062276

Most stable energy, Gibbs free energy (Ha), and geometry for protomer ./OH_Py2N2//4_4
E: -913.487573
G: -913.203312
Geometry:

Input orientation:

Center Number	Atomic Number	Atomic Type	Coordinates (Angstroms) X	Y	Z
1	6	0	-2.480304	0.890246	0.741912
2	6	0	-1.484809	1.867105	0.446112
3	6	0	-0.609904	1.665888	-0.594716
4	7	0	-0.603406	0.588086	-1.385616
5	6	0	-1.491153	-0.374276	-1.088220
6	6	0	-2.428540	-0.271489	-0.089594
7	6	0	-1.394853	-1.639976	-1.902591
8	7	0	-0.927663	-2.811340	-1.076643
9	6	0	0.542622	-2.904543	-0.812489
10	6	0	1.025289	-1.756307	0.022317
11	7	0	1.743261	-0.802434	-0.592178
12	6	0	2.082116	0.354793	0.008987
13	6	0	1.811109	0.531141	1.336725
14	6	0	1.130612	-0.478056	2.032073
15	6	0	0.718820	-1.626269	1.350332
16	6	0	2.649428	1.443039	-0.850010
17	7	0	1.575122	2.094976	-1.667629
18	6	0	0.447258	2.704693	-0.899190
19	1	0	-1.415034	2.760037	1.056048
20	1	0	-3.127955	-1.080778	0.090555
21	1	0	-0.691586	-1.529442	-2.724304
22	1	0	-2.368338	-1.931174	-2.290807
23	1	0	1.042531	-2.929381	-1.777780
24	1	0	0.709863	-3.842240	-0.287509
25	1	0	2.080133	1.457049	1.828392
26	1	0	0.147740	-2.392160	1.857671
27	1	0	3.097951	2.212705	-0.228722
28	1	0	3.387045	1.048920	-1.546899
29	1	0	1.155310	1.402109	-2.304118
30	1	0	0.039971	3.497845	-1.524572
31	1	0	0.853318	3.145316	0.009221
32	8	0	-3.331341	1.026592	1.678486
33	8	0	0.820844	-0.378374	3.313676
34	1	0	-1.192145	-3.670575	-1.567788
35	1	0	1.920801	-0.900377	-1.591180
36	1	0	2.024069	2.814693	-2.238619
37	1	0	-1.437186	-2.820308	-0.185059
38	1	0	1.143066	0.452355	3.697203

Most stable energy, Gibbs free energy (Ha), and geometry for protomer ./OH_Py2N2//5_5

E: -913.940300

G: -913.638773

Geometry:

Input orientation:

Center Number	Atomic Number	Atomic Type	Coordinates (Angstroms) X	Y	Z
1	6	0	-0.014708	-1.479018	1.994316
2	6	0	1.195775	-1.530061	1.210283
3	6	0	1.156622	-1.652946	-0.138764
4	7	0	-0.032321	-1.773140	-0.785133
5	6	0	-1.212463	-1.682142	-0.119005
6	6	0	-1.235900	-1.560529	1.230116

7	6	0	-2.462228	-1.641768	-0.950133
8	7	0	-3.091572	-0.278392	-0.915586
9	6	0	-2.374762	0.819703	-1.638950
10	6	0	-1.149203	1.288461	-0.907841
11	7	0	0.046712	1.034368	-1.472277
12	6	0	1.217779	1.327683	-0.878355
13	6	0	1.220580	1.985902	0.321893
14	6	0	-0.003907	2.305828	0.920866
15	6	0	-1.200498	1.943308	0.290959
16	6	0	2.471774	0.877706	-1.570319
17	7	0	3.104672	-0.294664	-0.884956
18	6	0	2.394953	-1.614080	-0.990922
19	1	0	2.150405	-1.438962	1.714177
20	1	0	-2.185467	-1.486535	1.745370
21	1	0	-3.205746	-2.326343	-0.549293
22	1	0	-2.273535	-1.879994	-1.995064
23	1	0	-3.079772	1.643676	-1.724649
24	1	0	-2.131714	0.446285	-2.631766
25	1	0	2.157053	2.232749	0.806128
26	1	0	-2.152552	2.163234	0.755685
27	1	0	2.279990	0.586012	-2.600725
28	1	0	3.206902	1.679331	-1.550540
29	1	0	3.271270	-0.067805	0.103060
30	1	0	3.105681	-2.365196	-0.654421
31	1	0	2.176589	-1.771172	-2.045358
32	8	0	-0.005522	-1.349281	3.232762
33	8	0	-0.091700	2.935242	2.080195
34	1	0	-3.260569	-0.005262	0.060327
35	1	0	-0.041024	-1.853847	-1.797100
36	1	0	0.067349	0.552380	-2.370811
37	1	0	4.031261	-0.415513	-1.307251
38	1	0	-4.016813	-0.361481	-1.350253
39	1	0	0.783419	3.136759	2.447373

Most stable energy, Gibbs free energy (Ha), and geometry for protomer ./OH_Py2N2//5_2
E: -913.947245
G: -913.648464
Geometry:
Input orientation:

Center Number	Atomic Number	Atomic Type	Coordinates (Angstroms) X	Y	Z
1	6	0	-1.093535	-0.364798	2.063738
2	6	0	-1.680221	0.721085	1.398127
3	6	0	-2.038587	0.580002	0.086828
4	7	0	-1.867205	-0.606631	-0.527738
5	6	0	-1.240856	-1.642068	0.055456
6	6	0	-0.859519	-1.553849	1.366704
7	6	0	-0.922572	-2.833484	-0.796965
8	7	0	0.544333	-2.910542	-1.088687
9	6	0	1.126816	-1.802233	-1.926349
10	6	0	1.379935	-0.548839	-1.128481
11	7	0	0.594284	0.500435	-1.390916
12	6	0	0.740845	1.590606	-0.646718
13	6	0	1.692118	1.715822	0.350720
14	6	0	2.538709	0.638448	0.578861
15	6	0	2.374387	-0.524060	-0.171867
16	6	0	-0.200387	2.736852	-0.930438

17	7	0	-1.434565	2.261688	-1.625276
18	6	0	-2.525893	1.727481	-0.742944
19	1	0	-1.813516	1.670628	1.899621
20	1	0	-0.362024	-2.384009	1.850048
21	1	0	-1.440701	-2.790828	-1.751907
22	1	0	-1.187277	-3.749864	-0.274388
23	1	0	0.428043	-1.615790	-2.737633
24	1	0	2.057918	-2.196542	-2.326545
25	1	0	1.769667	2.619669	0.942379
26	1	0	3.006676	-1.384817	0.006215
27	1	0	-0.497316	3.245246	-0.014567
28	1	0	0.274514	3.458265	-1.594180
29	1	0	-1.154965	1.541888	-2.305292
30	1	0	-3.341360	1.422780	-1.396419
31	1	0	-2.848451	2.540573	-0.099335
32	8	0	-0.711754	-0.308545	3.326974
33	8	0	3.509763	0.652351	1.510695
34	1	0	0.698926	-3.791522	-1.588808
35	1	0	-2.110786	-0.682320	-1.515002
36	1	0	-1.834080	3.041216	-2.154771
37	1	0	1.067357	-2.987706	-0.208159
38	1	0	-0.905534	0.554538	3.725419
39	1	0	3.539881	1.506580	1.965476

Most stable energy, Gibbs free energy (Ha), and geometry for protomer ./OH_Py2N2//5_1

E: -913.947239

G: -913.648448

Geometry:

Input orientation:

Center Number	Atomic Number	Atomic Type	Coordinates (Angstroms) X	Y	Z
1	6	0	-2.540959	0.631398	0.577484
2	6	0	-1.697381	1.711193	0.349579
3	6	0	-0.745235	1.588495	-0.647341
4	7	0	-0.595041	0.498545	-1.391188
5	6	0	-1.377810	-0.552910	-1.128940
6	6	0	-2.372831	-0.530787	-0.172891
7	6	0	-1.120744	-1.805839	-1.926223
8	7	0	-0.535596	-2.912145	-1.087765
9	6	0	0.931113	-2.831178	-0.795872
10	6	0	1.245928	-1.638685	0.056300
11	7	0	1.869430	-0.601529	-0.526898
12	6	0	2.036880	0.585803	0.087413
13	6	0	1.677547	0.726120	1.398540
14	6	0	1.094027	-0.361412	2.064252
15	6	0	0.863860	-1.551323	1.367380
16	6	0	2.521118	1.734602	-0.742353
17	7	0	1.428577	2.265804	-1.625009
18	6	0	0.192705	2.737485	-0.930859
19	1	0	-1.777949	2.614981	0.940937
20	1	0	-3.002768	-1.393301	0.005047
21	1	0	-0.422018	-1.617754	-2.737170
22	1	0	-2.050513	-2.202918	-2.326772
23	1	0	1.449202	-2.787453	-1.750786
24	1	0	1.198158	-3.746706	-0.272987
25	1	0	1.807758	1.676176	1.899850
26	1	0	0.368693	-2.382864	1.850745

27	1	0	2.841312	2.548589	-0.098705
28	1	0	3.337577	1.432253	-1.395667
29	1	0	1.151212	1.545250	-2.305140
30	1	0	-0.284012	3.457316	-1.595009
31	1	0	0.487616	3.246976	-0.014950
32	8	0	-3.512584	0.642649	1.508757
33	8	0	0.711724	-0.306157	3.327368
34	1	0	-0.687839	-3.793822	-1.587383
35	1	0	2.113646	-0.676765	-1.514020
36	1	0	1.826152	3.046408	-2.154378
37	1	0	-1.058576	-2.990178	-0.207287
38	1	0	-3.545500	1.496906	1.963279
39	1	0	0.902673	0.557545	3.725838

Most stable energy, Gibbs free energy (Ha), and geometry for protomer ./OH_Py2N2//5_6
E: -913.940296
G: -913.638710
Geometry:
Input orientation:

Center Number	Atomic Number	Atomic Type	Coordinates (Angstroms) X	Y	Z
1	6	0	-0.003419	-2.310433	0.905902
2	6	0	1.221186	-1.986526	0.309346
3	6	0	1.218759	-1.321679	-0.887188
4	7	0	0.047917	-1.025260	-1.480045
5	6	0	-1.148118	-1.283343	-0.917751
6	6	0	-1.199839	-1.945152	0.277218
7	6	0	-2.373276	-0.809824	-1.646193
8	7	0	-3.091186	0.281832	-0.914006
9	6	0	-2.464004	1.646497	-0.937949
10	6	0	-1.213601	1.681983	-0.107677
11	7	0	-0.033865	1.776957	-0.774014
12	6	0	1.155449	1.654150	-0.128843
13	6	0	1.195392	1.523834	1.219453
14	6	0	-0.014652	1.466802	2.003737
15	6	0	-1.236271	1.552530	1.240678
16	6	0	2.393705	1.621082	-0.981306
17	7	0	3.104375	0.301630	-0.882386
18	6	0	2.473237	-0.867243	-1.575256
19	1	0	2.157558	-2.235579	0.792630
20	1	0	-2.152021	-2.168177	0.740185
21	1	0	-3.077746	-1.633402	-1.739404
22	1	0	-2.129379	-0.427959	-2.635555
23	1	0	-3.208232	2.326376	-0.530467
24	1	0	-2.276744	1.894172	-1.980922
25	1	0	2.150331	1.431084	1.722463
26	1	0	-2.185533	1.475428	1.756041
27	1	0	2.175305	1.783963	-2.034884
28	1	0	3.103770	2.370907	-0.640542
29	1	0	3.269758	0.068887	0.104460
30	1	0	3.208811	-1.668537	-1.558785
31	1	0	2.282706	-0.570067	-2.604307
32	8	0	-0.091104	-2.945990	2.061827
33	8	0	-0.004771	1.329195	3.241340
34	1	0	-3.258788	0.000865	0.059948
35	1	0	0.069010	-0.538003	-2.375741
36	1	0	-0.043195	1.862222	-1.785642

37	1	0	4.031389	0.425756	-1.302769
38	1	0	-4.016897	0.366957	-1.347222
39	1	0	0.784187	-3.147394	2.428713

Most stable energy, Gibbs free energy (Ha), and geometry for protomer ./OH_Py2N2//5_3
E: -913.946515
G: -913.647747
Geometry:
Input orientation:

Center Number	Atomic Number	Atomic Type	Coordinates (Angstroms) X	Y	Z
1	6	0	0.296471	-2.116393	1.581152
2	6	0	-1.028751	-1.821197	1.262118
3	6	0	-1.332684	-1.421858	-0.015767
4	7	0	-0.349974	-1.348585	-0.925871
5	6	0	0.950402	-1.568585	-0.643607
6	6	0	1.301138	-1.975339	0.611083
7	6	0	1.947170	-1.322210	-1.739850
8	7	0	2.774033	-0.091652	-1.497553
9	6	0	2.127941	1.232251	-1.780819
10	6	0	1.041299	1.538642	-0.792080
11	7	0	-0.229714	1.530017	-1.237688
12	6	0	-1.298062	1.634415	-0.423061
13	6	0	-1.097352	1.864477	0.911533
14	6	0	0.210126	1.951642	1.402964
15	6	0	1.291914	1.768396	0.532236
16	6	0	-2.641687	1.350380	-1.029862
17	7	0	-2.669324	-0.026844	-1.528836
18	6	0	-2.714683	-1.031385	-0.473420
19	1	0	-1.806779	-1.876753	2.012124
20	1	0	2.336418	-2.164394	0.860563
21	1	0	1.458313	-1.197474	-2.704432
22	1	0	2.646566	-2.153375	-1.790235
23	1	0	1.747423	1.194772	-2.798986
24	1	0	2.916827	1.978281	-1.712287
25	1	0	-1.944815	1.936800	1.580394
26	1	0	2.307183	1.782047	0.905477
27	1	0	-3.405024	1.541802	-0.271739
28	1	0	-2.806384	2.023562	-1.871553
29	1	0	-3.477149	-0.136048	-2.128383
30	1	0	-3.198364	-1.927984	-0.865006
31	1	0	-3.278515	-0.708539	0.407143
32	8	0	0.672646	-2.506510	2.791763
33	8	0	0.480596	2.162110	2.680785
34	1	0	3.593219	-0.156982	-2.111886
35	1	0	-0.619576	-1.041598	-1.861482
36	1	0	-0.398829	1.339736	-2.225017
37	1	0	3.139725	-0.101844	-0.536974
38	1	0	-0.084977	-2.569764	3.394105
39	1	0	-0.329703	2.258030	3.206376

Most stable energy, Gibbs free energy (Ha), and geometry for protomer ./Cl_Py2N2//0_1
E: -1681.313002
G: -1681.088568
Geometry:
Input orientation:

Center Atomic Atomic Coordinates (Angstroms)

Number	Number	Type	X	Y	Z
1	6	0	-1.120483	2.015300	-0.151677
2	6	0	-0.337159	1.858117	-1.279127
3	6	0	0.987638	1.475399	-1.094037
4	7	0	1.518406	1.307451	0.117159
5	6	0	0.736747	1.403682	1.191269
6	6	0	-0.602252	1.768614	1.106595
7	6	0	1.370934	1.097727	2.536556
8	7	0	2.406035	0.066043	2.547430
9	6	0	1.895139	-1.309766	2.499743
10	6	0	1.082296	-1.596122	1.262578
11	7	0	1.749715	-1.585055	0.102482
12	6	0	1.060277	-1.559400	-1.032651
13	6	0	-0.332086	-1.641648	-1.066320
14	6	0	-0.999451	-1.723445	0.135829
15	6	0	-0.297810	-1.692917	1.331063
16	6	0	1.817207	-1.325207	-2.321947
17	7	0	1.506070	-0.072138	-3.013242
18	6	0	1.852275	1.159218	-2.295506
19	1	0	-0.740522	2.002504	-2.272963
20	1	0	-1.218110	1.835798	1.993420
21	1	0	0.587101	0.819715	3.242518
22	1	0	1.810331	2.028460	2.905450
23	1	0	1.287370	-1.484492	3.387528
24	1	0	2.751326	-1.983578	2.534753
25	1	0	-0.865489	-1.611621	-2.007885
26	1	0	-0.808121	-1.690827	2.284774
27	1	0	2.883987	-1.341137	-2.100878
28	1	0	1.604354	-2.140623	-3.014870
29	1	0	1.780045	1.983326	-3.006481
30	1	0	2.889332	1.084491	-1.969407
31	1	0	0.518851	-0.052967	-3.253023
32	1	0	2.991637	0.207510	1.728930
33	17	0	-2.781059	2.487304	-0.317368
34	17	0	-2.730548	-1.792242	0.160043

Most stable energy, Gibbs free energy (Ha), and geometry for protomer ./Cl_Py2N2//1_3
E: -1681.772845
G: -1681.534147
Geometry:

Input orientation:

Center Number	Atomic Number	Atomic Type	Coordinates (Angstroms) X	Y	Z
1	6	0	-0.590065	1.670484	-1.373195
2	6	0	0.747436	1.898230	-1.098414
3	6	0	1.147890	1.887859	0.230771
4	7	0	0.280523	1.721521	1.230810
5	6	0	-0.985153	1.448726	0.944424
6	6	0	-1.483953	1.411413	-0.350009
7	6	0	-1.873816	1.131492	2.120653
8	7	0	-1.151066	0.260727	3.100787
9	6	0	-1.188315	-1.210227	2.811743
10	6	0	-0.404250	-1.491736	1.561316
11	7	0	0.859190	-1.071481	1.579627
12	6	0	1.564637	-1.101299	0.454263
13	6	0	1.054283	-1.648773	-0.721278

14	6	0	-0.232988	-2.147706	-0.698256
15	6	0	-1.003578	-2.059021	0.451433
16	6	0	2.953577	-0.495239	0.497982
17	7	0	3.087569	0.728602	1.285350
18	6	0	2.607511	1.938039	0.612541
19	1	0	1.469021	2.038479	-1.891656
20	1	0	-2.518942	1.166477	-0.544798
21	1	0	-2.782464	0.618330	1.814844
22	1	0	-2.138851	2.046020	2.649872
23	1	0	-2.230767	-1.501231	2.717081
24	1	0	-0.742276	-1.704424	3.673010
25	1	0	1.644875	-1.658919	-1.627562
26	1	0	-2.035230	-2.381009	0.473444
27	1	0	3.623842	-1.250924	0.915319
28	1	0	3.289723	-0.299419	-0.520700
29	1	0	3.207821	2.102396	-0.281891
30	1	0	2.769092	2.780667	1.285708
31	1	0	-1.541724	0.414110	4.032266
32	1	0	2.557220	0.610431	2.144197
33	1	0	-0.167329	0.566265	3.119937
34	17	0	-1.135993	1.638301	-3.012878
35	17	0	-0.918684	-2.827060	-2.132799

Most stable energy, Gibbs free energy (Ha), and geometry for protomer ./Cl_Py2N2//1_2
E: -1681.767562
G: -1681.531027
Geometry:
Input orientation:

Center Number	Atomic Number	Atomic Type	Coordinates (Angstroms) X	Y	Z
1	6	0	-3.957452	0.047415	0.305755
2	6	0	-3.405973	-1.169835	-0.042616
3	6	0	-2.068774	-1.203162	-0.402015
4	7	0	-1.370700	-0.067383	-0.394995
5	6	0	-1.873547	1.125119	-0.030501
6	6	0	-3.199098	1.214293	0.320591
7	6	0	-0.906428	2.277437	0.019097
8	7	0	-0.015677	2.318934	-1.138277
9	6	0	1.393191	2.467802	-0.811797
10	6	0	2.055012	1.175489	-0.394423
11	7	0	1.331146	0.065977	-0.379369
12	6	0	1.875247	-1.101893	-0.024327
13	6	0	3.208275	-1.207240	0.323477
14	6	0	3.964148	-0.041730	0.308261
15	6	0	3.403538	1.167633	-0.043346
16	6	0	0.932452	-2.281820	0.019487
17	7	0	0.016397	-2.304828	-1.122873
18	6	0	-1.382812	-2.483519	-0.797295
19	1	0	-3.989279	-2.079849	-0.043826
20	1	0	-3.627454	2.165682	0.601161
21	1	0	-0.320354	2.158207	0.935600
22	1	0	-1.481156	3.196808	0.130107
23	1	0	1.570972	3.206784	-0.020508
24	1	0	1.911806	2.832705	-1.699715
25	1	0	3.642224	-2.159366	0.595392
26	1	0	3.984792	2.079741	-0.056641
27	1	0	1.511342	-3.202917	0.091774

28	1	0	0.353108	-2.199840	0.944590
29	1	0	-1.558255	-3.204023	0.011069
30	1	0	-1.904485	-2.866177	-1.675847
31	1	0	-0.323337	-0.073949	-0.604066
32	1	0	0.297110	-3.026750	-1.771245
33	1	0	-0.289570	3.087792	-1.734617
34	17	0	-5.621830	0.126908	0.735282
35	17	0	5.640318	-0.114868	0.733011

Most stable energy, Gibbs free energy (Ha), and geometry for protomer ./Cl_Py2N2//1_1
E: -1681.767562
G: -1681.531024
Geometry:
Input orientation:

Center Number	Atomic Number	Atomic Type	Coordinates (Angstroms) X	Y	Z
1	6	0	3.964315	0.043506	0.304715
2	6	0	3.207967	1.208706	0.320201
3	6	0	1.874916	1.102850	-0.027359
4	7	0	1.331231	-0.065201	-0.382442
5	6	0	2.055550	-1.174414	-0.397766
6	6	0	3.404136	-1.166045	-0.046929
7	6	0	1.394179	-2.466948	-0.815171
8	7	0	-0.014797	-2.318605	-1.141425
9	6	0	-0.905396	-2.277611	0.016084
10	6	0	-1.872977	-1.125664	-0.033216
11	7	0	-1.370671	0.067086	-0.397641
12	6	0	-2.069206	1.202585	-0.404402
13	6	0	-3.406329	1.168675	-0.044777
14	6	0	-3.957261	-0.048842	0.303531
15	6	0	-3.198435	-1.215414	0.318080
16	6	0	-1.383823	2.483271	-0.799636
17	7	0	0.015408	2.305182	-1.125441
18	6	0	0.931630	2.282374	0.016788
19	1	0	3.641572	2.160979	0.592148
20	1	0	3.985758	-2.077916	-0.060423
21	1	0	1.572371	-3.205936	-0.023982
22	1	0	1.912805	-2.831555	-1.703203
23	1	0	-0.319236	-2.158274	0.932518
24	1	0	-1.479751	-3.197221	0.127048
25	1	0	-3.989997	2.078456	-0.045759
26	1	0	-3.626359	-2.167015	0.598591
27	1	0	-1.905783	2.865820	-1.678065
28	1	0	-1.559440	3.203606	0.008841
29	1	0	0.352443	2.199972	0.941953
30	1	0	1.510135	3.203705	0.089166
31	1	0	-0.323346	0.074102	-0.606881
32	1	0	0.295743	3.027305	-1.773754
33	1	0	-0.288469	-3.087502	-1.737817
34	17	0	5.640531	0.117276	0.729181
35	17	0	-5.621537	-0.129060	0.733331

Most stable energy, Gibbs free energy (Ha), and geometry for protomer ./Cl_Py2N2//1_4
E: -1681.772857
G: -1681.534439
Geometry:
Input orientation:

Center Number	Atomic Number	Atomic Type	Coordinates (Angstroms) X	Y	Z
1	6	0	1.107893	1.937435	0.027228
2	6	0	0.383933	1.703584	-1.128204
3	6	0	-0.973394	1.458412	-0.980210
4	7	0	-1.588658	1.445894	0.194793
5	6	0	-0.863736	1.591287	1.304816
6	6	0	0.496133	1.869217	1.266930
7	6	0	-1.571546	1.306740	2.606897
8	7	0	-2.078631	-0.063523	2.715871
9	6	0	-1.051051	-1.097469	2.617322
10	6	0	-0.568723	-1.421764	1.216869
11	7	0	-1.478788	-1.421626	0.249128
12	6	0	-1.084341	-1.602451	-1.009784
13	6	0	0.219963	-1.893075	-1.365671
14	6	0	1.148413	-1.959096	-0.337398
15	6	0	0.773626	-1.700568	0.966001
16	6	0	-2.145046	-1.374880	-2.050281
17	7	0	-2.741501	-0.012544	-1.866539
18	6	0	-1.834675	1.137961	-2.175200
19	1	0	0.854188	1.686836	-2.101587
20	1	0	1.061933	1.986909	2.181137
21	1	0	-2.419155	1.985508	2.706363
22	1	0	-0.890070	1.494952	3.436044
23	1	0	-1.447797	-2.022432	3.043669
24	1	0	-0.196690	-0.807263	3.229768
25	1	0	0.504502	-2.026405	-2.399962
26	1	0	1.498669	-1.691351	1.768945
27	1	0	-1.747383	-1.427904	-3.059887
28	1	0	-2.961281	-2.086348	-1.936826
29	1	0	-3.040211	0.082591	-0.885143
30	1	0	-2.474923	1.985339	-2.416330
31	1	0	-1.239272	0.878222	-3.046984
32	1	0	-3.575805	0.055581	-2.452728
33	1	0	-2.750553	-0.213696	1.968221
34	17	0	2.805373	2.245344	-0.077574
35	17	0	2.802200	-2.303664	-0.705690

Most stable energy, Gibbs free energy (Ha), and geometry for protomer ./Cl_Py2N2//2_1
E: -1682.214279
G: -1681.961693
Geometry:
Input orientation:

Center Number	Atomic Number	Atomic Type	Coordinates (Angstroms) X	Y	Z
1	6	0	-1.165150	2.240826	0.001713
2	6	0	-0.533167	1.997139	1.213477
3	6	0	0.764976	1.538911	1.190332
4	7	0	1.363513	1.372653	0.001099
5	6	0	0.765052	1.540802	-1.187917
6	6	0	-0.533090	1.999060	-1.210405
7	6	0	1.557977	1.196504	-2.419893
8	7	0	2.423408	0.043204	-2.204536
9	6	0	1.756601	-1.242627	-2.402021
10	6	0	0.939900	-1.590893	-1.189825
11	7	0	1.555797	-1.501306	-0.001165

12	6	0	0.939948	-1.592722	1.187386
13	6	0	-0.399758	-1.907572	1.210428
14	6	0	-1.055542	-2.083472	-0.001550
15	6	0	-0.399798	-1.905768	-1.213279
16	6	0	1.756766	-1.246420	2.400055
17	7	0	2.423409	0.039802	2.204591
18	6	0	1.557765	1.192577	2.421832
19	1	0	-1.042193	2.131872	2.156998
20	1	0	-1.042054	2.135313	-2.153744
21	1	0	2.183709	2.058137	-2.660818
22	1	0	0.851627	1.056904	-3.243153
23	1	0	2.520786	-2.009485	-2.537894
24	1	0	1.095968	-1.254505	-3.272782
25	1	0	-0.920663	-1.977297	2.154444
26	1	0	-0.920744	-1.974079	-2.157373
27	1	0	1.096257	-1.259762	3.270888
28	1	0	2.521036	-2.013439	2.534554
29	1	0	2.183297	2.053898	2.664384
30	1	0	0.851304	1.051384	3.244726
31	1	0	2.321023	1.015450	0.000890
32	1	0	2.543202	-1.238928	-0.000996
33	1	0	3.214903	0.097843	2.831927
34	1	0	3.214897	0.102127	-2.831797
35	17	0	-2.785267	2.800761	0.002120
36	17	0	-2.727253	-2.457946	-0.001813

Most stable energy, Gibbs free energy (Ha), and geometry for protomer ./Cl_Py2N2//2_3
E: -1682.214461
G: -1681.960895
Geometry:

Input orientation:

Center Number	Atomic Number	Atomic Type	Coordinates (Angstroms) X	Y	Z
1	6	0	-1.965331	-1.387696	-0.279626
2	6	0	-1.467467	-0.829093	-1.441374
3	6	0	-1.227635	0.533355	-1.461844
4	7	0	-1.516305	1.251415	-0.376412
5	6	0	-1.926579	0.728539	0.793818
6	6	0	-2.183590	-0.615705	0.863451
7	6	0	-2.022588	1.640010	1.978279
8	7	0	-0.681951	2.160004	2.407951
9	6	0	0.359248	1.130327	2.699862
10	6	0	1.062282	0.714599	1.430634
11	7	0	1.116530	1.613861	0.458544
12	6	0	1.646578	1.266243	-0.718398
13	6	0	2.222699	0.022757	-0.924832
14	6	0	2.199012	-0.886984	0.121498
15	6	0	1.591457	-0.564406	1.319296
16	6	0	1.497899	2.243299	-1.856016
17	7	0	0.077654	2.471521	-2.158758
18	6	0	-0.615000	1.277406	-2.619705
19	1	0	-1.237775	-1.428565	-2.311060
20	1	0	-2.511861	-1.058626	1.792821
21	1	0	-2.442255	1.100434	2.822066
22	1	0	-2.638945	2.508616	1.751750
23	1	0	-0.122016	0.284798	3.186739
24	1	0	1.065573	1.585097	3.393263

25	1	0	2.645391	-0.237209	-1.885313
26	1	0	1.512080	-1.274874	2.130709
27	1	0	1.937587	3.200082	-1.574455
28	1	0	2.037630	1.854029	-2.723968
29	1	0	0.028067	0.576398	-3.162083
30	1	0	-1.425511	1.565003	-3.292122
31	1	0	-0.839036	2.721487	3.249312
32	1	0	-1.252960	2.241260	-0.439938
33	1	0	0.009872	3.188711	-2.869409
34	1	0	-0.300673	2.793144	1.691729
35	17	0	-2.259914	-3.072980	-0.211276
36	17	0	2.880525	-2.457419	-0.098566

Most stable energy, Gibbs free energy (Ha), and geometry for protomer ./Cl_Py2N2//2_6
E: -1682.227240
G: -1681.972123
Geometry:
Input orientation:

Center Number	Atomic Number	Atomic Type	Coordinates (Angstroms) X	Y	Z
1	6	0	-1.981331	1.194503	-0.323779
2	6	0	-2.138820	0.448955	0.834158
3	6	0	-1.812094	-0.894728	0.771200
4	7	0	-1.394580	-1.488834	-0.344039
5	6	0	-1.195203	-0.746553	-1.425046
6	6	0	-1.480440	0.610789	-1.473205
7	6	0	-0.611010	-1.454793	-2.619169
8	7	0	0.477291	-2.393035	-2.202603
9	6	0	1.825067	-1.772470	-1.995713
10	6	0	1.806245	-0.902858	-0.770667
11	7	0	1.384891	-1.493369	0.345046
12	6	0	1.189774	-0.748960	1.425363
13	6	0	1.483171	0.606711	1.472321
14	6	0	1.987896	1.186273	0.322481
15	6	0	2.141035	0.438756	-0.834779
16	6	0	0.602146	-1.452777	2.620451
17	7	0	-0.489317	-2.387932	2.205351
18	6	0	-1.834843	-1.762868	1.997199
19	1	0	-2.470534	0.899715	1.759170
20	1	0	-1.294498	1.187373	-2.368833
21	1	0	-0.194548	-0.753791	-3.338101
22	1	0	-1.375858	-2.058375	-3.106219
23	1	0	2.069072	-1.208326	-2.891735
24	1	0	2.528355	-2.594694	-1.874349
25	1	0	1.300689	1.185187	2.367454
26	1	0	2.475905	0.886584	-1.760071
27	1	0	1.364603	-2.058126	3.109062
28	1	0	0.187982	-0.748890	3.337911
29	1	0	-0.584815	-3.114465	2.918317
30	1	0	-2.077064	-1.196714	2.892442
31	1	0	-2.541060	-2.582677	1.876549
32	1	0	0.570112	-3.121163	-2.914286
33	1	0	-0.201033	-2.851934	1.333099
34	1	0	0.187550	-2.854490	-1.329500
35	17	0	-2.349313	2.879234	-0.311651
36	17	0	2.366668	2.868588	0.309025

Most stable energy, Gibbs free energy (Ha), and geometry for protomer ./Cl_Py2N2//2_2

E: -1682.214465
G: -1681.960859
Geometry:
Input orientation:

Center Number	Atomic Number	Atomic Type	Coordinates (Angstroms) X	Y	Z
1	6	0	-2.201494	-0.879132	0.126457
2	6	0	-2.224661	0.031781	-0.918864
3	6	0	-1.643651	1.273008	-0.712653
4	7	0	-1.109394	1.617523	0.463275
5	6	0	-1.055856	0.717080	1.434318
6	6	0	-1.589624	-0.560008	1.322997
7	6	0	-0.348538	1.129381	2.702235
8	7	0	0.695901	2.155328	2.408657
9	6	0	2.033372	1.630491	1.975203
10	6	0	1.931118	0.721144	0.789623
11	7	0	1.519600	1.247061	-0.378816
12	6	0	1.226238	0.531536	-1.464668
13	6	0	1.462278	-0.831611	-1.446943
14	6	0	1.961170	-1.393498	-0.287240
15	6	0	2.184143	-0.623967	0.856584
16	6	0	0.612889	1.279009	-2.619941
17	7	0	-0.074587	2.474819	-2.155726
18	6	0	-1.494750	2.250874	-1.849533
19	1	0	-2.650893	-0.225662	-1.878460
20	1	0	-1.510657	-1.271585	2.133481
21	1	0	0.130587	0.281808	3.187651
22	1	0	-1.051669	1.586424	3.397379
23	1	0	2.452826	1.087991	2.817221
24	1	0	2.652774	2.496889	1.748526
25	1	0	1.229038	-1.428998	-2.317120
26	1	0	2.513191	-1.069366	1.784490
27	1	0	1.422485	1.564720	-3.294254
28	1	0	-0.033958	0.580745	-3.161371
29	1	0	-2.038060	1.864399	-2.716503
30	1	0	-1.930571	3.208746	-1.565661
31	1	0	0.857209	2.715420	3.250139
32	1	0	1.258937	2.237769	-0.440112
33	1	0	-0.006294	3.192709	-2.865619
34	1	0	0.315128	2.790547	1.694023
35	17	0	-2.889690	-2.446672	-0.093392
36	17	0	2.251315	-3.079689	-0.222276

Most stable energy, Gibbs free energy (Ha), and geometry for protomer ./Cl_Py2N2//2_5
E: -1682.214464
G: -1681.960886
Geometry:
Input orientation:

Center Number	Atomic Number	Atomic Type	Coordinates (Angstroms) X	Y	Z
1	6	0	-1.967540	-1.385456	0.277861
2	6	0	-2.182798	-0.614613	-0.866542
3	6	0	-1.924440	0.729443	-0.798026
4	7	0	-1.515993	1.253319	0.372403
5	6	0	-1.230205	0.536242	1.459259

6	6	0	-1.471363	-0.825976	1.439913
7	6	0	-0.618951	1.280989	2.617409
8	7	0	0.076886	2.473167	2.156196
9	6	0	1.497330	2.241929	1.856655
10	6	0	1.646632	1.263352	0.720405
11	7	0	1.119564	1.610620	-0.457977
12	6	0	1.065695	0.710408	-1.429207
13	6	0	1.592508	-0.569355	-1.315458
14	6	0	2.197074	-0.891713	-0.116089
15	6	0	2.220204	0.019081	0.929332
16	6	0	0.365798	1.125977	-2.700232
17	7	0	-0.674551	2.157321	-2.411233
18	6	0	-2.016768	1.639490	-1.983881
19	1	0	-2.509661	-1.058284	-1.796059
20	1	0	-1.243894	-1.424669	2.310715
21	1	0	0.021590	0.579668	3.162369
22	1	0	-1.430507	1.571137	3.287477
23	1	0	2.034524	1.852644	2.726179
24	1	0	1.939378	3.197584	1.574959
25	1	0	1.513527	-1.280545	-2.126278
26	1	0	2.640418	-0.240635	1.890967
27	1	0	1.074119	1.579187	-3.392620
28	1	0	-0.115671	0.280687	-3.187331
29	1	0	-0.829200	2.718315	-3.253376
30	1	0	-2.435230	1.099376	-2.827916
31	1	0	-2.632538	2.509144	-1.759793
32	1	0	-1.251867	2.242965	0.435338
33	1	0	-0.293876	2.790565	-1.694793
34	1	0	0.008999	3.191445	2.865741
35	17	0	-2.263537	-3.070542	0.210950
36	17	0	2.875315	-2.463148	0.107030

Most stable energy, Gibbs free energy (Ha), and geometry for protomer ./Cl_Py2N2//2_4
E: -1682.214460
G: -1681.960885
Geometry:

Input orientation:

Center Number	Atomic Number	Atomic Type	Coordinates (Angstroms) X	Y	Z
1	6	0	-2.199510	0.885226	-0.121712
2	6	0	-1.591874	0.562754	-1.319502
3	6	0	-1.061956	-0.715958	-1.430620
4	7	0	-1.115540	-1.615041	-0.458320
5	6	0	-1.645680	-1.267483	0.718592
6	6	0	-2.222542	-0.024305	0.924812
7	6	0	-1.496282	-2.244181	1.856421
8	7	0	-0.075864	-2.471320	2.159151
9	6	0	0.615927	-1.276609	2.619830
10	6	0	1.228075	-0.532369	1.461832
11	7	0	1.516979	-1.250359	0.376419
12	6	0	1.926920	-0.727354	-0.793880
13	6	0	2.183308	0.617003	-0.863597
14	6	0	1.964847	1.388950	0.279474
15	6	0	1.467318	0.830184	1.441283
16	6	0	2.023526	-1.638827	-1.978297
17	7	0	0.683365	-2.160188	-2.407766
18	6	0	-0.358840	-1.131572	-2.699828

19	1	0	-1.513015	1.273091	-2.131081
20	1	0	-2.645306	0.235602	1.885277
21	1	0	-1.935305	-3.201334	1.575083
22	1	0	-2.036255	-1.855098	2.724307
23	1	0	1.426627	-1.563450	3.292340
24	1	0	-0.027658	-0.575934	3.162025
25	1	0	2.511316	1.060028	-1.793011
26	1	0	1.237475	1.429585	2.310978
27	1	0	2.442542	-1.098905	-2.822189
28	1	0	2.640778	-2.506810	-1.751816
29	1	0	0.302722	-2.793592	-1.691446
30	1	0	-1.064915	-1.587277	-3.392870
31	1	0	0.121539	-0.285821	-3.187190
32	1	0	1.254105	-2.240327	0.440040
33	1	0	0.840938	-2.721661	-3.249044
34	1	0	-0.007526	-3.188329	2.869932
35	17	0	-2.881990	2.455279	0.098075
36	17	0	2.258777	3.074346	0.211047

Most stable energy, Gibbs free energy (Ha), and geometry for protomer ./CF3_Py2N2//0_1
E: -1436.287138
G: -1436.040065
Geometry:

Input orientation:

Center Number	Atomic Number	Atomic Type	Coordinates (Angstroms) X	Y	Z
1	6	0	-0.093758	1.975881	0.008239
2	6	0	0.688544	1.885447	-1.134788
3	6	0	1.945468	1.316360	-1.016810
4	7	0	2.433611	0.914085	0.160565
5	6	0	1.661838	0.946437	1.238873
6	6	0	0.373093	1.482563	1.208985
7	6	0	2.230907	0.336126	2.505271
8	7	0	2.958226	-0.921663	2.329233
9	6	0	2.100632	-2.099957	2.161447
10	6	0	1.171030	-2.008101	0.976251
11	7	0	1.754729	-1.869002	-0.217982
12	6	0	1.023475	-1.532432	-1.272229
13	6	0	-0.365383	-1.411950	-1.200138
14	6	0	-0.972135	-1.643572	0.016997
15	6	0	-0.202660	-1.933412	1.135504
16	6	0	1.772136	-1.243515	-2.558844
17	7	0	2.998715	-0.458279	-2.412467
18	6	0	2.779159	0.980219	-2.229048
19	1	0	0.314499	2.203437	-2.100093
20	1	0	-0.241631	1.481730	2.099214
21	1	0	1.424171	0.179727	3.222364
22	1	0	2.912036	1.068500	2.945818
23	1	0	1.515577	-2.237615	3.070779
24	1	0	2.749832	-2.968322	2.043900
25	1	0	-0.935049	-1.119655	-2.072164
26	1	0	-0.651981	-2.049969	2.113980
27	1	0	2.030477	-2.203700	-3.012330
28	1	0	1.105976	-0.731397	-3.254120
29	1	0	2.295270	1.376523	-3.121768
30	1	0	3.756280	1.455767	-2.136650
31	1	0	3.490865	-0.805961	-1.593656

32	1	0	3.532444	-0.831557	1.495195
33	6	0	-1.485850	2.524813	-0.119966
34	9	0	-2.237834	1.758315	-0.926186
35	9	0	-1.487166	3.754101	-0.654963
36	9	0	-2.123115	2.604937	1.049624
37	6	0	-2.456875	-1.489928	0.188098
38	9	0	-2.748193	-0.480525	1.024081
39	9	0	-3.013046	-2.590861	0.713786
40	9	0	-3.089102	-1.241889	-0.960554

Most stable energy, Gibbs free energy (Ha), and geometry for protomer ./CF3_Py2N2//1_3

E: -1436.743001

G: -1436.481358

Geometry:

Input orientation:

Center Number	Atomic Number	Atomic Type	Coordinates (Angstroms) X	Y	Z
1	6	0	-0.777051	1.844566	0.102609
2	6	0	-0.061862	1.890406	-1.080887
3	6	0	1.307914	1.657228	-1.035438
4	7	0	1.939073	1.447706	0.120798
5	6	0	1.237364	1.368415	1.244904
6	6	0	-0.135785	1.561897	1.295235
7	6	0	2.013185	1.027640	2.493227
8	7	0	3.142059	0.096107	2.188086
9	6	0	2.782964	-1.361518	2.137174
10	6	0	1.881152	-1.595564	0.961368
11	7	0	2.439863	-1.382183	-0.235371
12	6	0	1.663809	-1.337591	-1.308992
13	6	0	0.287488	-1.580328	-1.235099
14	6	0	-0.264271	-1.852215	-0.004999
15	6	0	0.537953	-1.855786	1.131934
16	6	0	2.281920	-0.907001	-2.619840
17	7	0	1.811138	0.376986	-3.140793
18	6	0	2.107500	1.550372	-2.314617
19	1	0	-0.555704	2.069797	-2.028738
20	1	0	-0.674560	1.466333	2.227824
21	1	0	1.379506	0.557909	3.241938
22	1	0	2.454527	1.928719	2.917501
23	1	0	2.303950	-1.609877	3.080302
24	1	0	3.718581	-1.906668	2.033963
25	1	0	-0.321902	-1.525780	-2.128265
26	1	0	0.121133	-2.004856	2.120130
27	1	0	3.361804	-0.851630	-2.487555
28	1	0	2.071427	-1.663966	-3.376280
29	1	0	1.913687	2.437181	-2.919199
30	1	0	3.167968	1.536115	-2.065229
31	1	0	3.873226	0.222094	2.891030
32	1	0	0.812124	0.329620	-3.320478
33	1	0	3.535857	0.369152	1.277015
34	6	0	-2.266229	2.049282	0.051024
35	9	0	-2.847512	1.184447	-0.791953
36	9	0	-2.579220	3.277393	-0.384136
37	9	0	-2.848839	1.892718	1.240776
38	6	0	-1.742981	-2.069628	0.159641
39	9	0	-2.273511	-1.161829	0.992870
40	9	0	-2.010835	-3.272675	0.683711

41	9	0	-2.407124	-1.982433	-0.992861

Most stable energy, Gibbs free energy (Ha), and geometry for protomer ./CF3_Py2N2//1_2
E: -1436.735654
G: -1436.478312
Geometry:

Input orientation:

Center Number	Atomic Number	Atomic Type	Coordinates (Angstroms) X	Y	Z
1	6	0	-3.952898	-0.077112	-0.168222
2	6	0	-3.369921	-1.269066	0.207993
3	6	0	-2.034316	-1.261759	0.584352
4	7	0	-1.373237	-0.105821	0.562005
5	6	0	-1.901059	1.066978	0.171963
6	6	0	-3.226749	1.108323	-0.194592
7	6	0	-0.964299	2.242536	0.115810
8	7	0	-0.086392	2.319741	1.280946
9	6	0	1.319872	2.506581	0.964318
10	6	0	2.019506	1.231310	0.559999
11	7	0	1.332585	0.096690	0.566986
12	6	0	1.905916	-1.060444	0.232178
13	6	0	3.242488	-1.129212	-0.122768
14	6	0	3.964890	0.054717	-0.134744
15	6	0	3.363798	1.252356	0.200053
16	6	0	1.001250	-2.269929	0.218786
17	7	0	0.072204	-2.284728	1.350179
18	6	0	-1.316952	-2.511484	1.014535
19	1	0	-3.922103	-2.198766	0.221807
20	1	0	-3.674133	2.044534	-0.497486
21	1	0	-1.561559	3.145125	-0.012438
22	1	0	-0.366434	2.127424	-0.793652
23	1	0	1.821520	2.889870	1.854336
24	1	0	1.483645	3.246149	0.170675
25	1	0	3.696628	-2.076235	-0.378231
26	1	0	3.916365	2.183976	0.193988
27	1	0	0.432419	-2.239932	-0.715929
28	1	0	1.610928	-3.173249	0.185807
29	1	0	-1.838477	-2.889107	1.895555
30	1	0	-1.463560	-3.254482	0.220797
31	1	0	-0.329575	-0.080984	0.787812
32	1	0	0.365153	-2.977426	2.024389
33	1	0	-0.388064	3.084750	1.868784
34	6	0	-5.421109	-0.050694	-0.518944
35	9	0	-5.704023	0.906391	-1.403595
36	9	0	-6.165834	0.183596	0.568598
37	9	0	-5.831732	-1.209397	-1.035363
38	6	0	5.421601	0.066485	-0.512103
39	9	0	5.639667	0.815982	-1.601541
40	9	0	5.894703	-1.153238	-0.773874
41	9	0	6.178964	0.582605	0.465113

Most stable energy, Gibbs free energy (Ha), and geometry for protomer ./CF3_Py2N2//1_1
E: -1436.736095
G: -1436.478458
Geometry:

Input orientation:

Center Atomic Atomic Coordinates (Angstroms)

Number	Number	Type	X	Y	Z
1	6	0	-3.972425	-0.136138	0.113250
2	6	0	-3.358057	-1.304220	-0.277856
3	6	0	-2.003854	-1.247769	-0.615925
4	7	0	-1.329770	-0.112223	-0.549397
5	6	0	-1.920080	1.022824	-0.156616
6	6	0	-3.257703	1.056761	0.179701
7	6	0	-1.020909	2.232260	-0.067815
8	7	0	-0.119669	2.335256	-1.217721
9	6	0	1.272444	2.564252	-0.897587
10	6	0	2.014490	1.304075	-0.549553
11	7	0	1.371517	0.139415	-0.575188
12	6	0	1.923466	-1.044859	-0.253453
13	6	0	3.253112	-1.081958	0.091330
14	6	0	3.962719	0.115742	0.111302
15	6	0	3.358439	1.314151	-0.196111
16	6	0	1.006038	-2.237005	-0.245474
17	7	0	0.112305	-2.270414	-1.400681
18	6	0	-1.286966	-2.493491	-1.076713
19	1	0	-3.895030	-2.241312	-0.335305
20	1	0	-3.730874	1.982071	0.482569
21	1	0	-0.428291	2.130532	0.846843
22	1	0	-1.631109	3.128070	0.050752
23	1	0	1.425624	3.264675	-0.067011
24	1	0	1.770801	2.997568	-1.766301
25	1	0	3.722703	-2.024068	0.340927
26	1	0	3.895437	2.251849	-0.174575
27	1	0	1.620385	-3.134389	-0.173670
28	1	0	0.420289	-2.180024	0.677105
29	1	0	-1.429240	-3.265478	-0.309893
30	1	0	-1.792891	-2.851818	-1.974616
31	1	0	0.324242	0.107725	-0.782667
32	1	0	0.417094	-3.001090	-2.029147
33	1	0	-0.439302	3.064362	-1.839517
34	6	0	-5.432438	-0.112335	0.474930
35	9	0	-5.617190	0.296203	1.737934
36	9	0	-6.011887	-1.308365	0.359816
37	9	0	-6.117023	0.734926	-0.305433
38	6	0	5.421928	0.067857	0.493293
39	9	0	6.009860	1.259654	0.408359
40	9	0	6.099868	-0.771365	-0.295874
41	9	0	5.574552	-0.365879	1.749192

Most stable energy, Gibbs free energy (Ha), and geometry for protomer ./CF3_Py2N2//1_4
E: -1436.743004
G: -1436.481332
Geometry:

Input orientation:

Center Number	Atomic Number	Atomic Type	Coordinates (Angstroms) X	Y	Z
1	6	0	0.289207	1.845166	-0.002512
2	6	0	-0.516706	1.861994	-1.136719
3	6	0	-1.863671	1.625222	-0.961547
4	7	0	-2.422103	1.422917	0.237227
5	6	0	-1.643334	1.365558	1.308244
6	6	0	-0.263115	1.583493	1.229547

7	6	0	-2.264781	0.947506	2.621551
8	7	0	-1.814269	-0.343447	3.143075
9	6	0	-2.129603	-1.512603	2.317866
10	6	0	-1.331123	-1.634027	1.039340
11	7	0	-1.957281	-1.412004	-0.117477
12	6	0	-1.253448	-1.346082	-1.240901
13	6	0	0.115739	-1.566561	-1.290205
14	6	0	0.750301	-1.862743	-0.097442
15	6	0	0.033585	-1.894366	1.085705
16	6	0	-2.020298	-0.991594	-2.491062
17	7	0	-3.142861	-0.052390	-2.186798
18	6	0	-2.772248	1.402441	-2.134238
19	1	0	-0.100706	2.003377	-2.126383
20	1	0	0.348166	1.518760	2.120777
21	1	0	-3.345826	0.910216	2.492349
22	1	0	-2.039349	1.701876	3.376274
23	1	0	-3.189586	-1.481178	2.067966
24	1	0	-1.950711	-2.401860	2.923423
25	1	0	0.656906	-1.481973	-2.222473
26	1	0	0.523135	-2.084633	2.033678
27	1	0	-1.378551	-0.523130	-3.233731
28	1	0	-2.466432	-1.886713	-2.922903
29	1	0	-3.539389	-0.323247	-1.276235
30	1	0	-3.702913	1.954891	-2.025825
31	1	0	-2.295005	1.648795	-3.078796
32	1	0	-3.874215	-0.172391	-2.890536
33	1	0	-0.814545	-0.312058	3.322151
34	6	0	1.770714	2.038446	-0.171965
35	9	0	2.436970	1.941282	0.978532
36	9	0	2.056033	3.236817	-0.697554
37	9	0	2.284172	1.121913	-1.006300
38	6	0	2.234904	-2.097980	-0.045041
39	9	0	2.522568	-3.326144	0.406797
40	9	0	2.835539	-1.233892	0.785152
41	9	0	2.818760	-1.970010	-1.237651

Most stable energy, Gibbs free energy (Ha), and geometry for protomer ./CF3_Py2N2//2_1

E: -1437.179269

G: -1436.905932

Geometry:

Input orientation:

Center Number	Atomic Number	Atomic Type	Coordinates (Angstroms) X	Y	Z
1	6	0	-0.097241	2.081156	0.022811
2	6	0	0.515961	1.834543	-1.193959
3	6	0	1.814352	1.363996	-1.192069
4	7	0	2.428120	1.207037	-0.012000
5	6	0	1.854062	1.386292	1.187282
6	6	0	0.558911	1.856835	1.224370
7	6	0	2.627580	0.907266	2.380948
8	7	0	2.958009	-0.507105	2.201239
9	6	0	1.836185	-1.407755	2.432190
10	6	0	1.009232	-1.611147	1.190274
11	7	0	1.640140	-1.581516	0.009496
12	6	0	1.043654	-1.656602	-1.190260
13	6	0	-0.320412	-1.852456	-1.224157
14	6	0	-1.008158	-1.929570	-0.022000

15	6	0	-0.359134	-1.800935	1.193435
16	6	0	1.905710	-1.486090	-2.412434
17	7	0	2.967992	-0.511030	-2.198726
18	6	0	2.553098	0.876244	-2.405565
19	1	0	-0.005541	1.949738	-2.133782
20	1	0	0.067133	1.988587	2.178535
21	1	0	2.030624	1.098419	3.275912
22	1	0	3.557730	1.471833	2.453636
23	1	0	1.164671	-1.065011	3.225060
24	1	0	2.226697	-2.383401	2.728170
25	1	0	-0.827091	-1.902747	-2.177812
26	1	0	-0.890658	-1.811123	2.134199
27	1	0	2.363113	-2.452687	-2.632522
28	1	0	1.247921	-1.230168	-3.248095
29	1	0	1.905723	1.006492	-3.276090
30	1	0	3.446337	1.486820	-2.543139
31	1	0	3.371696	0.811683	-0.025185
32	1	0	2.649356	-1.407712	0.025865
33	1	0	3.738831	-0.719946	-2.819765
34	1	0	3.715241	-0.749157	2.827029
35	6	0	-1.557292	2.470786	0.057409
36	9	0	-1.816988	3.321195	1.048909
37	9	0	-2.316072	1.384940	0.251313
38	9	0	-1.952437	3.037073	-1.079417
39	6	0	-2.506760	-2.131205	-0.053758
40	9	0	-3.068329	-1.459078	-1.059290
41	9	0	-3.087492	-1.727461	1.074456
42	9	0	-2.807396	-3.422467	-0.220721

Most stable energy, Gibbs free energy (Ha), and geometry for protomer ./CF3_Py2N2//2_3
E: -1437.182653
G: -1436.907021
Geometry:
Input orientation:

Center Number	Atomic Number	Atomic Type	Coordinates (Angstroms) X	Y	Z
1	6	0	-1.886555	-0.767924	-0.138752
2	6	0	-1.461580	-0.158335	-1.297553
3	6	0	-1.245995	1.214156	-1.281135
4	7	0	-1.489118	1.876029	-0.153001
5	6	0	-1.843769	1.306720	1.014753
6	6	0	-2.079955	-0.043466	1.037861
7	6	0	-1.849710	2.170536	2.237445
8	7	0	-0.462040	2.598522	2.621488
9	6	0	0.535744	1.499325	2.797048
10	6	0	1.121465	1.101085	1.464117
11	7	0	1.233310	2.056655	0.555204
12	6	0	1.646708	1.747357	-0.678968
13	6	0	2.040424	0.461705	-1.010788
14	6	0	1.944046	-0.526906	-0.040735
15	6	0	1.464043	-0.224653	1.216066
16	6	0	1.515788	2.816179	-1.731211
17	7	0	0.094738	3.137873	-1.942278
18	6	0	-0.684273	2.008763	-2.429943
19	1	0	-1.251033	-0.715874	-2.199671
20	1	0	-2.352315	-0.527727	1.966412
21	1	0	-2.257250	1.614528	3.076494

22	1	0	-2.428046	3.078589	2.075758
23	1	0	0.037960	0.661468	3.281270
24	1	0	1.313926	1.881874	3.456269
25	1	0	2.359554	0.229528	-2.018946
26	1	0	1.322581	-0.982266	1.974890
27	1	0	2.019683	3.724242	-1.401644
28	1	0	1.990915	2.464794	-2.651391
29	1	0	-0.117422	1.318766	-3.063757
30	1	0	-1.526241	2.377709	-3.019463
31	1	0	-0.544575	3.114639	3.502076
32	1	0	-1.241188	2.872320	-0.179049
33	1	0	0.030108	3.896599	-2.609126
34	1	0	-0.087718	3.256518	1.924928
35	6	0	-2.039882	-2.270444	-0.076128
36	9	0	-1.086639	-2.799220	0.698816
37	9	0	-1.946772	-2.838760	-1.274315
38	9	0	-3.216223	-2.614456	0.448871
39	6	0	2.250802	-1.948658	-0.426244
40	9	0	2.250959	-2.775396	0.619409
41	9	0	3.442933	-2.055376	-1.022111
42	9	0	1.339590	-2.410794	-1.295955

Most stable energy, Gibbs free energy (Ha), and geometry for protomer ./CF3_Py2N2//2_6
E: -1437.198032
G: -1436.921467
Geometry:
Input orientation:

Center Number	Atomic Number	Atomic Type	Coordinates (Angstroms) X	Y	Z
1	6	0	0.593474	1.879004	-0.259087
2	6	0	0.034539	1.373094	-1.414542
3	6	0	-1.337940	1.139381	-1.416215
4	7	0	-2.105485	1.388288	-0.367376
5	6	0	-1.543867	1.820353	0.762015
6	6	0	-0.194841	2.102848	0.861884
7	6	0	-2.450231	1.864549	1.957674
8	7	0	-3.016080	0.498200	2.208147
9	6	0	-2.023591	-0.543762	2.619112
10	6	0	-1.339220	-1.140249	1.416169
11	7	0	-2.106488	-1.389621	0.367219
12	6	0	-1.544417	-1.821164	-0.762127
13	6	0	-0.195169	-2.102734	-0.861856
14	6	0	0.592867	-1.878378	0.259197
15	6	0	0.033418	-1.372941	1.414627
16	6	0	-2.450472	-1.865225	-1.958002
17	7	0	-3.015209	-0.498463	-2.208944
18	6	0	-2.021637	0.542616	-2.619428
19	1	0	0.632749	1.133606	-2.283022
20	1	0	0.232491	2.439666	1.797734
21	1	0	-3.298825	2.523273	1.783131
22	1	0	-1.921282	2.169059	2.856648
23	1	0	-2.577790	-1.314101	3.153825
24	1	0	-1.310126	-0.080163	3.295946
25	1	0	0.232502	-2.439156	-1.797697
26	1	0	0.631366	-1.133022	2.283166
27	1	0	-1.921508	-2.170331	-2.856760
28	1	0	-3.299595	-2.523253	-1.783473

29	1	0	-3.491146	-0.172178	-1.357160
30	1	0	-2.574726	1.313071	-3.155129
31	1	0	-1.307820	0.078232	-3.295359
32	1	0	-3.491815	0.172368	1.356076
33	1	0	-3.726090	-0.579685	-2.939510
34	1	0	-3.727312	0.579886	2.938323
35	6	0	2.077984	2.099402	-0.145406
36	9	0	2.721800	1.858315	-1.286713
37	9	0	2.608093	1.297009	0.788339
38	9	0	2.361865	3.355497	0.216927
39	6	0	2.077567	-2.097577	0.145639
40	9	0	2.721126	-1.855638	1.286913
41	9	0	2.607044	-1.294985	-0.788292
42	9	0	2.362529	-3.353524	-0.216350

Most stable energy, Gibbs free energy (Ha), and geometry for protomer ./CF3_Py2N2//2_2
E: -1437.182481
G: -1436.905873
Geometry:
Input orientation:

Center Number	Atomic Number	Atomic Type	Coordinates (Angstroms) X	Y	Z
1	6	0	-1.909090	-0.545139	-0.081835
2	6	0	-1.959815	0.338988	-1.150784
3	6	0	-1.606770	1.659797	-0.928420
4	7	0	-1.276662	2.099709	0.290558
5	6	0	-1.209923	1.243167	1.297539
6	6	0	-1.515611	-0.107725	1.165753
7	6	0	-0.711504	1.790269	2.611914
8	7	0	0.318585	2.847776	2.377649
9	6	0	1.717990	2.363754	2.126080
10	6	0	1.776405	1.399720	0.981613
11	7	0	1.492253	1.863827	-0.251217
12	6	0	1.322022	1.103607	-1.330044
13	6	0	1.548744	-0.262634	-1.215805
14	6	0	1.899123	-0.766429	0.015889
15	6	0	2.012014	0.058257	1.135720
16	6	0	0.821499	1.789385	-2.573867
17	7	0	0.003703	2.944322	-2.231427
18	6	0	-1.423447	2.619572	-2.074225
19	1	0	-2.211184	0.001109	-2.148141
20	1	0	-1.410463	-0.782057	2.004778
21	1	0	-0.265738	1.011863	3.227443
22	1	0	-1.524625	2.260496	3.163238
23	1	0	2.058925	1.873867	3.033649
24	1	0	2.325727	3.245283	1.928774
25	1	0	1.399838	-0.897691	-2.077921
26	1	0	2.224650	-0.341794	2.118252
27	1	0	1.693273	2.118650	-3.143106
28	1	0	0.298321	1.040573	-3.177539
29	1	0	-1.836549	2.169232	-2.981164
30	1	0	-1.957386	3.546772	-1.868741
31	1	0	0.359841	3.456629	3.200094
32	1	0	1.239770	2.851139	-0.381820
33	1	0	0.098362	3.642614	-2.958032
34	1	0	-0.001550	3.431835	1.593336
35	6	0	-2.164893	-2.005473	-0.340608

36	9	0	-2.221392	-2.722406	0.781739
37	9	0	-1.188914	-2.530346	-1.097161
38	9	0	-3.311893	-2.200803	-0.999191
39	6	0	2.059799	-2.255889	0.216891
40	9	0	3.233652	-2.542904	0.781219
41	9	0	1.980117	-2.932021	-0.924554
42	9	0	1.103069	-2.716393	1.029657

Most stable energy, Gibbs free energy (Ha), and geometry for protomer ./CF3_Py2N2//2_5

E: -1437.182658

G: -1436.906934

Geometry:

Input orientation:

Center Number	Atomic Number	Atomic Type	Coordinates (Angstroms) X	Y	Z
1	6	0	-1.897527	-0.748954	-0.141952
2	6	0	-2.088640	-0.031811	1.039561
3	6	0	-1.847724	1.317678	1.025636
4	7	0	-1.491839	1.893626	-0.138516
5	6	0	-1.251951	1.238799	-1.271406
6	6	0	-1.471533	-0.132945	-1.296918
7	6	0	-0.689700	2.039521	-2.415732
8	7	0	0.092915	3.163385	-1.921920
9	6	0	1.513423	2.836635	-1.715062
10	6	0	1.643295	1.761151	-0.669489
11	7	0	1.231211	2.063606	0.566835
12	6	0	1.118793	1.102835	1.470121
13	6	0	1.458798	-0.222007	1.213776
14	6	0	1.937485	-0.517337	-0.045127
15	6	0	2.035026	0.476985	-1.009209
16	6	0	0.535208	1.494021	2.806020
17	7	0	-0.460691	2.596274	2.638351
18	6	0	-1.849852	2.173527	2.254138
19	1	0	-2.362558	-0.521611	1.964769
20	1	0	-1.263232	-0.685112	-2.202855
21	1	0	-1.531547	2.414362	-3.001675
22	1	0	-0.125528	1.352225	-3.054867
23	1	0	2.020236	3.741341	-1.380732
24	1	0	1.986223	2.489626	-2.638100
25	1	0	1.316577	-0.984082	1.967976
26	1	0	2.353345	0.250397	-2.018898
27	1	0	0.036428	0.654078	3.285573
28	1	0	1.314812	1.871019	3.466715
29	1	0	-0.085859	3.257353	1.944939
30	1	0	-2.426891	3.083604	2.099404
31	1	0	-2.256784	1.612413	3.090156
32	1	0	-1.240304	2.889200	-0.157718
33	1	0	-0.540597	3.107489	3.522033
34	1	0	0.029275	3.926661	-2.583646
35	6	0	-2.055752	-2.251324	-0.089261
36	9	0	-1.108246	-2.787877	0.687391
37	9	0	-1.958272	-2.812648	-1.290366
38	9	0	-3.235935	-2.594652	0.427513
39	6	0	2.242065	-1.936963	-0.439905
40	9	0	3.436005	-2.042089	-1.032492
41	9	0	1.332764	-2.390343	-1.316140
42	9	0	2.236646	-2.771443	0.599533

Most stable energy, Gibbs free energy (Ha), and geometry for protomer ./CF3_Py2N2//2_4
E: -1437.182649
G: -1436.906048
Geometry:
Input orientation:

Center Number	Atomic Number	Atomic Type	Coordinates (Angstroms) X	Y	Z
1	6	0	1.937481	-0.449866	0.144291
2	6	0	1.366748	-0.056781	1.336265
3	6	0	0.995805	1.279407	1.453149
4	7	0	1.163348	2.162174	0.481700
5	6	0	1.667865	1.763675	-0.691761
6	6	0	2.095601	0.461810	-0.891267
7	6	0	1.611562	2.748013	-1.830297
8	7	0	0.210546	3.051488	-2.165879
9	6	0	-0.535915	1.888264	-2.624113
10	6	0	-1.179690	1.183202	-1.460274
11	7	0	-1.493791	1.932287	-0.406849
12	6	0	-1.929421	1.456282	0.775377
13	6	0	-2.167979	0.111296	0.890001
14	6	0	-1.896882	-0.704234	-0.209554
15	6	0	-1.398881	-0.186875	-1.383692
16	6	0	-2.017995	2.411886	1.924216
17	7	0	-0.657836	2.858575	2.380119
18	6	0	0.313285	1.770962	2.706473
19	1	0	1.178287	-0.753706	2.141564
20	1	0	2.489880	0.156655	-1.852292
21	1	0	2.148575	2.328020	-2.685353
22	1	0	2.094891	3.679592	-1.537470
23	1	0	0.073259	1.154030	-3.161538
24	1	0	-1.332117	2.210629	-3.298196
25	1	0	-2.502309	-0.298476	1.833735
26	1	0	-1.131697	-0.813601	-2.223192
27	1	0	-2.570224	3.309180	1.650066
28	1	0	-2.495599	1.924637	2.769003
29	1	0	-0.799352	3.439018	3.211567
30	1	0	-0.226495	0.969855	3.207140
31	1	0	1.041686	2.195525	3.396196
32	1	0	-1.231786	2.921784	-0.495175
33	1	0	-0.227423	3.460101	1.664354
34	1	0	0.197683	3.758510	-2.890008
35	6	0	2.285424	-1.893050	-0.103854
36	9	0	3.533634	-2.031167	-0.562935
37	9	0	1.471098	-2.426768	-1.026796
38	9	0	2.178632	-2.641493	0.994057
39	6	0	-2.047733	-2.197932	-0.035274
40	9	0	-3.258289	-2.511679	0.427088
41	9	0	-1.152655	-2.649689	0.849856
42	9	0	-1.860278	-2.861722	-1.171486

Most stable energy, Gibbs free energy (Ha), and geometry for protomer ./NMe2_Py2N2//0_1
E: -1030.040193
G: -1029.652568
Geometry:
Input orientation:

Center Atomic Atomic Coordinates (Angstroms)

Number	Number	Type	X	Y	Z
1	6	0	0.830177	-1.651477	-1.040851
2	6	0	-0.022481	-1.172250	-2.051713
3	6	0	-1.378432	-1.070875	-1.807528
4	7	0	-1.961754	-1.492937	-0.681307
5	6	0	-1.158072	-1.890058	0.307082
6	6	0	0.216614	-1.988142	0.179541
7	6	0	-1.834509	-2.145097	1.638143
8	7	0	-2.697013	-1.053381	2.106069
9	6	0	-1.976690	0.096075	2.661671
10	6	0	-1.065847	0.817251	1.691216
11	7	0	-1.621254	1.163461	0.524230
12	6	0	-0.812473	1.603287	-0.436983
13	6	0	0.544241	1.825084	-0.245951
14	6	0	1.115546	1.591619	1.017745
15	6	0	0.263475	1.033425	1.993162
16	6	0	-1.451732	1.881485	-1.789005
17	7	0	-2.604001	1.048898	-2.145055
18	6	0	-2.250272	-0.256169	-2.727886
19	1	0	0.367282	-0.792356	-2.984557
20	1	0	0.799958	-2.278821	1.041198
21	1	0	-1.082879	-2.348616	2.401691
22	1	0	-2.450505	-3.042283	1.540442
23	1	0	-1.395794	-0.239588	3.521440
24	1	0	-2.724184	0.802809	3.029077
25	1	0	1.140914	2.162484	-1.081800
26	1	0	0.639242	0.721067	2.956432
27	1	0	-1.773254	2.926997	-1.783949
28	1	0	-0.693323	1.793541	-2.568732
29	1	0	-1.739535	-0.085783	-3.675860
30	1	0	-3.179515	-0.788800	-2.931745
31	1	0	-3.103573	0.860011	-1.279149
32	1	0	-3.217643	-0.722664	1.297390
33	7	0	2.430570	1.860870	1.282967
34	7	0	2.186936	-1.717654	-1.213312
35	6	0	3.016747	-1.760305	-0.017557
36	1	0	2.852527	-2.682442	0.539419
37	1	0	4.061587	-1.730201	-0.314199
38	1	0	2.813450	-0.913447	0.648083
39	6	0	3.006486	1.365350	2.522766
40	1	0	4.039941	1.695943	2.582934
41	1	0	2.473698	1.771098	3.382627
42	1	0	2.983764	0.272029	2.589018
43	6	0	2.757134	-1.049460	-2.373915
44	1	0	2.386326	-1.496931	-3.295721
45	1	0	2.522913	0.021124	-2.392138
46	1	0	3.836589	-1.172216	-2.352655
47	6	0	3.339901	2.043339	0.162886
48	1	0	4.337269	2.233104	0.550151
49	1	0	3.377144	1.159697	-0.484310
50	1	0	3.047674	2.901924	-0.441655

Most stable energy, Gibbs free energy (Ha), and geometry for protomer ./NMe2_Py2N2//1_3
E: -1030.504345
G: -1030.101374
Geometry:
Input orientation:

Center Number	Atomic Number	Atomic Type	Coordinates (Angstroms) X	Y	Z
1	6	0	1.623433	1.434264	-0.347907
2	6	0	0.857367	1.978705	0.705537
3	6	0	-0.516968	2.026720	0.602702
4	7	0	-1.180821	1.630062	-0.491430
5	6	0	-0.474424	1.044992	-1.452432
6	6	0	0.900440	0.907107	-1.436220
7	6	0	-1.273657	0.510013	-2.620454
8	7	0	-2.634968	0.073586	-2.177241
9	6	0	-2.749095	-1.366334	-1.757497
10	6	0	-1.892712	-1.543326	-0.538128
11	7	0	-2.316576	-0.873263	0.534698
12	6	0	-1.438728	-0.710951	1.527775
13	6	0	-0.177459	-1.278619	1.523082
14	6	0	0.239549	-2.063007	0.430662
15	6	0	-0.674537	-2.177614	-0.636869
16	6	0	-1.874479	0.218460	2.641116
17	7	0	-2.397393	1.512241	2.190886
18	6	0	-1.371765	2.469369	1.769355
19	1	0	1.322199	2.310043	1.622415
20	1	0	1.389413	0.387295	-2.247405
21	1	0	-0.777999	-0.334012	-3.094735
22	1	0	-1.421234	1.293683	-3.362345
23	1	0	-2.423284	-1.976856	-2.595092
24	1	0	-3.801447	-1.542969	-1.546287
25	1	0	0.490725	-1.064735	2.344546
26	1	0	-0.411820	-2.669231	-1.561720
27	1	0	-2.658944	-0.284029	3.211900
28	1	0	-1.040580	0.395082	3.321157
29	1	0	-0.727825	2.687603	2.621646
30	1	0	-1.880924	3.396123	1.496900
31	1	0	-3.310784	0.254551	-2.920607
32	1	0	-3.001706	1.332365	1.393331
33	1	0	-2.878242	0.664112	-1.366274
34	7	0	1.482561	-2.615819	0.370127
35	7	0	2.982865	1.388086	-0.297578
36	6	0	3.685260	0.497020	-1.208043
37	1	0	3.382071	-0.546905	-1.071600
38	1	0	3.503077	0.776874	-2.245689
39	1	0	4.752659	0.576312	-1.022776
40	6	0	1.939560	-3.148572	-0.904941
41	1	0	2.949646	-3.528950	-0.782637
42	1	0	1.304819	-3.974280	-1.226375
43	1	0	1.941019	-2.386459	-1.691964
44	6	0	3.654602	1.747015	0.941381
45	1	0	3.405863	2.767926	1.231043
46	1	0	3.389318	1.078994	1.767501
47	1	0	4.728095	1.696392	0.782775
48	6	0	2.498555	-2.118855	1.287154
49	1	0	3.444622	-2.600186	1.056640
50	1	0	2.623456	-1.033551	1.201651
51	1	0	2.241819	-2.355499	2.319852

Most stable energy, Gibbs free energy (Ha), and geometry for protomer ./NMe2_Py2N2//1_2
E: -1030.504116
G: -1030.108446
Geometry:

Input orientation:

Center Number	Atomic Number	Atomic Type	Coordinates (Angstroms) X	Y	Z
1	6	0	-3.974082	-0.026048	0.320432
2	6	0	-3.163609	-1.196699	0.295577
3	6	0	-1.859190	-1.107405	-0.089667
4	7	0	-1.343778	0.079188	-0.470473
5	6	0	-2.051478	1.216409	-0.450451
6	6	0	-3.364180	1.195839	-0.057150
7	6	0	-1.371291	2.491418	-0.916033
8	7	0	0.052580	2.438298	-1.178156
9	6	0	0.919702	2.270195	-0.005361
10	6	0	1.871596	1.102562	-0.121784
11	7	0	1.323213	-0.040750	-0.554220
12	6	0	2.055769	-1.150993	-0.561431
13	6	0	3.384242	-1.161035	-0.177915
14	6	0	4.005697	0.040430	0.214336
15	6	0	3.189695	1.197521	0.254557
16	6	0	1.387899	-2.438718	-0.998710
17	7	0	-0.037567	-2.303448	-1.265676
18	6	0	-0.885229	-2.258790	-0.071918
19	1	0	-3.555601	-2.160047	0.583443
20	1	0	-3.914276	2.124887	-0.053577
21	1	0	-1.572113	3.265244	-0.174170
22	1	0	-1.883258	2.801607	-1.828848
23	1	0	0.306953	2.099640	0.887127
24	1	0	1.495692	3.177948	0.178936
25	1	0	3.923769	-2.096896	-0.193609
26	1	0	3.575921	2.149764	0.585451
27	1	0	1.869499	-2.771718	-1.919605
28	1	0	1.604276	-3.201229	-0.239934
29	1	0	-0.258432	-2.134813	0.816413
30	1	0	-1.447342	-3.182919	0.064359
31	1	0	-0.299781	0.068682	-0.684083
32	1	0	-0.326373	-3.082886	-1.840873
33	1	0	0.258067	1.734718	-1.877524
34	7	0	5.321657	0.091336	0.540653
35	7	0	-5.260388	-0.081533	0.687489
36	6	0	-6.066647	1.130309	0.706812
37	1	0	-5.652566	1.866098	1.398959
38	1	0	-6.122345	1.580277	-0.286369
39	1	0	-7.070873	0.877699	1.030094
40	6	0	5.859988	1.294670	1.154192
41	1	0	6.925306	1.158095	1.315204
42	1	0	5.726530	2.156829	0.500761
43	1	0	5.384406	1.511437	2.115831
44	6	0	-5.863303	-1.351539	1.067354
45	1	0	-5.365514	-1.778815	1.939896
46	1	0	-6.907092	-1.183830	1.311737
47	1	0	-5.809853	-2.071572	0.248684
48	6	0	6.059378	-1.149835	0.707348
49	1	0	6.051939	-1.733229	-0.213549
50	1	0	7.091822	-0.913978	0.947124
51	1	0	5.644043	-1.766501	1.510705

Most stable energy, Gibbs free energy (Ha), and geometry for protomer ./NMe2_Py2N2//1_1

E: -1030.504116

G: -1030.108451
Geometry:

Input orientation:

Center Number	Atomic Number	Atomic Type	Coordinates (Angstroms) X	Y	Z
1	6	0	-4.005554	-0.039691	0.227401
2	6	0	-3.189513	-1.196734	0.268373
3	6	0	-1.871502	-1.102030	-0.108333
4	7	0	-1.323244	0.040958	-0.541792
5	6	0	-2.055832	1.151154	-0.549763
6	6	0	-3.384248	1.161466	-0.165999
7	6	0	-1.388067	2.438566	-0.988179
8	7	0	0.037235	2.302933	-1.255869
9	6	0	0.885540	2.259050	-0.062509
10	6	0	1.859547	1.107708	-0.080129
11	7	0	1.343840	-0.079176	-0.459645
12	6	0	2.051476	-1.216437	-0.439157
13	6	0	3.364437	-1.195598	-0.046752
14	6	0	3.974693	0.026600	0.329274
15	6	0	3.164237	1.197261	0.304117
16	6	0	1.370885	-2.491800	-0.903196
17	7	0	-0.053145	-2.438823	-1.164468
18	6	0	-0.919540	-2.269541	0.008704
19	1	0	-3.575651	-2.148736	0.600054
20	1	0	-3.923836	2.097278	-0.182435
21	1	0	-1.603887	3.201529	-0.229705
22	1	0	-1.870160	2.771109	-1.908979
23	1	0	0.259189	2.135430	0.826176
24	1	0	1.447615	3.183313	0.072988
25	1	0	3.914457	-2.124690	-0.042629
26	1	0	3.556453	2.160792	0.591063
27	1	0	1.882259	-2.802804	-1.816065
28	1	0	1.572141	-3.264989	-0.160788
29	1	0	-0.306237	-2.098061	0.900633
30	1	0	-1.495409	-3.177111	0.194285
31	1	0	0.299778	-0.068786	-0.672591
32	1	0	-0.259028	-1.735943	-1.864422
33	1	0	0.325842	3.081915	-1.831783
34	7	0	5.261256	0.082331	0.695379
35	7	0	-5.321442	-0.090362	0.554105
36	6	0	-5.859654	-1.293216	1.168644
37	1	0	-6.924933	-1.156507	1.329811
38	1	0	-5.726393	-2.155887	0.515844
39	1	0	-5.383866	-1.509311	2.130337
40	6	0	5.864165	1.352435	1.074935
41	1	0	5.367231	1.779358	1.948157
42	1	0	6.908317	1.185046	1.317982
43	1	0	5.809527	2.072633	0.256508
44	6	0	-6.059170	1.150907	0.719846
45	1	0	-5.643724	1.768306	1.522585
46	1	0	-6.051933	1.733517	-0.201554
47	1	0	-7.091570	0.915238	0.960012
48	6	0	6.067101	-1.129761	0.716469
49	1	0	7.071746	-0.876833	1.038189
50	1	0	5.653432	-1.864013	1.410514
51	1	0	6.121680	-1.581755	-0.275837

Most stable energy, Gibbs free energy (Ha), and geometry for protomer ./NMe2_Py2N2//1_4
E: -1030.504346
G: -1030.101345
Geometry:

Input orientation:

Center Number	Atomic Number	Atomic Type	Coordinates (Angstroms) X	Y	Z
1	6	0	0.236761	-2.063538	0.429970
2	6	0	-0.677256	-2.176343	-0.637842
3	6	0	-1.894454	-1.540215	-0.539164
4	7	0	-2.317534	-0.869802	0.533769
5	6	0	-1.439670	-0.709192	1.527093
6	6	0	-0.179331	-1.278913	1.522567
7	6	0	-1.874201	0.220410	2.640753
8	7	0	-2.395430	1.515043	2.191022
9	6	0	-1.368518	2.471040	1.769974
10	6	0	-0.514171	2.027602	0.603309
11	7	0	-1.178489	1.632609	-0.491123
12	6	0	-0.472881	1.046672	-1.452205
13	6	0	0.901730	0.906495	-1.435779
14	6	0	1.625440	1.432005	-0.347127
15	6	0	0.860066	1.977137	0.706441
16	6	0	-1.272835	0.513563	-2.620570
17	7	0	-2.634648	0.078387	-2.177588
18	6	0	-2.750406	-1.361588	-1.758597
19	1	0	-0.415008	-2.667945	-1.562827
20	1	0	0.488956	-1.066330	2.344282
21	1	0	-1.040039	0.395682	3.320830
22	1	0	-2.659294	-0.281232	3.211427
23	1	0	-0.724483	2.688231	2.622462
24	1	0	-1.876509	3.398469	1.497653
25	1	0	1.389974	0.386174	-2.247071
26	1	0	1.325209	2.307031	1.623680
27	1	0	-1.419594	1.298026	-3.361799
28	1	0	-0.778248	-0.330668	-3.095592
29	1	0	-3.310192	0.260459	-2.920944
30	1	0	-2.425183	-1.972066	-2.596456
31	1	0	-3.802981	-1.537193	-1.547631
32	1	0	-2.877366	0.668786	-1.366368
33	1	0	-2.999872	1.336303	1.393306
34	7	0	2.984808	1.383618	-0.296659
35	7	0	1.478910	-2.618148	0.369560
36	6	0	2.495251	-2.123581	1.287441
37	1	0	2.622116	-1.038440	1.202687
38	1	0	2.237505	-2.360424	2.319848
39	1	0	3.440623	-2.606395	1.057171
40	6	0	3.657002	1.740964	0.942499
41	1	0	3.408529	2.761576	1.233359
42	1	0	3.391901	1.072029	1.767939
43	1	0	4.730449	1.690434	0.783479
44	6	0	1.935502	-3.151607	-0.905354
45	1	0	1.938523	-2.389430	-1.692315
46	1	0	2.944940	-3.533617	-0.782739
47	1	0	1.299550	-3.976270	-1.227079
48	6	0	3.685936	0.491512	-1.207079
49	1	0	3.504299	0.771669	-2.244733

50	1	0	4.753441	0.569214	-1.021704
51	1	0	3.381207	-0.551966	-1.070702

Most stable energy, Gibbs free energy (Ha), and geometry for protomer ./NMe2_Py2N2//2_1
E: -1030.973795
G: -1030.560243
Geometry:
Input orientation:

Center Number	Atomic Number	Atomic Type	Coordinates (Angstroms) X	Y	Z
1	6	0	-0.891913	-2.048452	-0.213898
2	6	0	-0.147462	-2.030570	1.000627
3	6	0	1.188201	-1.770867	0.980230
4	7	0	1.815521	-1.566681	-0.198550
5	6	0	1.147471	-1.464968	-1.367537
6	6	0	-0.189790	-1.715880	-1.407577
7	6	0	1.946544	-0.982435	-2.545780
8	7	0	2.633645	0.267149	-2.219142
9	6	0	1.766590	1.440529	-2.260311
10	6	0	0.942998	1.584326	-1.007339
11	7	0	1.557207	1.302157	0.161628
12	6	0	0.902675	1.274132	1.341024
13	6	0	-0.406146	1.648922	1.397966
14	6	0	-1.087221	2.047238	0.214576
15	6	0	-0.364118	1.967730	-1.008599
16	6	0	1.686666	0.814496	2.543520
17	7	0	2.627059	-0.252723	2.218245
18	6	0	2.023607	-1.586343	2.214677
19	1	0	-0.627239	-2.165998	1.957407
20	1	0	-0.701717	-1.602125	-2.350446
21	1	0	1.271961	-0.884668	-3.401263
22	1	0	2.702344	-1.730786	-2.788649
23	1	0	1.080425	1.431868	-3.112959
24	1	0	2.400145	2.324841	-2.350031
25	1	0	-0.911295	1.597057	2.349955
26	1	0	-0.837269	2.169371	-1.957294
27	1	0	2.257799	1.667318	2.915075
28	1	0	0.970631	0.528961	3.320759
29	1	0	1.390955	-1.766956	3.088346
30	1	0	2.829097	-2.322228	2.213300
31	1	0	2.796240	-1.300792	-0.183614
32	1	0	2.516216	0.965148	0.139146
33	1	0	3.378760	-0.241724	2.895438
34	1	0	3.390128	0.401173	-2.877691
35	7	0	-2.362737	2.432390	0.243055
36	7	0	-2.198025	-2.300382	-0.223077
37	6	0	-2.954104	-2.220352	-1.467993
38	1	0	-2.530720	-2.888405	-2.218411
39	1	0	-3.977744	-2.521806	-1.274319
40	1	0	-2.953922	-1.201699	-1.861535
41	6	0	-3.062532	2.751613	-0.995637
42	1	0	-2.561844	3.563030	-1.525053
43	1	0	-3.113047	1.881352	-1.652992
44	1	0	-4.072207	3.066186	-0.754187
45	6	0	-2.910034	-2.540692	1.027554
46	1	0	-2.473081	-3.383798	1.563092
47	1	0	-2.878903	-1.659136	1.671198

48	1	0	-3.944957	-2.772339	0.800152
49	6	0	-3.100435	2.436808	1.500550
50	1	0	-2.597241	3.060477	2.239911
51	1	0	-4.090795	2.841966	1.322946
52	1	0	-3.199511	1.426068	1.901883

Most stable energy, Gibbs free energy (Ha), and geometry for protomer ./NMe2_Py2N2//2_3
E: -1030.962483
G: -1030.546553
Geometry:

Input orientation:

Center Number	Atomic Number	Atomic Type	Coordinates (Angstroms) X	Y	Z
1	6	0	-1.091926	1.847581	0.426568
2	6	0	-0.775168	1.002423	1.522523
3	6	0	-1.249280	-0.276558	1.562756
4	7	0	-2.038280	-0.730953	0.572382
5	6	0	-2.265980	-0.016965	-0.552090
6	6	0	-1.842662	1.270175	-0.642443
7	6	0	-2.896176	-0.740688	-1.701223
8	7	0	-1.957985	-1.757511	-2.286589
9	6	0	-0.624303	-1.242666	-2.721436
10	6	0	0.334695	-1.235787	-1.553045
11	7	0	0.117903	-2.160202	-0.621767
12	6	0	0.885833	-2.107550	0.477230
13	6	0	1.935149	-1.226074	0.619851
14	6	0	2.215255	-0.288156	-0.399874
15	6	0	1.336902	-0.288590	-1.500910
16	6	0	0.480678	-2.994104	1.627401
17	7	0	-0.853991	-2.626817	2.125938
18	6	0	-0.922652	-1.268107	2.651201
19	1	0	-0.124823	1.331413	2.318555
20	1	0	-2.024768	1.808140	-1.559773
21	1	0	-3.145288	-0.040173	-2.492424
22	1	0	-3.788439	-1.281518	-1.389920
23	1	0	-0.758262	-0.247423	-3.139886
24	1	0	-0.271415	-1.913258	-3.503788
25	1	0	2.498656	-1.234511	1.540742
26	1	0	1.408207	0.445660	-2.290110
27	1	0	0.436147	-4.031411	1.294054
28	1	0	1.237048	-2.916805	2.414881
29	1	0	0.001873	-0.941244	3.138950
30	1	0	-1.720975	-1.223300	3.394404
31	1	0	-2.432694	-2.187047	-3.083859
32	1	0	-2.259229	-1.725087	0.607513
33	1	0	-1.104469	-3.271701	2.864883
34	1	0	-1.771678	-2.501347	-1.598231
35	7	0	3.241129	0.592530	-0.298732
36	7	0	-0.650339	3.101233	0.361167
37	6	0	-0.893360	3.911898	-0.827243
38	1	0	-0.537618	4.919241	-0.637953
39	1	0	-0.366787	3.507395	-1.694528
40	1	0	-1.959169	3.957847	-1.048747
41	6	0	3.924076	0.741533	0.977206
42	1	0	4.723275	1.467776	0.861983
43	1	0	3.245854	1.085723	1.764745
44	1	0	4.368786	-0.202266	1.291331

45	6	0	0.257570	3.606162	1.384800
46	1	0	0.480814	4.645460	1.169733
47	1	0	-0.202168	3.545603	2.371350
48	1	0	1.190073	3.036699	1.393829
49	6	0	3.323558	1.694492	-1.243538
50	1	0	3.391353	1.322901	-2.265856
51	1	0	2.457443	2.360855	-1.172789
52	1	0	4.222018	2.267536	-1.034059

Most stable energy, Gibbs free energy (Ha), and geometry for protomer ./NMe2_Py2N2//2_6
E: -1030.964576
G: -1030.546663
Geometry:
Input orientation:

Center Number	Atomic Number	Atomic Type	Coordinates (Angstroms) X	Y	Z
1	6	0	-1.845595	-1.053804	-0.363300
2	6	0	-2.185574	-0.166330	0.684210
3	6	0	-1.863433	1.164676	0.565871
4	7	0	-1.282140	1.704554	-0.510350
5	6	0	-0.895333	0.863461	-1.465955
6	6	0	-1.136679	-0.495408	-1.447057
7	6	0	-0.138271	1.483936	-2.616932
8	7	0	0.725365	2.610721	-2.141508
9	6	0	2.109615	2.226604	-1.698060
10	6	0	1.984591	1.307577	-0.518717
11	7	0	1.431554	1.860272	0.564957
12	6	0	1.034738	1.022935	1.521616
13	6	0	1.230850	-0.341239	1.490208
14	6	0	1.891840	-0.918112	0.385999
15	6	0	2.266272	-0.032362	-0.648598
16	6	0	0.269375	1.657522	2.656318
17	7	0	-0.707898	2.659146	2.122517
18	6	0	-2.043949	2.106811	1.721910
19	1	0	-2.636248	-0.518641	1.599844
20	1	0	-0.751663	-1.108531	-2.249054
21	1	0	-0.836386	1.908865	-3.337009
22	1	0	0.492060	0.755819	-3.122425
23	1	0	2.617357	3.153093	-1.438221
24	1	0	2.600469	1.752705	-2.543513
25	1	0	0.829193	-0.947310	2.288997
26	1	0	2.690924	-0.390972	-1.574274
27	1	0	-0.280979	0.920934	3.236388
28	1	0	0.946602	2.200426	3.313984
29	1	0	-0.271914	3.096784	1.298410
30	1	0	-2.658453	2.961638	1.444939
31	1	0	-2.467202	1.613280	2.592378
32	1	0	0.237084	3.047713	-1.346568
33	1	0	-0.856599	3.389622	2.820958
34	1	0	0.805930	3.309752	-2.881647
35	7	0	2.086358	-2.254691	0.285128
36	7	0	-2.139071	-2.374044	-0.299827
37	6	0	-1.505959	-3.286564	-1.239566
38	1	0	-1.850560	-4.295493	-1.032675
39	1	0	-1.779354	-3.038958	-2.265719
40	1	0	-0.414282	-3.263614	-1.155396
41	6	0	2.519723	-2.806893	-0.990176

42	1	0	1.808244	-2.585833	-1.792407
43	1	0	3.493437	-2.406592	-1.272615
44	1	0	2.613430	-3.884008	-0.890068
45	6	0	-2.669553	-2.923319	0.938678
46	1	0	-1.970741	-2.803287	1.772469
47	1	0	-3.609155	-2.437555	1.203063
48	1	0	-2.864425	-3.981832	0.794864
49	6	0	1.381307	-3.135308	1.205193
50	1	0	1.698015	-2.957785	2.233246
51	1	0	0.296530	-2.994270	1.147307
52	1	0	1.615162	-4.165331	0.952591

Most stable energy, Gibbs free energy (Ha), and geometry for protomer ./NMe2_Py2N2//2_2

E: -1030.962487

G: -1030.546721

Geometry:

Input orientation:

Center Number	Atomic Number	Atomic Type	Coordinates (Angstroms) X	Y	Z
1	6	0	-2.228658	0.296178	0.224296
2	6	0	-1.878087	1.195334	-0.808406
3	6	0	-0.850138	2.093583	-0.621826
4	7	0	-0.170341	2.199853	0.529948
5	6	0	-0.452274	1.312517	1.479888
6	6	0	-1.440500	0.353598	1.390460
7	6	0	0.417707	1.378880	2.714136
8	7	0	1.783524	1.863841	2.348707
9	6	0	2.749669	0.810816	1.887181
10	6	0	2.204709	0.045998	0.721325
11	7	0	2.067404	0.715263	-0.444902
12	6	0	1.354262	0.222309	-1.474057
13	6	0	0.876404	-1.055055	-1.420943
14	6	0	1.108329	-1.857222	-0.272651
15	6	0	1.771165	-1.236079	0.829176
16	6	0	1.111715	1.170994	-2.621419
17	7	0	1.005478	2.549568	-2.157229
18	6	0	-0.362156	2.933917	-1.774215
19	1	0	-2.368746	1.160275	-1.769495
20	1	0	-1.565869	-0.349521	2.200912
21	1	0	0.515230	0.408140	3.195222
22	1	0	0.014076	2.093347	3.430295
23	1	0	2.920578	0.141974	2.725526
24	1	0	3.673678	1.325769	1.629102
25	1	0	0.286287	-1.414844	-2.250116
26	1	0	1.879749	-1.737333	1.778225
27	1	0	1.962881	1.098574	-3.300945
28	1	0	0.225528	0.824123	-3.163431
29	1	0	-1.058432	2.821993	-2.611281
30	1	0	-0.341832	3.984107	-1.481536
31	1	0	2.205281	2.332100	3.153942
32	1	0	2.297501	1.706039	-0.503581
33	1	0	1.310570	3.164562	-2.901069
34	1	0	1.651477	2.571099	1.610898
35	7	0	0.666809	-3.110112	-0.193235
36	7	0	-3.233466	-0.601936	0.077748
37	6	0	-3.397017	-1.651913	1.069752
38	1	0	-3.538977	-1.225419	2.062475

39	1	0	-4.282397	-2.230490	0.823002
40	1	0	-2.534113	-2.325542	1.101291
41	6	0	0.814494	-3.869611	1.043681
42	1	0	1.860100	-3.908137	1.347530
43	1	0	0.225773	-3.427428	1.850363
44	1	0	0.468635	-4.883265	0.871090
45	6	0	-3.795981	-0.825080	-1.245685
46	1	0	-4.571386	-1.581678	-1.171445
47	1	0	-4.249383	0.086385	-1.634828
48	1	0	-3.037560	-1.167057	-1.957108
49	6	0	-0.150537	-3.663726	-1.266655
50	1	0	0.383040	-3.622560	-2.216125
51	1	0	-0.369591	-4.700920	-1.037394
52	1	0	-1.090517	-3.115178	-1.364685

Most stable energy, Gibbs free energy (Ha), and geometry for protomer ./NMe2_Py2N2//2_5
E: -1030.962481
G: -1030.546548
Geometry:

Input orientation:

Center Number	Atomic Number	Atomic Type	Coordinates (Angstroms) X	Y	Z
1	6	0	-1.080441	-1.853263	-0.430228
2	6	0	-1.834768	-1.281302	0.639158
3	6	0	-2.265223	0.003598	0.550142
4	7	0	-2.040788	0.720206	-0.573346
5	6	0	-1.248537	0.271453	-1.563723
6	6	0	-0.767333	-1.004910	-1.524728
7	6	0	-0.926886	1.266044	-2.650889
8	7	0	-0.866224	2.624560	-2.124168
9	6	0	0.465949	2.998758	-1.624192
10	6	0	0.874729	2.113848	-0.474040
11	7	0	0.105512	2.162117	0.624265
12	6	0	0.326259	1.238543	1.555429
13	6	0	1.333398	0.296590	1.503920
14	6	0	2.212566	0.300890	0.403540
15	6	0	1.928705	1.237862	-0.616040
16	6	0	-0.633912	1.240048	2.722847
17	7	0	-1.970584	1.745468	2.286117
18	6	0	-2.900901	0.722206	1.699548
19	1	0	-2.014161	-1.821336	1.555820
20	1	0	-0.114497	-1.329250	-2.320628
21	1	0	-1.724549	1.217526	-3.394566
22	1	0	-0.000251	0.945015	-3.138550
23	1	0	0.415537	4.035663	-1.290411
24	1	0	1.223486	2.925908	-2.410982
25	1	0	1.407755	-0.437625	2.292882
26	1	0	2.492941	1.249400	-1.536460
27	1	0	-0.761792	0.244320	3.142022
28	1	0	-0.286398	1.913753	3.504895
29	1	0	-1.788551	2.490748	1.598210
30	1	0	-3.796887	1.256766	1.388105
31	1	0	-3.145488	0.019435	2.490172
32	1	0	-2.267669	1.713025	-0.607751
33	1	0	-2.449337	2.171651	3.082759
34	1	0	-1.119784	3.268813	-2.862608
35	7	0	3.242599	-0.574903	0.302814

36	7	0	-0.632525	-3.104762	-0.366378
37	6	0	0.279534	-3.603198	-1.389496
38	1	0	-0.178834	-3.542897	-2.376694
39	1	0	1.209247	-3.029138	-1.395828
40	1	0	0.507479	-4.641837	-1.176151
41	6	0	3.928291	-0.719287	-0.972152
42	1	0	4.369445	0.226815	-1.284392
43	1	0	4.730394	-1.442250	-0.856522
44	1	0	3.252853	-1.065419	-1.761232
45	6	0	-0.872472	-3.918423	0.820566
46	1	0	-0.347216	-3.513709	1.688561
47	1	0	-1.938091	-3.968533	1.042063
48	1	0	-0.513300	-4.924186	0.629330
49	6	0	3.329875	-1.676557	1.247554
50	1	0	2.466271	-2.346196	1.177229
51	1	0	4.230368	-2.246158	1.037452
52	1	0	3.396812	-1.304793	2.269892

Most stable energy, Gibbs free energy (Ha), and geometry for protomer ./NMe2_Py2N2//2_4
E: -1030.962479
G: -1030.546571
Geometry:
Input orientation:

Center Number	Atomic Number	Atomic Type	Coordinates (Angstroms) X	Y	Z
1	6	0	-2.209672	0.320375	-0.403694
2	6	0	-1.331305	0.309838	-1.504688
3	6	0	-0.316062	1.243005	-1.555696
4	7	0	-0.086892	2.163660	-0.623697
5	6	0	-0.855916	2.120938	0.474935
6	6	0	-1.917319	1.253892	0.616663
7	6	0	-0.438732	3.001163	1.625667
8	7	0	0.890396	2.614850	2.124529
9	6	0	0.939150	1.255784	2.651049
10	6	0	1.250329	0.258262	1.563493
11	7	0	2.045916	0.699436	0.572339
12	6	0	2.262577	-0.019320	-0.551339
13	6	0	1.820007	-1.300200	-0.639780
14	6	0	1.061741	-1.864999	0.430611
15	6	0	0.757195	-1.013539	1.525059
16	6	0	2.904769	0.693172	-1.701044
17	7	0	1.985701	1.727539	-2.285695
18	6	0	0.643690	1.237407	-2.723410
19	1	0	-1.412335	-0.423067	-2.294230
20	1	0	-2.480857	1.269205	1.537471
21	1	0	-1.196400	2.934309	2.412859
22	1	0	-0.379388	4.037881	1.292751
23	1	0	0.010137	0.943404	3.139865
24	1	0	1.737200	1.199822	3.393779
25	1	0	1.993159	-1.841978	-1.556623
26	1	0	0.102089	-1.331725	2.321578
27	1	0	3.807151	1.217193	-1.390161
28	1	0	3.140521	-0.011609	-2.492594
29	1	0	2.469040	2.149877	-3.081595
30	1	0	0.761262	0.241406	-3.144908
31	1	0	0.303158	1.916655	-3.503675
32	1	0	2.282744	1.689910	0.606909

33	1	0	1.811642	2.473734	-1.596728
34	1	0	1.150424	3.256663	2.862838
35	7	0	0.602917	-3.112600	0.367856
36	7	0	-3.246779	-0.547050	-0.303205
37	6	0	-3.932279	-0.687633	0.972306
38	1	0	-4.367181	0.261078	1.285458
39	1	0	-4.739029	-1.405460	0.857043
40	1	0	-3.258337	-1.038433	1.760600
41	6	0	-0.313826	-3.601983	1.391164
42	1	0	-1.238440	-3.019695	1.396550
43	1	0	-0.550851	-4.638800	1.178825
44	1	0	0.144683	-3.544644	2.378477
45	6	0	-3.343827	-1.646702	-1.249361
46	1	0	-2.485265	-2.323004	-1.181227
47	1	0	-4.248322	-2.209693	-1.038658
48	1	0	-3.409417	-1.273035	-2.271101
49	6	0	0.835481	-3.929141	-0.818528
50	1	0	0.469727	-4.932216	-0.625639
51	1	0	0.311841	-3.521777	-1.686272
52	1	0	1.900436	-3.986856	-1.041377

Most stable energy, Gibbs free energy (Ha), and geometry for protomer ./OMe_Py2N2//0_1
E: -991.168369
G: -990.859416
Geometry:

Input orientation:

Center Number	Atomic Number	Atomic Type	Coordinates (Angstroms) X	Y	Z
1	6	0	-1.547593	-1.133492	1.193605
2	6	0	-0.851075	-1.868051	0.241933
3	6	0	0.536247	-1.747729	0.207562
4	7	0	1.217102	-0.975185	1.047707
5	6	0	0.534969	-0.217270	1.915990
6	6	0	-0.838728	-0.293214	2.049351
7	6	0	1.309101	0.827348	2.691528
8	7	0	1.093369	2.215041	2.260843
9	6	0	1.669565	2.580062	0.963473
10	6	0	1.117382	1.833947	-0.233778
11	7	0	1.953912	1.069194	-0.923594
12	6	0	1.436022	0.256739	-1.861013
13	6	0	0.103411	0.266248	-2.207825
14	6	0	-0.767721	1.092301	-1.495254
15	6	0	-0.253004	1.895088	-0.491547
16	6	0	2.336716	-0.848682	-2.345333
17	7	0	2.563461	-1.874935	-1.315078
18	6	0	1.344586	-2.519045	-0.823554
19	1	0	-1.352674	-2.493341	-0.483083
20	1	0	-1.368435	0.329015	2.760491
21	1	0	2.373837	0.608275	2.612822
22	1	0	1.029462	0.767251	3.744336
23	1	0	2.744951	2.409024	1.005318
24	1	0	1.506022	3.650656	0.827119
25	1	0	-0.287923	-0.423715	-2.944617
26	1	0	-0.885361	2.518072	0.126106
27	1	0	1.898396	-1.329210	-3.220150
28	1	0	3.308057	-0.442846	-2.627630
29	1	0	1.623019	-3.472911	-0.366687

30	1	0	0.702859	-2.756383	-1.673445
31	1	0	3.002100	-1.415862	-0.521186
32	1	0	0.094667	2.401335	2.247420
33	6	0	-3.648382	-1.820671	0.330702
34	1	0	-4.692751	-1.644621	0.573116
35	1	0	-3.425776	-1.419539	-0.660569
36	1	0	-3.442489	-2.892099	0.350877
37	6	0	-3.004805	1.648719	-0.938721
38	1	0	-2.883427	2.731593	-0.997534
39	1	0	-3.994073	1.371261	-1.292169
40	1	0	-2.872919	1.316992	0.093741
41	8	0	-2.893541	-1.139102	1.326138
42	8	0	-2.080087	0.988722	-1.797370

Most stable energy, Gibbs free energy (Ha), and geometry for protomer ./OMe_Py2N2//1_3

E: -991.629674

G: -991.306117

Geometry:

Input orientation:

Center Number	Atomic Number	Atomic Type	Coordinates (Angstroms) X	Y	Z
1	6	0	-1.693864	-1.035305	-1.116673
2	6	0	-0.962689	-0.249661	-2.005532
3	6	0	0.417148	-0.262008	-1.934024
4	7	0	1.079369	-1.064475	-1.089562
5	6	0	0.375534	-1.786897	-0.228822
6	6	0	-1.011285	-1.816374	-0.189001
7	6	0	1.173556	-2.597729	0.767188
8	7	0	2.474017	-1.930847	1.085121
9	6	0	2.432123	-0.921681	2.201371
10	6	0	1.549816	0.208320	1.763661
11	7	0	2.052876	0.971459	0.782285
12	6	0	1.218004	1.782560	0.144801
13	6	0	-0.126244	1.917522	0.495450
14	6	0	-0.619331	1.153390	1.540013
15	6	0	0.250316	0.281029	2.202517
16	6	0	1.734719	2.495072	-1.086830
17	7	0	1.087798	2.127733	-2.348308
18	6	0	1.225124	0.723198	-2.750638
19	1	0	-1.478878	0.404160	-2.697847
20	1	0	-1.518283	-2.403602	0.562934
21	1	0	1.414973	-3.574194	0.348607
22	1	0	0.625560	-2.738397	1.696291
23	1	0	3.457843	-0.597949	2.361432
24	1	0	2.052960	-1.431289	3.082958
25	1	0	-0.762298	2.573561	-0.083122
26	1	0	-0.123310	-0.381141	2.972514
27	1	0	1.607067	3.570971	-0.957597
28	1	0	2.801490	2.291545	-1.175173
29	1	0	2.278789	0.451698	-2.690227
30	1	0	0.917173	0.649143	-3.794432
31	1	0	2.783194	-1.450680	0.227552
32	1	0	0.100987	2.365159	-2.302450
33	1	0	3.168940	-2.642830	1.316927
34	6	0	-3.797856	-1.580329	-0.167771
35	1	0	-4.835882	-1.334476	-0.372517
36	1	0	-3.662940	-2.662499	-0.197529

37	1	0	-3.512178	-1.195923	0.813666
38	6	0	-2.850391	1.841841	1.136042
39	1	0	-2.819915	1.491204	0.101982
40	1	0	-3.824480	1.633977	1.569487
41	1	0	-2.651694	2.914022	1.171046
42	8	0	-3.036075	-0.946664	-1.191354
43	8	0	-1.906684	1.128272	1.931451

Most stable energy, Gibbs free energy (Ha), and geometry for protomer ./OMe_Py2N2//1_2

E: -991.628490

G: -991.308690

Geometry:

Input orientation:

Center Number	Atomic Number	Atomic Type	Coordinates (Angstroms) X	Y	Z
1	6	0	-3.931647	-0.132809	0.413870
2	6	0	-3.152999	-1.302835	0.437724
3	6	0	-1.846001	-1.233555	0.050733
4	7	0	-1.341541	-0.052452	-0.363613
5	6	0	-2.041743	1.080119	-0.392648
6	6	0	-3.367052	1.071167	-0.000945
7	6	0	-1.368388	2.340227	-0.897193
8	7	0	0.051713	2.287184	-1.161786
9	6	0	0.930738	2.181240	0.006958
10	6	0	1.869612	0.999188	-0.039533
11	7	0	1.333518	-0.158474	-0.428396
12	6	0	2.060120	-1.274918	-0.404316
13	6	0	3.384086	-1.265322	-0.005893
14	6	0	3.963067	-0.055349	0.366431
15	6	0	3.190690	1.108144	0.354220
16	6	0	1.400483	-2.569982	-0.823964
17	7	0	-0.022842	-2.439573	-1.097005
18	6	0	-0.871793	-2.381713	0.093695
19	1	0	-3.587876	-2.237450	0.762838
20	1	0	-3.928741	1.993004	-0.031120
21	1	0	-1.574328	3.132831	-0.176537
22	1	0	-1.890342	2.619384	-1.814857
23	1	0	0.327402	2.078880	0.915718
24	1	0	1.522456	3.089687	0.125825
25	1	0	3.968942	-2.175782	0.014730
26	1	0	3.589500	2.068489	0.644936
27	1	0	1.888053	-2.912507	-1.738213
28	1	0	1.617666	-3.320038	-0.053533
29	1	0	-0.250091	-2.241763	0.983223
30	1	0	-1.434379	-3.303219	0.243109
31	1	0	-0.296206	-0.064901	-0.583711
32	1	0	-0.311242	-3.222744	-1.667309
33	1	0	0.266970	1.576297	-1.850418
34	6	0	-6.034595	0.892376	0.799007
35	1	0	-6.119320	1.293911	-0.211061
36	1	0	-7.005685	0.555113	1.147616
37	1	0	-5.637904	1.650250	1.475016
38	6	0	5.879665	1.130851	1.114857
39	1	0	5.386420	1.550252	1.992777
40	1	0	6.907874	0.877889	1.356400
41	1	0	5.858975	1.850824	0.295591
42	8	0	-5.198180	-0.266252	0.804746

43	8	0	5.259264	-0.091888	0.725188

Most stable energy, Gibbs free energy (Ha), and geometry for protomer ./OMe_Py2N2//1_1
E: -991.628471
G: -991.308544
Geometry:
Input orientation:

Center Number	Atomic Number	Atomic Type	Coordinates (Angstroms) X	Y	Z
1	6	0	-3.976801	0.267419	-0.295060
2	6	0	-3.341722	1.381993	0.244603
3	6	0	-2.007531	1.283565	0.595447
4	7	0	-1.323547	0.154436	0.417404
5	6	0	-1.915269	-0.908696	-0.130158
6	6	0	-3.249714	-0.909078	-0.491189
7	6	0	-1.025238	-2.100930	-0.387871
8	7	0	-0.151170	-2.439912	0.739599
9	6	0	1.265693	-2.506817	0.461823
10	6	0	1.997514	-1.209365	0.184249
11	7	0	1.345583	-0.057763	0.335050
12	6	0	1.907100	1.151843	0.127722
13	6	0	3.223123	1.226191	-0.225936
14	6	0	3.951924	0.034994	-0.386621
15	6	0	3.329402	-1.194458	-0.184867
16	6	0	0.983314	2.333867	0.263071
17	7	0	0.124506	2.240834	1.444390
18	6	0	-1.289057	2.474392	1.190914
19	1	0	-3.890957	2.303397	0.389016
20	1	0	-3.693058	-1.798243	-0.913790
21	1	0	-0.419086	-1.868156	-1.270256
22	1	0	-1.653445	-2.951504	-0.655464
23	1	0	1.437836	-3.167172	-0.389441
24	1	0	1.772500	-2.968165	1.311827
25	1	0	3.702715	2.181563	-0.386236
26	1	0	3.852262	-2.132248	-0.301075
27	1	0	1.586719	3.241587	0.263701
28	1	0	0.365678	2.363290	-0.639591
29	1	0	-1.464879	3.333369	0.531471
30	1	0	-1.767368	2.710753	2.142686
31	1	0	0.297181	-0.034695	0.544179
32	1	0	0.440654	2.910736	2.132080
33	1	0	-0.338411	-1.849087	1.540576
34	6	0	-5.955642	-0.719900	-1.161000
35	1	0	-6.978873	-0.396971	-1.328993
36	1	0	-5.499391	-1.010135	-2.108358
37	1	0	-5.943335	-1.561553	-0.467170
38	6	0	6.017904	-1.003926	-0.911705
39	1	0	5.605240	-1.618184	-1.712461
40	1	0	7.009673	-0.656444	-1.183615
41	1	0	6.063322	-1.574069	0.016465
42	8	0	-5.279902	0.403418	-0.600899
43	8	0	5.230535	0.175158	-0.733495

Most stable energy, Gibbs free energy (Ha), and geometry for protomer ./OMe_Py2N2//1_4
E: -991.629661
G: -991.306050
Geometry:
Input orientation:

Center Atomic Atomic Coordinates (Angstroms)
Number Number Type X Y Z

Center Number	Atomic Number	Atomic Type	X	Y	Z
1	6	0	0.627664	-1.127926	1.554678
2	6	0	-0.244190	-0.249311	2.205974
3	6	0	-1.545069	-0.188891	1.769335
4	7	0	-2.047064	-0.968807	0.800702
5	6	0	-1.210274	-1.786205	0.173841
6	6	0	0.135231	-1.910572	0.523601
7	6	0	-1.726426	-2.518900	-1.046088
8	7	0	-1.084664	-2.165998	-2.314384
9	6	0	-1.229310	-0.767802	-2.735708
10	6	0	-0.422900	0.232186	-1.935626
11	7	0	-1.086098	1.045058	-1.102002
12	6	0	-0.383017	1.782728	-0.253602
13	6	0	1.003768	1.817798	-0.217337
14	6	0	1.687098	1.025352	-1.134749
15	6	0	0.956824	0.223607	-2.009884
16	6	0	-1.182125	2.604553	0.732585
17	7	0	-2.479273	1.936864	1.062263
18	6	0	-2.431370	0.943538	2.192366
19	1	0	0.128500	0.426305	2.964680
20	1	0	0.772475	-2.573091	-0.046228
21	1	0	-2.794502	-2.322373	-1.134480
22	1	0	-1.592615	-3.592158	-0.902030
23	1	0	-2.283915	-0.499900	-2.675599
24	1	0	-0.925153	-0.706978	-3.781465
25	1	0	1.510282	2.418051	0.524595
26	1	0	1.473871	-0.438836	-2.693309
27	1	0	-0.632921	2.760312	1.658639
28	1	0	-1.428201	3.574113	0.300909
29	1	0	-2.787618	1.443205	0.212067
30	1	0	-3.455464	0.617813	2.358591
31	1	0	-2.053018	1.466746	3.066329
32	1	0	-3.176955	2.649073	1.284919
33	1	0	-0.096650	-2.398331	-2.268204
34	6	0	2.860949	-1.811513	1.154547
35	1	0	2.668818	-2.884142	1.207852
36	1	0	2.824523	-1.478113	0.114975
37	1	0	3.835401	-1.590882	1.580805
38	6	0	3.791433	1.590826	-0.198407
39	1	0	3.653155	2.672052	-0.243177
40	1	0	3.509138	1.219446	0.789025
41	1	0	4.829770	1.345296	-0.401980
42	8	0	1.916003	-1.090608	1.941851
43	8	0	3.029447	0.940358	-1.211255

Most stable energy, Gibbs free energy (Ha), and geometry for protomer ./OMe_Py2N2//2_1
E: -992.086573
G: -991.752066
Geometry:
Input orientation:

Center Atomic Atomic Coordinates (Angstroms)
Number Number Type X Y Z

Center Number	Atomic Number	Atomic Type	X	Y	Z
1	6	0	1.226627	-1.863293	-0.793699
2	6	0	0.440243	-1.399656	-1.863089

3	6	0	-0.881767	-1.137741	-1.654899
4	7	0	-1.407887	-1.377769	-0.434104
5	6	0	-0.686545	-1.760143	0.627706
6	6	0	0.653438	-2.026332	0.471708
7	6	0	-1.409353	-1.836859	1.948233
8	7	0	-2.372915	-0.750126	2.094651
9	6	0	-1.794392	0.470832	2.658240
10	6	0	-0.890539	1.124965	1.652507
11	7	0	-1.416848	1.358862	0.430551
12	6	0	-0.697629	1.748937	-0.629877
13	6	0	0.639042	2.029990	-0.471190
14	6	0	1.211205	1.874404	0.795530
15	6	0	0.428075	1.401634	1.863293
16	6	0	-1.418712	1.819085	-1.951784
17	7	0	-2.376000	0.726928	-2.098335
18	6	0	-1.790208	-0.491031	-2.661224
19	1	0	0.895087	-1.199859	-2.822913
20	1	0	1.230688	-2.316744	1.336492
21	1	0	-0.658642	-1.854902	2.744132
22	1	0	-1.949155	-2.784963	1.977241
23	1	0	-1.213419	0.285471	3.565670
24	1	0	-2.609886	1.152872	2.902612
25	1	0	1.214783	2.326561	-1.334883
26	1	0	0.883039	1.207643	2.824249
27	1	0	-1.963557	2.764240	-1.983216
28	1	0	-0.666752	1.840238	-2.746438
29	1	0	-1.210838	-0.302865	-3.569074
30	1	0	-2.601585	-1.178356	-2.904379
31	1	0	-2.384076	-1.135399	-0.270800
32	1	0	-2.390162	1.106222	0.265352
33	1	0	-3.132592	1.027800	-2.699113
34	1	0	-3.127996	-1.055569	2.695024
35	6	0	3.375611	-2.457965	0.010464
36	1	0	4.359659	-2.558395	-0.435580
37	1	0	3.389691	-1.688806	0.783190
38	1	0	3.053076	-3.411652	0.427414
39	6	0	3.353551	2.498201	-0.003964
40	1	0	4.334999	2.613124	0.444319
41	1	0	3.380683	1.729368	-0.776689
42	1	0	3.017917	3.447049	-0.421617
43	8	0	2.507544	-2.064940	-1.058018
44	8	0	2.488922	2.092470	1.062590

Most stable energy, Gibbs free energy (Ha), and geometry for protomer ./OMe_Py2N2//2_3
E: -992.081276
G: -991.744279
Geometry:

Input orientation:

Center Number	Atomic Number	Atomic Type	Coordinates (Angstroms) X	Y	Z
1	6	0	-1.343983	-1.942407	-0.093838
2	6	0	-1.231326	-1.327745	1.153210
3	6	0	-1.471537	0.026157	1.246408
4	7	0	-1.840658	0.706225	0.158397
5	6	0	-1.873863	0.167131	-1.080406
6	6	0	-1.651448	-1.166681	-1.230504
7	6	0	-2.080216	1.096867	-2.235212

8	7	0	-0.923193	2.037021	-2.408533
9	6	0	0.418381	1.401915	-2.576039
10	6	0	1.021545	1.101844	-1.224010
11	7	0	0.673274	1.913433	-0.238078
12	6	0	1.100225	1.622833	1.001549
13	6	0	1.958121	0.575405	1.255724
14	6	0	2.361922	-0.244546	0.196964
15	6	0	1.862829	0.007240	-1.075090
16	6	0	0.507354	2.424714	2.130992
17	7	0	-0.945774	2.207931	2.214253
18	6	0	-1.308369	0.828933	2.512271
19	1	0	-0.926633	-1.864647	2.039138
20	1	0	-1.663002	-1.621550	-2.210645
21	1	0	-2.966018	1.713068	-2.090110
22	1	0	-2.173254	0.526904	-3.154853
23	1	0	1.037099	2.113365	-3.121322
24	1	0	0.299735	0.500558	-3.173818
25	1	0	2.283677	0.352445	2.263051
26	1	0	2.084521	-0.622566	-1.924043
27	1	0	1.009305	2.144005	3.061768
28	1	0	0.675147	3.487263	1.954061
29	1	0	-2.265406	0.817162	3.037137
30	1	0	-0.580035	0.311034	3.144813
31	1	0	-0.855913	2.667638	-1.596974
32	1	0	-1.922675	1.717794	0.285843
33	1	0	-1.317535	2.808619	2.938919
34	1	0	-1.126910	2.616316	-3.226679
35	6	0	-0.753220	-4.056469	0.793353
36	1	0	0.192772	-3.715067	1.213964
37	1	0	-1.535320	-4.044514	1.552319
38	1	0	-0.642468	-5.055542	0.384871
39	6	0	3.549400	-2.155056	-0.549071
40	1	0	2.666855	-2.645761	-0.962801
41	1	0	4.082813	-1.619347	-1.335292
42	1	0	4.203932	-2.893549	-0.095933
43	8	0	-1.125702	-3.227630	-0.313362
44	8	0	3.178489	-1.265073	0.501844

Most stable energy, Gibbs free energy (Ha), and geometry for protomer ./OMe_Py2N2//2_6
E: -992.088799
G: -991.750207
Geometry:

Input orientation:

Center Number	Atomic Number	Atomic Type	Coordinates (Angstroms) X	Y	Z
1	6	0	-1.825953	-1.258333	-0.218612
2	6	0	-1.871616	-0.403379	-1.324373
3	6	0	-1.652862	0.938399	-1.119601
4	7	0	-1.459243	1.476444	0.090459
5	6	0	-1.384316	0.654380	1.125542
6	6	0	-1.563201	-0.719970	1.035917
7	6	0	-1.033661	1.291170	2.448541
8	7	0	-0.003171	2.359980	2.258249
9	6	0	1.423445	1.889415	2.271044
10	6	0	1.635220	0.950553	1.119742
11	7	0	1.433425	1.485061	-0.090611
12	6	0	1.369052	0.661553	-1.125153

13	6	0	1.567085	-0.710166	-1.034761
14	6	0	1.838156	-1.244061	0.219838
15	6	0	1.872188	-0.387964	1.325233
16	6	0	1.011355	1.292420	-2.449234
17	7	0	-0.024578	2.356181	-2.260458
18	6	0	-1.448914	1.878126	-2.271534
19	1	0	-1.999062	-0.807569	-2.319641
20	1	0	-1.451408	-1.334472	1.917316
21	1	0	-0.642725	0.562548	3.155073
22	1	0	-1.911166	1.771210	2.879336
23	1	0	1.597621	1.407732	3.229184
24	1	0	2.042866	2.779287	2.180621
25	1	0	1.463969	-1.326650	-1.915778
26	1	0	2.006198	-0.789684	2.320636
27	1	0	1.885294	1.775699	-2.883677
28	1	0	0.623180	0.559059	-3.152421
29	1	0	0.082450	3.061832	-2.991775
30	1	0	-1.621481	1.395255	-3.229362
31	1	0	-2.072900	2.764685	-2.180393
32	1	0	-0.114154	3.066384	2.988281
33	1	0	0.164908	2.813488	-1.357677
34	1	0	-0.194525	2.814759	1.354602
35	6	0	-1.754594	-3.475595	0.614542
36	1	0	-2.497968	-3.322056	1.397776
37	1	0	-1.862819	-4.470435	0.192900
38	1	0	-0.749412	-3.351408	1.022857
39	6	0	1.802556	-3.462148	-0.613324
40	1	0	0.796067	-3.354299	-1.022923
41	1	0	2.544318	-3.296340	-1.395577
42	1	0	1.926355	-4.455156	-0.191643
43	8	0	-1.968967	-2.568497	-0.465722
44	8	0	2.000863	-2.551866	0.467308

Most stable energy, Gibbs free energy (Ha), and geometry for protomer ./OMe_Py2N2//2_2
E: -992.081284
G: -991.744239
Geometry:

Input orientation:

Center Number	Atomic Number	Atomic Type	Coordinates (Angstroms) X	Y	Z
1	6	0	-2.353448	-0.292141	-0.190817
2	6	0	-1.968501	0.514993	-1.266245
3	6	0	-1.134273	1.586262	-1.034020
4	7	0	-0.712973	1.911223	0.199064
5	6	0	-1.043530	1.112834	1.201761
6	6	0	-1.860942	-0.002681	1.075711
7	6	0	-0.445162	1.452862	2.546560
8	7	0	0.882772	2.111859	2.364543
9	6	0	2.057754	1.191949	2.201964
10	6	0	1.864635	0.229889	1.071361
11	7	0	1.816536	0.736497	-0.180590
12	6	0	1.458849	0.022280	-1.249993
13	6	0	1.248295	-1.333902	-1.123192
14	6	0	1.378822	-1.914815	0.138330
15	6	0	1.671826	-1.104400	1.254837
16	6	0	1.275414	0.791692	-2.533729
17	7	0	0.898280	2.173074	-2.266044

18	6	0	-0.557035	2.375043	-2.180486
19	1	0	-2.288637	0.263996	-2.268733
20	1	0	-2.069619	-0.619805	1.937205
21	1	0	-0.307291	0.566487	3.162298
22	1	0	-1.077961	2.162232	3.078316
23	1	0	2.170414	0.646179	3.134077
24	1	0	2.929279	1.822963	2.035515
25	1	0	0.952356	-1.898560	-1.994825
26	1	0	1.696388	-1.534814	2.245739
27	1	0	2.228085	0.778423	-3.066403
28	1	0	0.548067	0.249977	-3.147200
29	1	0	-1.059482	2.071373	-3.103712
30	1	0	-0.736340	3.438556	-2.021895
31	1	0	1.077790	2.706626	3.173726
32	1	0	1.874507	1.746128	-0.335201
33	1	0	1.260130	2.761509	-3.005597
34	1	0	0.801116	2.728877	1.544092
35	6	0	-3.492798	-2.216883	0.593230
36	1	0	-4.127661	-2.980872	0.154620
37	1	0	-2.597617	-2.675992	1.016032
38	1	0	-4.039992	-1.680391	1.369387
39	6	0	0.842031	-4.065738	-0.693984
40	1	0	-0.112689	-3.761000	-1.122991
41	1	0	1.623785	-4.051651	-1.453287
42	1	0	0.758405	-5.056648	-0.260001
43	8	0	-3.145392	-1.337824	-0.474793
44	8	0	1.191763	-3.198973	0.390834

Most stable energy, Gibbs free energy (Ha), and geometry for protomer ./OMe_Py2N2//2_5
E: -992.081280
G: -991.744503
Geometry:
Input orientation:

Center Number	Atomic Number	Atomic Type	Coordinates (Angstroms) X	Y	Z
1	6	0	-1.366042	1.927617	0.141876
2	6	0	-1.662548	1.118125	1.258122
3	6	0	-1.865553	-0.214524	1.073581
4	7	0	-1.823114	-0.720121	-0.178960
5	6	0	-1.461783	-0.007490	-1.248142
6	6	0	-1.241524	1.347071	-1.120398
7	6	0	-1.285240	-0.777035	-2.532775
8	7	0	-0.920818	-2.162087	-2.266571
9	6	0	0.532556	-2.378191	-2.182794
10	6	0	1.119352	-1.596136	-1.036512
11	7	0	0.696076	-1.916960	0.197010
12	6	0	1.036899	-1.122853	1.199654
13	6	0	1.867169	-0.016943	1.073224
14	6	0	2.361256	0.267597	-0.193796
15	6	0	1.965416	-0.534336	-1.269216
16	6	0	0.436467	-1.456202	2.545251
17	7	0	-0.896706	-2.104894	2.364682
18	6	0	-2.064953	-1.176181	2.203469
19	1	0	-1.682696	1.547767	2.249462
20	1	0	-0.943092	1.910480	-1.991983
21	1	0	-0.552868	-0.241176	-3.145425
22	1	0	-2.237631	-0.754386	-3.065631

23	1	0	1.036843	-2.078702	-3.106380
24	1	0	0.701661	-3.443552	-2.025318
25	1	0	2.084166	0.596969	1.934947
26	1	0	2.287082	-0.286430	-2.271979
27	1	0	1.063851	-2.169683	3.077888
28	1	0	0.305974	-0.567698	3.159577
29	1	0	-1.095374	-2.698566	3.173748
30	1	0	-2.173085	-0.630288	3.136026
31	1	0	-2.941179	-1.800664	2.037080
32	1	0	-1.888659	-1.729173	-0.334513
33	1	0	-0.820699	-2.721940	1.543675
34	1	0	-1.289139	-2.746630	-3.006000
35	6	0	-0.818855	4.076008	-0.689969
36	1	0	-1.602603	4.066543	-1.447308
37	1	0	-0.728920	5.066163	-0.255520
38	1	0	0.133171	3.766602	-1.121574
39	6	0	3.523782	2.178472	0.589805
40	1	0	4.066205	1.635440	1.364769
41	1	0	4.166463	2.935633	0.150735
42	1	0	2.634349	2.647232	1.014131
43	8	0	-1.170405	3.210350	0.395135
44	8	0	3.165067	1.304015	-0.478237

Most stable energy, Gibbs free energy (Ha), and geometry for protomer ./OMe_Py2N2//2_4
E: -992.081277
G: -991.744313
Geometry:
Input orientation:

Center Number	Atomic Number	Atomic Type	Coordinates (Angstroms) X	Y	Z
1	6	0	-2.358973	-0.253316	-0.201139
2	6	0	-1.863997	0.000712	1.072069
3	6	0	-1.028539	1.099450	1.223207
4	7	0	-0.682234	1.913142	0.238300
5	6	0	-1.105346	1.620945	-1.002277
6	6	0	-1.957365	0.569256	-1.258681
7	6	0	-0.513574	2.425725	-2.130190
8	7	0	0.940336	2.213275	-2.211332
9	6	0	1.307439	0.835505	-2.509454
10	6	0	1.471340	0.032527	-1.243794
11	7	0	1.835869	0.713338	-0.154718
12	6	0	1.869267	0.173536	1.083789
13	6	0	1.652013	-1.161240	1.232558
14	6	0	1.349634	-1.937471	0.094849
15	6	0	1.236438	-1.322419	-1.151957
16	6	0	2.070772	1.103067	2.239619
17	7	0	0.911122	2.040233	2.411483
18	6	0	-0.429168	1.401708	2.576423
19	1	0	-2.084296	-0.630531	1.920309
20	1	0	-2.279603	0.344989	-2.266786
21	1	0	-0.684818	3.487594	-1.952559
22	1	0	-1.013235	2.144291	-3.061973
23	1	0	2.265217	0.826786	-3.033024
24	1	0	0.581498	0.315778	-3.143251
25	1	0	1.663888	-1.616805	2.212370
26	1	0	0.935297	-1.859929	-2.038753
27	1	0	2.163479	0.532689	3.159044

28	1	0	2.955328	1.721552	2.096664
29	1	0	0.843679	2.671342	1.600352
30	1	0	-1.050893	2.111881	3.119970
31	1	0	-0.309427	0.500998	3.174942
32	1	0	1.914222	1.725290	-0.281561
33	1	0	1.112022	2.619430	3.230413
34	1	0	1.311528	2.815435	-2.935067
35	6	0	-3.536595	-2.171501	0.541229
36	1	0	-4.186108	-2.913296	0.086270
37	1	0	-2.651893	-2.657512	0.955911
38	1	0	-4.074342	-1.639698	1.327145
39	6	0	0.770098	-4.053687	-0.794612
40	1	0	-0.176930	-3.716867	-1.216592
41	1	0	1.553391	-4.037212	-1.552238
42	1	0	0.663773	-5.053602	-0.387014
43	8	0	-3.169116	-1.278310	-0.508209
44	8	0	1.136470	-3.223730	0.313266
